# Identification of 113 new histone marks by CHiMA, a tailored database search strategy

**DOI:** 10.1126/sciadv.adf1416

**Published:** 2023-04-05

**Authors:** Jinjun Gao, Xinlei Sheng, Jianfeng Du, Di Zhang, Chang Han, Yue Chen, Chu Wang, Yingming Zhao

**Affiliations:** ^1^ Ben May Department for Cancer Research, The University of Chicago, Chicago, IL 60637, USA.; ^2^ State Key Laboratory of Protein and Plant Gene Research, School of Life Sciences, Peking University, Beijing 100871, China.; ^3^ Peking–Tsinghua Center for Life Sciences, Academy for Advanced Interdisciplinary Studies, Peking University, Beijing 100871, China.; ^4^ Department of Biochemistry, Molecular Biology and Biophysics, The University of Minnesota at Twin Cities, Minneapolis, MN 55455, USA.; ^5^ Synthetic and Functional Biomolecules Center; Beijing National Laboratory for Molecular Sciences; Key Laboratory of Bioorganic Chemistry and Molecular Engineering of the Ministry of Education; College of Chemistry and Molecular Engineering, Peking University, Beijing 100871, China.

## Abstract

Shotgun proteomics has been widely used to identify histone marks. Conventional database search methods rely on the “target-decoy” strategy to calculate the false discovery rate (FDR) and distinguish true peptide-spectrum matches (PSMs) from false ones. This strategy has a caveat of inaccurate FDR caused by the small data size of histone marks. To address this challenge, we developed a tailored database search strategy, named “Comprehensive Histone Mark Analysis (CHiMA).” Instead of target-decoy–based FDR, this method uses “50% matched fragment ions” as the key criterion to identify high-confidence PSMs. CHiMA identified twice as many histone modification sites as the conventional method in benchmark datasets. Reanalysis of our previous proteomics data using CHiMA led to the identification of 113 new histone marks for four types of lysine acylations, almost doubling the number of previously reported marks. This tool not only offers a valuable approach for identifying histone modifications but also greatly expands the repertoire of histone marks.

## Teaser

Histone mark identification can be greatly improved by a tailored database search strategy.

## INTRODUCTION

Protein posttranslational modifications (PTMs) are widely known as a mechanism for regulating protein structure and function. PTM reactions can occur through intrinsic chemical reactivity (*1*, *2*) or enzyme-catalyzed reactions (*3*), which use highly reactive metabolic end-products or activated intermediates (*4*). For example, acetyl–coenzyme A and *S*-adenosylmethionine can be used as substrates by acetyltransferases [for lysine acetylation (Kac)] and methyltransferases (for lysine methylation) (*3*, *5*), respectively. l-lactate, traditionally known as a metabolic waste product, has recently been found to stimulate and be a precursor for lysine l-lactylation, most likely via l-lactyl–coenzyme A (*6*).

PTMs on histones, or histone marks, play a key role in chromatin structure and function (*7*). Dysregulation of histone PTMs can contribute to physiological changes and diseases such as cancers and neurological disorders (*8*, *9*). The first steps toward studying the functions of histone marks are to characterize which residues are modified by PTMs and how changes in histone marks correlate with biological outcomes. To this end, analytical tools and reagents for identifying and quantifying histone marks are critical to epigenetics studies (*10*).

Mass spectrometry (MS)–based shotgun proteomics has become the method of choice for identifying and quantifying histone marks. In shotgun proteomics, proteolytic peptides, with or without enrichment by modification-specific antibodies, are subjected to liquid chromatography (LC)–MS/MS analysis. By matching the measured mass/charge ratio (*m*/*z*) of precursors and their associated fragment ions with theoretical values derived from a protein library, computational methods can determine the peptide sequence and locate the PTM site(s) in the peptide, a process also known as a “database search” (*11*, *12*).

During a database search, the first step is to match experimentally recorded MS/MS spectra with in silico simulated spectra of theoretical peptides derived from the protein database. As this step may introduce false-positive identifications, differentiating true peptide-spectrum matches (PSMs) from false ones is critical. The most commonly used strategy is the “target-decoy” approach (*13*), in which MS/MS spectra are used to search a database containing an equal number of “targets” (true protein sequences) and “decoys” (usually the reversed sequences). While the target-decoy approach is powerful for whole proteome data, it has limitations when analyzing histone marks. In a typical pipeline for identifying histone marks, histone proteins are extracted and proteolytically digested, followed by LC-MS/MS analysis. The resulting datasets are relatively small, with only hundreds or even dozens of histone peptides after enrichment with an anti-PTM antibody. When we examined four datasets previously generated in our group using the target-decoy strategy and applied a 1% false discovery rate (FDR), we failed to identify a substantial proportion (ranging from 12.5 to 36.4%) of histone modification sites. Because the score distributions of target and decoy hits are almost identical when small MS/MS datasets are analyzed, it is challenging to calculate a reliable threshold to differentiate positive identifications from false ones in the target-decoy search strategy.

**Fig. 1.**
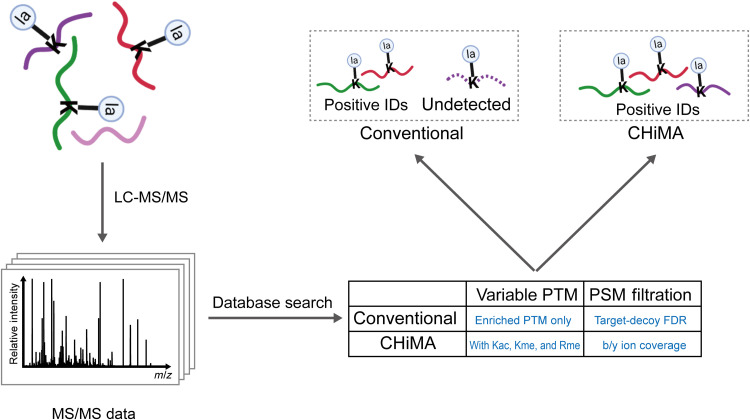
Schematic workflows of a conventional database search strategy and CHiMA. As compared to the conventional strategy, CHiMA uses b/y ion coverage as the key criterion for PSM filtration and includes multiple background variable modifications during database search.

In this study, we developed a tailored database search strategy, named “Comprehensive Histone Mark Analysis (CHiMA),” for identifying histone marks (Fig. 1). Instead of using the target-decoy FDR, CHiMA assigns high-confidence PSMs if at least 50% of the expected b or y fragment ions are observed in the acquired MS/MS spectrum. In addition, Kac, lysine monomethylation (Kme1), and arginine monomethylation (Rme1) are included as possible modifications along with the modification of interest during the database search. We benchmarked the performance of CHiMA and showed that this approach could identify twice as many peptides bearing histone marks as the conventional strategy. Using CHiMA, we identified 113 previously unreported histone marks in the examined databases including lysine lactylation (Kla), crotonylation (Kcr), 2-hydroxyisobutyrylation (Khib), and benzoylation (Kbz). These unreported histone marks were validated by MS/MS analysis of synthetic peptides. These results suggest that CHiMA could serve as a valuable analytical tool for identification and characterization of epigenetic histone marks.

## RESULTS

### The target-decoy strategy fails to identify some histone marks

To investigate possible limitations of the conventional database search strategy for identifying histone marks in small datasets, we reanalyzed four MS/MS datasets, namely “datasets A to D,” which were previously generated to quantify histone deacetylase 3 (HDAC3)–mediated histone delactylation (*14*). In that study, stable isotope labeling by amino acids in cell culture (SILAC) was used to generate four quantitative proteomics datasets derived from immunoprecipitated histone peptides containing Kla. Histones were prepared using one of the following four workflows: (i) Histones were extracted from HeLa cells cultured in “heavy” and “light” SILAC media, followed by incubation with or without recombinant HDAC3, respectively (dataset A); (ii) the same experiment that generated dataset A but with the reverse labeling (dataset B); (iii) histones were extracted from HeLa cells cultured in light and heavy SILAC media, followed by incubation with or without dimethyl sulfoxide, respectively (dataset C); and (iv) the same experiment that generated dataset C but with the reverse labeling (dataset D). These four datasets were generated using identical analytical conditions including the analytical column, the high-performance LC (HPLC) gradient, and the mass spectrometric parameters (*14*). Therefore, we expected each specific Kla-containing peptide to elute at similar retention times, which enables detection of missed Kla peptides by cross-referencing the four datasets.

**Fig. 2.**
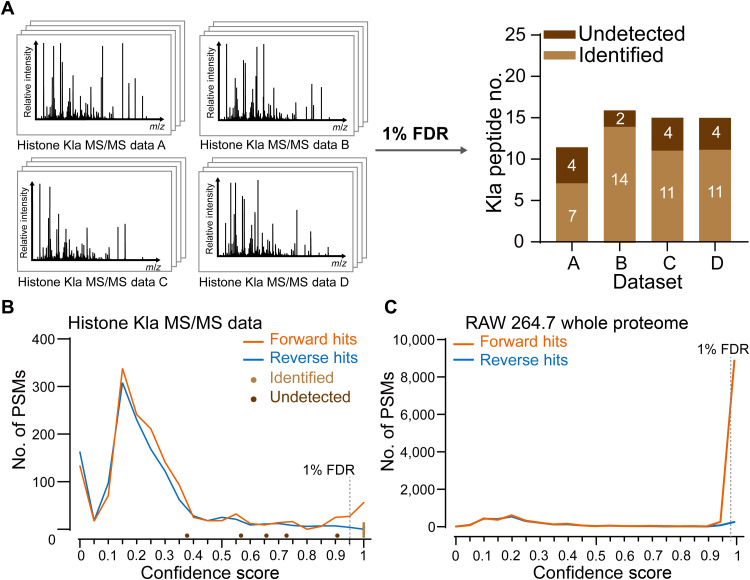
Undetected histone marks by target-decoy FDR filtration of PSMs. (**A**) Schematic showing the conventional database search method using 1% FDR cutoff. Four MS/MS datasets were used to assess how many modified peptides were undetected by the conventional database search approach. PSMs were filtered with a defined peptide FDR of 1% based on the target-decoy strategy. Missed identifications in each individual dataset were deduced by referencing the identified Kla peptides across the datasets, judging by their *m*/*z*, retention time, and MS/MS spectra. (**B**) Confidence score distributions for forward and reverse hits from dataset A. Target hits (forward hits, orange) and decoy hits (reverse hits, blue) showed similar score distributions. Each light and heavy brown dot represents a Kla peptide that was identified and missed, respectively, by the conventional strategy. Five missed Kla peptides correspond to four sites. The manually calculated 1% FDR threshold was indicated by a dashed gray line. (**C**) Confidence score distributions of target (orange) and decoy (blue) hits for the whole proteome of the RAW 264.7 cell line. The manually calculated 1% FDR threshold was indicated by a dashed gray line.

To examine the degree to which conventional methods failed to identify modified peptides, we analyzed these four MS/MS datasets (Fig. 2A, left), using ProLuCID for peptide-spectrum matching and DTASelect 2.0 for PSM filtration (*15*), with 1% FDR. We manually scrutinized the peptide identifications to remove false positives as previously described (*16*). All peptides identified from the four datasets were combined to generate a comprehensive list of detected Kla peptides. To identify Kla peptides that the conventional analysis methods failed to detect, we compared the identifications from each individual dataset with the “comprehensive” list to obtain the Kla peptides that were missed in each dataset. Each of the missed peptides was validated by manually locating the corresponding MS/MS spectrum and visually examining its precursor and fragment ions. This analysis enabled us to calculate the corresponding “loss rates” in each dataset, which are defined as the ratio of the number of “missed” Kla peptides divided by the total number of verified Kla peptides.

The loss rates of these four datasets ranged from 12.5 to 36.4% (Fig. 2A, right). Unexpectedly, the overall PSM quality of the missed Kla peptides was similar to that of the detected peptides (fig. S1A). To examine whether this phenomenon was specific to ProLuCID, we obtained similar results using Andromeda (*17*), another algorithm for peptide-spectrum matching, but identified a different set of missed Kla peptides. Thus, our result suggests that the missed identification was not specific to a peptide-spectrum matching algorithm (fig. S1B) and that identification of histone marks by the target-decoy strategy may be stochastic.

### Implementation of FDR has limitations for analyzing small-scale datasets

We next examined the mechanisms underlying the failure to detect certain Kla peptides when the target-decoy strategy was used. This strategy relies heavily on differentially distributed target and decoy PSM scores to determine a proper score threshold. When histone modifications are searched by this strategy, only a small number of modified histone peptides are enriched so that the number of PSMs is often orders of magnitude smaller than that in whole proteome samples, which may compromise the power of FDR. Thus, we hypothesized that the distributions of target and decoy PSM scores would be indistinguishable when small datasets such as those described above are analyzed. To test this hypothesis, we manually verified all the Kla PSMs that were missed by the target-decoy strategy. We found that these missed peptides were indeed excluded by FDR-based filtering despite that they could be successfully matched to their corresponding MS/MS spectra (table S1 and data S1).

We next compared the distributions of the confidence scores, the major metric used by DTASelect to assess PSM confidence (*18*). We used the first of the four test datasets (dataset A) for the analysis. Consistent with our hypothesis, the target and decoy PSMs for dataset A showed nearly identical distributions for the confidence scores (Fig. 2B). The highest score for the PSMs of reversed peptides is 0.95, while only 65 PSMs for the forward peptides were scored higher above this cutoff. Since the cutoff score for 1% FDR was entirely determined by the highest scored reverse PSM under this condition, it can be quite random and lose discriminating power. Similar score distributions were also observed using Mascot (*19*), in which the highest scored PSM for the reversed peptides ranked 60th, showing that this phenomenon was independent of the scoring algorithm (fig. S2). In contrast, the score distributions of target and decoy PSMs using the whole proteome derived from Raw 264.7 cells were well separated (Fig. 2C), which allows the search engine to determine an appropriate cutoff to achieve targeted FDR values. Therefore, for small datasets such as those derived from modified histone peptides, the distributions hold less statistical power. These observations suggest that the target-decoy strategy might consistently fail to identify some modified peptides in a small dataset, even if those peptides give rise to good MS/MS spectra.

### Fragment ion coverage–based PSM filtration improves identification of histone marks

To recover the missed histone marks, we optimized the PSM filtering process. It has been demonstrated that manual examination of PSMs is helpful to verify peptide identification when very small datasets are analyzed (*20*). In addition, a credible PSM should be adequately explained by the observed fragment ions (*16*). On the basis of the rationale from these two studies, we reasoned that the “fragment ion coverage (FIC),” or the percentage coverage calculated by the number of observed b and y fragment ions divided by the number of theoretical b and y fragment ions, can serve as a valid criterion for high-quality PSMs.

**Fig. 3.**
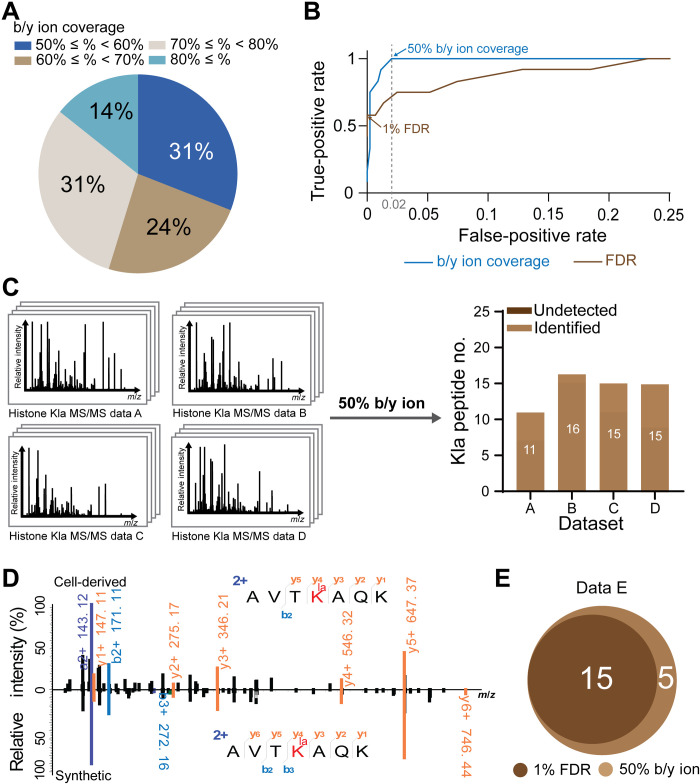
Optimization of filtration strategy to improve comprehensiveness of identifications by leveraging FIC. (**A**) Pie chart of the FICs for all Kla peptides identified by the conventional strategy. Peptides from all four test datasets were combined. (**B**) The ROC curves of FDR (brown) and b/y ion coverage strategies (blue) for dataset A. The blue and brown arrows indicate 50% b/y ion coverage and 1% FDR, respectively. Dashed gray line indicates false-positive rate of 0.02. (**C**) Using 50% b/y ion coverage as the criterion to select true positives recovered all Kla peptides that were undetected by 1% FDR. (**D**) Representative MS/MS spectra of cell-derived and synthetic PSMs corresponding to a Kla peptide recovered from dataset A by the 50% b/y ion coverage strategy. (**E**) Venn diagram of Kla peptides identified in dataset E by distinct PSM filtration criteria, i.e., 1% FDR and 50% b/y ion coverage.

To test this hypothesis, we calculated FICs for each of the identified Kla peptides from the four abovementioned datasets. All identified Kla peptides had at least 50% coverage of b/y fragment ions (Fig. 3A). Receiver operating characteristic (ROC) plot is a common method for comparing the discrimination ability of different classifiers. In ROC curves, one can read the false-positive rate to obtain any given true-positive rate. We next used the dataset A to plot ROC curves for both the conventional FDR-based and the FIC-based strategies, considering the validated Kla peptides as true positives and peptides identified from the reversed proteins as false positives. The curves indicate the true-positive and false-positive rates using various cutoff values including FDR values from 1 to 100% and fragment coverage values from 100 to 0% (from the strictest to the loosest), respectively (Fig. 3B and table S2). The ROC curves clearly demonstrated the improvement in terms of sensitivity by the FIC-based strategy as compared to that by the FDR-based strategy. For example, an FIC percentage cutoff of 50% could pick out all the true positives with the false-positive rate of only 2%, while, under the similar false-positive rate, the FDR-based approach could only achieve a true-positive rate of 75% (Fig. 3B). Therefore, we applied a FIC percentage cutoff of 50%, instead of 1% FDR, to filter PSMs in the four datasets. Using this optimized filtering method, we not only kept all previously identified Kla peptides but also recovered all the ones that had been missed (Fig. 3C). To evaluate the reliability of this approach, we chemically synthesized all the five missed Kla peptides that were recovered from dataset A (table S3). The MS/MS spectra of the synthesized peptides aligned well with the spectra derived from the endogenous peptides in dataset A (Fig. 3D and fig. S3), validating the identities of the recovered peptides.

To test whether the FIC-based strategy is generally applicable, we applied it to another MS/MS dataset (dataset E) that was generated from the enriched Kla peptides prepared by a different workflow (*6*). While 15 Kla sites were identified by the conventional FDR-based strategy using 1% as the cutoff, 5 additional Kla peptides were detected by the FIC-based approach with the 50% FIC, calculating to an increase of 33% (Fig. 3E). The additional Kla peptides were all manually verified (see MS/MS spectra in data S1).

### Including coexisting histone PTMs within a single peptide identifies more histone marks

**Fig. 4.**
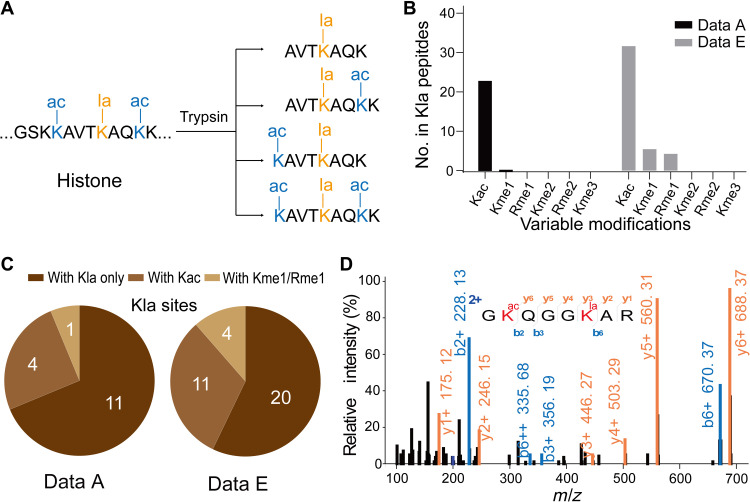
Optimization of database search parameters to identify coexisting histone marks within a single peptide. (**A**) A hypothetical example demonstrating that inclusion of arginine and lysine modifications can help the identification of histone marks. The sequence GSKK(ac)AVTK(la)AQK(ac)K may generate four forms of peptides bearing Kla, three of which contain Kac at nearby lysine sites. Considering Kac as an additional variable modification during database searching can increase the possibility of identifying this histone Kla mark. (**B**) The most common histone modifications, Kac, Kme1, Rme1, lysine dimethylation (Kme2), arginine dimethylation (Rme2), and lysine trimethylation (Kme3), were incorporated into the search strategy as variable modifications to determine their cooccurrence with the target PTM (Kla) in the same peptide. This analysis was conducted on two representative datasets (A and E). The numbers of these variable modifications coexisting with Kla on identified peptides are shown as a histogram. (**C**) Pie charts of identified Kla sites in datasets A and E ascribed to Kla only, Kla + Kac, and Kla + Kme1 or Rme1. (**D**) Representative MS/MS spectrum of a peptide bearing H2AK9la, which was identified in dataset E only when Kac was considered in the database search.

Cross-talk between different PTMs is a common theme for the most frequently studied histone marks (*21*). Understanding coexisting PTMs, particularly in a restricted peptide region, is critical to chromatin regulation and epigenetic mechanisms. Therefore, we proposed that inclusion of a few common arginine and lysine modifications as coexisting PTMs would enhance the identification of peptides bearing histone marks (Fig. 4A).

To test this hypothesis, we reanalyzed datasets A and E by filtering PSMs using the FIC-based strategy and incorporating the six most common histone marks (Kac, Kme1, and Rme1, dimethylation on K and R, and trimethylation on K) as variable modifications. We found that Kac was the most common modification coexisting with Kla in these test datasets, followed by Kme1 and Rme1 (Fig. 4B). On the basis of these observations, we reasoned that integrating these three most common coexisting modifications (Kac, Kme1, and Rme1) into the database searching should markedly improve Kla identification. Upon inclusion of variable acetylation, the number of identified Kla sites in datasets A and E went from 11 and 20 to 15 and 31, respectively (Fig. 4, C and D). When Kac, Kme1, and Rme1 were all included, we identified 16 and 35 Kla sites in the two databases, respectively, corresponding to an increase of 45 and 75%. MS/MS spectra of the peptides bearing new Kla marks are listed in data S2.

### CHiMA detects more histone Kla sites in benchmark datasets

We integrated the FIC-based PSM filtering and the optimized database search parameters as described above into a data analysis pipeline tailored for histone marks. In the so-called CHiMA strategy (Fig. 1), high-confidence PSMs were selected for manual validation if the observed spectrum matched at least 50% of b and y fragment ions in the theoretical spectrum, which enables identification of modified histone peptides as long as they can yield MS/MS spectra with sufficient daughter ions. In addition, Kac, Kme1, and Rme1 were included as background variable modifications during the database search, which allows identification of peptides bearing multiple PTMs.

**Fig. 5.**
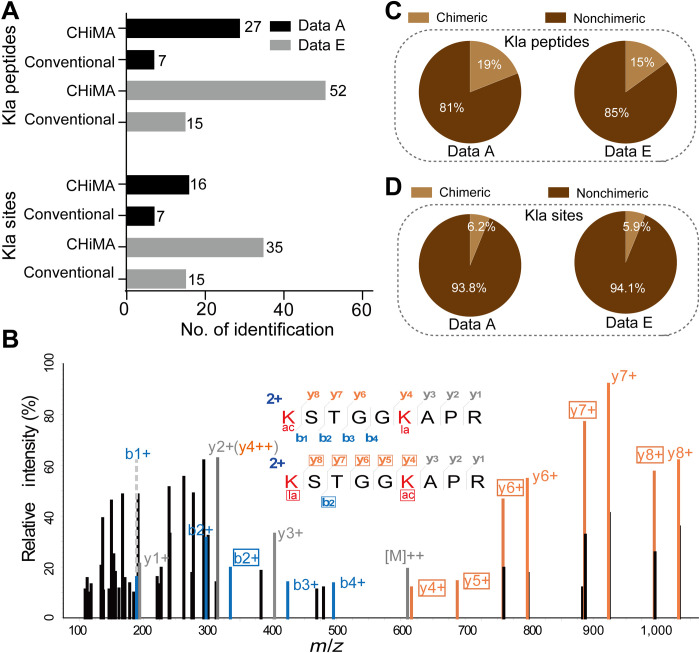
Improved Kla identification by CHiMA. (**A**) Numbers of identified Kla peptides and sites in datasets A and E using either the conventional strategy or CHiMA. (**B**) A representative chimeric MS/MS spectrum containing isomeric peptides. The two isomeric peptides K(la)STGGK(ac)APR and K(ac)STGGK(la)APR are identical except for the positions of acetylation and lactylation. Fragment ions from both isomeric peptides were captured in this spectrum. The unique daughter ions derived from K(ac)STGGK(la)APR are labeled orange (y ions) and blue (b ions); the unique daughter ions derived from K(la)STGGK(ac)APR are labeled by boxes. Shared daughter ions are labeled gray. (**C**) Percentage of Kla peptides identified from chimeric and nonchimeric MS/MS spectra from datasets A and E. (**D**) Percentage of Kla sites additionally identified from chimeric MS/MS spectra from datasets A and E.

In a pilot experiment, we analyzed MS/MS datasets A and E using CHiMA. While the conventional database search strategy identified 7 and 15 Kla sites (from 7 and 15 peptides) in datasets A and E, respectively, CHiMA led to identification of 16 and 35 Kla sites (from 27 and 52 peptides), respectively, from the same datasets, representing increases in Kla site identification of 129 and 133% (Fig. 5A).

### CHiMA allows identification of chimeric spectra containing isomeric peptides

A careful manual examination of the Kla peptides identified by CHiMA uncovered a number of chimeric MS/MS spectra, which were generated by coeluted isomeric peptides that have identical amino acid sequences, but different PTMs at several lysine residues. These MS/MS spectra typically have lower PSM scores due to interfering fragment ions derived from the isomeric peptide. For example, a chimeric MS/MS spectrum was generated by two isomeric peptides, K(ac)STGGK(la)APR and K(la)STGGK(ac)APR (Fig. 5B). The PSM confidence score of K(ac)STGGK(la)APR was low because many high-intensity peaks were derived from the fragment ions of its isomeric counterpart, K(la)STGGK(ac)APR. If both chimeras were considered, then those previously unmatched fragment ion peaks could be perfectly explained so that the confidence for the Kla identification could be improved (Fig. 5B). In addition, these chimeric spectra tend to have lower DeltaCN scores because the highest scored PSM is only slightly better than the runner-up. While many mainstream database search engines reject these chimeric spectra with low DeltaCN scores as false-positive hits, CHiMA specifically counts the number of matched fragment ions in chimeric PSMs, resulting in matches to the target isomeric peptides and identification of more Kla peptides. We found that 19 and 15% of the Kla peptides had chimeric spectra in datasets A and E, respectively (Fig. 5C), dissection of which using CHiMA resulted in additional 6.2 and 5.9% identified Kla sites (Fig. 5D). Identification of these isomeric peptides from chimeric spectra by CHiMA should help us to further understand the coexistence of histone marks.

### CHiMA found 113 unreported histone marks in previous proteomic datasets

**Fig. 6.**
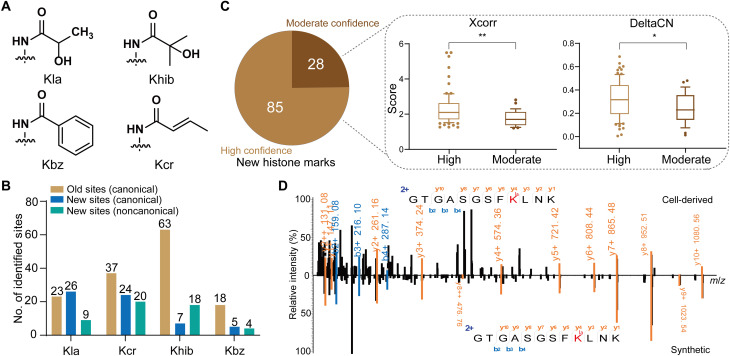
Identification of an additional 113 histone marks in previously analyzed MS/MS datasets by CHiMA. (**A**) Chemical structures of four types of lysine acylation: Kla, Khib, Kbz, and Kcr. (**B**) Number of histone marks identified in previous studies (*6*, *23*, *25*) and in this study by CHiMA. Newly identified histone marks by CHiMA are classified into two groups depending on their presence on the canonical replication–dependent histones or noncanonical replication–independent histone variants. (**C**) Classification of the newly identified histone marks by confidence. The box and whisker plots show the Xcorr and DeltaCN scores for high-confidence and moderate-confidence peptides. Statistical differences were examined by unpaired Student's t-test. Symbols indicate statistical significance (*P < 0.05, **P < 0.01). (**D**) Representative MS/MS spectra of cell-derived and synthetic PSMs corresponding to a Kla peptide [GTGASGSFK(la)LNK] identified with moderate confidence. Despite the high-intensity noise peaks, good b/y ion coverage was observed, and the ion intensities derived from the cell-derived and synthetic peptides were well correlated.

To demonstrate the power of CHiMA in identifying histone marks, we applied this method to the analysis of several previous proteomic datasets, which were generated to identify histone lysine acylation marks, including 4 datasets for Kla (*6*), 14 datasets for Kcr (*22*), 4 datasets for Kbz (*23*), and 12 datasets for Khib (*24*) (Fig. 6A).

Both the conventional strategy and CHiMA were applied on these datasets to compare their performance in identification of the four types of histone marks. In general, CHiMA can identify more modification sites than the conventional strategy (fig. S4). For the few histone marks that were uniquely identified by the conventional strategy, we confirmed by manual verification that they had low confidence PSMs and were very likely to be false positives (data S3).

We then compared the identified histone marks by CHiMA with the previously published maps to findunreported histone marks. In total, CHiMA found 113 previously unreported histone marks bearing the four lysine acylations on “canonical” replication–dependent histones and “noncanonical” replication–independent histone variants. More specifically, 26 unreported Kla sites were found on canonical histones using CHiMA, as compared to the originally reported 23 Kla sites (*6*); 9 more Kla sites were identified in addition to the 3 previously reported sites on noncanonical histone variants (Fig. 6B). Similarly, we identified 44 additional Kcr sites (24 on canonical histones and 20 on variants) and 25 additional Khib sites (7 on canonical histones and 18 on variants) (Fig. 6B) (*25*). Last, nine additional Kbz sites (five on canonical histones and four on variants) were identified as compared to previously reported Kbz marks (*23*).

We classified the 113 newly identified histone marks into two categories (Fig. 6C). The first category included the high-confidence PSMs that met the following criteria: (i) There were no obvious unmatched fragment ion peaks with larger *m*/*z* than the precursor ion, (ii) most fragment ions had a reasonable isotope distribution, and (iii) there were no noise peaks with a larger *m*/*z* than the largest fragment ion (*16*). The second category contained PSMs with moderate quality that had good coverage of fragment ions but exhibited low peak intensities or unexplained peaks. In general, the PSMs with moderate confidence had lower scores in Xcorr and/or DeltaCN than those with high confidence (Fig. 6C). To verify these moderate-confidence identifications, all newly identified Kla and Kcr peptides were chemically synthesized (table S3), and their MS/MS spectra were compared with those of the corresponding endogenous peptides (Figs. 3D and 5D, fig. S3, and data S4). The MS/MS spectra derived from all peptide pairs matched well, suggesting that these histone marks newly identified by CHiMA are bona fide PTMs on cellular histone proteins. Including several common lysine and arginine PTMs as background variable modifications not only helped identify more histone marks but also led to identification of coexisting histone marks. We summarized all the coexisting histone marks from the peptides bearing multiple histone marks (table S4). All the PSMs were manually checked and reported as data S5. To further validate these identifications, we synthesized several peptides and showed that the synthetic peptides’ MS/MS spectra matched well with their corresponding endogenous peptides (fig. S5).

**Fig. 7.**
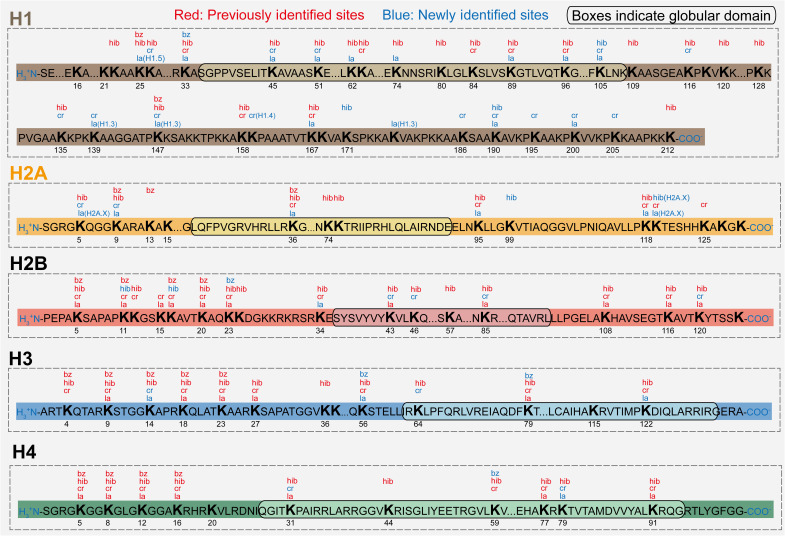
A comprehensive map of Kla, Kbz, Kcr, and Khib on canonical histone proteins. A total of 203 histone marks were detected on canonical replication–dependent histones, including 62 newly identified marks. H1.2, H2A1, H2B1C, H3.1, and H4 were selected as representative canonical replication–dependent histones. Histone marks detected only on homologous sites of other histone subtypes were also integrated, with their associated histone variant indicated. Notably, although H2A.X is technically regarded as a noncanonical histone variant, it shares high homology with the canonical H2A, with minor differences at the C terminus. Therefore, histone marks identified in the homologous regions of H2A.X were also integrated into H2A.

We then updated the modification map by incorporating all the 113 newly identified histone Kla, Kcr, Khib, and Kbz sites (Fig. 7 and fig. S6). Previous histone mark maps focused on the canonical histones, selecting one subtype for each histone family (*6*, *22*, *23*, *25*, *26*). However, the unique functions of the noncanonical histone variants are beginning to be recognized (*27*). Therefore, H1.2 and H2A1, H2B1C, H3.1, and H4 were selected as representative canonical histones (Fig. 7), and a few noncanonical histones, the sequences of which are largely distinct from the canonical ones, were also displayed (fig. S6). Compared with previous maps of histone marks (*6*, *23*), 113 histone marks were added, with 62 marks on canonical histones and 51 on histone variants. PSMs for all newly identified histone marks can be found in data S6.

## DISCUSSION

In this study, we first examined which step in the database search process is responsible for failure to identify histone marks. This study led to a few interesting observations: (i) The FDR strategy can result in missed identification of peptides when applied to small MS/MS datasets; (ii) some abundant PTMs should be included as variable PTMs when identifying histone marks; and (iii) chimeric MS/MS spectra exist for isomeric peptides bearing two or more PTMs. To address these challenges, we developed CHiMA as a tailored database search strategy to maximize the identification of modified histone peptides. CHiMA uses FIC instead of target-decoy strategy–based FDR to filter out false positives. This feature is beneficial for small MS/MS datasets derived from a limited number of PTM peptides, such as datasets of histone peptides bearing one or a few PTMs. The conventional database search strategy is not ideal for small datasets, while it remains powerful for identification of peptides when a large MS/MS dataset is involved. Moreover, CHiMA is more suitable for short peptides, where only a small number of fragment ions can be generated, hindering reliable identification by target-decoy–based approaches. In addition, CHiMA can identify isomeric peptides bearing two PTMs in the same peptide sequence.

Peptide identification is the focus of CHiMA. Nevertheless, CHiMA can assist quantification of histone marks in two ways: (i) When a peptide bears only one histone mark, the quantification of this mark, by convention, is equivalent to the abundance of the peptide. This quantification strategy, in principle, can be applied to any kind of modifications. The identifications by CHiMA could directly contribute to the quantification of these histone marks. (ii) CHiMA can also aid the quantification of coexisting histone marks. Accurate quantification of histone marks that coexist on a peptide is dependent on the deconvolution of the combinatorial histone PTMs (*28*). CHiMA could be implemented in this workflow to acquire a more complete landscape of histone marks, so that more accurate quantification could be achieved.

Application of CHiMA to our previous MS/MS datasets allowed us to identify 113 previously undescribed histone marks, expanding the repertoire of histone PTMs to an almost doubled level. The reliability of CHiMA for peptide identification was substantiated by MS/MS analysis of synthetic peptides, the gold standard for verifying peptide identification. The identification of an additional 113 Kla sites opens a window for future studies of histone Kla in various physiological contexts. Moreover, this approach can be directly applied to analysis of nonhistone proteins when small MS/MS datasets are involved. Thus, CHiMA offers a valuable tool for studies of both histone and nonhistone modifications.

## MATERIALS AND METHODS

### Synthetic peptides

All modified histone peptides were synthesized by commercial sources with purity of >70%. Peptides were solubilized in mobile phase A (see below) and diluted to the desired concentration for HPLC-MS/MS analysis.

### Cell culture and preparation of protein lysates

RAW 264.7 cells were obtained from the American Type Culture Collection and cultured in Dulbecco’s modified Eagle’s medium supplemented with 10% fetal bovine serum and 1% GlutaMAX (Thermo Fisher Scientific Inc., Waltham, MA, USA). RAW 264.7 cells were harvested by scraping with lysis buffer containing 8 M urea (Sigma-Aldrich, St. Louis, MO, USA) and 1× protease inhibitor cocktail (Roche Diagnostics GmbH, Mannheim, Germany), followed by sonication to completely lyse the cells. Extracted proteins were quantified with the Bradford protein assay (Bio-Rad Laboratories Inc., Hercules, CA, USA). Proteins were then treated with dithiothreitol (Sigma-Aldrich) and iodoacetamide (Sigma-Aldrich), digested with trypsin (1:50; Promega Corp., Madison, WI, USA) for 16.5 hours in 100 mM ammonium bicarbonate (Sigma-Aldrich), and concentrated by vacuum.

### HPLC-MS/MS analysis of synthetic peptides

Peptide samples were loaded onto a home-made silica column (12-cm length × 3-μm inside diameter) packed with C18 resin (Dr. Maisch GmbH, Ammerbuch-Entringen, Germany). LC-MS/MS was performed on an Orbitrap Exploris 480 mass spectrometer (Thermo Fisher Scientific Inc., Waltham, MA, USA) coupled with an EASY-nLC 1000 system (Thermo Fisher Scientific Inc.). Mobile phase A was 0.1% formic acid in water (v/v), and mobile phase B was 0.1% formic acid in acetonitrile (v/v). The eluting flow rate was 0.3 μl/min. Samples were separated and eluted with a gradient of 5 to 35% mobile phase B in A over 20 min for synthetic peptides and over 60 min for RAW 264.7 whole proteome. Under the positive ion mode, full-scan mass spectra were acquired over the *m*/*z* range from 300 to 1400 using the Orbitrap mass analyzer with mass resolution of 120,000. MS/MS fragmentation was performed in a data-dependent mode, in which the 15 most intense ions were selected for MS/MS analysis at a resolution of 30,000 using higher energy collisional dissociation (HCD) collision mode. Other important parameters: isolation window, 2.0 *m*/*z* units; default charge, 2+; normalized collision energy, 30%; maximum injection time (IT), auto; automatic gain control (AGC) target, standard; dynamic exclusion, exclude after two times within 20 s.

### Data analysis

All test datasets were generated in previous studies. Datasets A to D were generated by Moreno-Yruela *et al.* (*14*), and dataset E was generated by Zhang *et al.* (*6*). For test datasets, MS/MS spectra were used to search the reverse-concatenated nonredundant FASTA human database compiled from UniProt (version 2017). Other search parameters and data analysis processes are thoroughly explained in Results. A detailed guidance with example data for CHiMA can be accessed via https://github.com/JinjunGao/CHiMA and https://zenodo.org/record/7686113#.Y_5hPnbMKJY. For the whole-cell lysate derived from RAW 264.7, ProLuCID (*18*) was used to search MS/MS spectra against the reverse-concatenated nonredundant FASTA mouse database compiled from UniProt. One static modification (+57.02147 Da for carbamidomethylation) was set on cysteine. The precursor and fragmentation tolerances were 10 and 40 parts per million, respectively. ProLuCID search results were filtered and assembled by DTASelect 2.0 (*15*) with a defined peptide false-positive rate of 1%.

## Notes

View/request a protocol for this paper from *Bio-protocol*.
