# Supplementary material for: Identification of 113 new histone marks by CHiMA, a tailored database search strategy
Source: Sci Adv. 2023 Apr 5;9(14):eadf1416. doi: 10.1126/sciadv.adf1416 (PMC10075957; doi:10.1126/sciadv.adf1416)
Supplement: Supplementary file 1 — Figs. S1 to S6 Tables S1 to S4 Data S1 to S6 [file sciadv.adf1416_sm.pdf]

Science Advances
AAAS

Supplementary Materials for

Identification of 113 new histone marks by CHiMA, a tailored database search strategy

Jinjun Gao *et al.*

Corresponding author: Chu Wang, chuwang@pku.edu.cn; Yingming Zhao, yingming.zhao@uchicago.edu

Sci. Adv. **9**, eadf1416 (2023)
DOI: 10.1126/sciadv.adf1416

This PDF file includes:

Figs. S1 to S6
Tables S1 to S4
Data S1 to S6

I. Supplemental figures

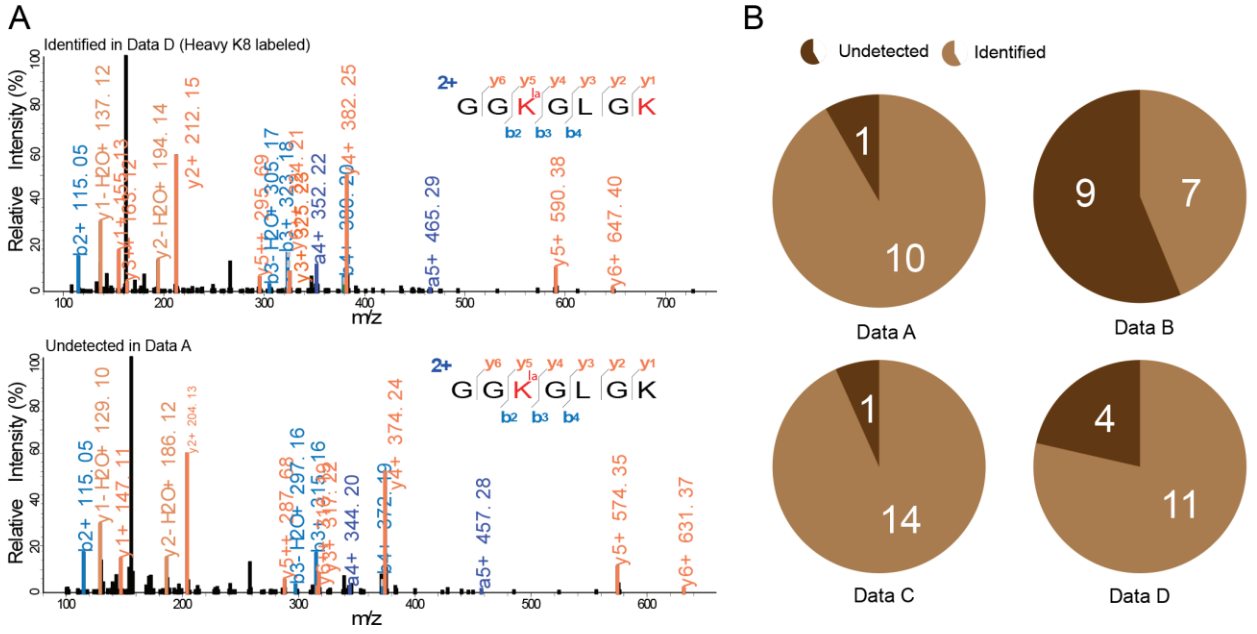

Figure S1, related to Figure 2A. Conventional search strategies fail to detect some Kla peptides. (A) MS/MS spectra of a representative Kla peptide that was detected in Dataset D but not in Dataset A. **(B)** Pie charts of Kla peptides in each dataset that Andromeda failed to detect.

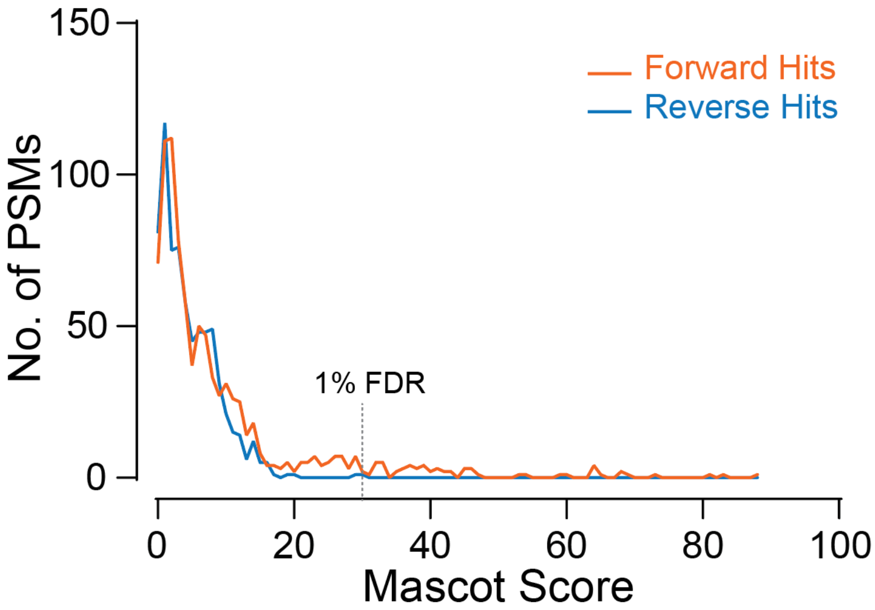

Figure S2, related to Figure 2B. Similar score distributions of target and decoy hits for Dataset A analyzed by MASCOT. Dataset A was searched by Mascot against a reverse-concatenated database. All PSMs were extracted by setting the significance threshold p-value to 1. The score distributions for forward (orange) and reverse PSMs (blue) are similar. The manually calculated 1% FDR threshold was indicated by a dashed gray line. The score of the top-ranked reverse peptide PSM was 30, and only 59 forward peptide PSMs scored larger than this threshold.

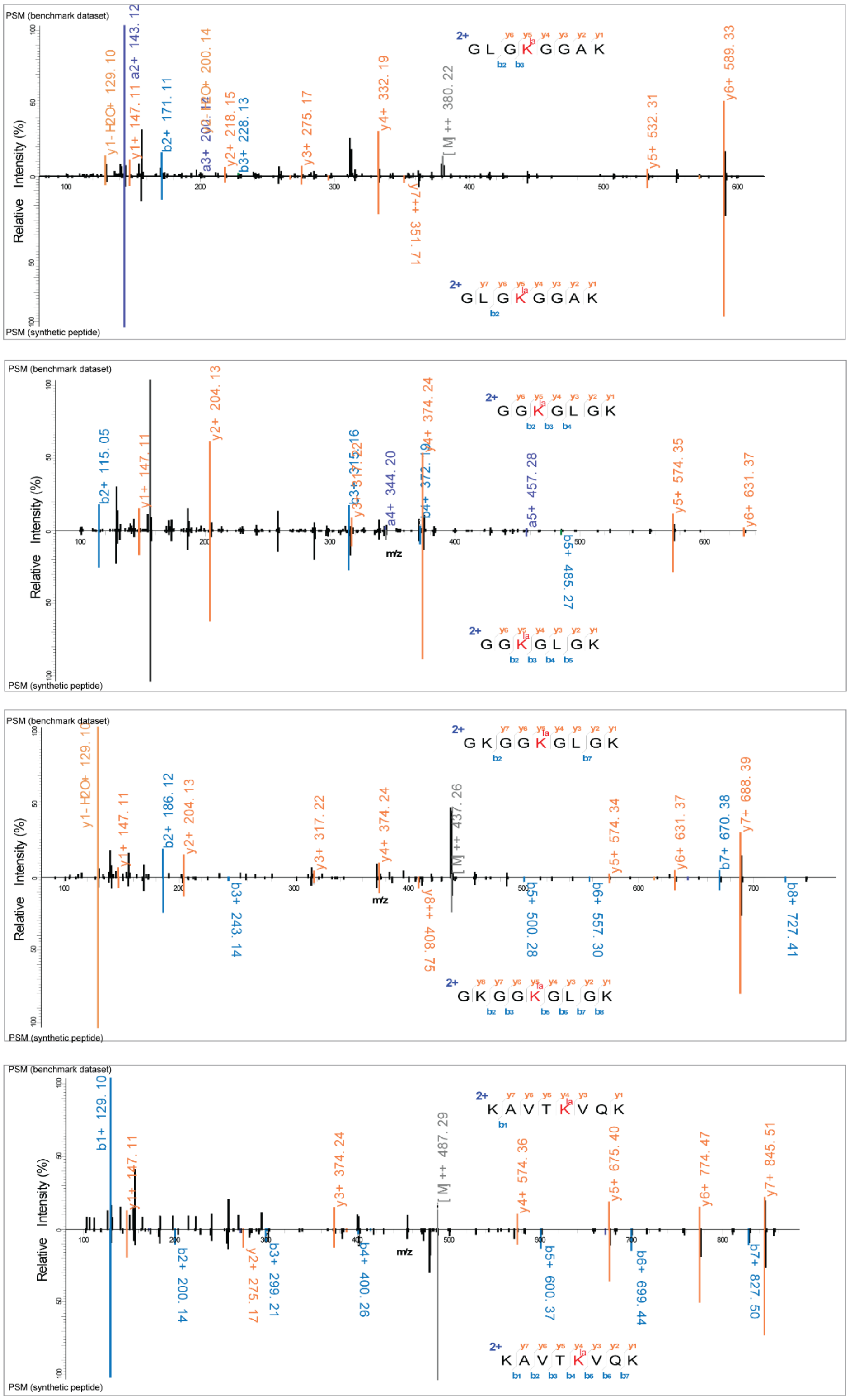

Figure S3, related to Figure 3D. The MS/MS spectra of Kla peptides identified in Dataset A by the 50% b/y ion coverage strategy perfectly match those of the corresponding synthetic peptides.

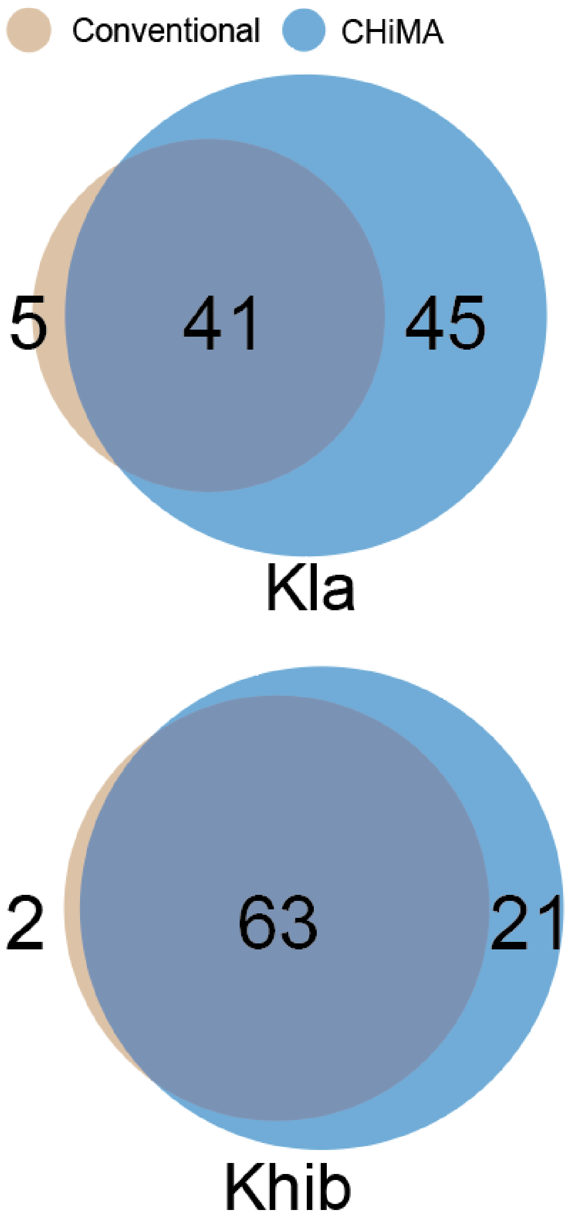

Figure S4, related to Figure 6. **Venn diagrams of modification sites identified by conventional strategy and CHiMA for analyzing previous Kla, Kbz, Khib, and Kcr datasets.**

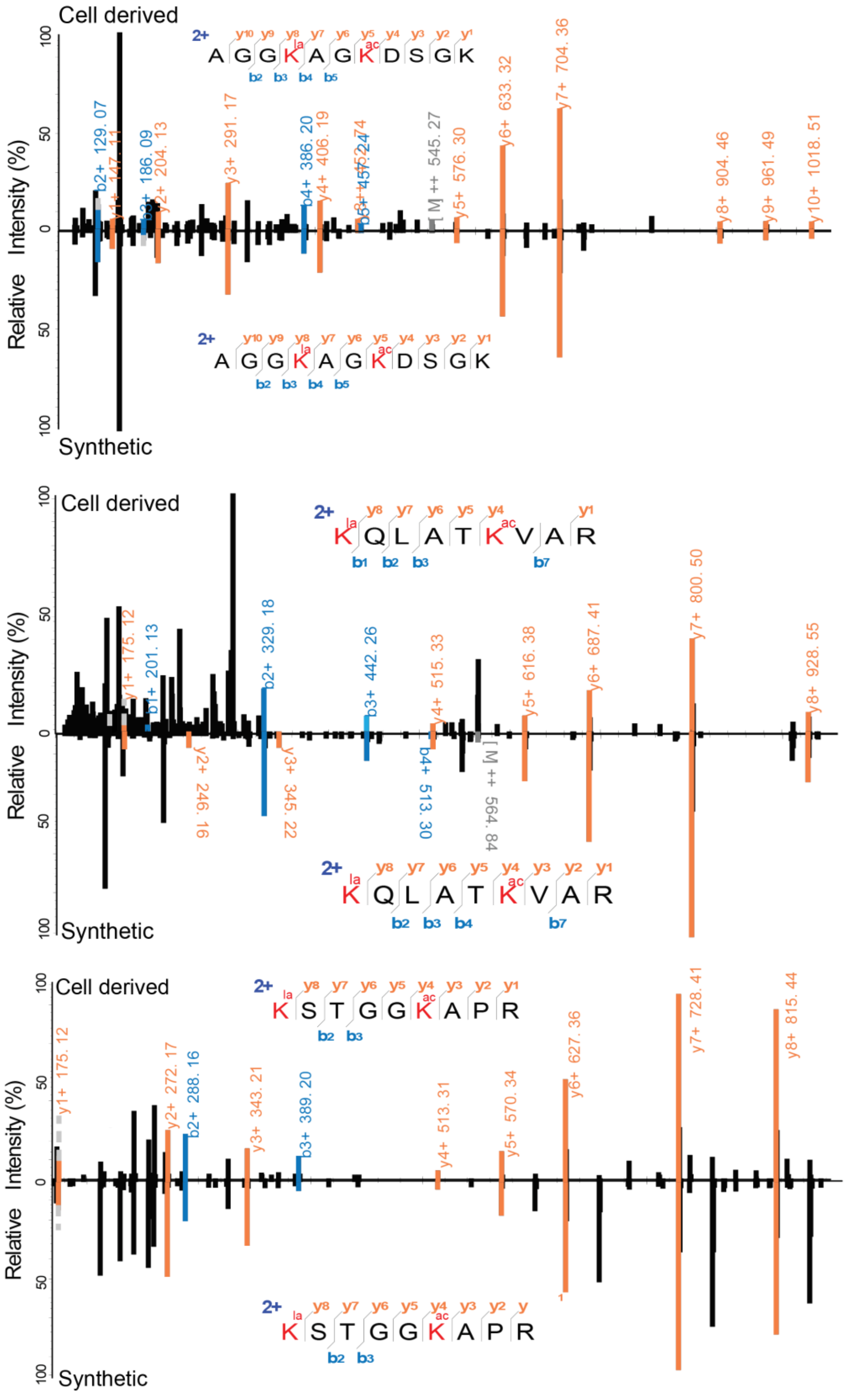

Figure S5, related to Figure 6. The MS/MS spectra of peptides identified with coexisting histone marks perfectly match those of the corresponding synthetic peptides.

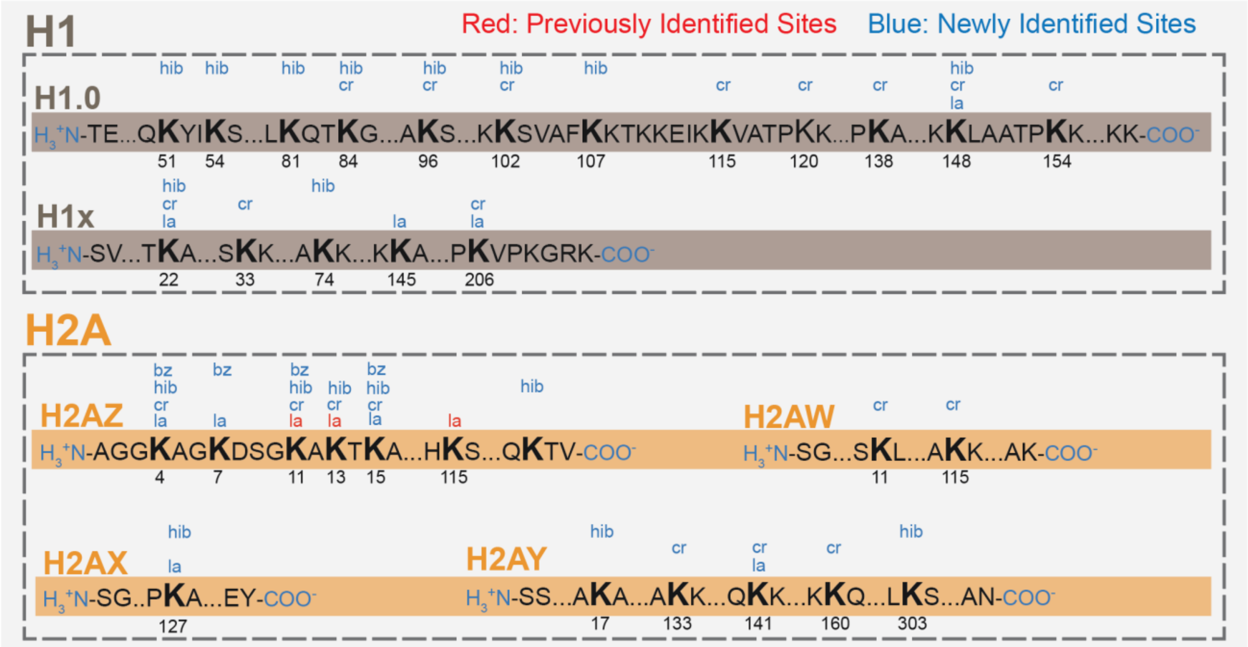

Figure S6, related to Figure 7. A comprehensive map of Kla, Kbz, Kcr, and Khib on noncanonical histones. A total of 54 histone marks were detected on noncanonical replication-independent histones, with 51 marks newly identified. bz: benzoylation; cr: crotonylation; hib: 2-hydroxyisobutyrylation; la: lactylation

II. Supplemental tables

Table S1 Xcorr and DeltaCN scores of PSMs in the four test datasets that were filtered out by false discovery rate screening.

Sequence	Dataset	MS/MS Scan No.	Xcorr	DeltaCN
GLGK*GGAK	A	1377	1.3556	0.3489
AVTK*AQK	A	986	1.8648	0.1504
AVTK*AQK	B	965	1.2928	0.0283
GGK*GLGK	B	1259	1.9045	0.1035
KAVTK*VQK	B	1083	1.8579	0.1468
GKGGK*GLGK	B	959	1.5376	0.0138
GLGK*GGAK	B	1417	1.3659	0.052
SAPAPK*K	C	1527	1.3665	0.1915
AVTK*VQK	C	1992	1.1913	0.2776
GLGK*GGAK	C	2244	1.2545	0.3282
AVTK*AQK	C	1510	1.7691	0.1674
GGK*GLGK	D	1686	1.7433	0.1908
SAPAPK*K	D	1280	1.4292	0.231
AVTK*VQK	D	1670	1.1248	0.1848
LAHYNK*R	D	3057	1.0395	0.1842

Table S2 The true positive and false positive rates using various cutoff values for dataset A, including FDR values from 1% to 100% and fragment coverage values from 100% to 0%.

FDR	True positive rate	False positive rate
**0.01**	0.42	0
**0.05**	0.58	0
**0.1**	0.58	0
**0.15**	0.58	0.01
**0.2**	0.58	0.01
**0.25**	0.67	0.01
**0.3**	0.75	0.02
**0.35**	0.75	0.04
**0.4**	0.75	0.05
**0.45**	0.75	0.05
**0.5**	0.83	0.07
**0.55**	0.92	0.13
**0.6**	0.92	0.18
**0.65**	1.00	0.23
**0.7**	1.00	0.34
**0.75**	1.00	0.77
**0.8**	1.00	0.9
**0.85**	1.00	0.92
**0.9**	1.00	0.95
**0.95**	1.00	0.98
**1**	1.00	1

FIC	True positive rate	False positive rate
**1**	0	0
**0.95**	0	0
**0.9**	0.08	0
**0.85**	0.08	0
**0.8**	0.17	0
**0.75**	0.33	0
**0.7**	0.42	0
**0.65**	0.75	0
**0.6**	0.83	0.01
**0.55**	0.92	0.01
**0.5**	1	0.02
**0.45**	1	0.02
**0.4**	1	0.05
**0.35**	1	0.08
**0.3**	1	0.12
**0.25**	1	0.19
**0.2**	1	0.28
**0.15**	1	0.45
**0.1**	1	0.68
**0.05**	1	0.92
**0**	1	1

Table S3 Chemically synthesized peptides. Five Kla peptides (green) undetected by the target-decoy strategy in Dataset A but identified by the 50% b/y ion coverage strategy were synthesized. MS/MS spectra of these peptides are shown in Figures 2E and S3. The remaining 18 peptides (black) were identified with moderate confidence for Kla and Kcr in the other datasets. MS/MS spectra of these peptides are shown in Figures 5D and Data S5.

Sequence	Histone	mass	m/z
AVTK(la)AQK	H2B	816.4705	409.2425
GGK(la)GLGK	H4	687.3915	344.703
GKGGK(la)GLGK	H4	872.508	437.2613
GLGK(la)GGAK	H4	758.4287	380.2216
KAVTK(la)VQK	H2B	972.5968	487.3057
GTGASGSFK(la)LNK	H1	1237.63	619.8223
K(la)VAGAATPK	H1	913.5233	457.7689
PSVPK(la)VPK	H1	922.5488	462.2817
K(la)LAATPK	H1	799.4804	400.7475
K(la)TSATVGPK	H2A	959.5288	480.7717
TSATVGPK(la)APSGGK	H2A	1328.694	665.3543
K(la)ESYSVYVYK	H2B	1336.655	669.3348
VTIMPK(la)DIQLAR	H3	1455.812	728.9133
K(cr)AGGTPR	H1	753.4133	377.7139
KPVGAAK(cr)K	H1	865.5385	433.7765
AAK(cr)SAAK	H1	713.4072	357.7109
AVKPK(cr)AAK	H1	879.5542	440.7844
K(cr)PAAAAGAK	H1	851.4865	426.7505
QTK(cr)GVGASGSFR	H1	1261.642	631.8283
VATPK(cr)K	H1	710.4327	356.2236
AGGSAALSPSK(cr)K	H1	1140.614	571.3143
PSVPK(cr)VPK	H1	918.5539	460.2842
IHPELLAK(cr)K	H2A	1115.67	558.8423

Table S4 Co-existing histone marks. Homologous sites of canonical histone subtypes were integrated, with their associated histone variant indicated.

H1	H2A	H2AZ	H2B	H3	H4
K33la, R34me1	K5la, K9ac	K4la, K7ac	K5ac, K11la	K9la, K14ac	K5la, K8ac
K45la, K51me1	K95la, R99me1	K7ac, K11la	K5ac, K11la, K16ac	K9ac, K14la	K5ac, K8la
K51la, R53me1	K5ac, K9hib	K7la, K11ac	K5la, K11ac, K16ac	K27la, K36me1	K8lac, K12ac
K62la, K63me1	K5ac, K9ce	K13ac, K15la	K11la, R12me1	K18la, K23ac	K5la, K12ac
K74la, R78me1	K5ce, K9ac	K4hib, K7ac	K11ac, K12ac, K15la	K9hib, K14ac	K12ac, K16la, R17me1
K147la, K148me1		K4ac, K7hib	K15la, K16ac	K18hib, K23ac	K77la, R78me1
K167la, K168me1		K7ac, K11hib	K15ac, K16la	K18ac, K23hib	R78me1, K79la
		K11ac, K13hib	K16ac,K20la	K9me1, K14cr	K91la, R92me1
		K13ac, K15hib	K16la, K20ac	K9cr, K14ac	K5hib, K8ac
		K4ce, K7ac	K16la, K20la	K9ac, K14cr	K8hib, K12ac
		K7ac, K11cr	K20la, K23ac	K18cr, K23ac	K12hib, K16ac
		K11ac, K13cr	K20ac, K23la	K18ac, K23cr	K8cr, K8ac
			K43la, K46me1	K18cr, K23ac	K5ac, K8cr
			K85la, R86me1	K18cr, K23cr	K8cr, K12ac
			K5ac, K11hib	K9bz, K14ac	K8ac, K12cr
			K20ac, K23hib	K18bz, K23ac	K12cr, K16cr
			K15ac, K15hib		K12cr, K16cr
			K16ac, K20hib		
			K11cr, K12ac		
			K16ac, K20cr		
			K5ac, K11cr		
			K16cr, K20ac		
			K15ac, K16cr		
			K20ac, K23cr		
			K16cr, K20cr		

III. Supplementary Data

Data S1. Kla peptide PSMs in Datasets A through E that were missed by the conventional database search strategy but identified by the strategy of 50% b/y ion coverage.

PSMs for Dataset A are shown in Fig. S3.

Dataset B

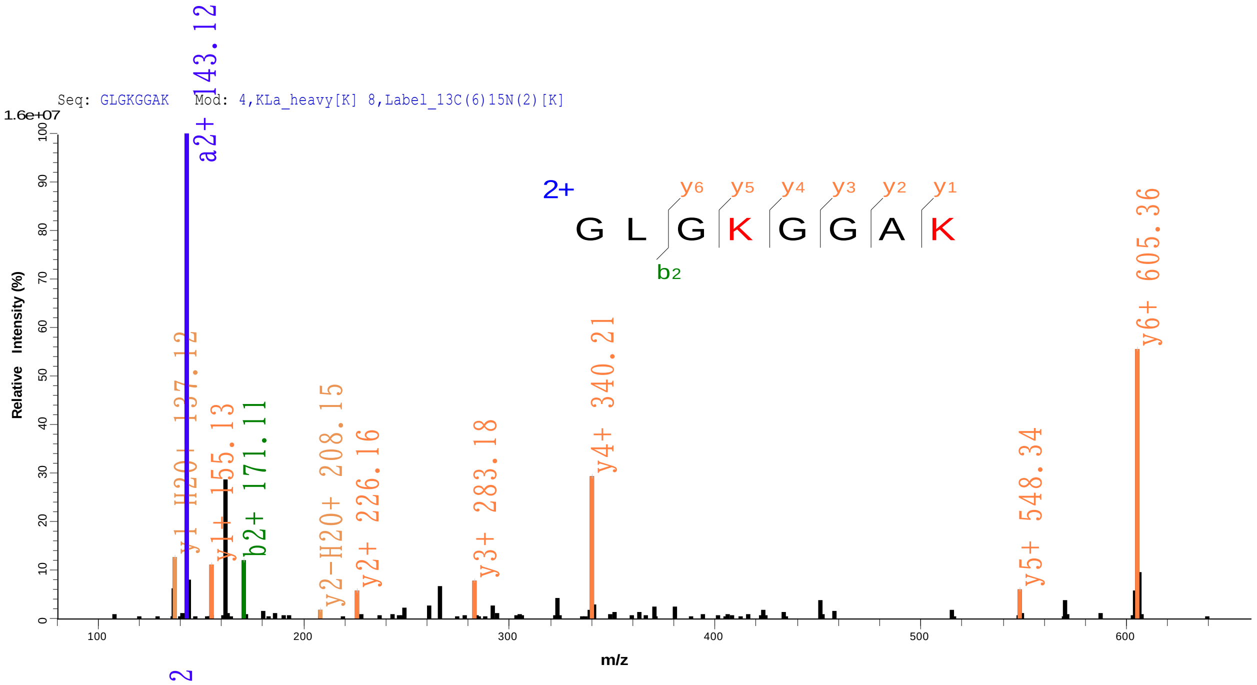

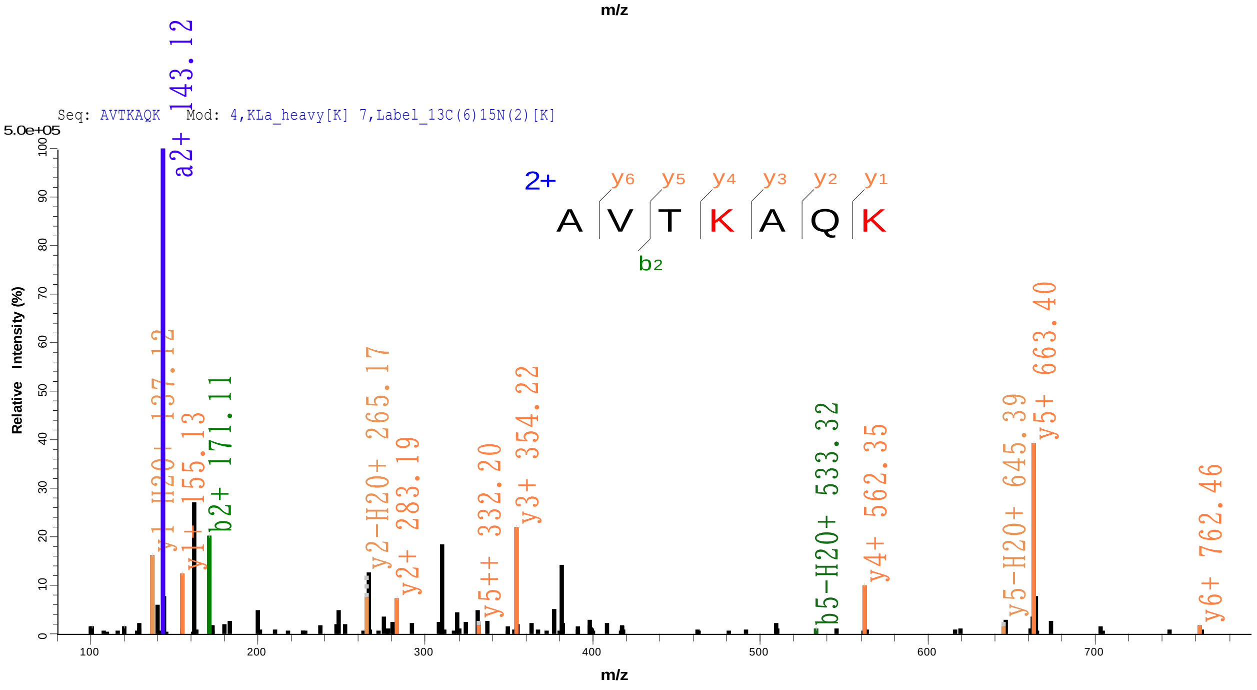

Dataset C

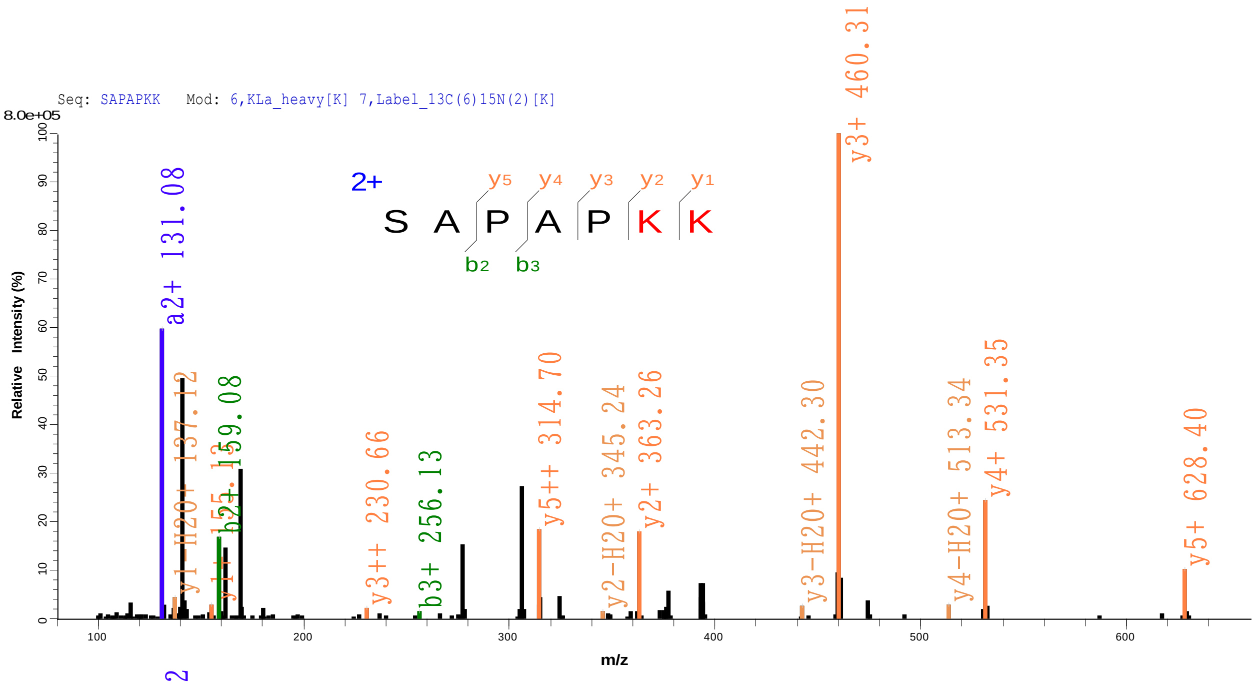
Seq: SAPAPKK Mod: 6,KLa_heavy[K] 7,Label_13C(6)15N(2)[K]
8.0e+05
Relative Intensity (%)
m/z
2+
S A P A P K K
y5 y4 y3 y2 y1
b2 b3
a2+ 131.08
y1-H2O+ 137.12
y1+ 155.13
b2+ 159.08
y3++ 230.66
b3+ 256.13
y5++ 314.70
y2-H2O+ 345.24
y2+ 363.26
y3-H2O+ 442.30
y3+ 460.31
y4-H2O+ 513.34
y4+ 531.35
y5+ 628.40

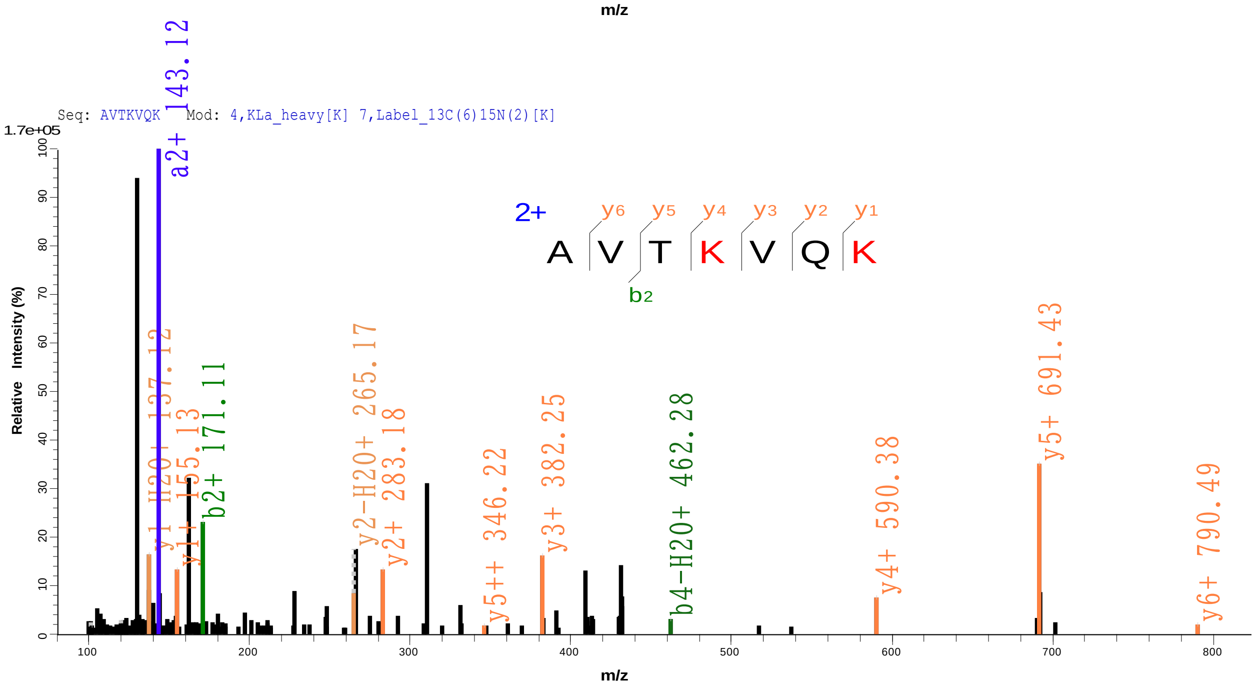
Seq: AVTKVQK Mod: 4,KLa_heavy[K] 7,Label_13C(6)15N(2)[K]
1.7e+05
Relative Intensity (%)
m/z
2+
A V T K V Q K
y6 y5 y4 y3 y2 y1
b2
a2+ 143.12
y1-H2O+ 137.12
y1+ 155.13
b2+ 171.11
y2-H2O+ 265.17
y2+ 283.18
y5++ 346.22
y3+ 382.25
b4-H2O+ 462.28
y4+ 590.38
y5+ 691.43
y6+ 790.49

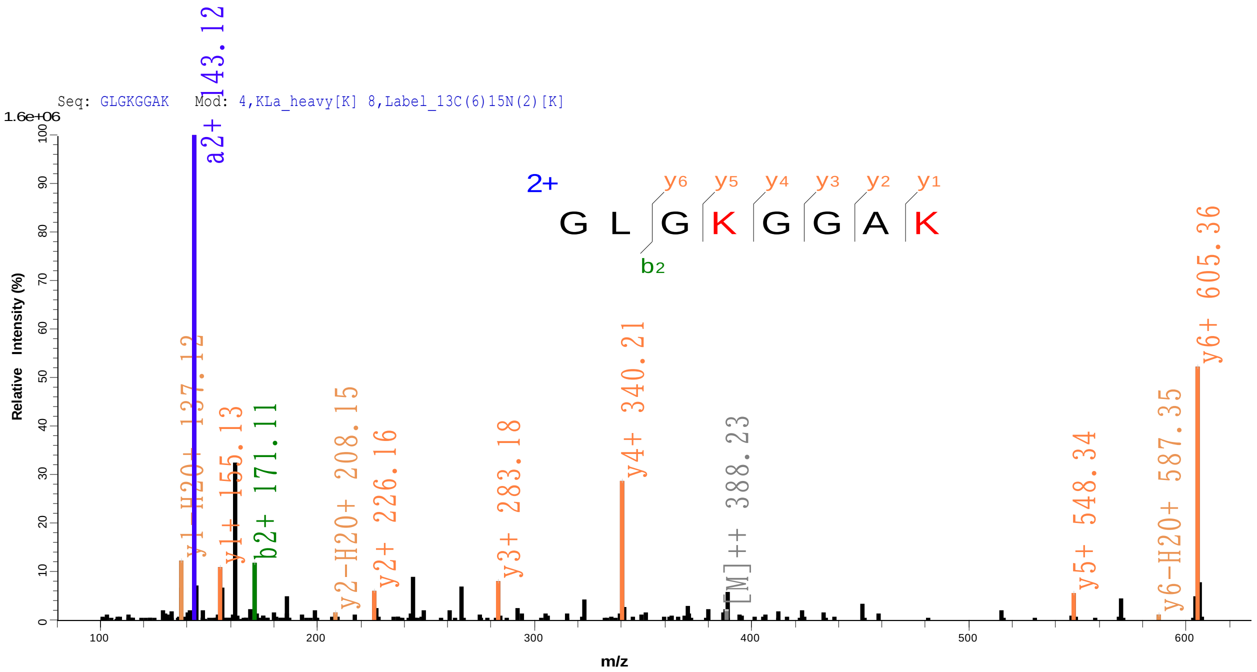
Seq: GLGKGGAK Mod: 4,KLa_heavy[K] 8,Label_13C(6)15N(2)[K]
1.6e+06
Relative Intensity (%)
m/z
2+
G L G K G G A K
y6 y5 y4 y3 y2 y1
b2
a2+ 143.12
y1-H2O+ 137.12
y1+ 155.13
b2+ 171.11
y2-H2O+ 208.15
y2+ 226.16
y3+ 283.18
y4+ 340.21
[M]++ 388.23
y5+ 548.34
y6-H2O+ 587.35
y6+ 605.36

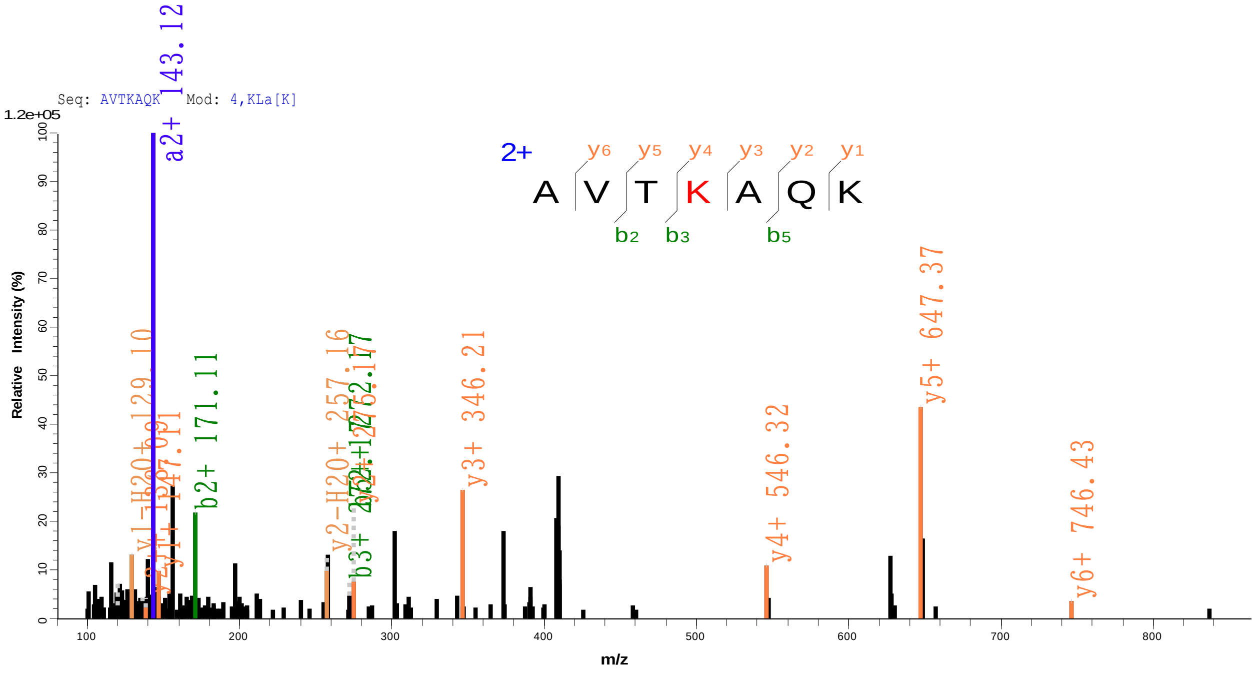
Seq: AVTKAQK Mod: 4,KLa[K]
1.2e+05
Relative Intensity (%)
m/z
2+
A V T K A Q K
y6 y5 y4 y3 y2 y1
b2 b3 b5
a2+ 143.12
y1-H2O+ 129.10
b2+ 171.11
y2-H2O+ 257.16
y3+ 346.21
y4+ 546.32
y5+ 647.37
y6+ 746.43

Dataset D

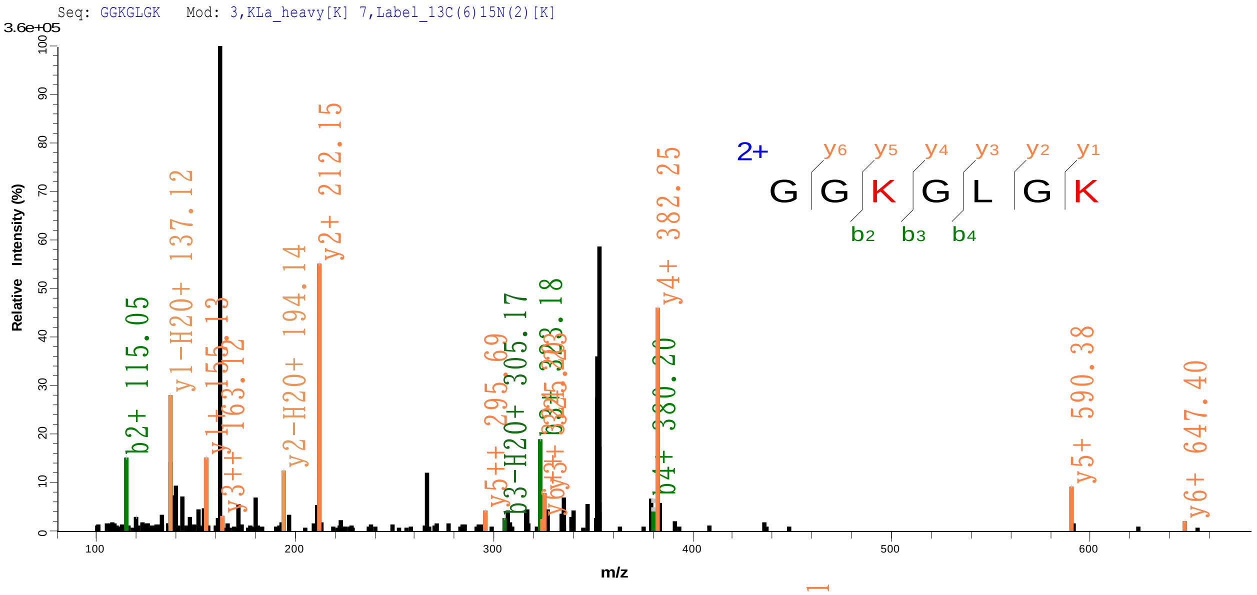
Seq: GGKGLGK Mod: 3,KLa_heavy[K] 7,Label_13C(6)15N(2)[K]
3.6e+05
Relative Intensity (%)
m/z
2+
G G K G L G K
y6 y5 y4 y3 y2 y1
b2 b3 b4
b2+ 115.05
y1-H2O+ 137.12
y2-H2O+ 194.14
y2+ 212.15
y4+ 382.25
b4+ 380.20
y5+ 590.38
y6+ 647.40

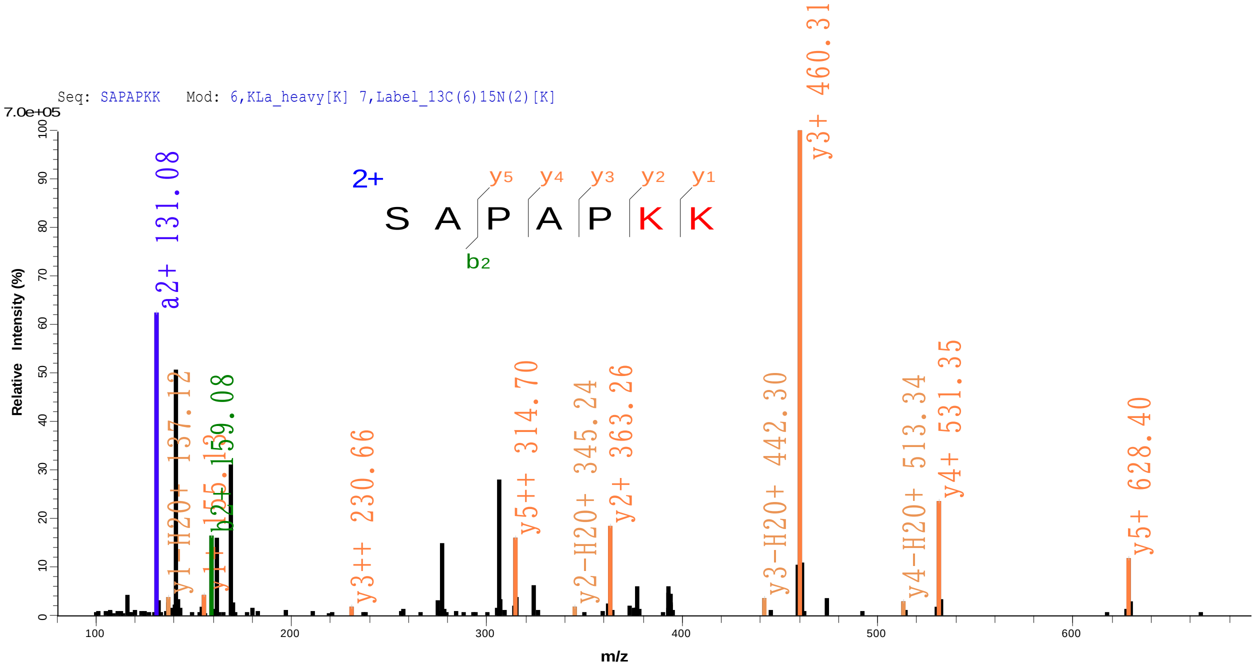
Seq: SAPAPKK Mod: 6,KLa_heavy[K] 7,Label_13C(6)15N(2)[K]
7.0e+05
Relative Intensity (%)
m/z
2+
S A P A P K K
y5 y4 y3 y2 y1
b2
a2+ 131.08
y3++ 230.66
y5++ 314.70
y2-H2O+ 345.24
y2+ 363.26
y3-H2O+ 442.30
y3+ 460.31
y4-H2O+ 513.34
y4+ 531.35
y5+ 628.40

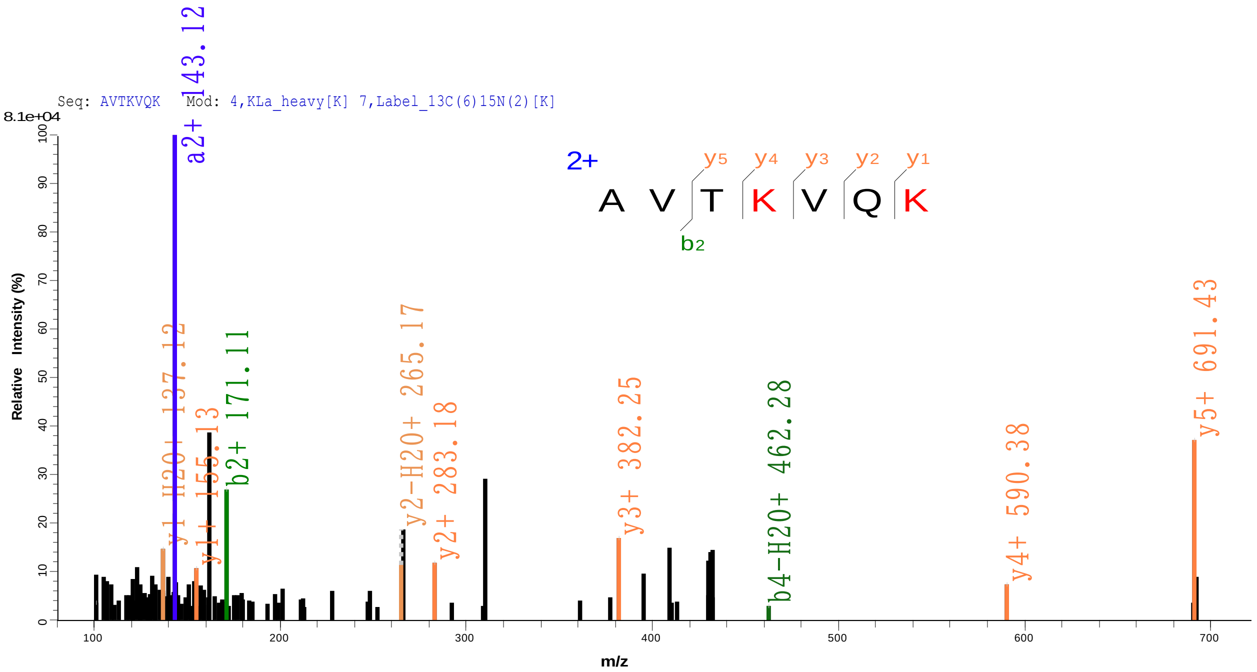
Seq: AVTKVQK Mod: 4,KLa_heavy[K] 7,Label_13C(6)15N(2)[K]
8.1e+04
Relative Intensity (%)
m/z
2+
A V T K V Q K
y5 y4 y3 y2 y1
b2
a2+ 143.12
b2+ 171.11
y1+ 155.13
y2-H2O+ 265.17
y2+ 283.18
y3+ 382.25
b4-H2O+ 462.28
y4+ 590.38
y5+ 691.43

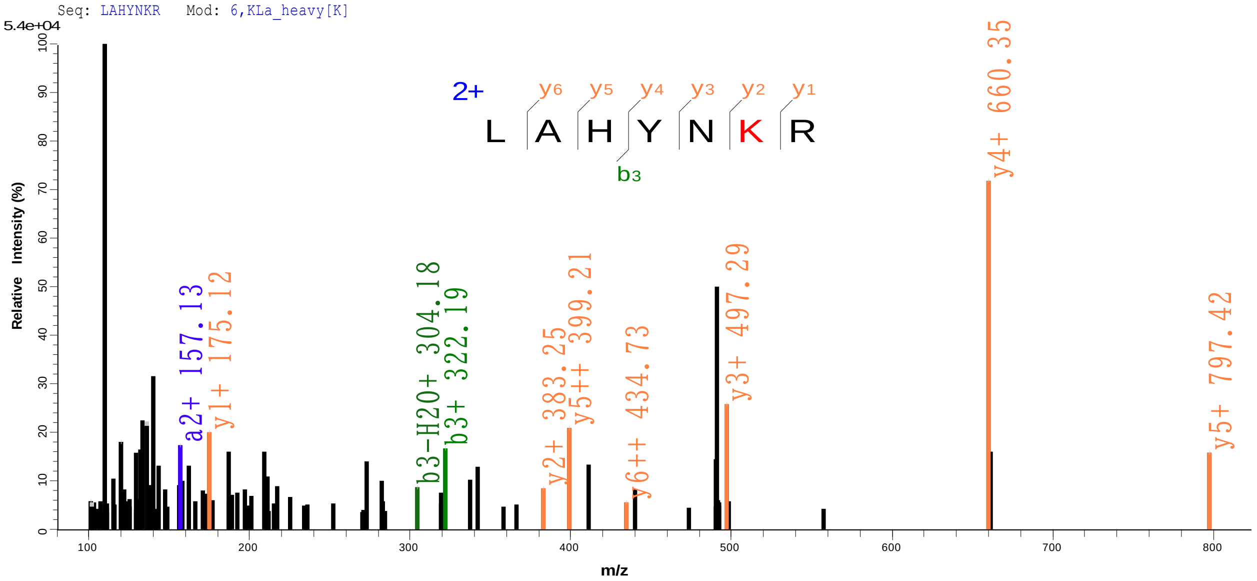
Seq: LAHYNKR Mod: 6,KLa_heavy[K]
5.4e+04
Relative Intensity (%)
m/z
2+
L A H Y N K R
y6 y5 y4 y3 y2 y1
b3
a2+ 157.13
y1+ 175.12
b3-H2O+ 304.18
b3+ 322.19
y2+ 383.25
y5++ 399.21
y6++ 434.73
y3+ 497.29
y4+ 660.35
y5+ 797.42

Dataset E

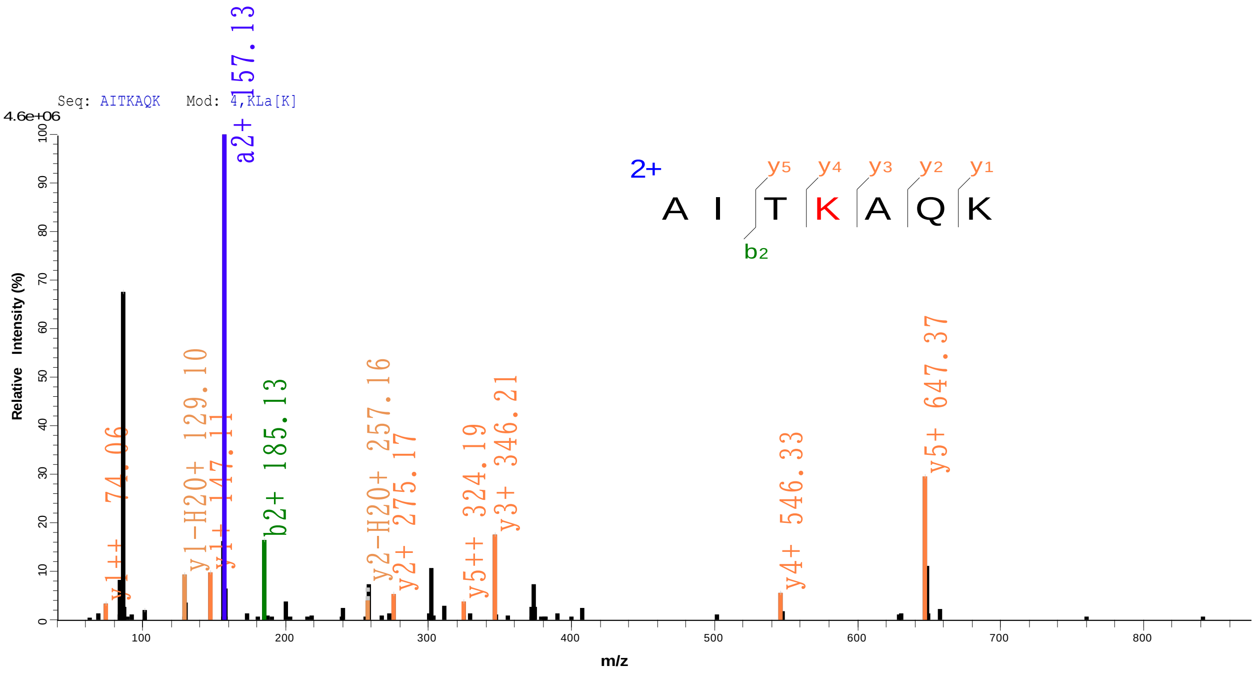

Seq: AITKAQK Mod: 4,KLa[K]
4.6e+06
Relative Intensity (%)
m/z
2+
A I T K A Q K
y5 y4 y3 y2 y1
b2
a2+ 157.13
b2+ 185.13
y1++ 74.06
y1-H2O+ 129.10
y1+ 147.11
y2-H2O+ 257.16
y2+ 275.17
y5++ 324.19
y3+ 346.21
y4+ 546.33
y5+ 647.37

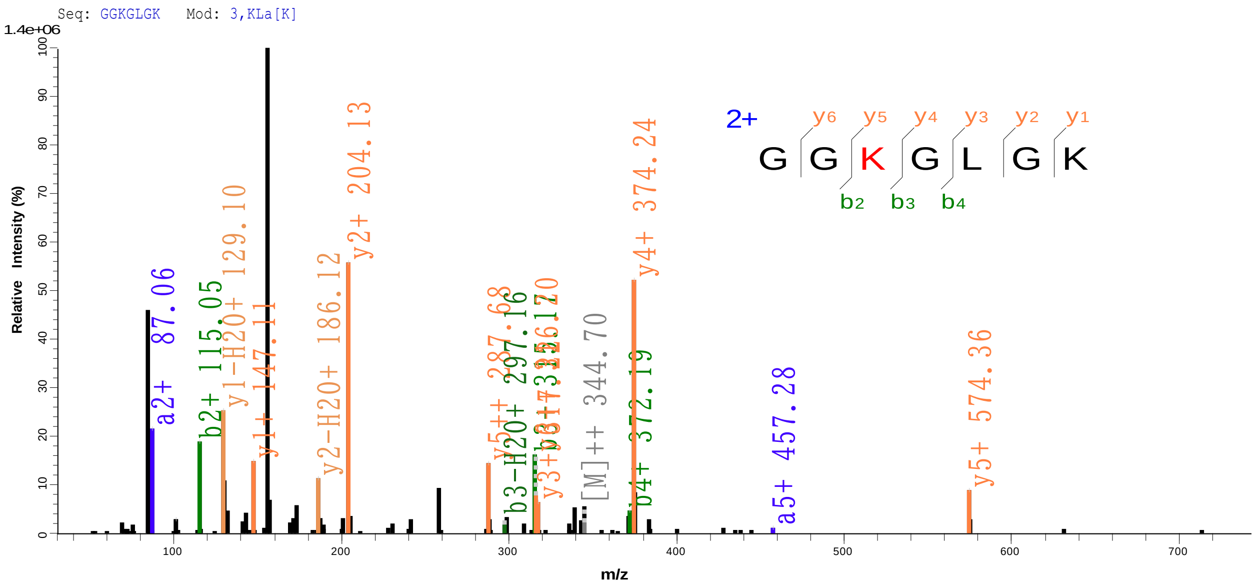

Seq: GGKGLGK Mod: 3,KLa[K]
1.4e+06
Relative Intensity (%)
m/z
2+
G G K G L G K
y6 y5 y4 y3 y2 y1
b2 b3 b4
a2+ 87.06
b2+ 115.05
y1-H2O+ 129.10
y1+ 147.11
y2-H2O+ 186.12
y2+ 204.13
y5++ 287.68
b3-H2O+ 297.16
[M]++ 344.70
b4+ 372.19
y4+ 374.24
a5+ 457.28
y5+ 574.36

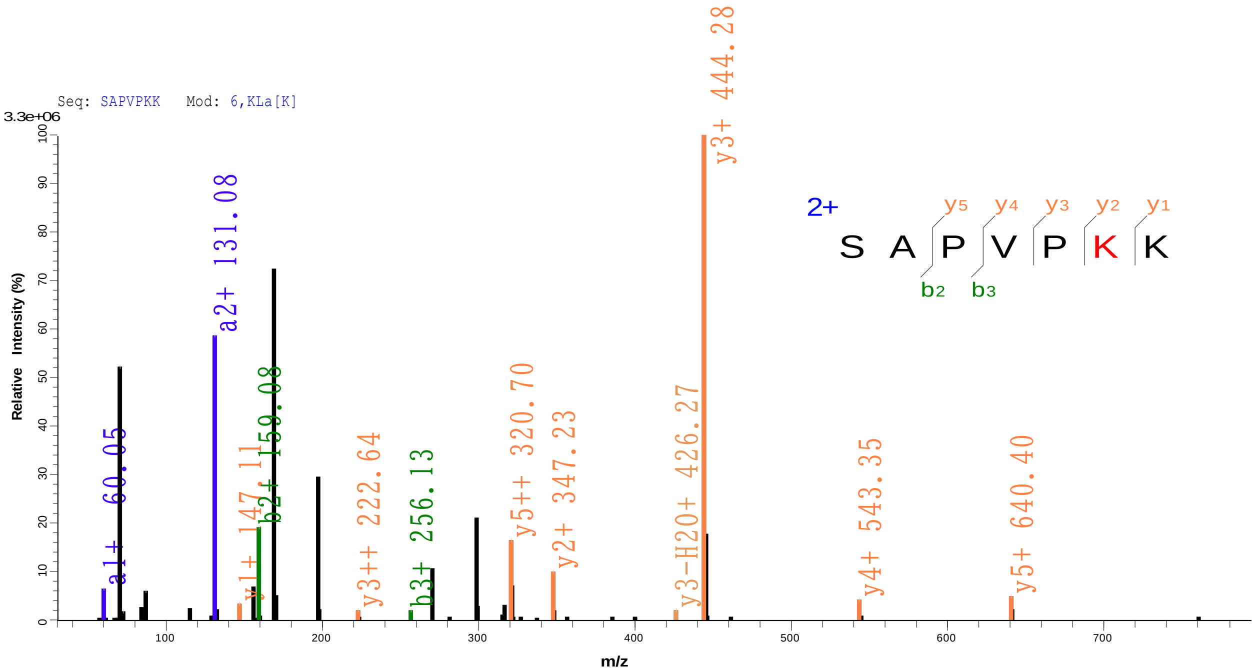
Seq: SAPVPKK Mod: 6,KLa[K]
3.3e+06
Relative Intensity (%)
m/z
2+
S A P V P K K
y5 y4 y3 y2 y1
b2 b3
a1+ 60.05
a2+ 131.08
y1+ 147.11
b2+ 159.08
y3++ 222.64
b3+ 256.13
y5++ 320.70
y2+ 347.23
y3-H2O+ 426.27
y3+ 444.28
y4+ 543.35
y5+ 640.40

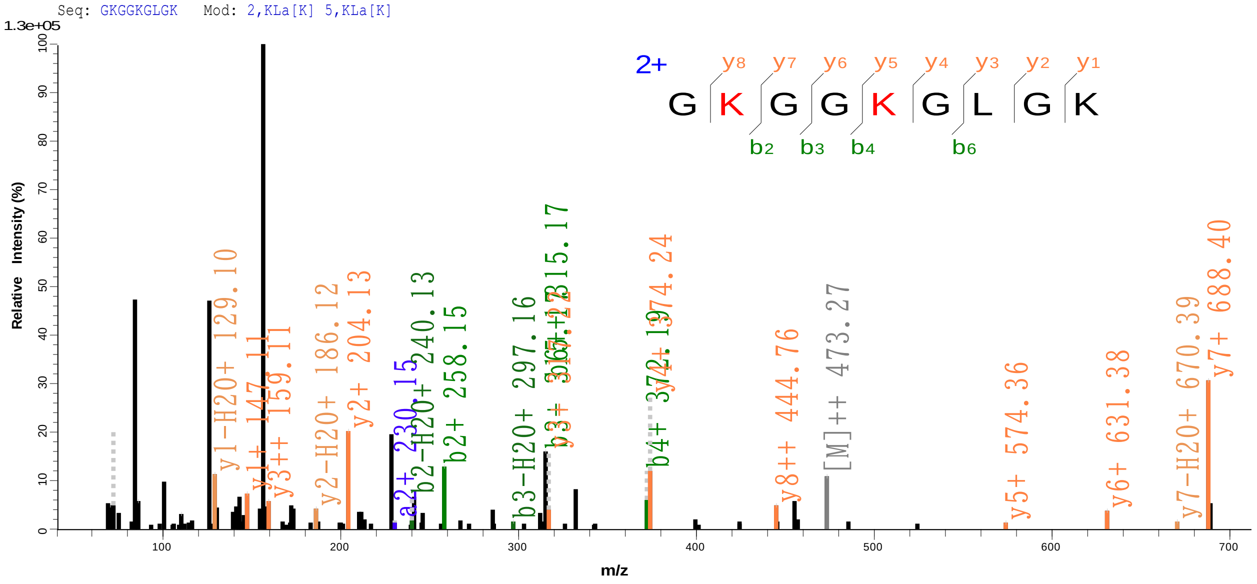
Seq: GKGGKGLGK Mod: 2,KLa[K] 5,KLa[K]
1.3e+05
Relative Intensity (%)
m/z
2+
G K G G K G L G K
y8 y7 y6 y5 y4 y3 y2 y1
b2 b3 b4 b6
y1-H2O+ 129.10
y1+ 147.11
y3++ 159.11
y2-H2O+ 186.12
y2+ 204.13
a2+ 230.15
b2-H2O+ 240.13
b2+ 258.15
b3-H2O+ 297.16
b4+ 372.19
y8++ 444.76
[M]++ 473.27
y5+ 574.36
y6+ 631.38
y7-H2O+ 670.39
y7+ 688.40

Seq: KSAPATGGVK Mod: 1,KLa[K]

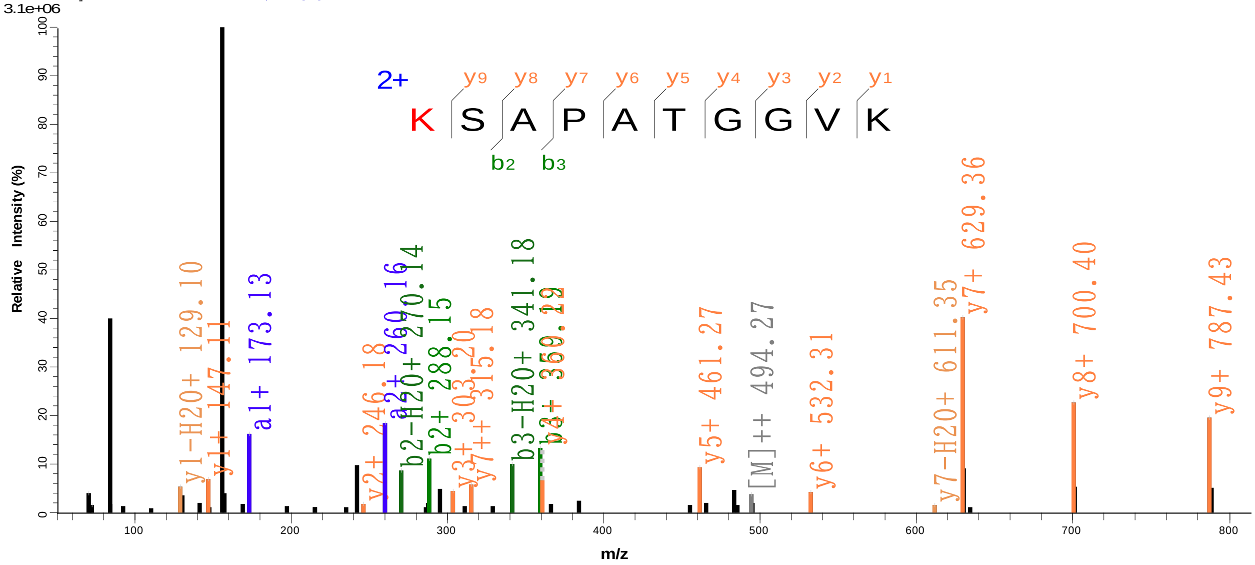

Data S2. Kla peptide PSMs in Datasets A and E that were additionally identified by adding Kac, Kme1, and Rme1 as variable modifications.

Dataset A

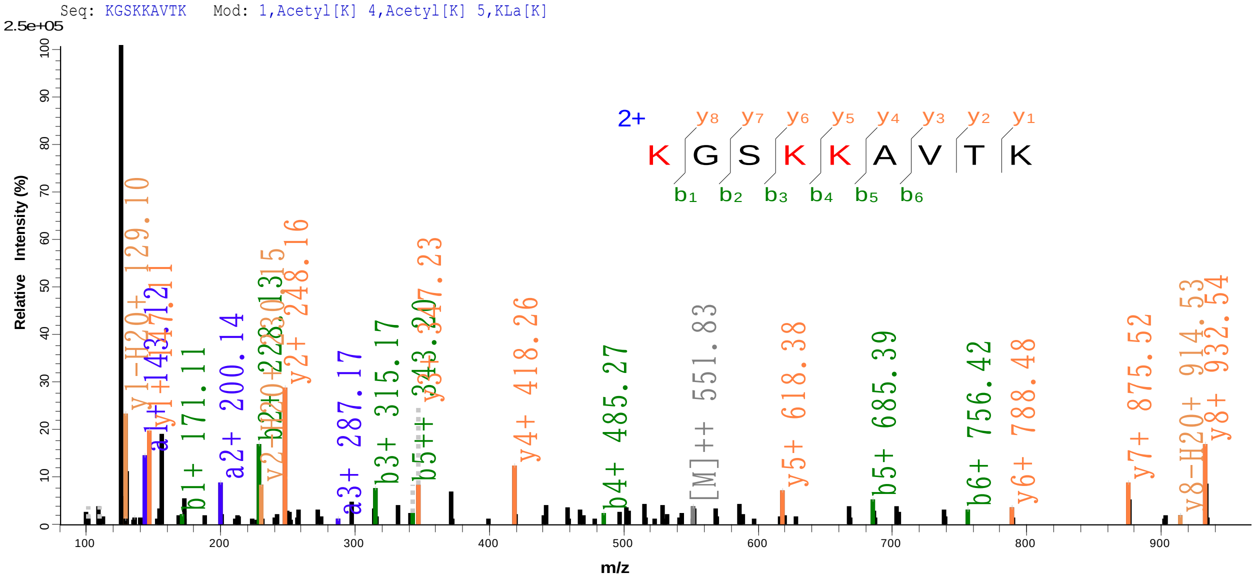

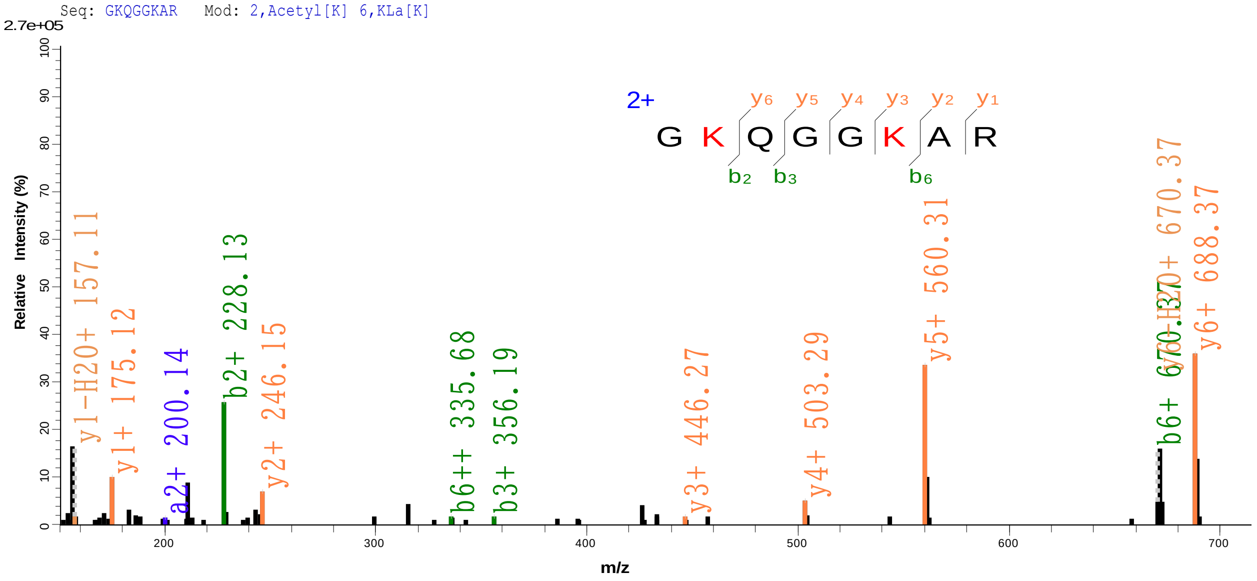

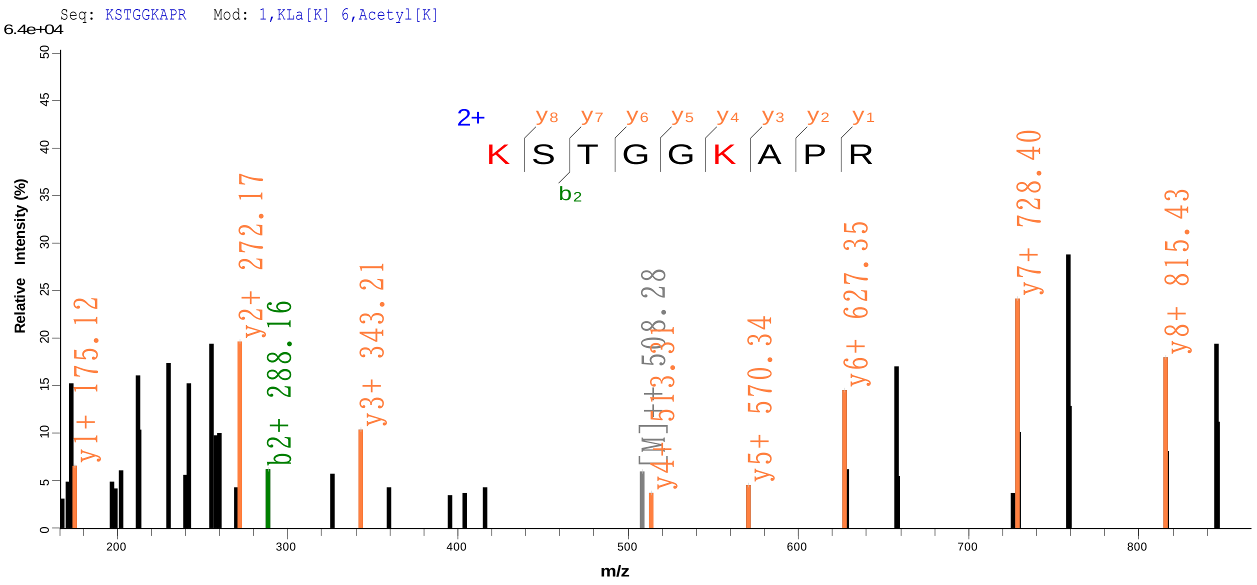
Seq: KSTGGKAPR Mod: 1,KLa[K] 6,Acetyl[K]
6.4e+04
Relative Intensity (%)
2+
K S T G G K A P R
y8 y7 y6 y5 y4 y3 y2 y1
b2
y1+ 175.12
y2+ 272.17
b2+ 288.16
y3+ 343.21
[M]++ 508.28
y4+ 513.31
y5+ 570.34
y6+ 627.35
y7+ 728.40
y8+ 815.43
m/z

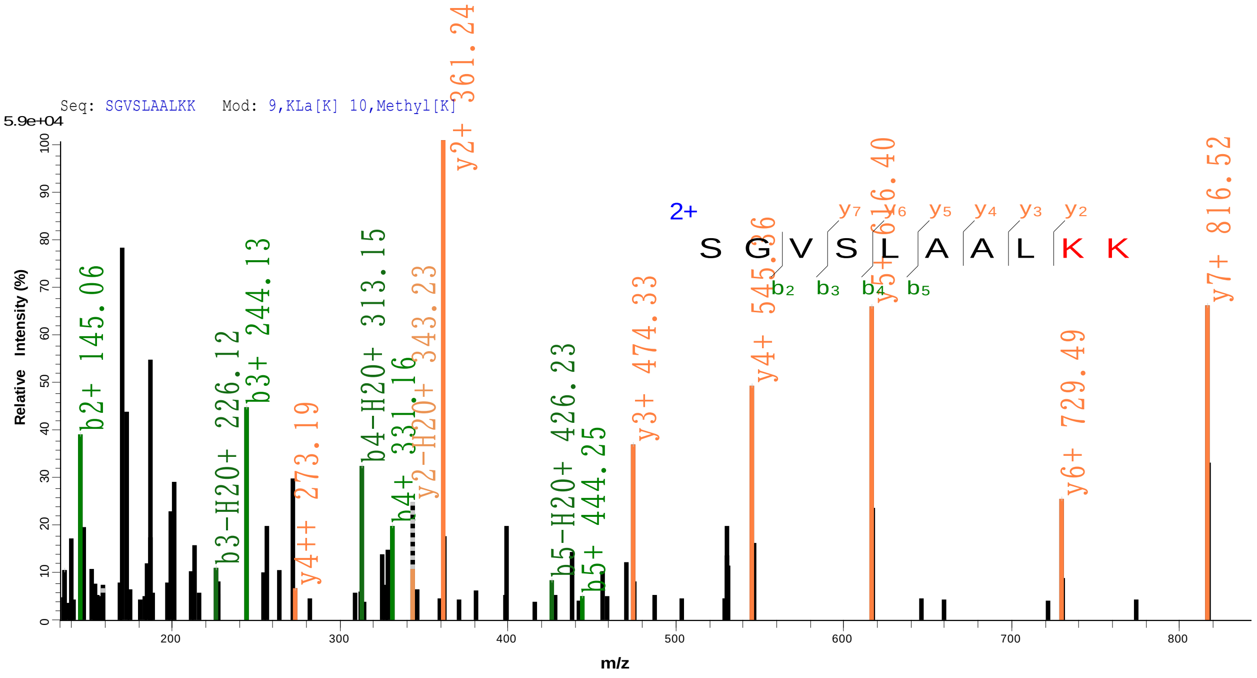
Seq: SGVSLAALKK Mod: 9,KLa[K] 10,Methyl[K]
5.9e+04
Relative Intensity (%)
2+
S G V S L A A L K K
y7 y6 y5 y4 y3 y2
b2 b3 b4 b5
b2+ 145.06
b3-H2O+ 226.12
b3+ 244.13
y4++ 273.19
b4-H2O+ 313.15
b4+ 331.16
y2-H2O+ 343.23
y2+ 361.24
b5-H2O+ 426.23
b5+ 444.25
y3+ 474.33
y4+ 545.36
y5+ 616.40
y6+ 729.49
y7+ 816.52
m/z

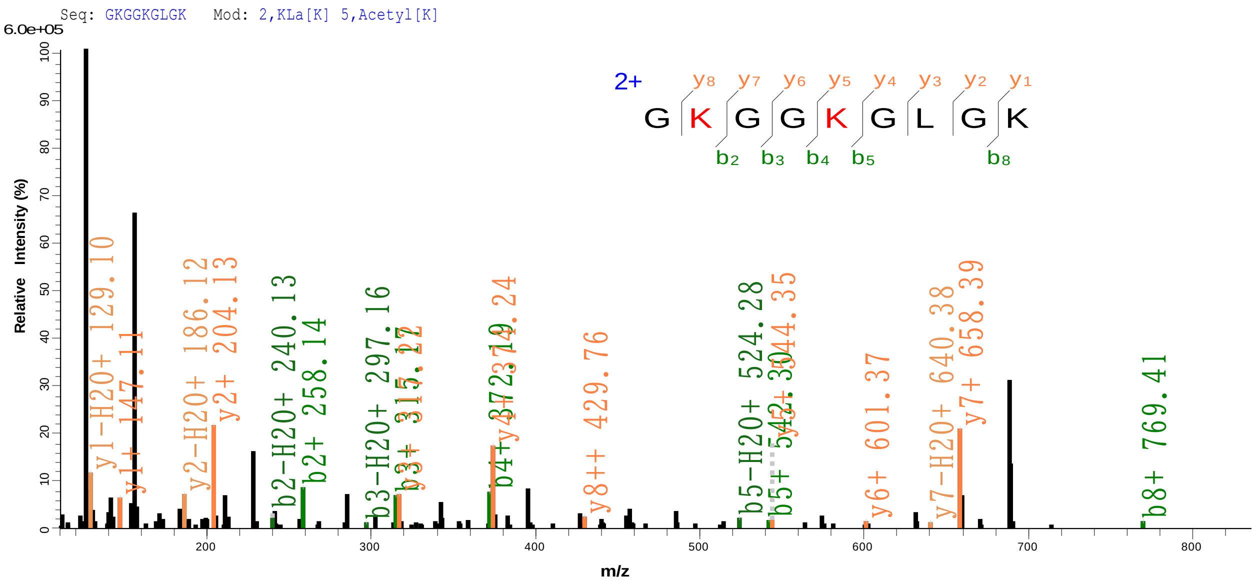
Seq: GKGGKGLGK Mod: 2,KLa[K] 5,Acetyl[K]
6.0e+05
Relative Intensity (%)
m/z
2+
G K G G K G L G K
y8 y7 y6 y5 y4 y3 y2 y1
b2 b3 b4 b5 b8
y1-H2O+ 129.10
y1+ 147.11
y2-H2O+ 186.12
y2+ 204.13
b2-H2O+ 240.13
b2+ 258.14
b3-H2O+ 297.16
b3+ 315.17
y8++ 429.76
b5-H2O+ 524.28
y6+ 601.37
y7-H2O+ 640.38
y7+ 658.39
b8+ 769.41

Dataset E

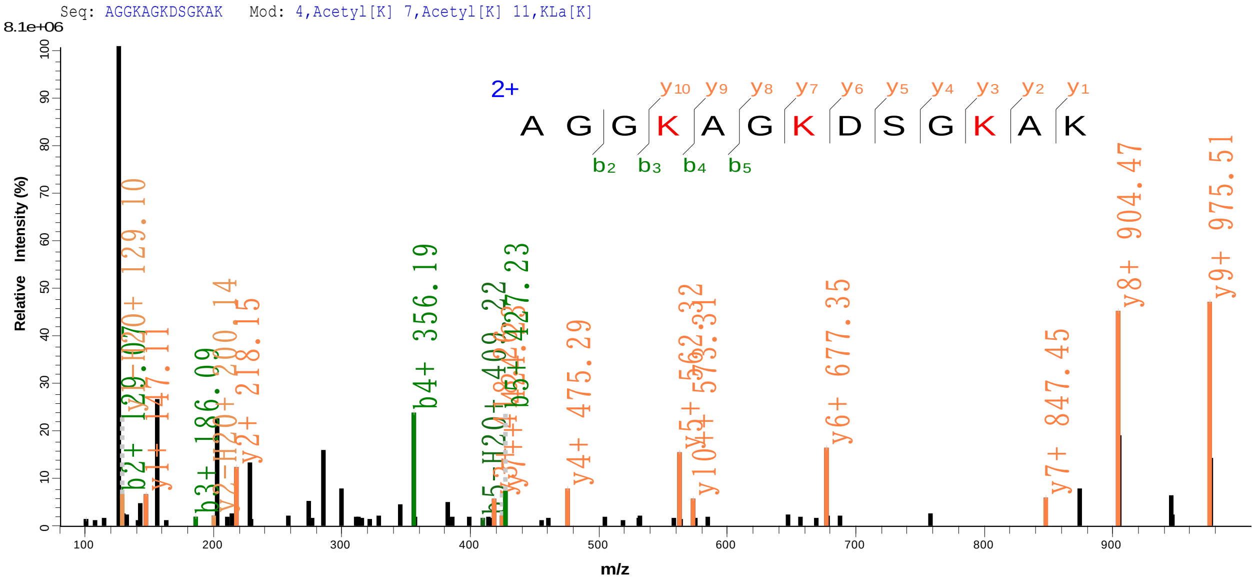
Seq: AGGKAGKDSGKAK Mod: 4,Acetyl[K] 7,Acetyl[K] 11,KLa[K]
8.1e+06
Relative Intensity (%)
m/z
2+
A G G K A G K D S G K A K
y10 y9 y8 y7 y6 y5 y4 y3 y2 y1
b2 b3 b4 b5
y1+ 147.11
b3+ 186.09
y2+ 218.15
b4+ 356.19
y4+ 475.29
y6+ 677.35
y7+ 847.45
y8+ 904.47
y9+ 975.51

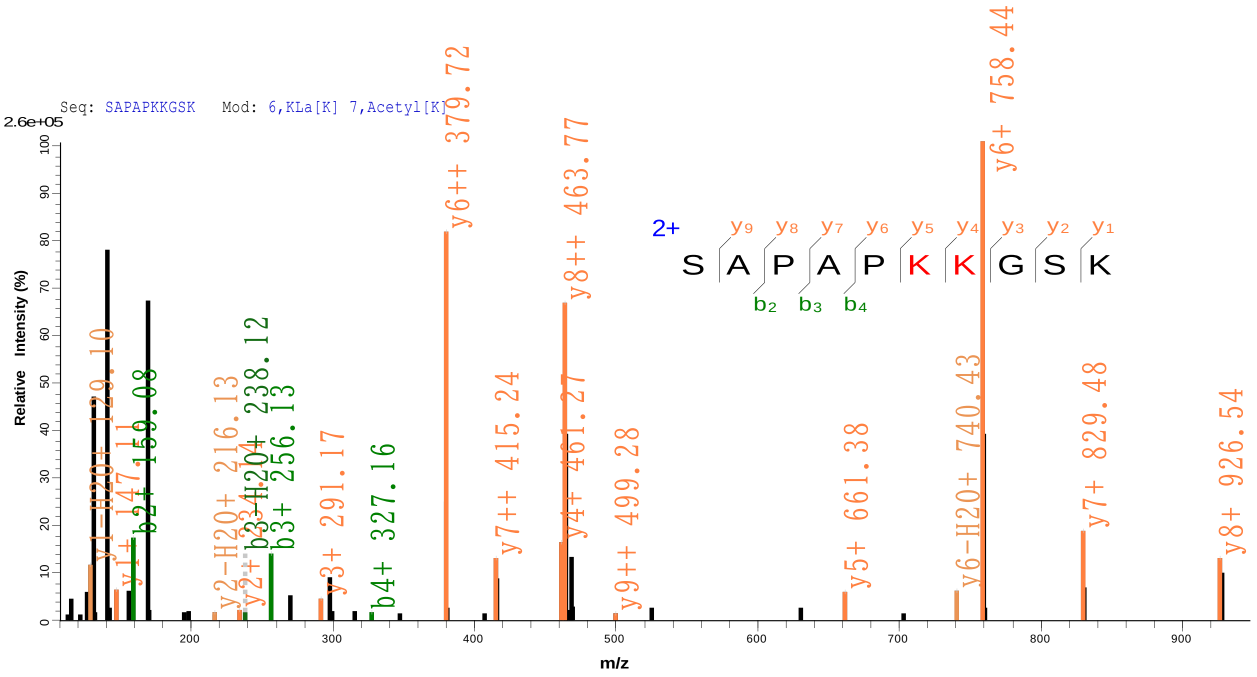
Seq: SAPAPKKGSK Mod: 6,KLa[K] 7,Acetyl[K]
2.6e+05
Relative Intensity (%)
m/z
2+
S A P A P K K G S K
y6++ 379.72
y6+ 758.44
y8++ 463.77
y7++ 415.24
y3+ 291.17
b4+ 327.16
y9++ 499.28
y5+ 661.38
y6-H2O+ 740.43
y7+ 829.48
y8+ 926.54
b3-H2O+ 238.12
b3+ 256.13
y2-H2O+ 216.13
b2+ 159.08

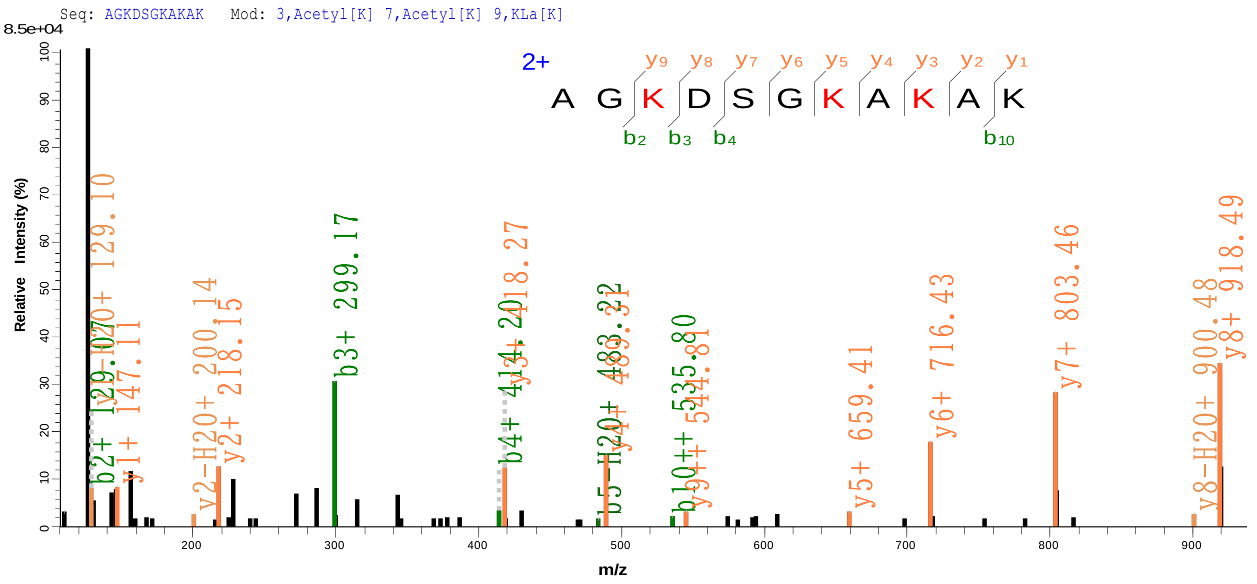
Seq: AGKDSGKAKAK Mod: 3,Acetyl[K] 7,Acetyl[K] 9,KLa[K]
8.5e+04
Relative Intensity (%)
m/z
2+
A G K D S G K A K A K
b3+ 299.17
y2-H2O+ 200.14
y2+ 218.15
y1+ 147.11
y5+ 659.41
y6+ 716.43
y7+ 803.46
y8-H2O+ 900.48
y8+ 918.49
b10++ 535.80

Seq: AKTKAVSR   Mod: 2,Acetyl[K] 4,KLa[K]

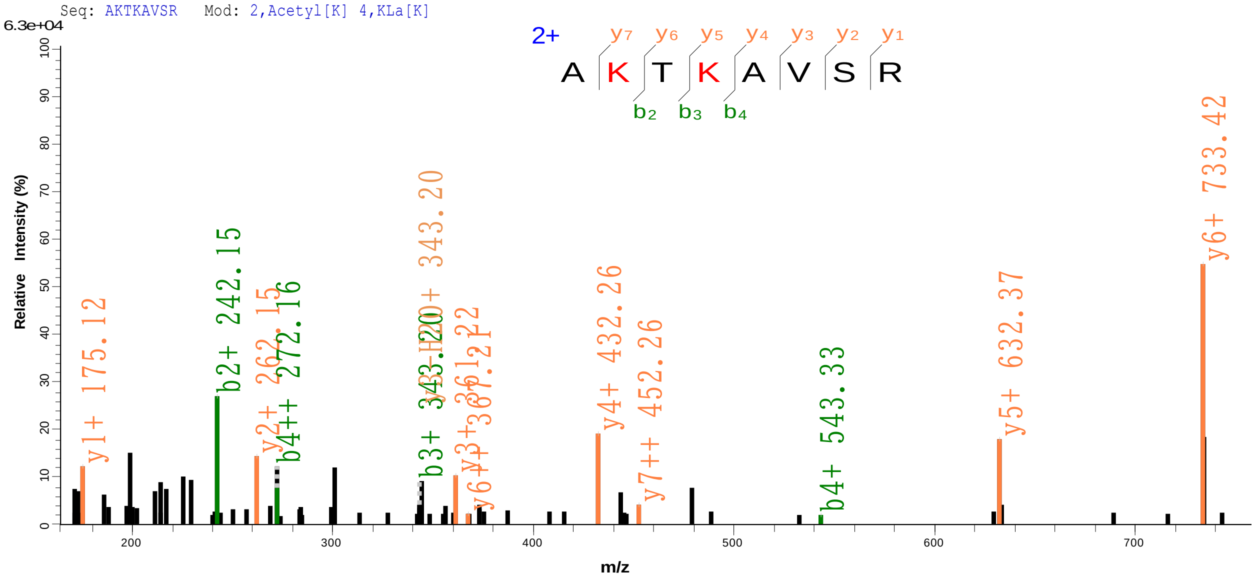

Seq: ATGPPVSELITKAVAASK   Mod: 12,KLa[K] 18,Methyl[K]

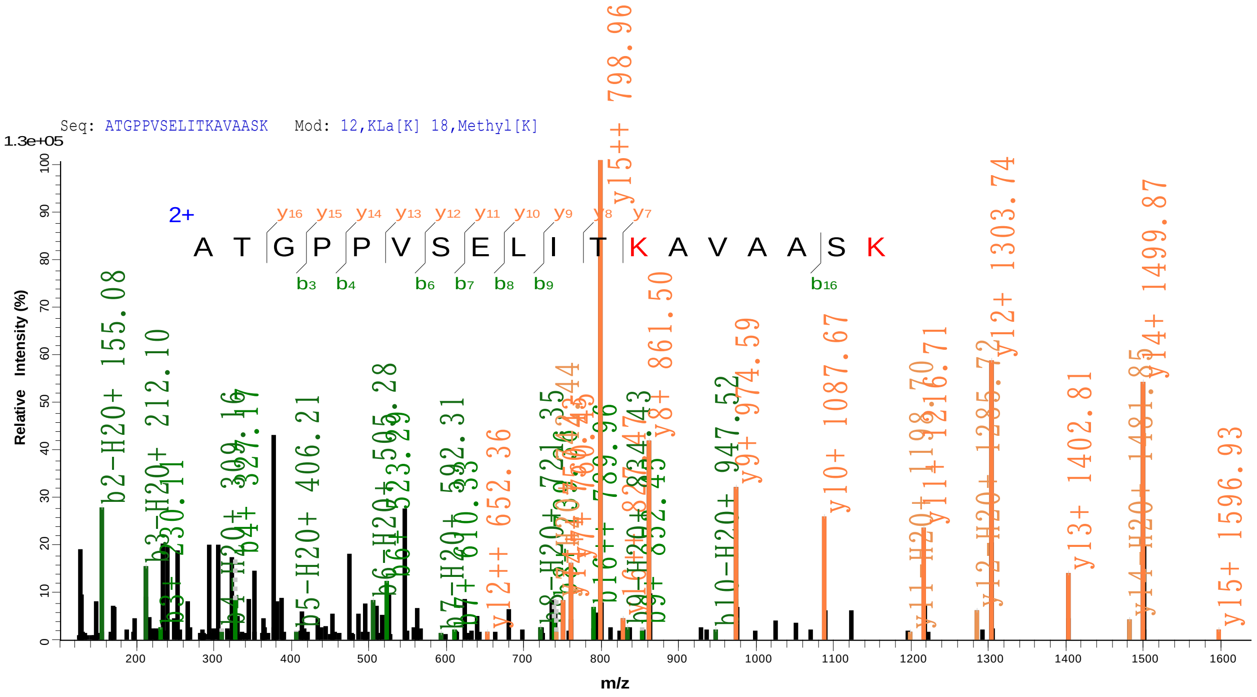

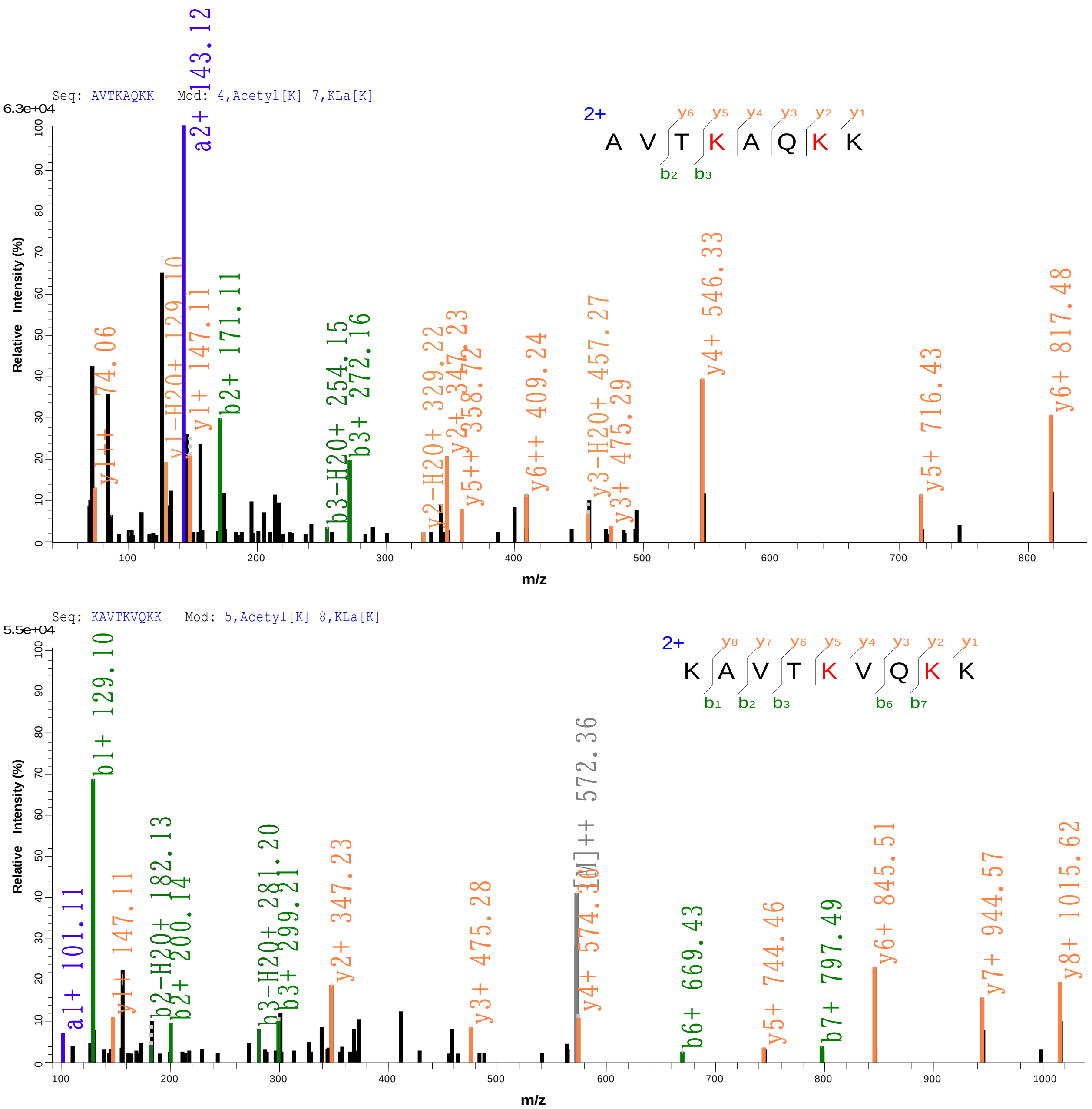

Seq: AVTKAQKK Mod: 4,Acetyl[K] 7,KLa[K]
6.3e+04
2+
A V T K A Q K K
y6 y5 y4 y3 y2 y1
b2 b3
Relative Intensity (%)
m/z
a2+ 143.12
y1++ 74.06
y1-H2O+ 129.10
y1+ 147.11
b2+ 171.11
b3-H2O+ 254.15
b3+ 272.16
y2-H2O+ 329.22
y2+ 347.23
y5++ 358.72
y6++ 409.24
y3-H2O+ 457.27
y3+ 475.29
y4+ 546.33
y5+ 716.43
y6+ 817.48
Seq: KAVTKVQKK Mod: 5,Acetyl[K] 8,KLa[K]
5.5e+04
2+
K A V T K V Q K K
y8 y7 y6 y5 y4 y3 y2 y1
b1 b2 b3 b6 b7
a1+ 101.11
b1+ 129.10
y1+ 147.11
b2-H2O+ 182.13
b2+ 200.14
b3-H2O+ 281.20
b3+ 299.21
y2+ 347.23
y3+ 475.28
[M]++ 572.36
y4+ 574.36
b6+ 669.43
y5+ 744.46
b7+ 797.49
y6+ 845.51
y7+ 944.57
y8+ 1015.62

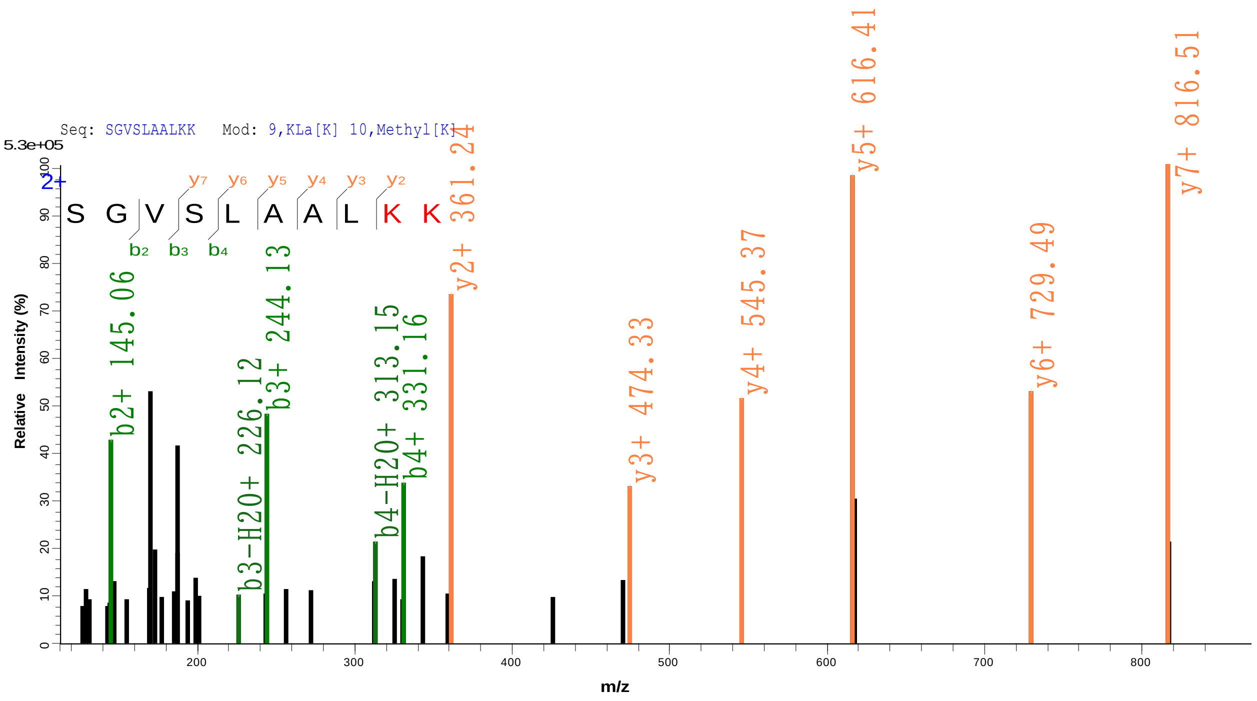
Seq: SGVSLAALKK Mod: 9,KLa[K] 10,Methyl[K]
5.3e+05
2+
S G V S L A A L K K
y7 y6 y5 y4 y3 y2
b2 b3 b4
Relative Intensity (%)
m/z
b2+ 145.06
b3-H2O+ 226.12
b3+ 244.13
b4-H2O+ 313.15
b4+ 331.16
y2+ 361.24
y3+ 474.33
y4+ 545.37
y5+ 616.41
y6+ 729.49
y7+ 816.51

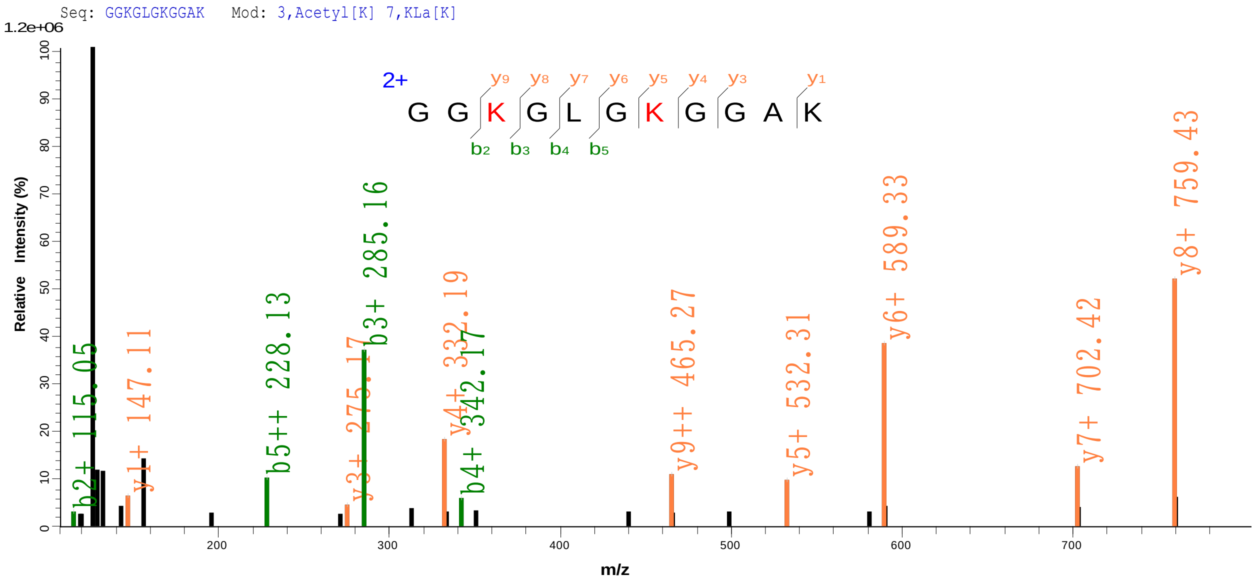
Seq: GGKGLGKGGAK Mod: 3,Acetyl[K] 7,KLa[K]
1.2e+06
2+
G G K G L G K G G A K
y9 y8 y7 y6 y5 y4 y3 y1
b2 b3 b4 b5
Relative Intensity (%)
m/z
b2+ 115.05
y1+ 147.11
b5++ 228.13
y3+ 275.17
b3+ 285.16
y4+ 332.19
b4+ 342.17
y9++ 465.27
y5+ 532.31
y6+ 589.33
y7+ 702.42
y8+ 759.43

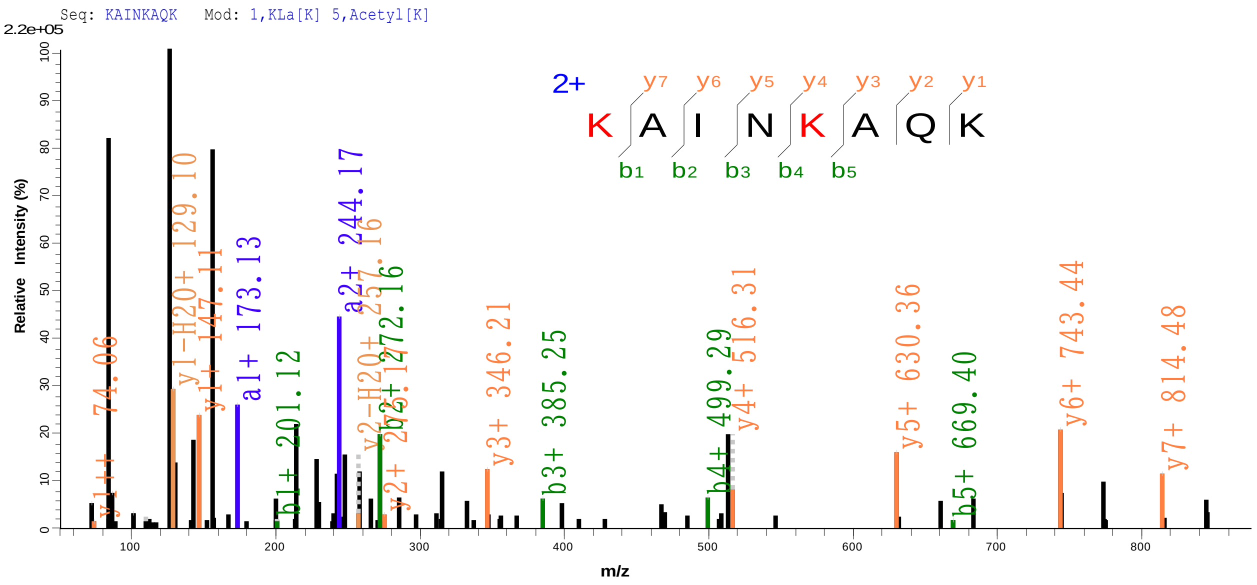
Seq: KAINKAQK Mod: 1,KLa[K] 5,Acetyl[K]
2.2e+05
Relative Intensity (%)
m/z
2+
K A I N K A Q K
y7 y6 y5 y4 y3 y2 y1
b1 b2 b3 b4 b5
y1-H2O+ 129.10
y1+ 147.11
a1+ 173.13
b1+ 201.12
a2+ 244.17
y3+ 346.21
b3+ 385.25
b4+ 499.29
y4+ 516.31
y5+ 630.36
b5+ 669.40
y6+ 743.44
y7+ 814.48

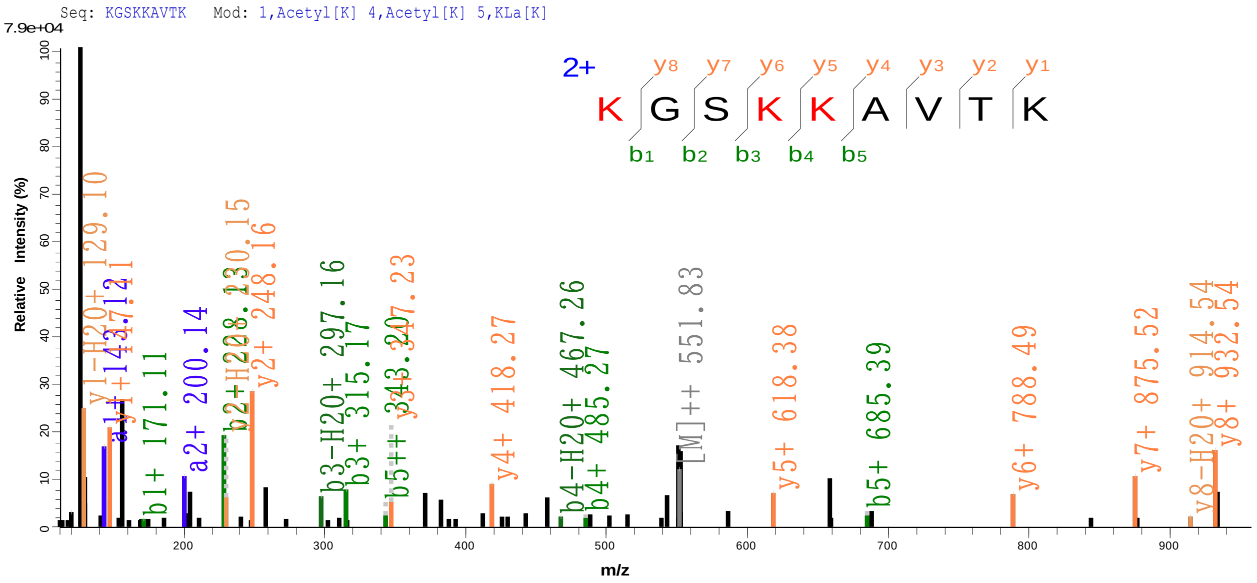
Seq: KGSKKAVTK Mod: 1,Acetyl[K] 4,Acetyl[K] 5,KLa[K]
7.9e+04
Relative Intensity (%)
m/z
2+
K G S K K A V T K
y8 y7 y6 y5 y4 y3 y2 y1
b1 b2 b3 b4 b5
y1-H2O+ 129.10
b1+ 171.11
a2+ 200.14
y2+ 248.16
b3-H2O+ 297.16
b3+ 315.17
y4+ 418.27
b4-H2O+ 467.26
b4+ 485.27
[M]++ 551.83
y5+ 618.38
b5+ 685.39
y6+ 788.49
y7+ 875.52
y8-H2O+ 914.54
y8+ 932.54

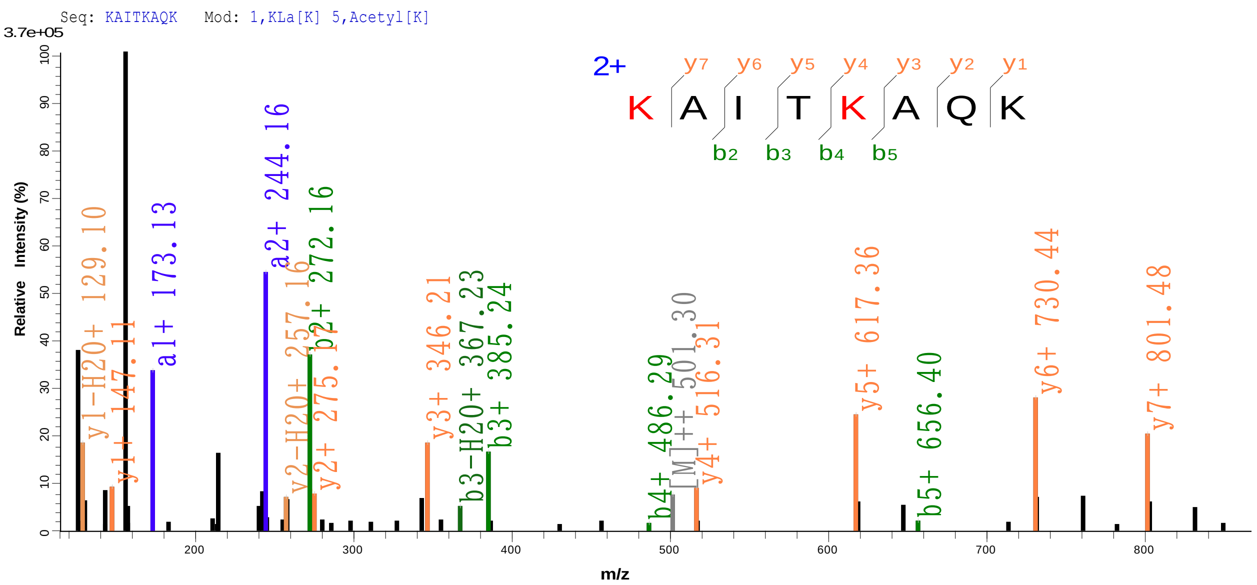

Seq: KAITKAQK Mod: 1,KLa[K] 5,Acetyl[K]
3.7e+05
Relative Intensity (%)
m/z
2+
K A I T K A Q K
y7 y6 y5 y4 y3 y2 y1
b2 b3 b4 b5
y1-H2O+ 129.10
y1+ 147.11
a1+ 173.13
a2+ 244.16
y2-H2O+ 257.16
b2+ 272.16
y2+ 275.17
y3+ 346.21
b3-H2O+ 367.23
b3+ 385.24
b4+ 486.29
[M]++ 501.30
y4+ 516.31
y5+ 617.36
b5+ 656.40
y6+ 730.44
y7+ 801.48

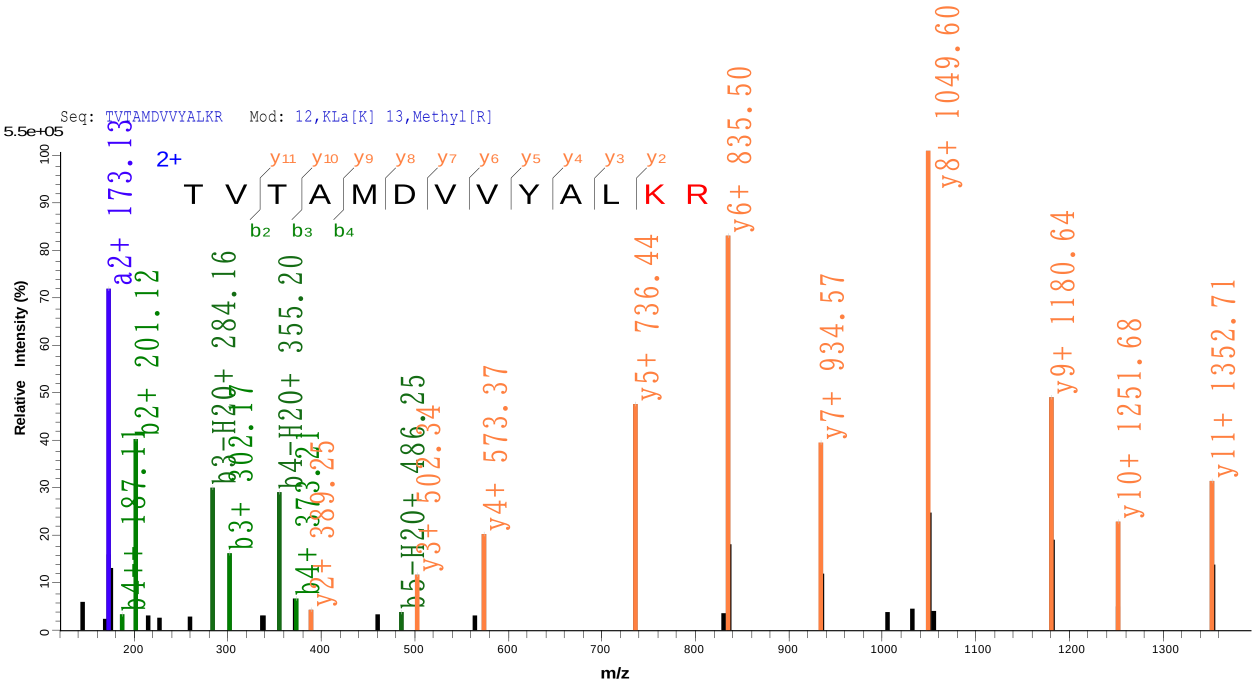

Seq: TVTAMDVVYALKR Mod: 12,KLa[K] 13,Methyl[R]
5.5e+05
Relative Intensity (%)
m/z
2+
T V T A M D V V Y A L K R
y11 y10 y9 y8 y7 y6 y5 y4 y3 y2
b2 b3 b4
a2+ 173.13
b4++ 187.11
b2+ 201.12
b3-H2O+ 284.16
b3+ 302.17
b4-H2O+ 355.20
b4+ 373.21
y2+ 389.25
b5-H2O+ 486.25
y3+ 502.34
y4+ 573.37
y5+ 736.44
y6+ 835.50
y7+ 934.57
y8+ 1049.60
y9+ 1180.64
y10+ 1251.68
y11+ 1352.71

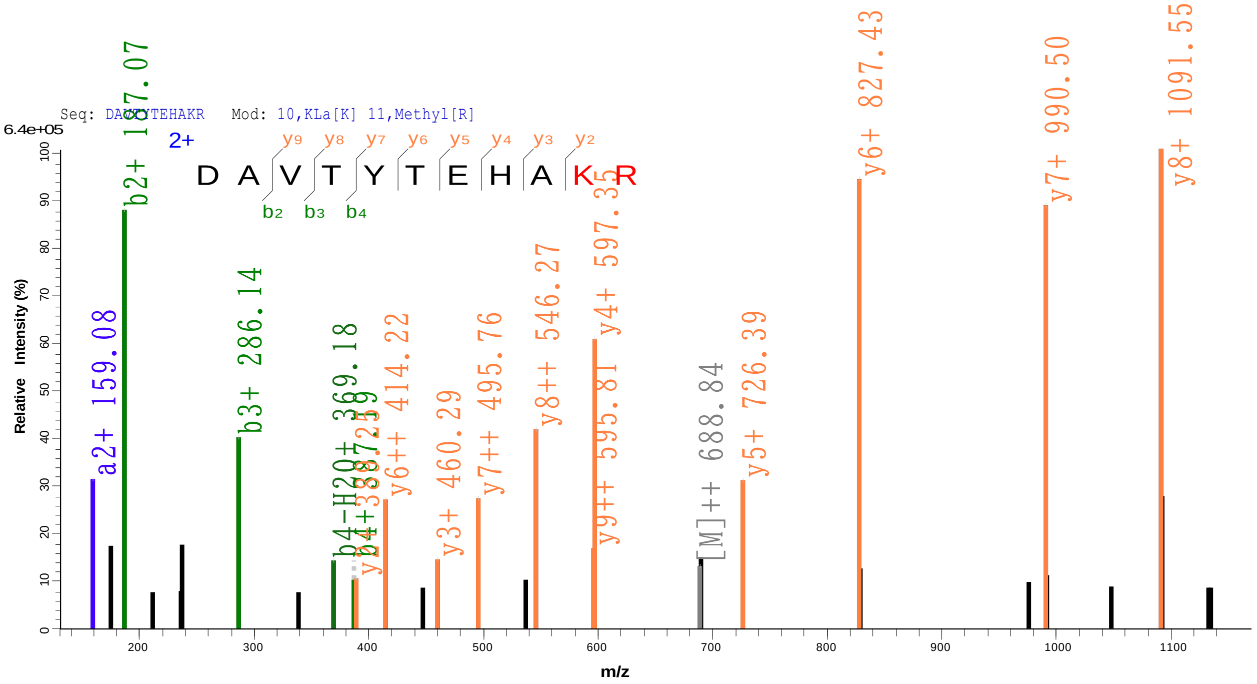
Seq: DAVTYTEHAKR Mod: 10,KLa[K] 11,Methyl[R]
6.4e+05
Relative Intensity (%)
m/z

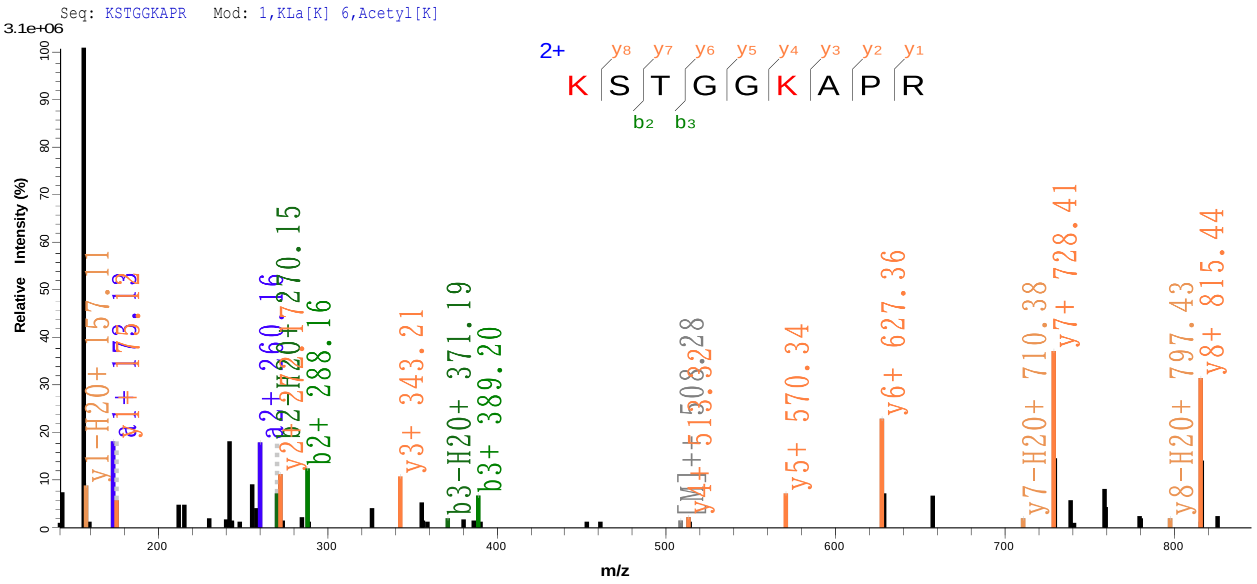
Seq: KSTGGKAPR Mod: 1,KLa[K] 6,Acetyl[K]
3.1e+06
Relative Intensity (%)
m/z

Data S3. All PSMs identified by conventional strategy but not CHiMA when analyzing previous Kla, Kbz, Khib, and Kcr datasets.

Kla

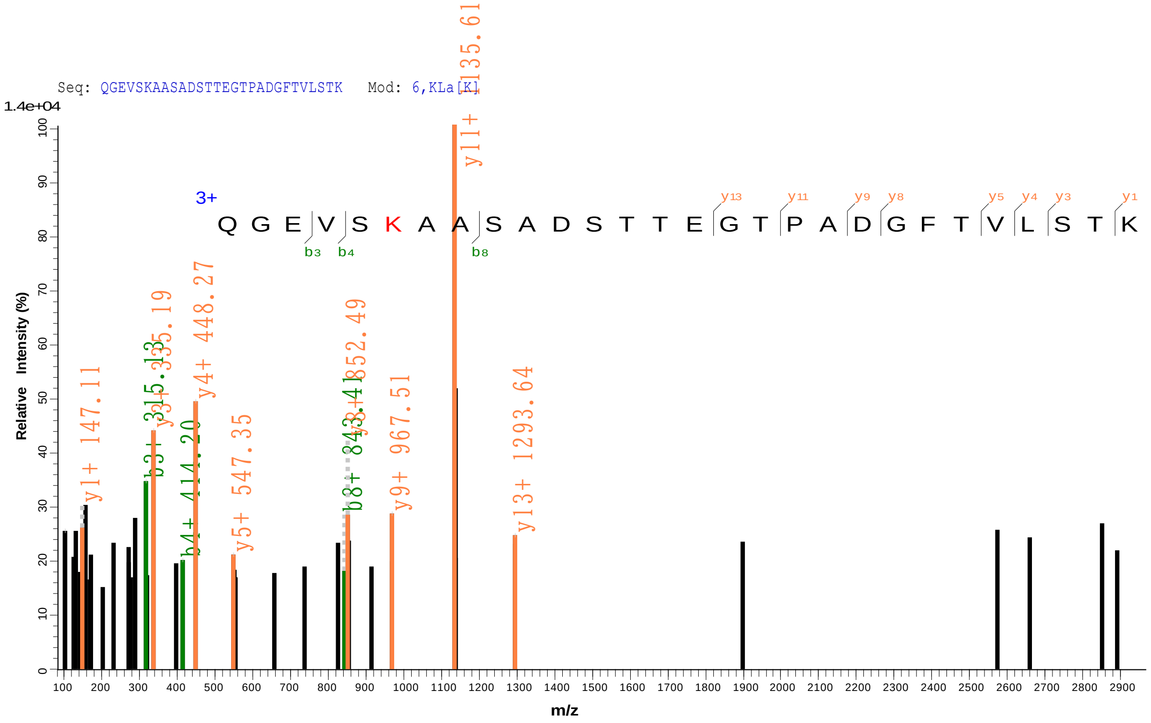

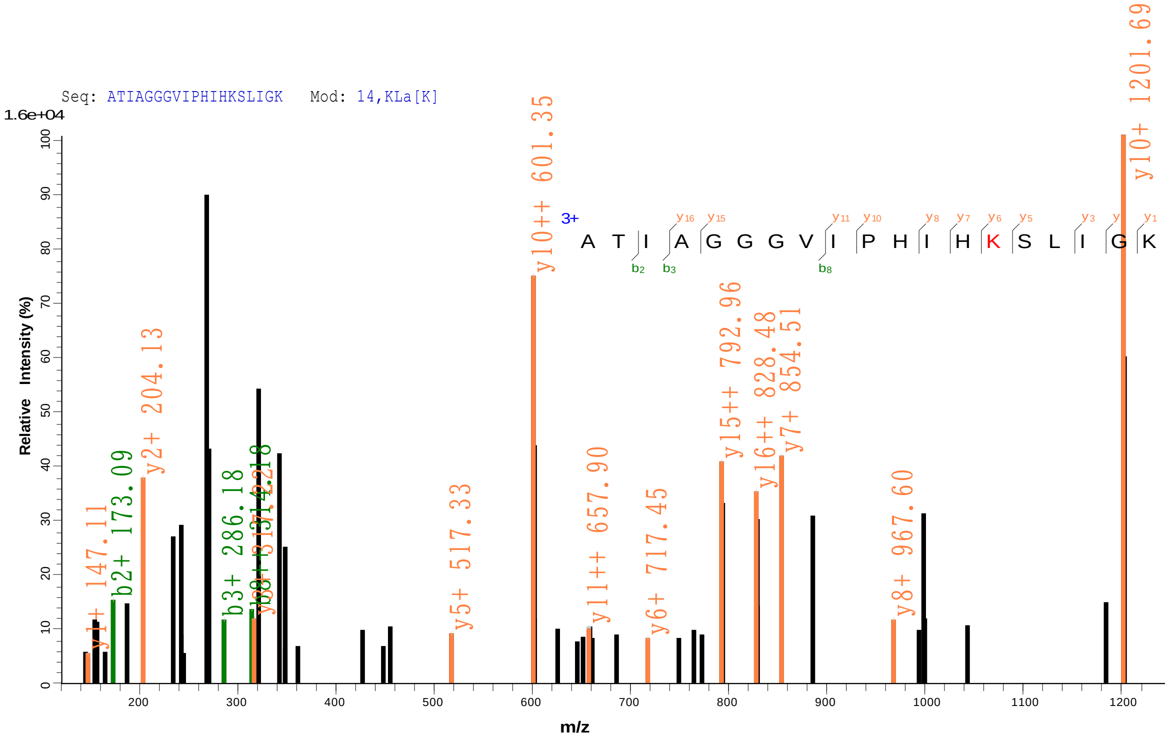

Seq: GAPAAATAPAPTAHKAK   Mod: 15,KLa[K]

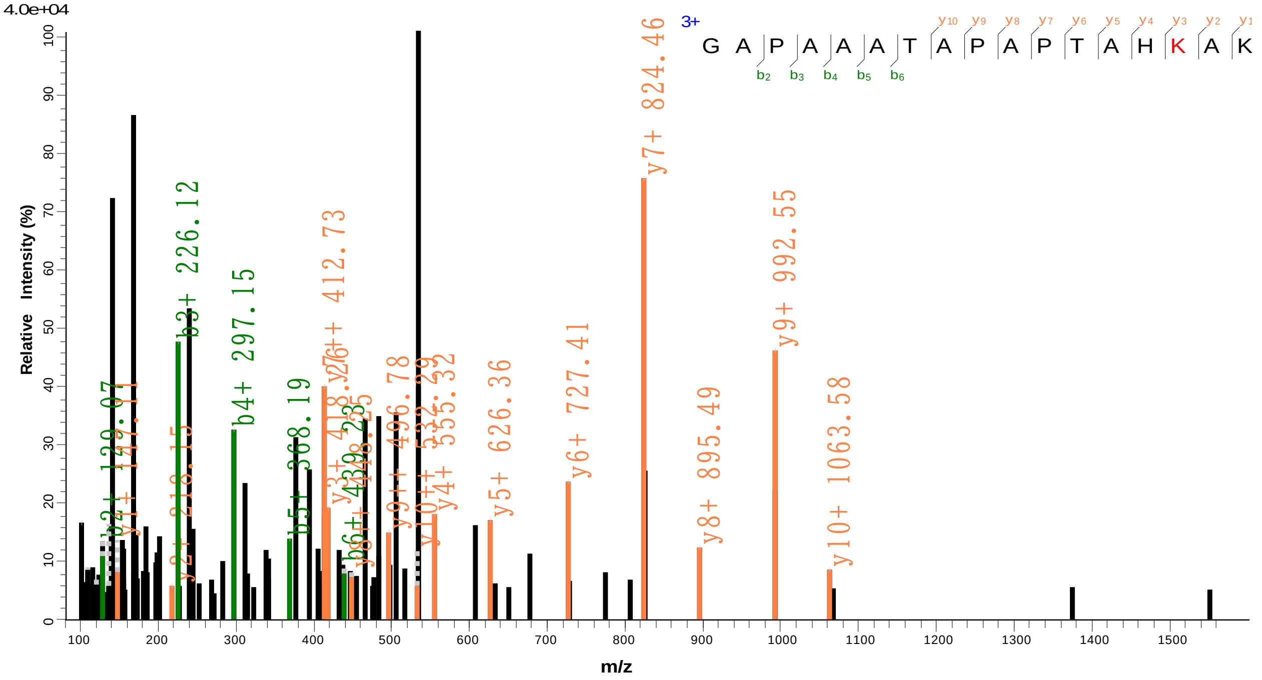

Seq: ESYSIYIYKVLK   Mod: 9,KLa[K]

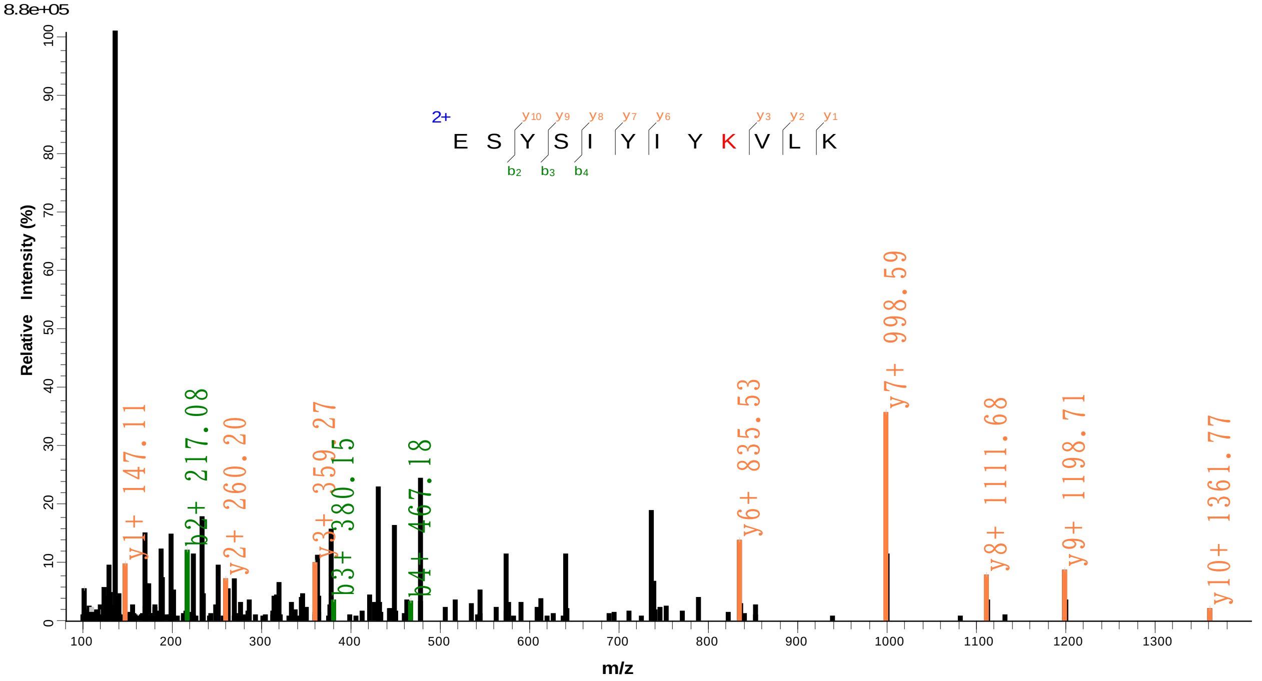

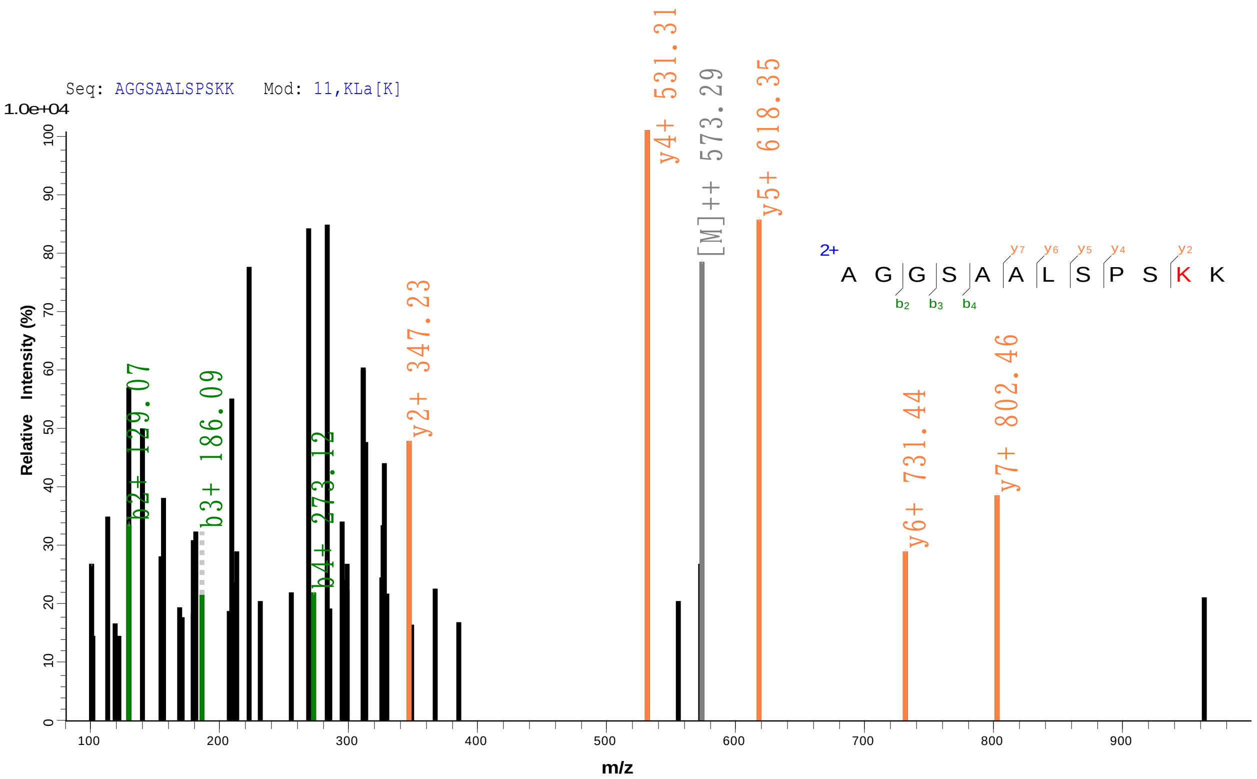
Seq: AGGSAALSPSKK Mod: 11,KLa[K]
1.0e+04
Relative Intensity (%)
m/z
y4+ 531.31
[M]++ 573.29
y5+ 618.35
y2+ 347.23
b2+ 129.07
b3+ 186.09
b4+ 273.12
y6+ 731.44
y7+ 802.46
2+
A G G S A A L S P S K K
b2 b3 b4
y7 y6 y5 y4 y2

Kbz

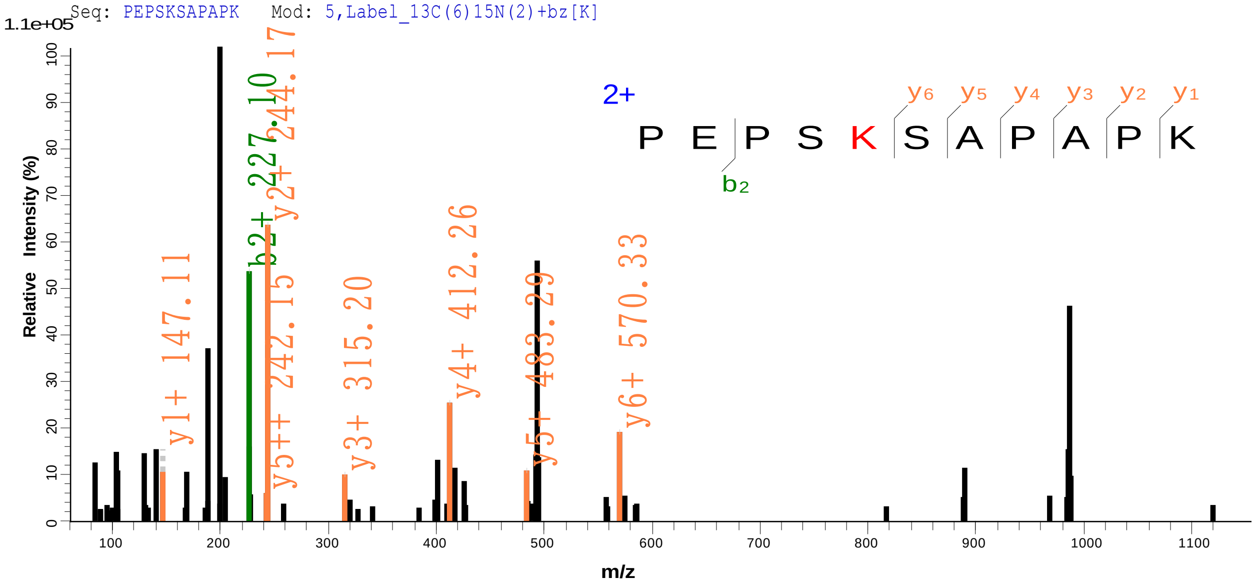
Seq: PEPSKSAPAPK Mod: 5,Label_13C(6)15N(2)+bz[K]
1.1e+05
Relative Intensity (%)
m/z
y1+ 147.11
b2+ 227.10
y2+ 244.17
y5++ 242.15
y3+ 315.20
y4+ 412.26
y5+ 483.29
y6+ 570.33
2+
P E P S K S A P A P K
b2
y6 y5 y4 y3 y2 y1

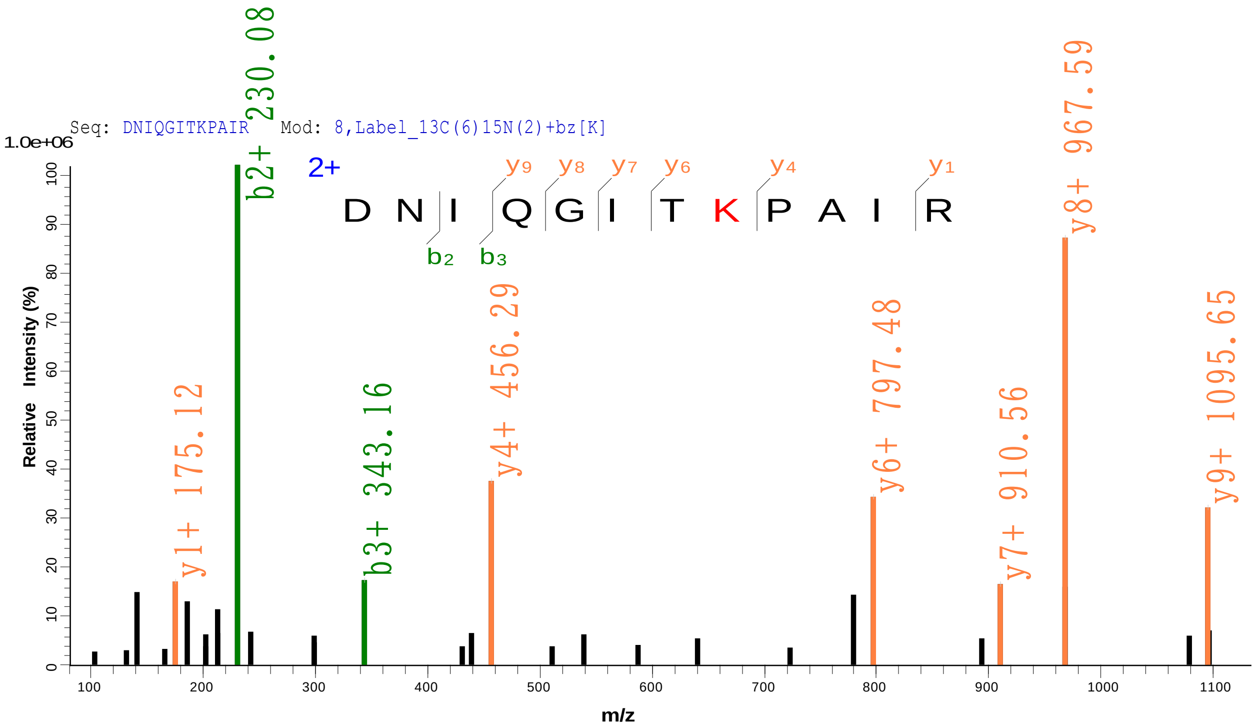
Seq: DNIQGITKPAIR Mod: 8,Label_13C(6)15N(2)+bz[K]
1.0e+06
2+
D N I Q G I T K P A I R
b2 b3
y9 y8 y7 y6 y4 y1
b2+ 230.08
y1+ 175.12
b3+ 343.16
y4+ 456.29
y6+ 797.48
y7+ 910.56
y8+ 967.59
y9+ 1095.65
Relative Intensity (%)
m/z

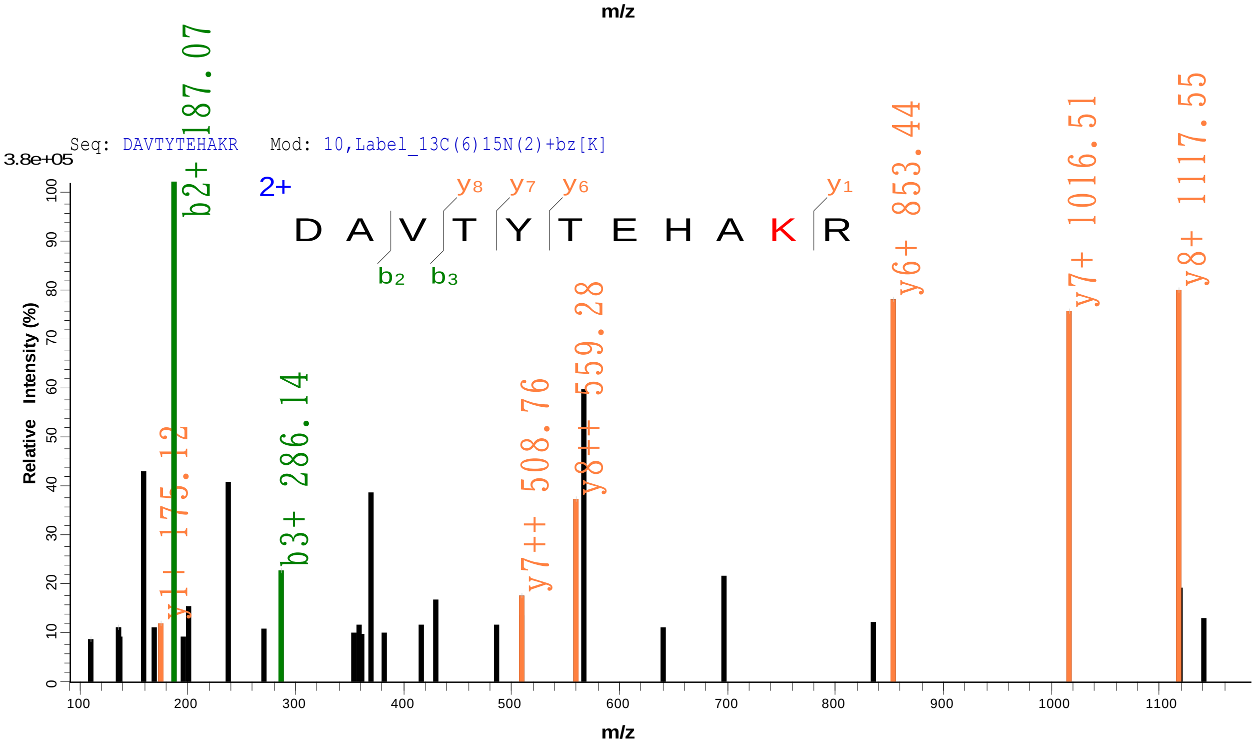
Seq: DAVTYTEHAKR Mod: 10,Label_13C(6)15N(2)+bz[K]
3.8e+05
2+
D A V T Y T E H A K R
b2 b3
y8 y7 y6 y1
b2+ 187.07
y1+ 175.12
b3+ 286.14
y7++ 508.76
y8++ 559.28
y6+ 853.44
y7+ 1016.51
y8+ 1117.55
Relative Intensity (%)
m/z

Khib

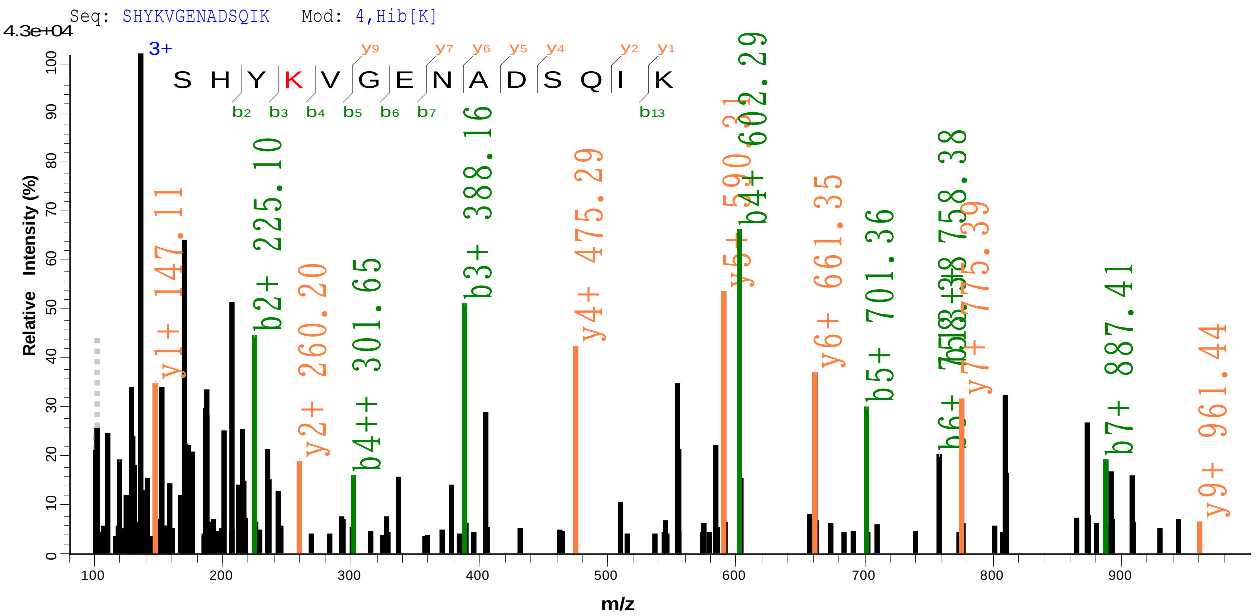
Seq: SHYKVGENADSQIK   Mod: 4,Hib[K]
4.3e+04
Relative Intensity (%)
m/z

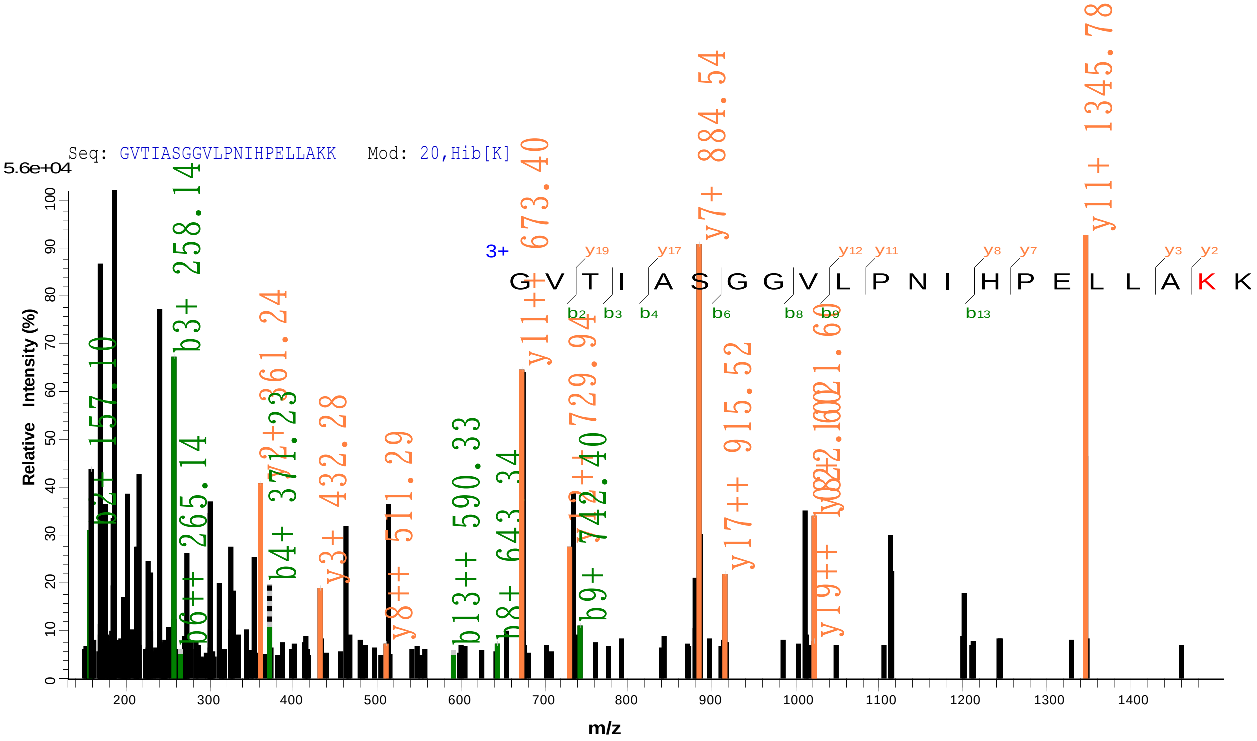
Seq: GVTIASGGVLPNIHPELLAKK   Mod: 20,Hib[K]
5.6e+04
Relative Intensity (%)
m/z

Kcr

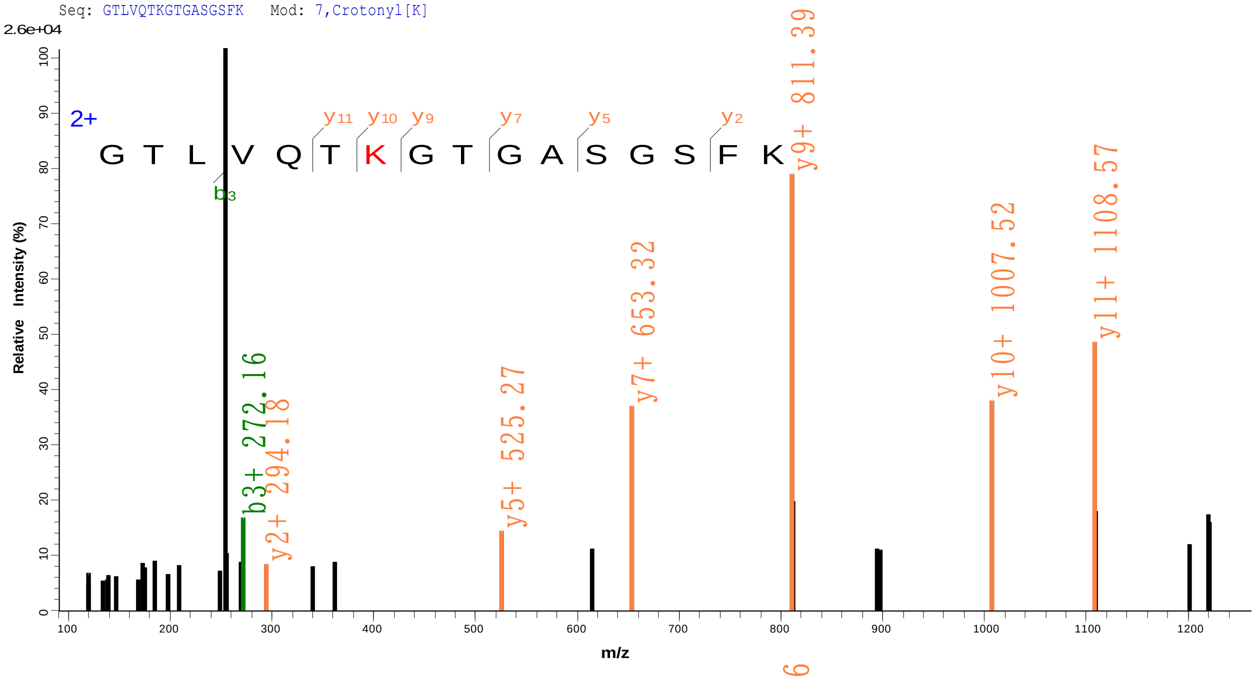
Seq: GTLVQTKGTGASGSFK Mod: 7,Crotonyl[K]
2.6e+04
Relative Intensity (%)
2+
G T L V Q T K G T G A S G S F K
y11 y10 y9 y7 y5 y2
b3
b3+ 272.16
y2+ 294.18
y5+ 525.27
y7+ 653.32
y9+ 811.39
y10+ 1007.52
y11+ 1108.57
m/z

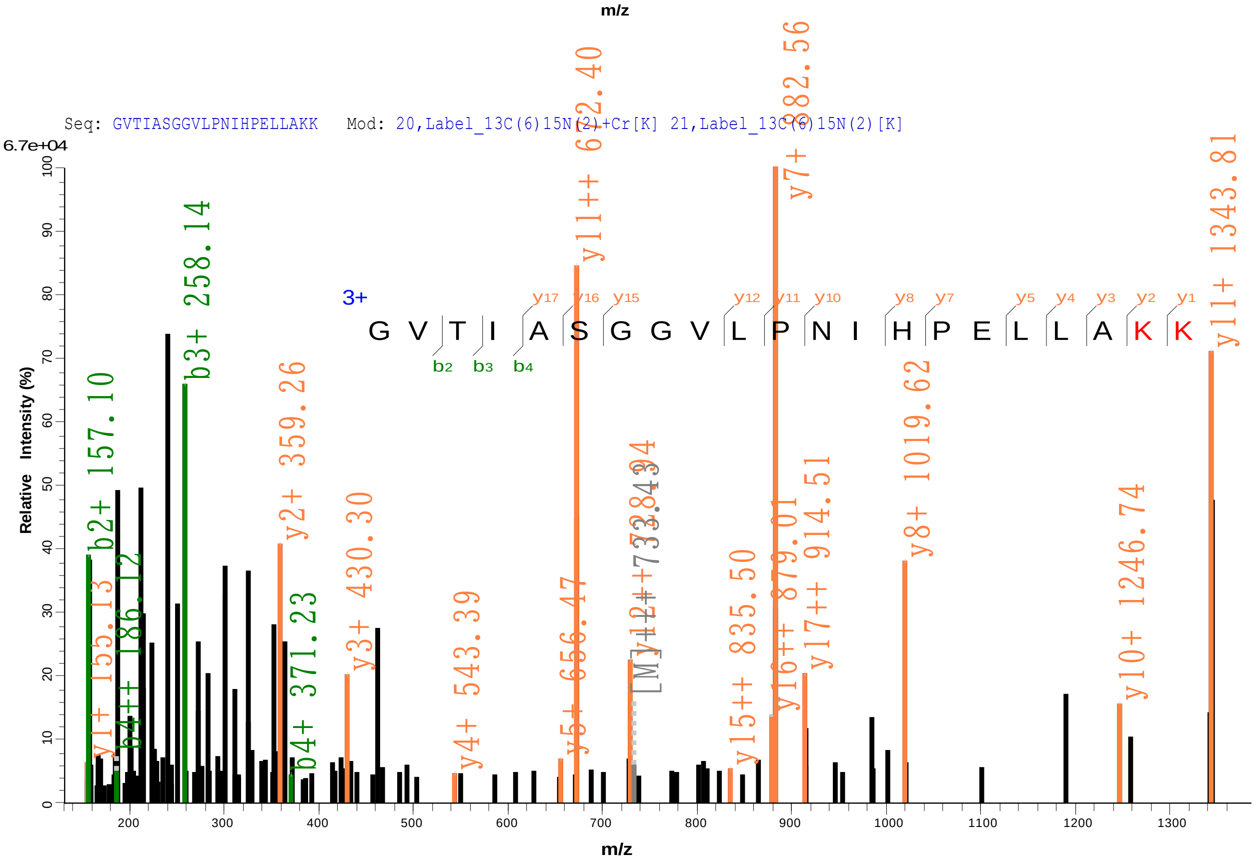
Seq: GVTIASGGVLPNIHPELLAKK Mod: 20,Label_13C(6)15N(2)+Cr[K] 21,Label_13C(6)15N(2)[K]
6.7e+04
Relative Intensity (%)
3+
G V T I A S G G V L P N I H P E L L A K K
y17 y16 y15 y12 y11 y10 y8 y7 y5 y4 y3 y2 y1
b2 b3 b4
y1+ 155.13
b2+ 157.10
b4++ 186.12
b3+ 258.14
y2+ 359.26
b4+ 371.23
y3+ 430.30
y4+ 543.39
y5+ 656.47
y11++ 672.40
y12++ 728.94
[M]+++ 733.43
y15++ 835.50
y16++ 879.01
y7+ 882.56
y17++ 914.51
y8+ 1019.62
y10+ 1246.74
y11+ 1343.81
m/z

Seq: SAKAGVIFPVGR Mod: 3,Crotonyl[K]

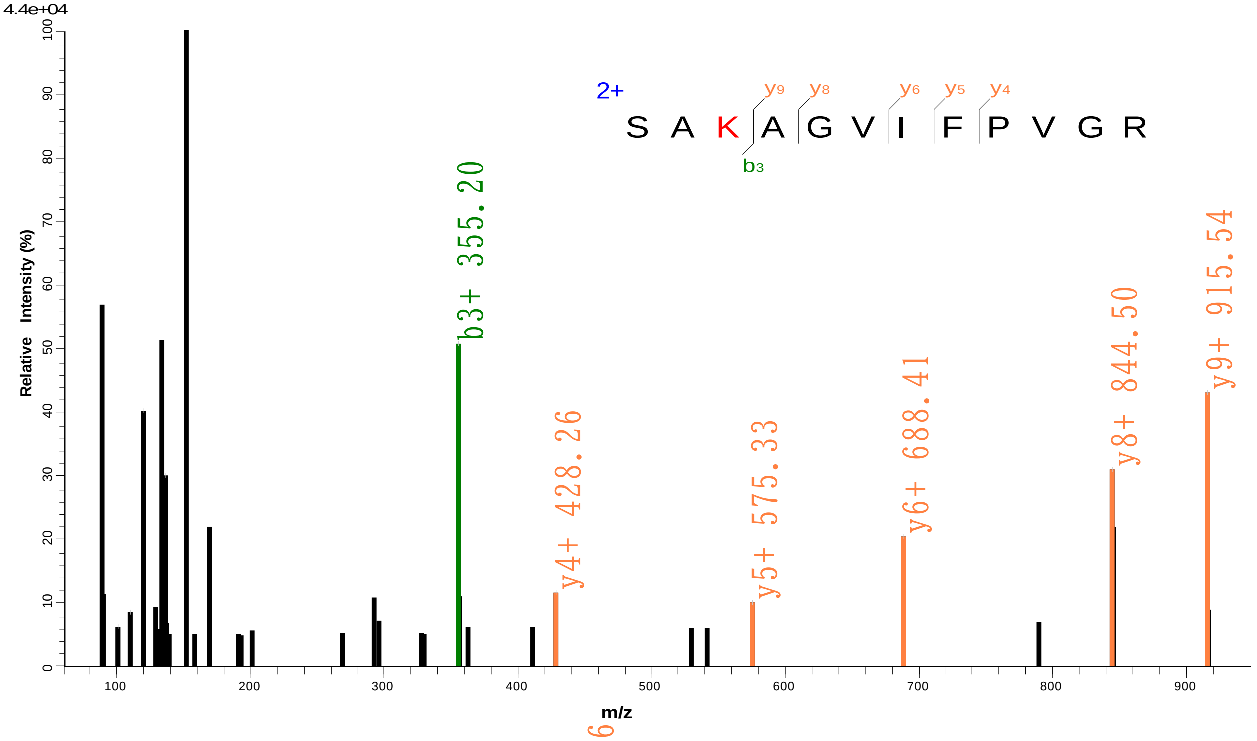

Seq: ATIAGGGVIPHIHKSLIGK Mod: 14,Crotonyl[K]

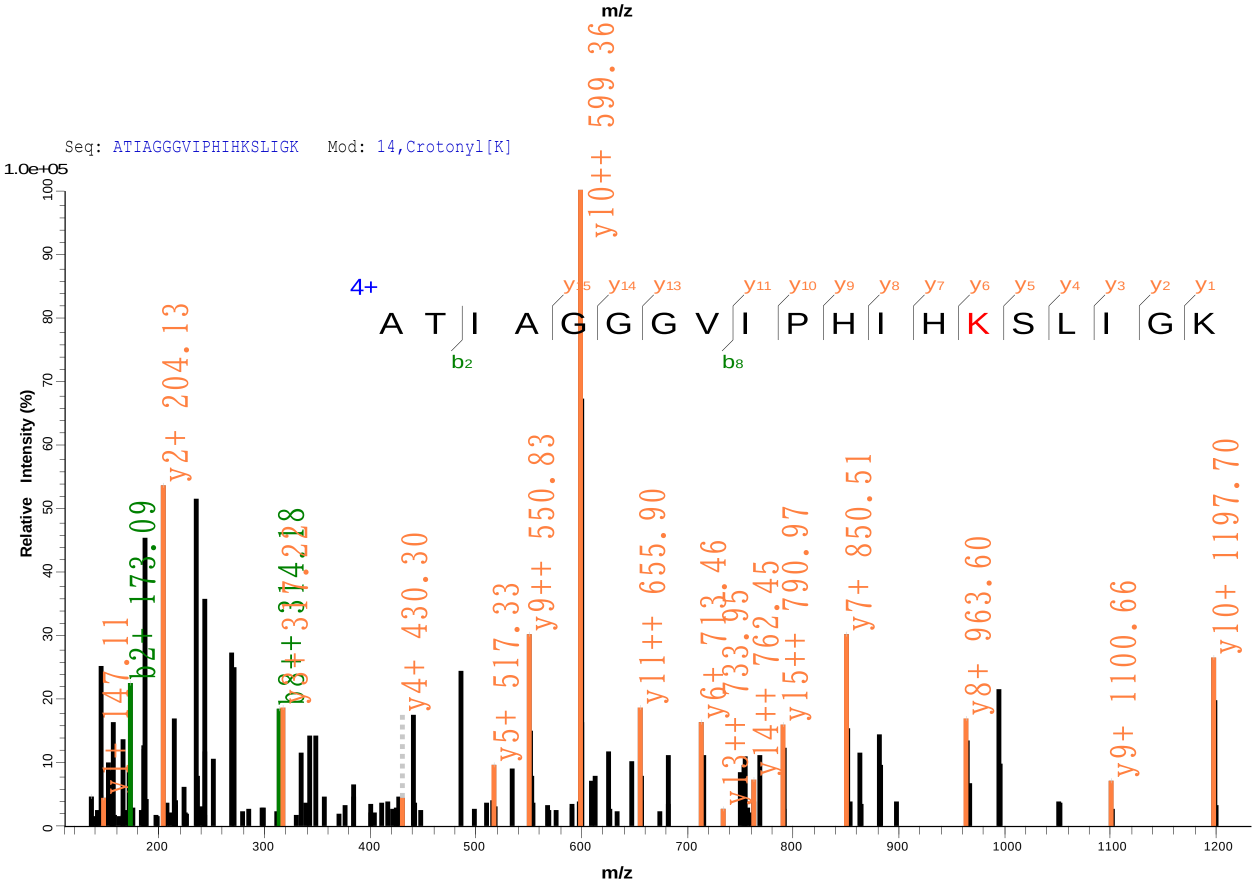

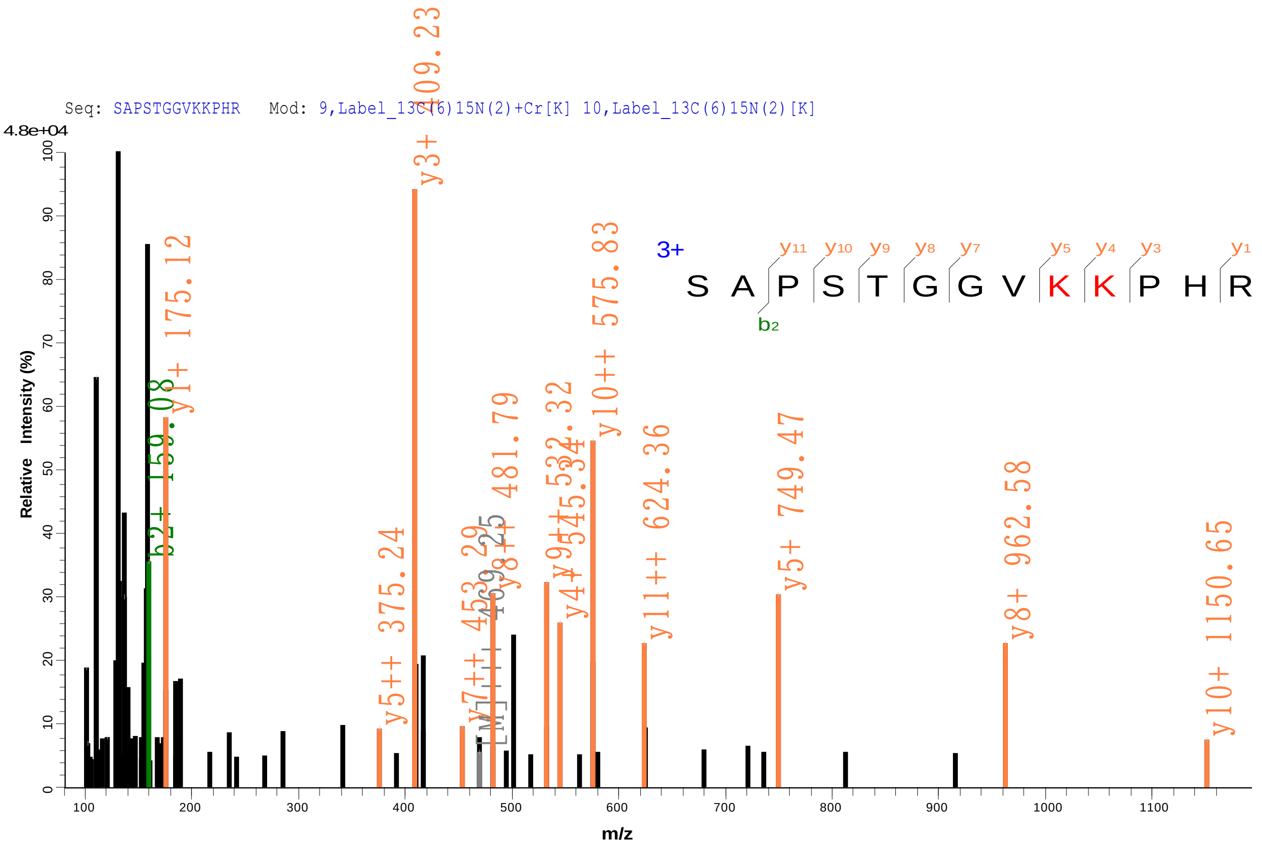

Seq: SAPSTGGVKKPHR Mod: 9,Label_13C(6)15N(2)+Cr[K] 10,Label_13C(6)15N(2)[K]
4.8e+04
Relative Intensity (%)
m/z
3+
S A P S T G G V K K P H R
y11 y10 y9 y8 y7 y5 y4 y3 y1
b2
y3+ 409.23
y1+ 175.12
b2+ 159.08
y5++ 375.24
y7++ 453.29
[M]+++ 469.25
y8++ 481.79
y9++ 532.32
y4+ 545.34
y10++ 575.83
y11++ 624.36
y5+ 749.47
y8+ 962.58
y10+ 1150.65

Data S4. All moderate-confidence PSMs for newly identified Kla and Kcr sites were validated using synthetic peptides.

We chemically synthesized all Kla and Kcr peptides newly identified with moderate confidence, including 8 Kla and 10 Kcr peptides. The PSMs of synthetic peptides were compared to the originally identified PSMs. All paired spectra are perfectly matched to each other. For each comparison, the upper spectrum is the originally identified PSM, and the lower one is the PSM of the synthetic peptide.

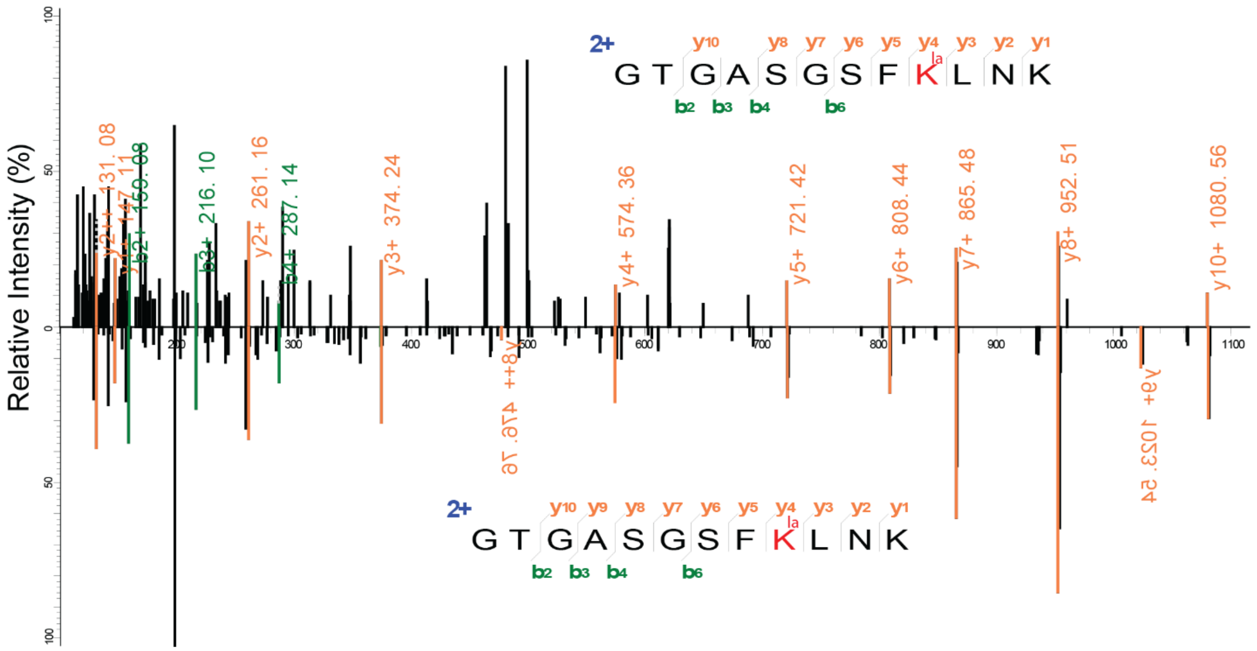

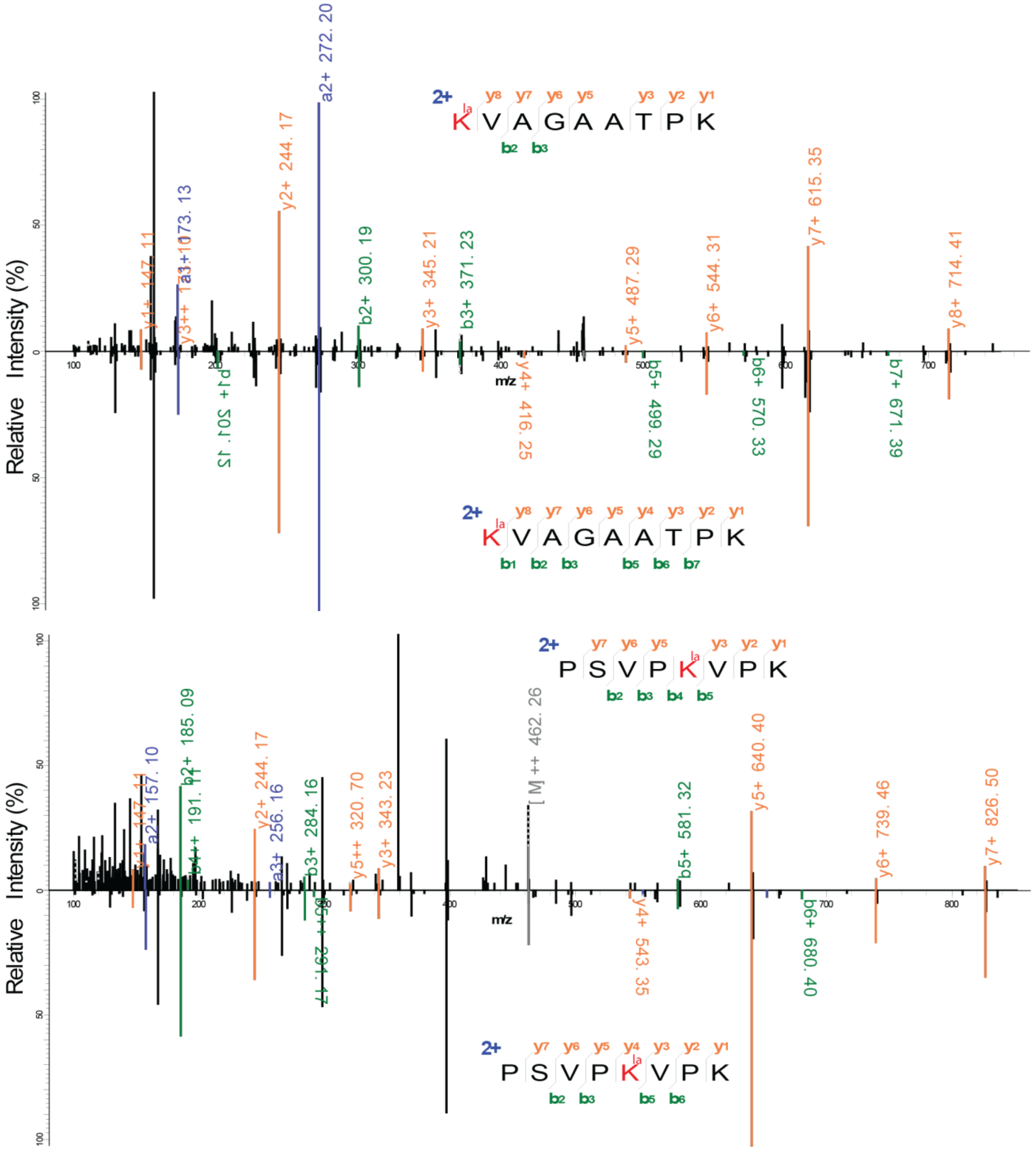

Relative Intensity (%)
m/z
2+
KVAGAATPK
PSVPKVPK
a2+ 272.20
y2+ 244.17
y7+ 615.35
b2+ 300.19
y3+ 345.21
b3+ 371.23
y5+ 487.29
y6+ 544.31
y8+ 714.41
b1+ 201.12
y4+ 416.25
b5+ 499.29
b6+ 570.33
b7+ 671.39
b2+ 185.09
a2+ 157.10
y2+ 244.17
a3+ 256.16
b3+ 284.16
y5++ 320.70
y3+ 343.23
[M]++ 462.26
b5+ 581.32
y5+ 640.40
y6+ 739.46
y7+ 826.50
y4+ 543.35
b6+ 680.40

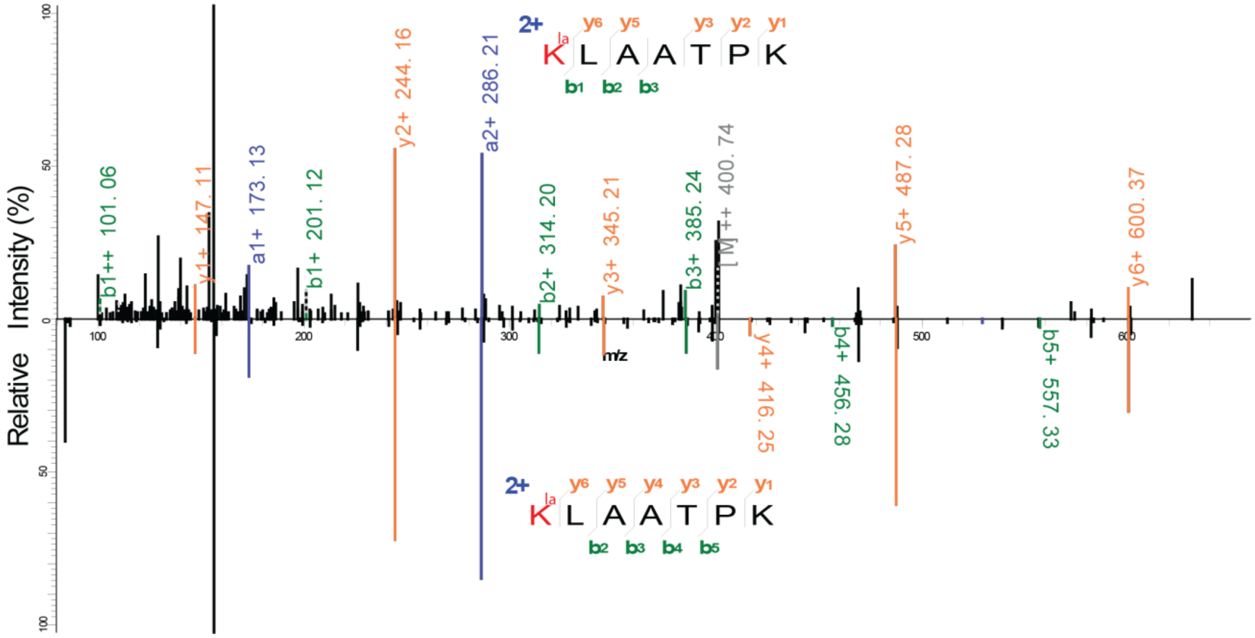
Relative Intensity (%)
100
50
0
50
100
m/z
100
200
300
400
500
600
2+ K la L A A T P K
y6 y5 y3 y2 y1
b1 b2 b3
b1++ 101. 06
y1+ 147. 11
a1+ 173. 13
b1+ 201. 12
y2+ 244. 16
a2+ 286. 21
b2+ 314. 20
y3+ 345. 21
b3+ 385. 24
[M]++ 400. 74
y5+ 487. 28
y6+ 600. 37
y4+ 416. 25
b4+ 456. 28
b5+ 557. 33
2+ K la L A A T P K
y6 y5 y4 y3 y2 y1
b2 b3 b4 b5

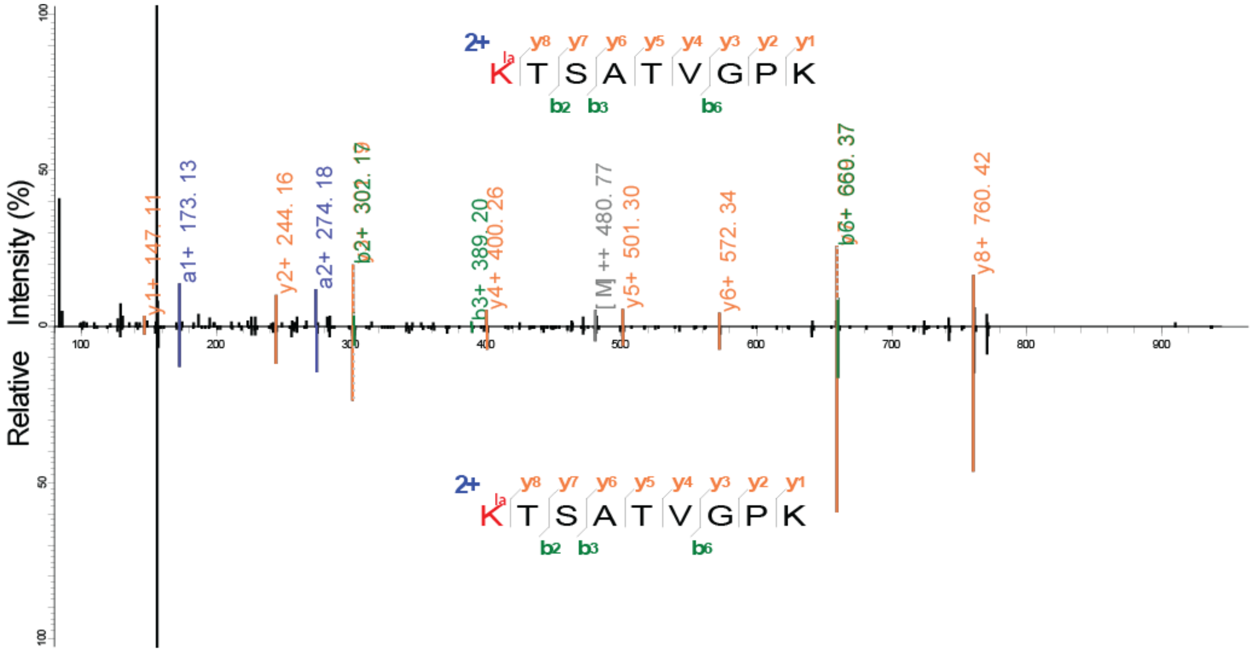
Relative Intensity (%)
100
50
0
50
100
100
200
300
400
500
600
700
800
900
2+ K la T S A T V G P K
y8 y7 y6 y5 y4 y3 y2 y1
b2 b3 b6
y1+ 147. 11
a1+ 173. 13
y2+ 244. 16
a2+ 274. 18
b2+ 302. 17
b3+ 389. 20
y4+ 400. 26
[M]++ 480. 77
y5+ 501. 30
y6+ 572. 34
b6+ 660. 37
y8+ 760. 42
2+ K la T S A T V G P K
y8 y7 y6 y5 y4 y3 y2 y1
b2 b3 b6

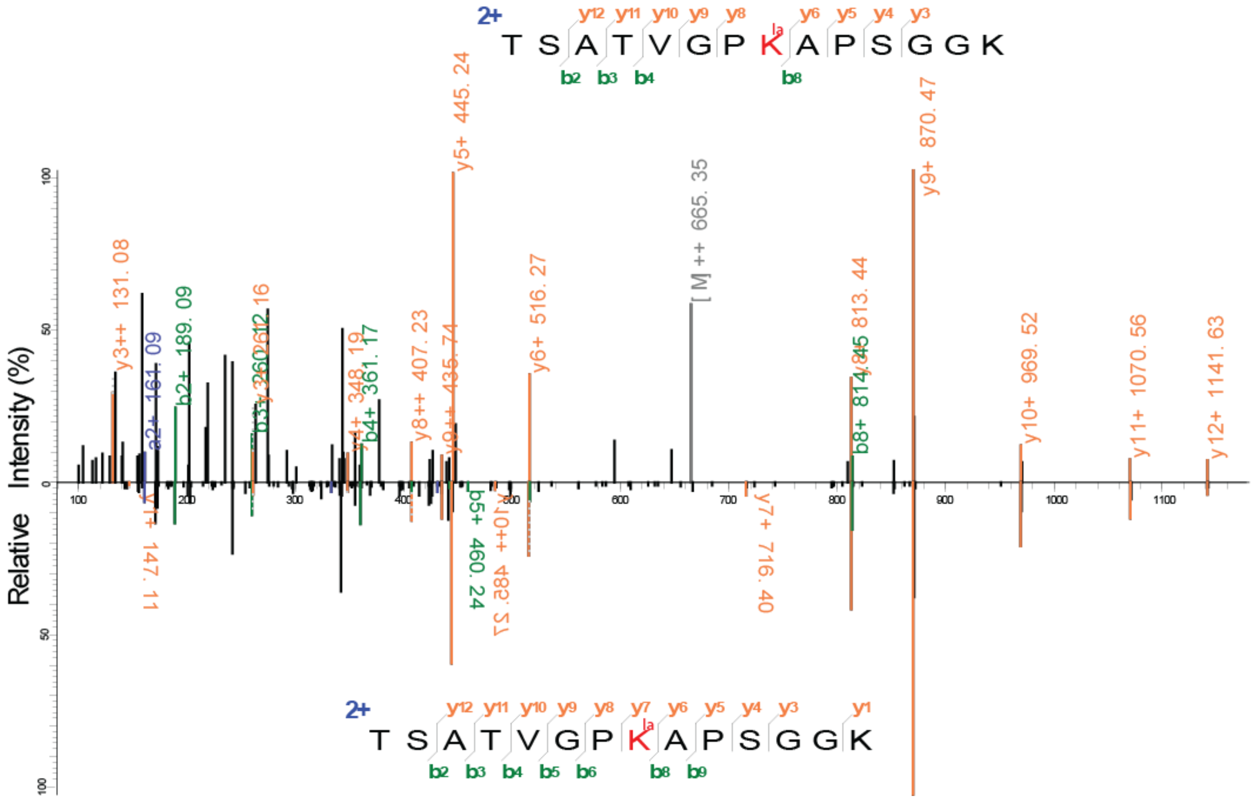
2+
TSATVGPKAPSGGK
y12 y11 y10 y9 y8 y6 y5 y4 y3
b2 b3 b4 b8
la
Relative Intensity (%)
y5+ 445.24
y9+ 870.47
[M]++ 665.35
y3++ 131.08
b2+ 189.09
361.17
y8++ 407.23
y6+ 516.27
b8+ 814.45
813.44
y10+ 969.52
y11+ 1070.56
y12+ 1141.63
147.11
b5+ 460.24
y7+ 716.40
y12 y11 y10 y9 y8 y7 y6 y5 y4 y3 y1
b2 b3 b4 b5 b6 b8 b9

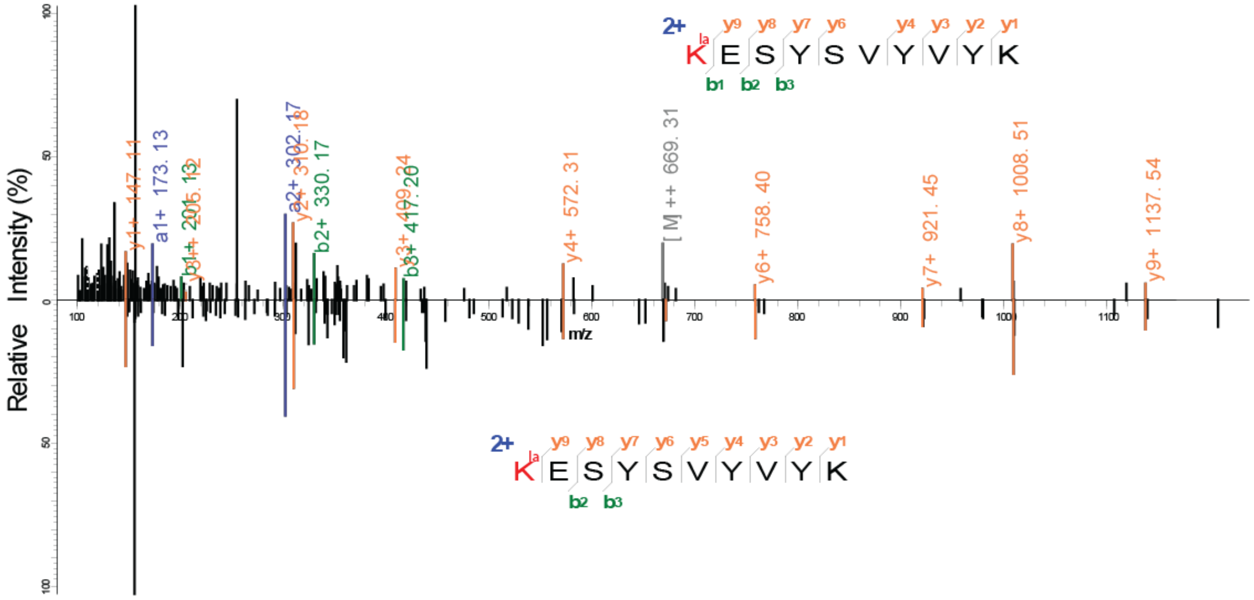
2+
KESYSVYVYK
y9 y8 y7 y6 y4 y3 y2 y1
b1 b2 b3
la
Relative Intensity (%)
a1+ 173.13
a2+ 302.17
b2+ 330.17
y4+ 572.31
[M]++ 669.31
y6+ 758.40
y7+ 921.45
y8+ 1008.51
y9+ 1137.54
m/z
y9 y8 y7 y6 y5 y4 y3 y2 y1
b2 b3

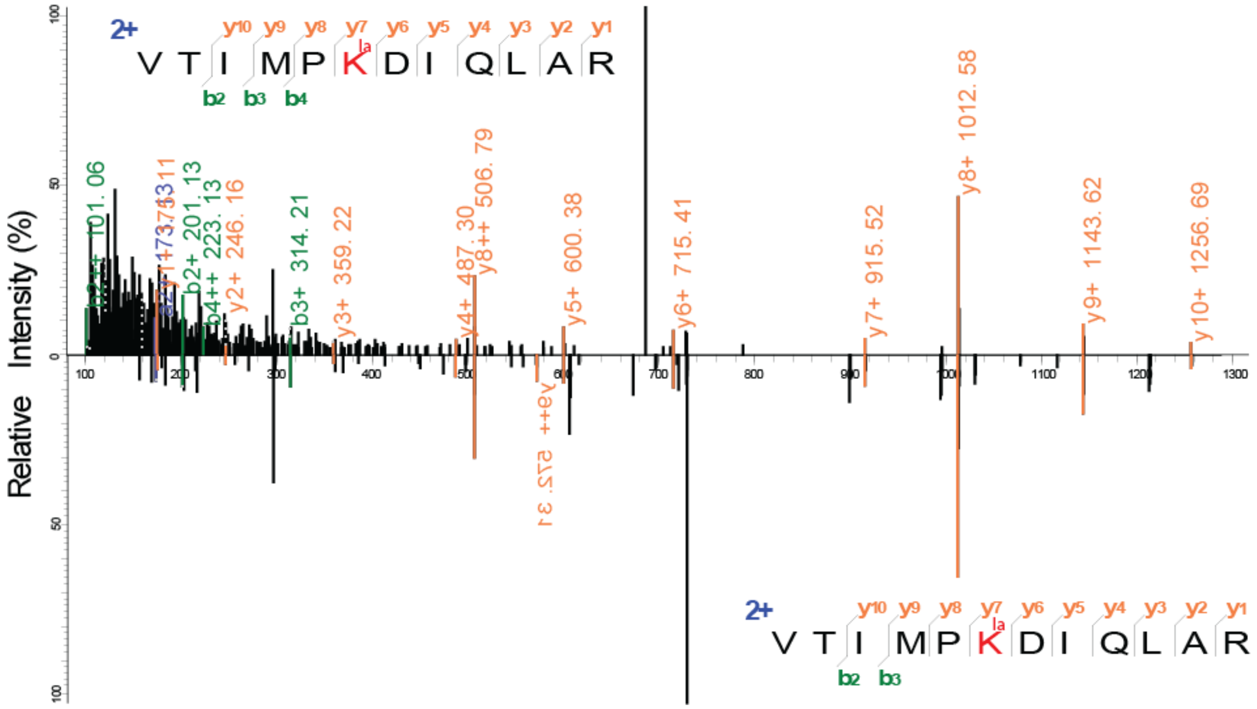
Relative Intensity (%)
2+
V T I M P K D I Q L A R
y10 y9 y8 y7 y6 y5 y4 y3 y2 y1
la
b2 b3 b4
b2+ 201. 13
b4++ 223. 13
y2+ 246. 16
b3+ 314. 21
y3+ 359. 22
y4+ 487. 30
y8++ 506. 79
y5+ 600. 38
y6+ 715. 41
y7+ 915. 52
y8+ 1012. 58
y9+ 1143. 62
y10+ 1256. 69
y8++ 512. 31
100
200
300
400
500
600
700
800
900
1000
1100
1200
1300

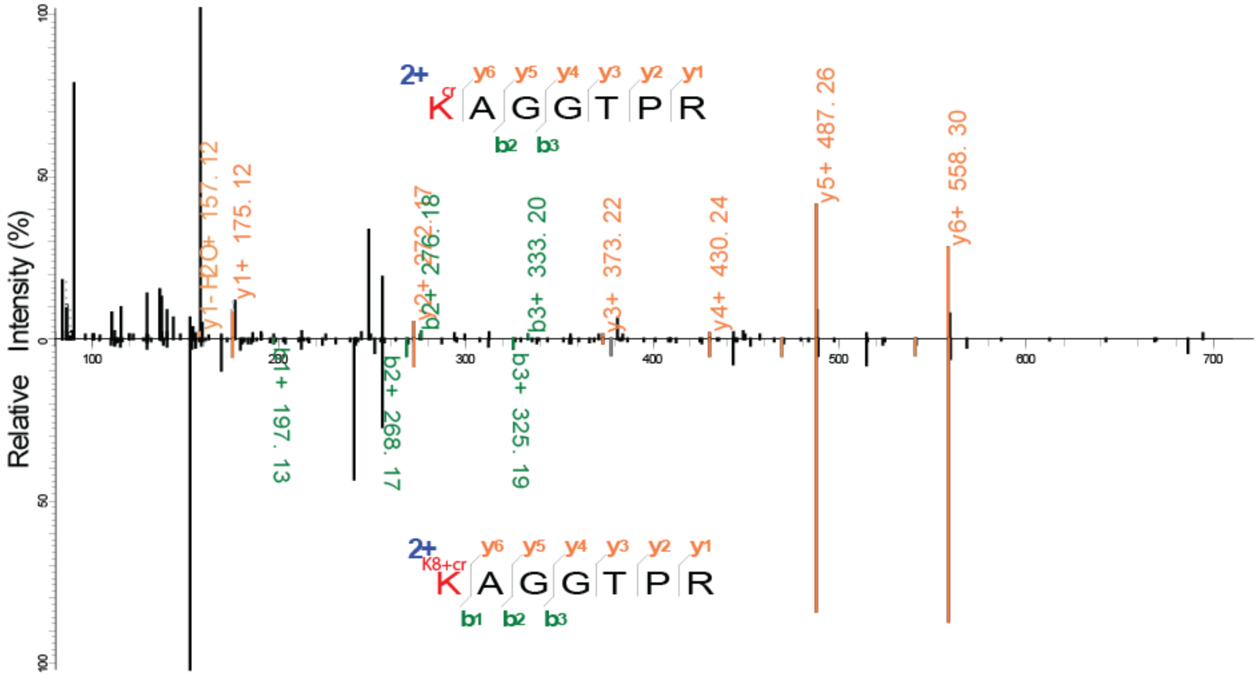
Relative Intensity (%)
2+
K A G G T P R
y6 y5 y4 y3 y2 y1
cr
b2 b3
y1- H2O+ 157. 12
y1+ 175. 12
b2+ 276. 18
b3+ 333. 20
y3+ 373. 22
y4+ 430. 24
y5+ 487. 26
y6+ 558. 30
b1+ 197. 13
b2+ 268. 17
b3+ 325. 19
K8+cr
b1 b2 b3
100
200
300
400
500
600
700

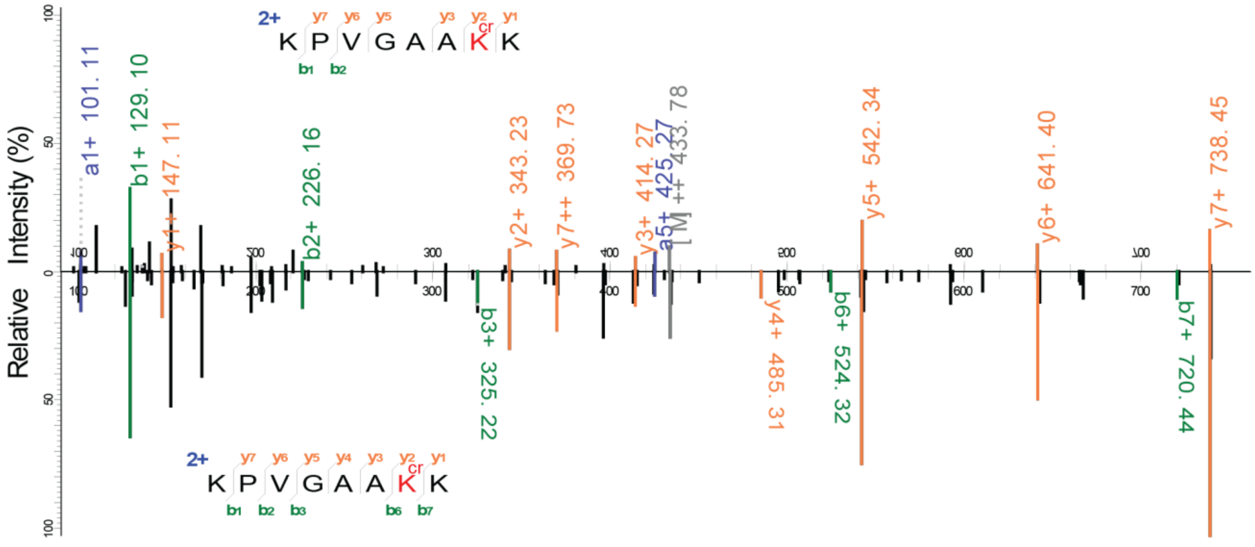

2+
y7 y6 y5 y3 y2 y1
KPVGAAKK
cr
b1 b2
Relative Intensity (%)
a1+ 101.11
b1+ 129.10
y1+ 147.11
b2+ 226.16
y2+ 343.23
y7++ 369.73
y3+ 414.27
a5+ 425.27
[M]++ 433.78
y5+ 542.34
y6+ 641.40
y7+ 738.45
b3+ 325.22
y4+ 485.31
b6+ 524.32
b7+ 720.44
2+
y7 y6 y5 y4 y3 y2 y1
KPVGAAKK
cr
b1 b2 b3 b6 b7

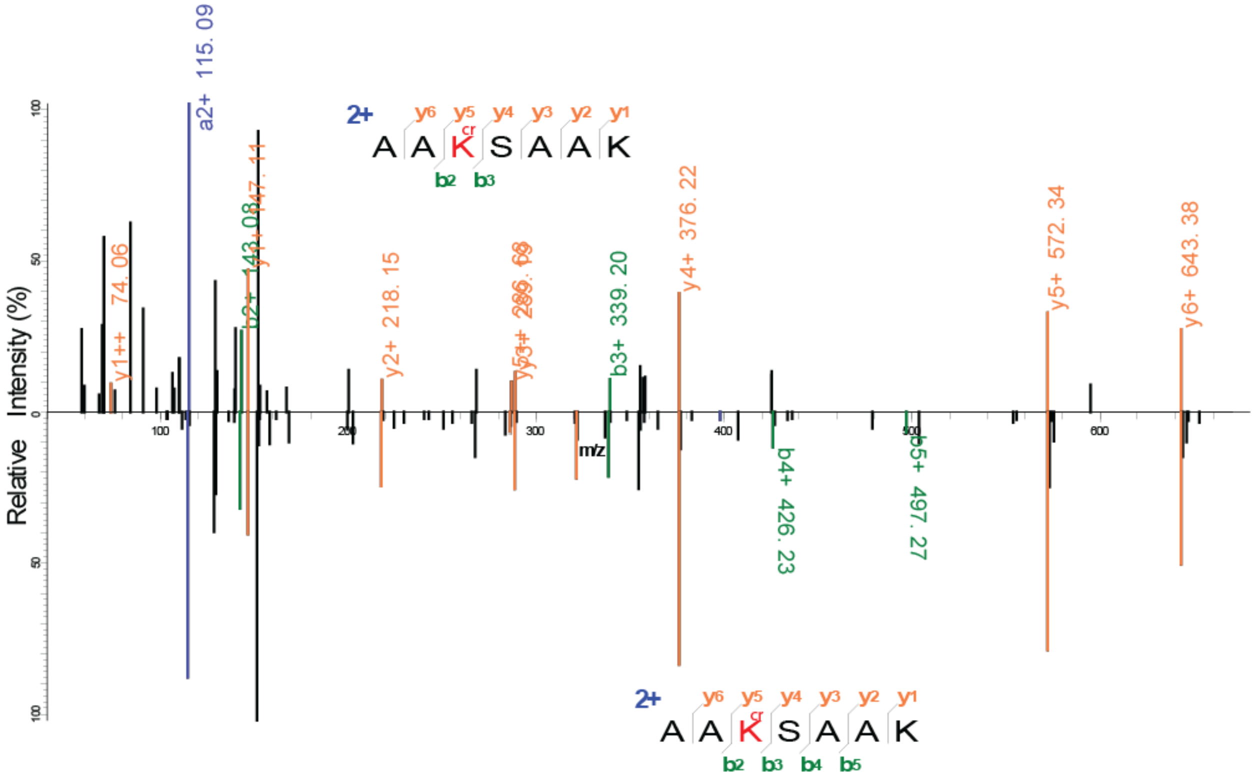

a2+ 115.09
2+
y6 y5 y4 y3 y2 y1
AAKSAAK
cr
b2 b3
Relative Intensity (%)
y1++ 74.06
b2+ 143.08
y1+ 147.11
y2+ 218.15
y3+ 289.19
y4+ 376.22
b3+ 339.20
y5+ 572.34
y6+ 643.38
m/z
b4+ 426.23
b5+ 497.27
2+
y6 y5 y4 y3 y2 y1
AAKSAAK
cr
b2 b3 b4 b5

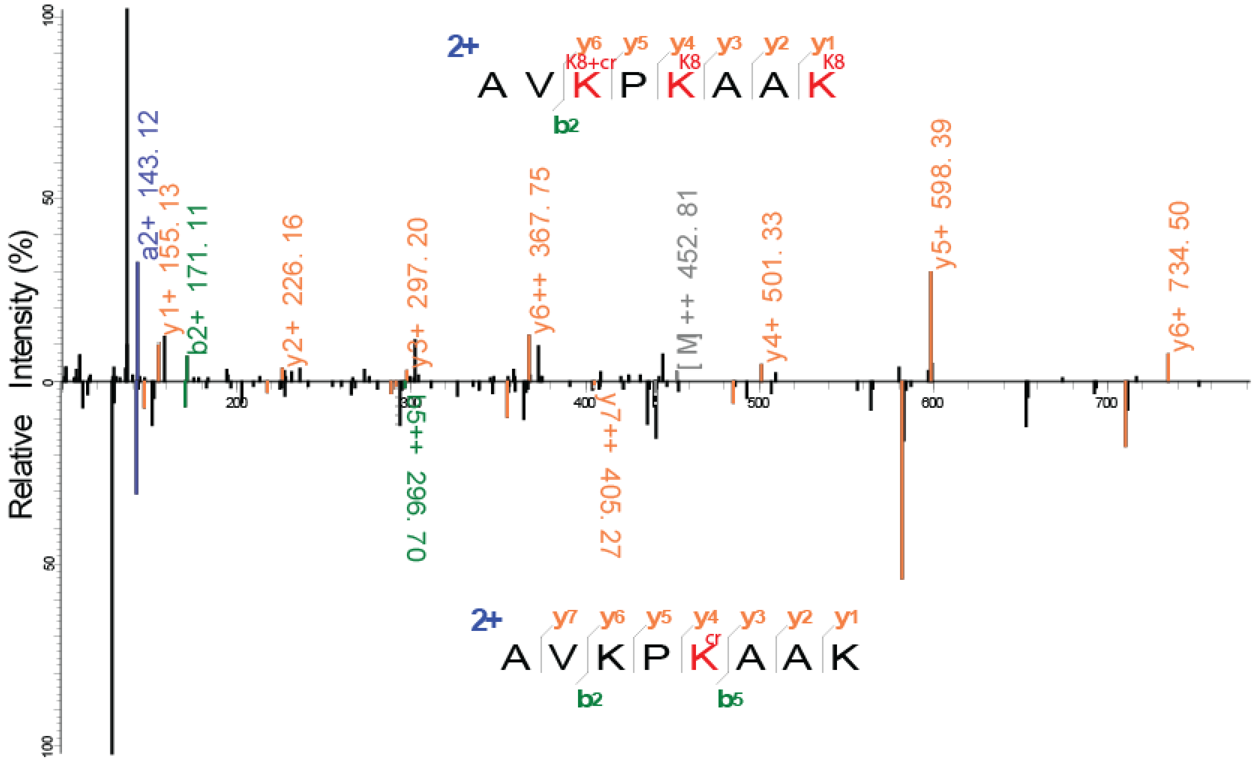
2+
A V K P K A A K
y6 y5 y4 y3 y2 y1
K8+cr K8 K8
b2
a2+ 143. 12
y1+ 155. 13
b2+ 171. 11
y2+ 226. 16
y3+ 297. 20
y6++ 367. 75
[M]++ 452. 81
y4+ 501. 33
y5+ 598. 39
y6+ 734. 50
b5++ 296. 70
y7++ 405. 27
A V K P K A A K
y7 y6 y5 y4 y3 y2 y1
cr
b2 b5
Relative Intensity (%)

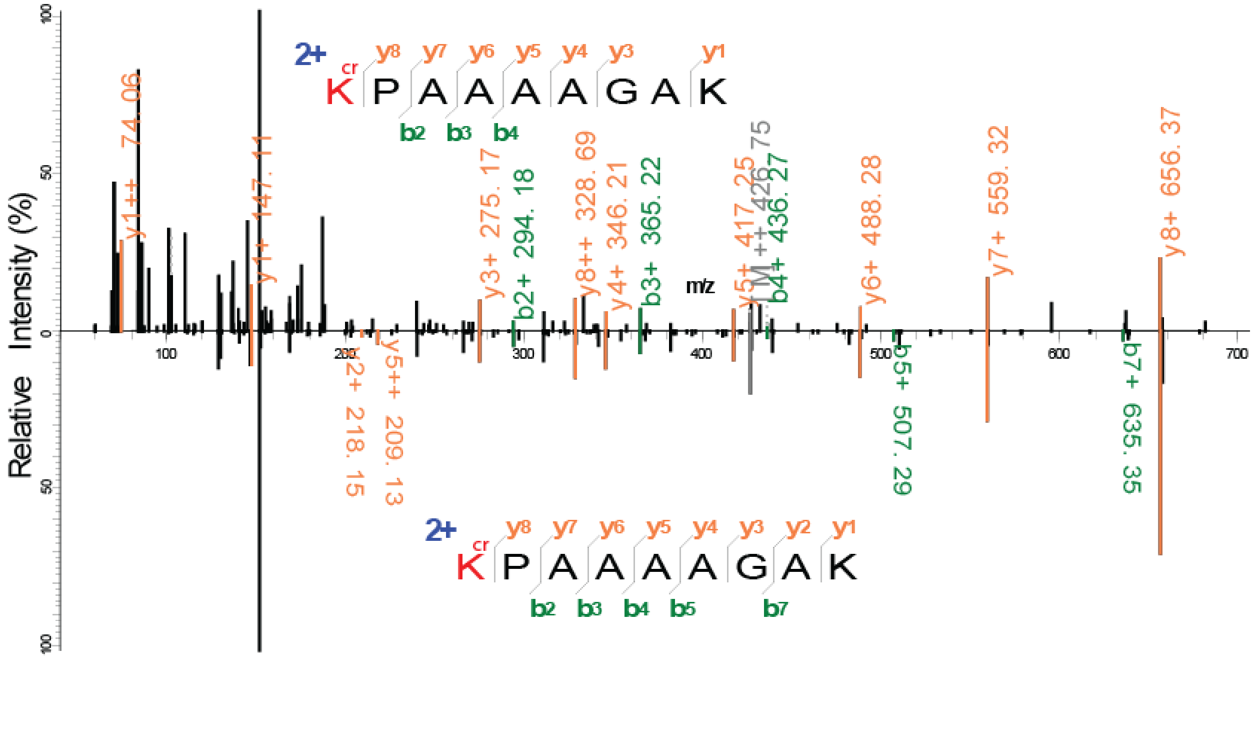
2+
K P A A A A G A K
cr
y8 y7 y6 y5 y4 y3 y1
b2 b3 b4
y1++ 74. 06
y1+ 147. 11
y3+ 275. 17
b2+ 294. 18
y8++ 328. 69
y4+ 346. 21
b3+ 365. 22
y5+ 417. 25
b4+ 436. 27
y6+ 488. 28
y7+ 559. 32
y8+ 656. 37
m/z
y2+ 218. 15
y5++ 209. 13
b5+ 507. 29
b7+ 635. 35
K P A A A A G A K
y8 y7 y6 y5 y4 y3 y2 y1
b2 b3 b4 b5 b7
Relative Intensity (%)

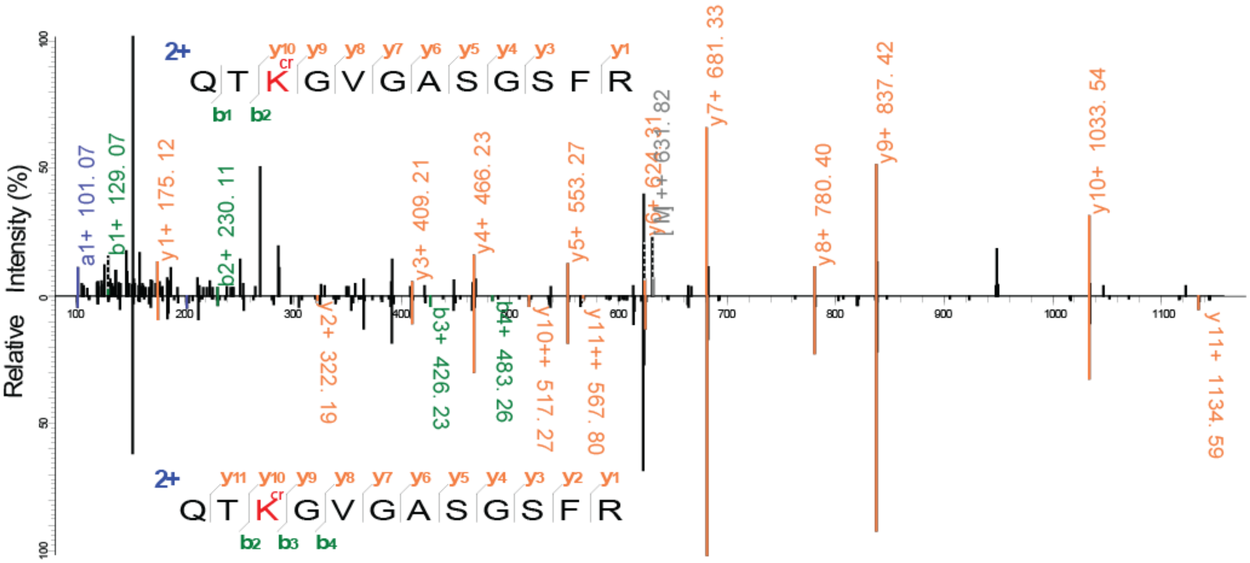

Relative Intensity (%)
2+
Q T K G V G A S G S F R
cr
y10 y9 y8 y7 y6 y5 y4 y3 y1
b1 b2
a1+ 101. 07
b1+ 129. 07
y1+ 175. 12
b2+ 230. 11
y3+ 409. 21
y4+ 466. 23
y5+ 553. 27
y7+ 681. 33
y8+ 780. 40
y9+ 837. 42
y10+ 1033. 54
y2+ 322. 19
b3+ 426. 23
b4+ 483. 26
y10++ 517. 27
y11++ 567. 80
y11+ 1134. 59
631. 82
y11 y10 y9 y8 y7 y6 y5 y4 y3 y2 y1
b2 b3 b4

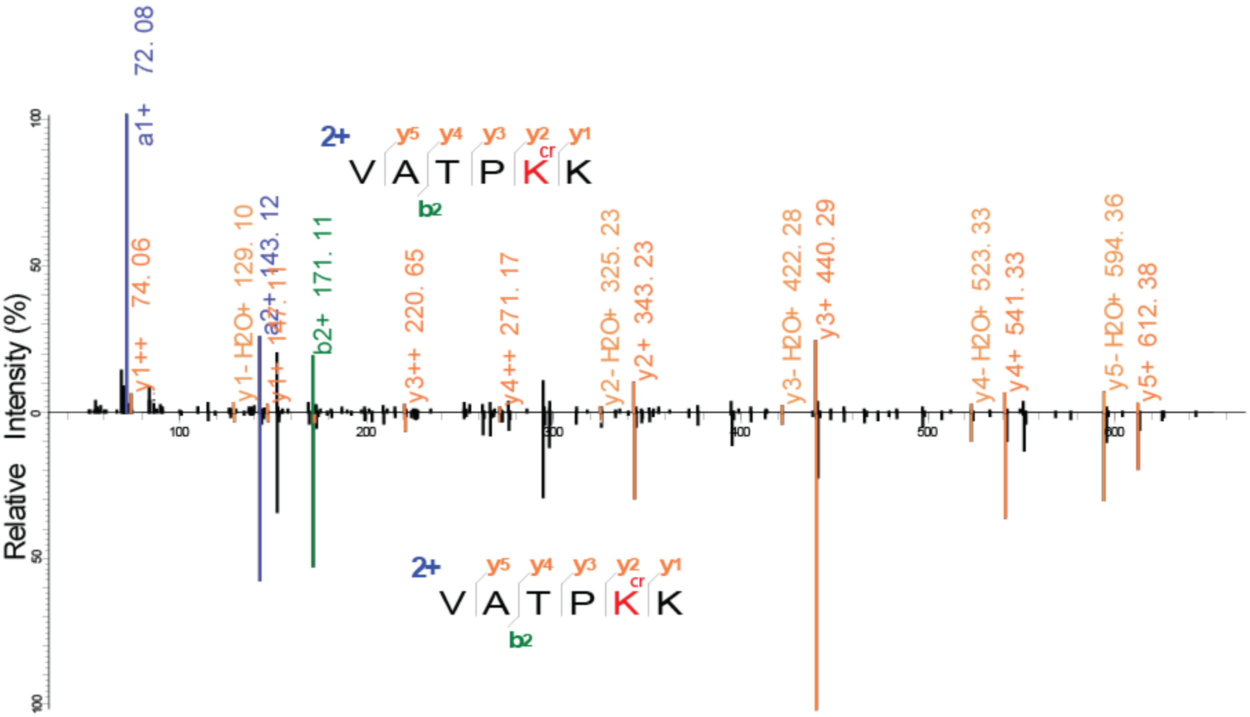

Relative Intensity (%)
2+
V A T P K K
cr
y5 y4 y3 y2 y1
b2
a1+ 72. 08
y1++ 74. 06
y1- H2O+ 129. 10
a2+ 143. 12
b2+ 171. 11
y3++ 220. 65
y4++ 271. 17
y2- H2O+ 325. 23
y2+ 343. 23
y3- H2O+ 422. 28
y3+ 440. 29
y4- H2O+ 523. 33
y4+ 541. 33
y5- H2O+ 594. 36
y5+ 612. 38

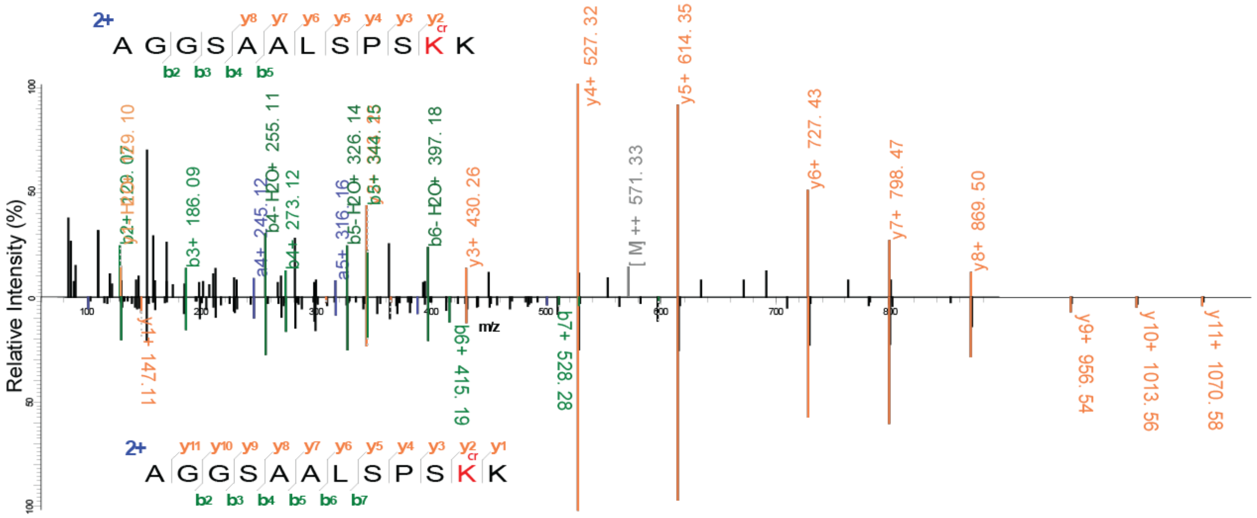
2+
A G G S A A L S P S K K
cr
y8 y7 y6 y5 y4 y3 y2
b2 b3 b4 b5
Relative Intensity (%)
m/z
y4+ 527. 32
y5+ 614. 35
[M]++ 571. 33
y6+ 727. 43
y7+ 798. 47
y8+ 869. 50
y3+ 430. 26
b6- H2O+ 397. 18
b3+ 186. 09
b4- H2O+ 255. 11
b4+ 273. 12
a4+ 245. 12
a5+ 316. 16
b5- H2O+ 326. 14
b5+ 344. 15
y1+ 147. 11
b6+ 415. 19
b7+ 528. 28
y9+ 956. 54
y10+ 1013. 56
y11+ 1070. 58
2+
A G G S A A L S P S K K
y11 y10 y9 y8 y7 y6 y5 y4 y3 y2 y1
b2 b3 b4 b5 b6 b7

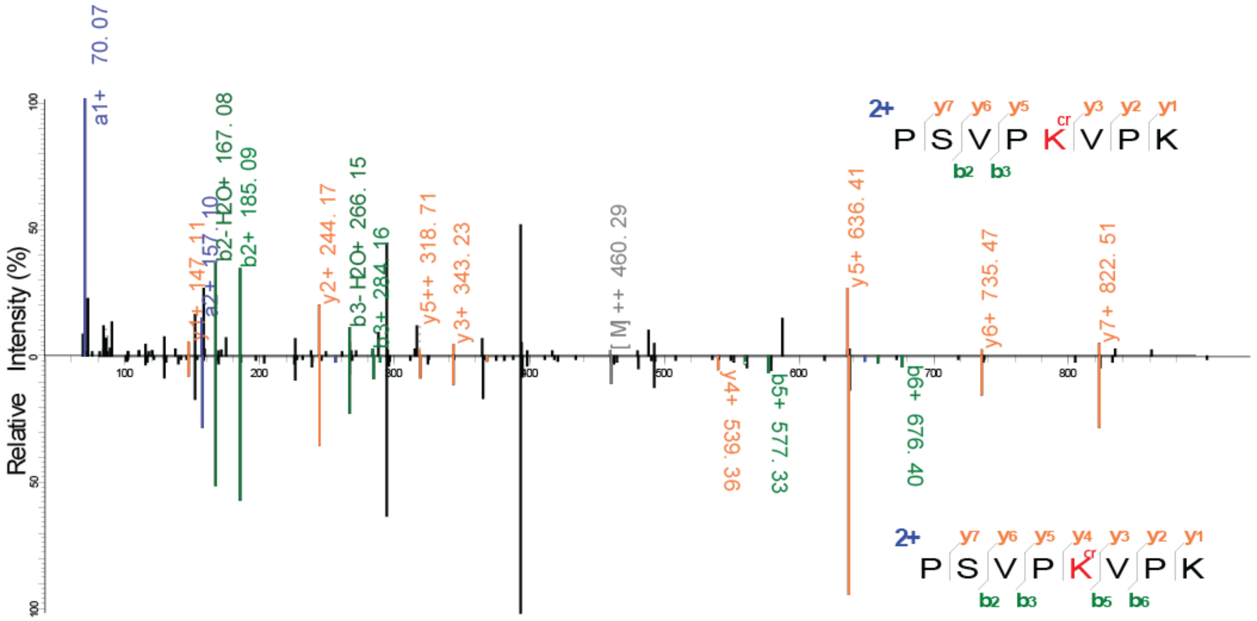
a1+ 70. 07
2+
P S V P K V P K
cr
y7 y6 y5 y3 y2 y1
b2 b3
Relative Intensity (%)
y1+ 147. 11
a2+ 157. 10
b2- H2O+ 167. 08
b2+ 185. 09
y2+ 244. 17
b3- H2O+ 266. 15
b3+ 284. 16
y5++ 318. 71
y3+ 343. 23
[M]++ 460. 29
y5+ 636. 41
y6+ 735. 47
y7+ 822. 51
y4+ 539. 36
b5+ 577. 33
b6+ 676. 40
2+
P S V P K V P K
y7 y6 y5 y4 y3 y2 y1
b2 b3 b5 b6

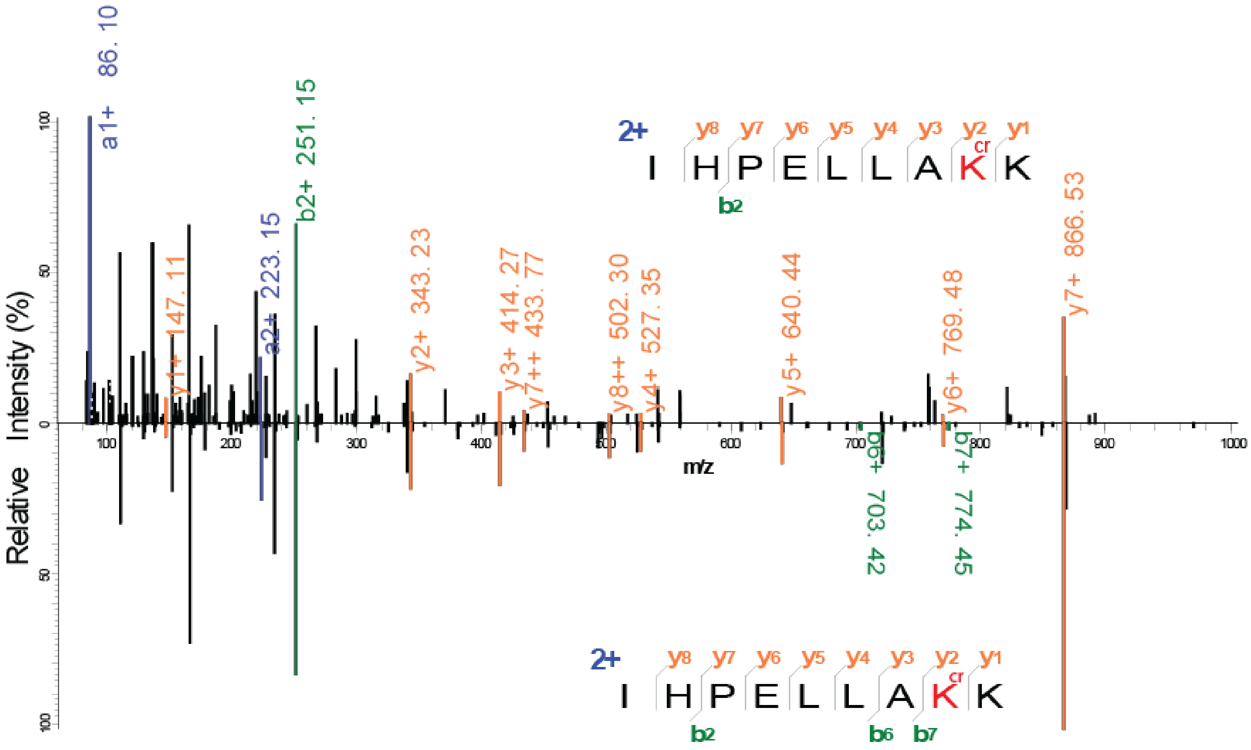

a1+ 86. 10
b2+ 251. 15
a2+ 223. 15
y1+ 147. 11
y2+ 343. 23
y3+ 414. 27
y7++ 433. 77
y8++ 502. 30
y4+ 527. 35
y5+ 640. 44
y6+ 769. 48
y7+ 866. 53
b6+ 703. 42
b7+ 774. 45
2+
y8 y7 y6 y5 y4 y3 y2 y1
I H P E L L A K K
cr
b2
b6 b7
Relative Intensity (%)
m/z
100
50
0
100
200
300
400
500
600
700
800
900
1000

Data S5. PSMs of peptides with co-existing histone marks.

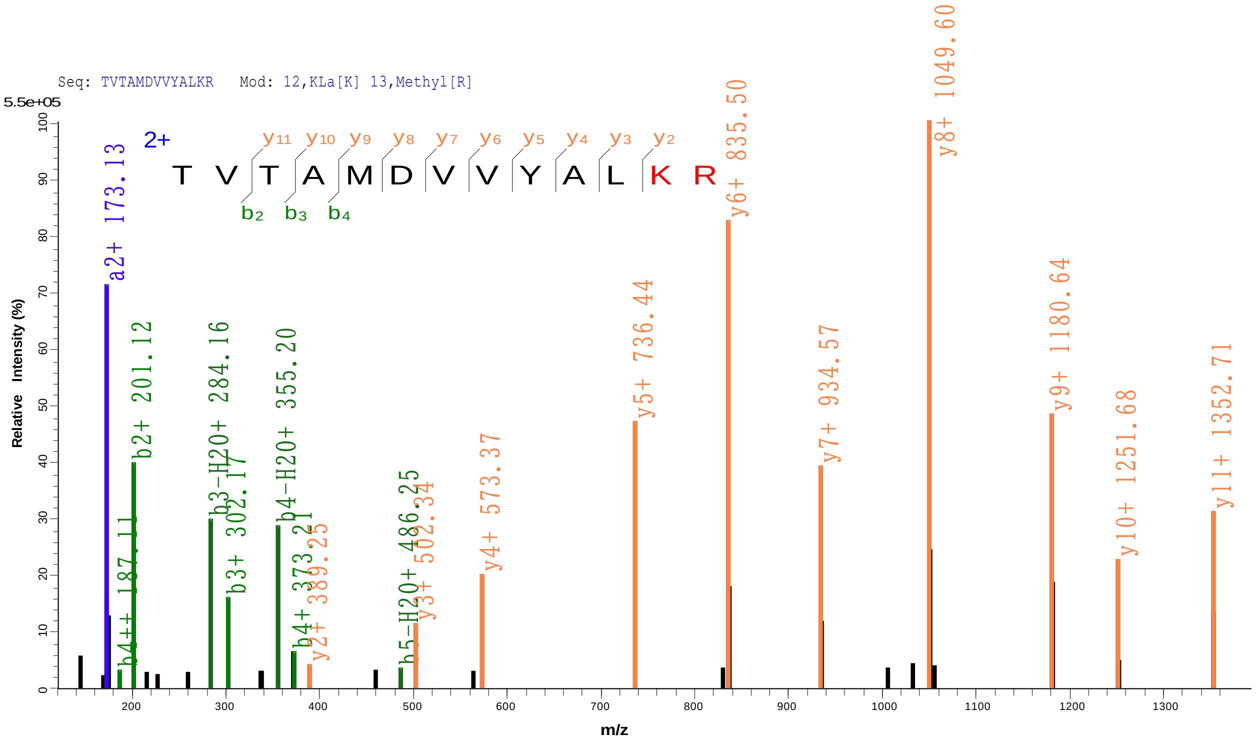

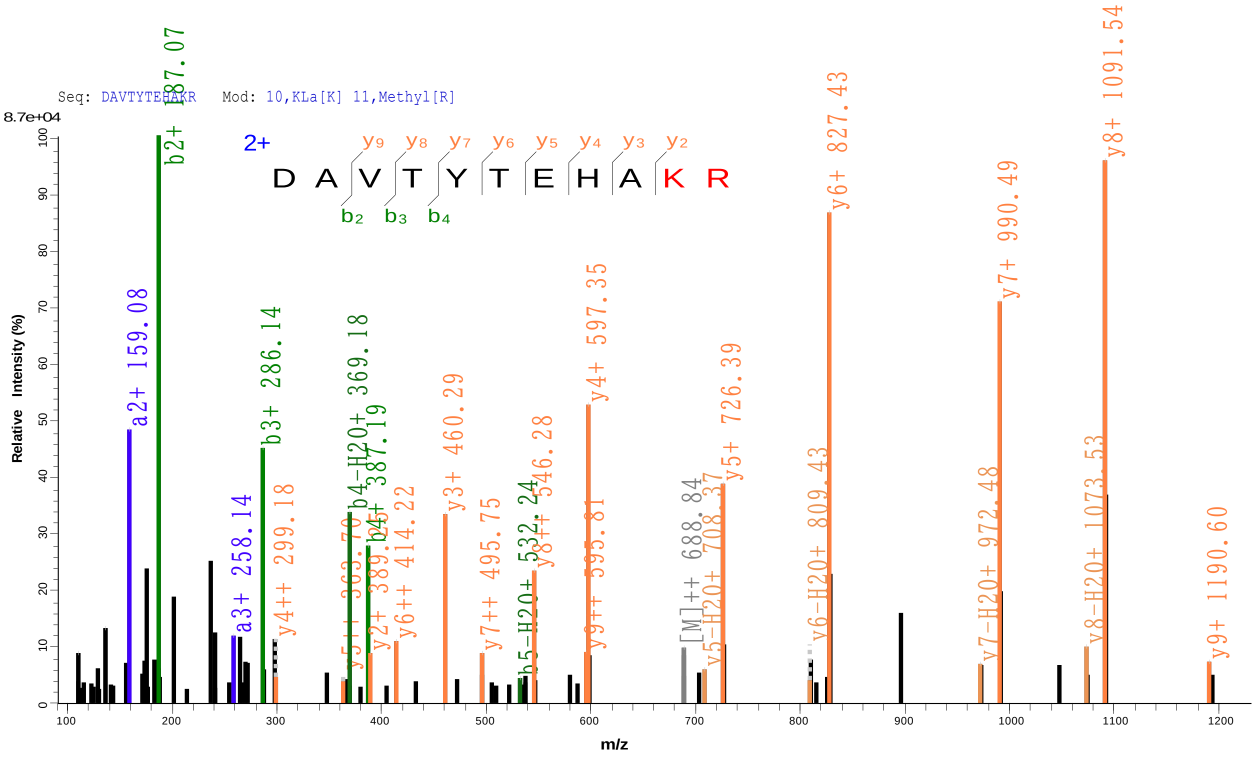

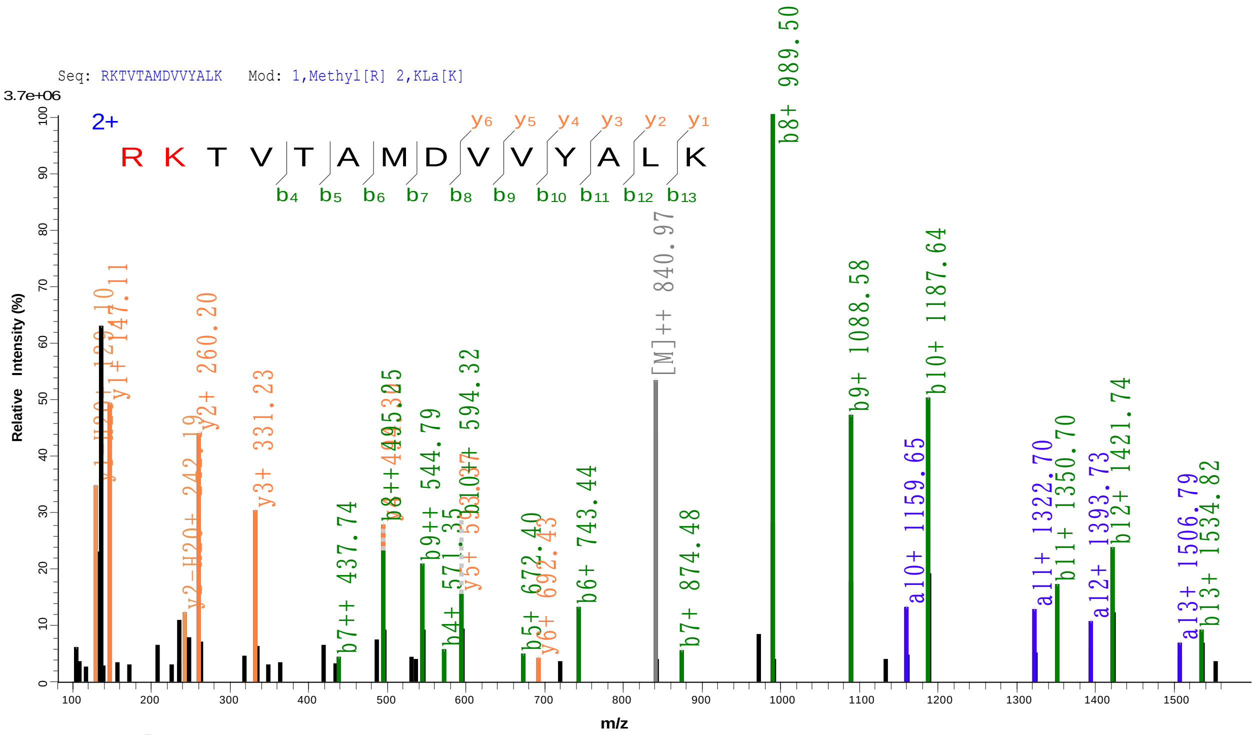

Seq: RKTVTAMDVVYALK Mod: 1,Methyl[R] 2,KLa[K]
3.7e+06
2+
R K T V T A M D V V Y A L K
y6 y5 y4 y3 y2 y1
b4 b5 b6 b7 b8 b9 b10 b11 b12 b13
Relative Intensity (%)
m/z
b8+ 989.50
[M]++ 840.97
b9+ 1088.58
b10+ 1187.64
a10+ 1159.65
a11+ 1322.70
b11+ 1350.70
a12+ 1393.73
b12+ 1421.74
a13+ 1506.79
b13+ 1534.82
y2+ 260.20
y3+ 331.23
b7++ 437.74
b9++ 544.79
b10++ 594.32
b6+ 743.44
b7+ 874.48
b5+ 672.40
y6+ 692.43

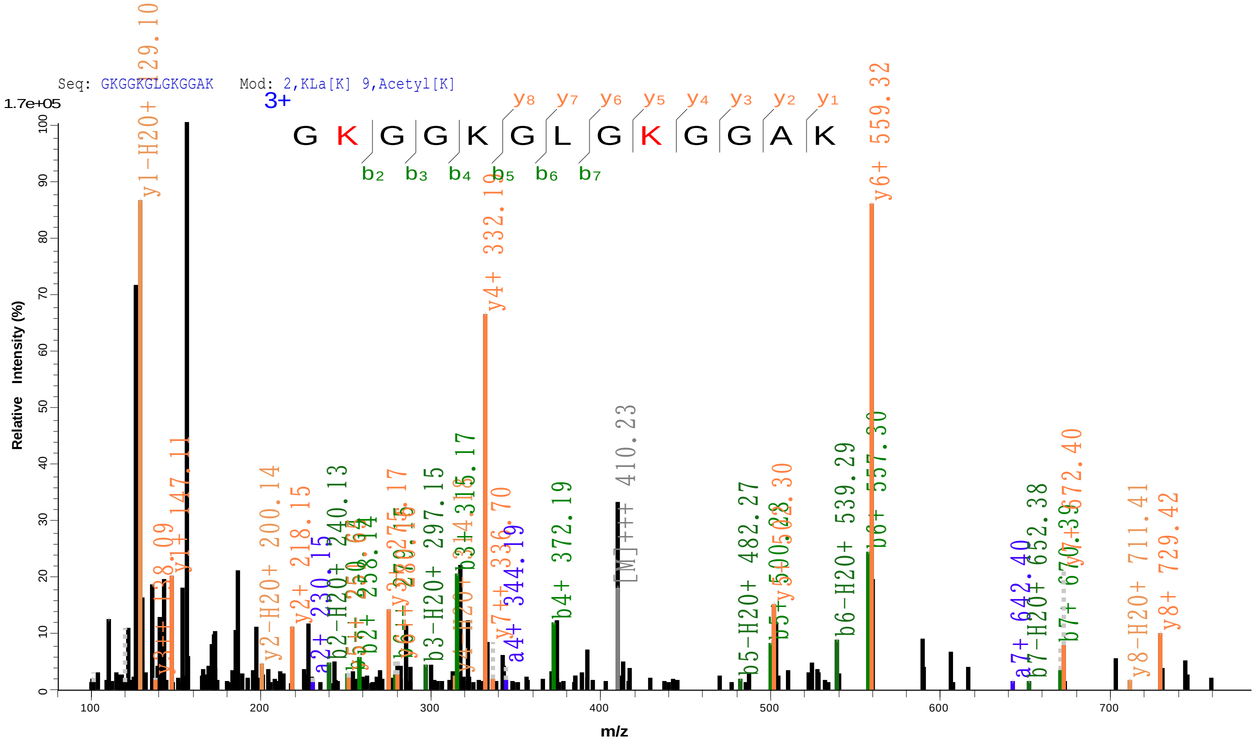

Seq: GKGGKGLGKGGAK Mod: 2,KLa[K] 9,Acetyl[K]
1.7e+05
3+
G K G G K G L G K G G A K
y8 y7 y6 y5 y4 y3 y2 y1
b2 b3 b4 b5 b6 b7
Relative Intensity (%)
m/z
y1-H2O+ 129.10
y1+ 147.11
y2-H2O+ 200.14
y2+ 218.15
y4+ 332.19
y6+ 559.32
[M]+++ 410.23
b4+ 372.19
b3-H2O+ 297.15
b5-H2O+ 482.27
b6-H2O+ 539.29
b6+ 557.30
a7+ 642.40
b7-H2O+ 652.38
y7+ 672.40
y8-H2O+ 711.41
y8+ 729.42
a4+ 344.19

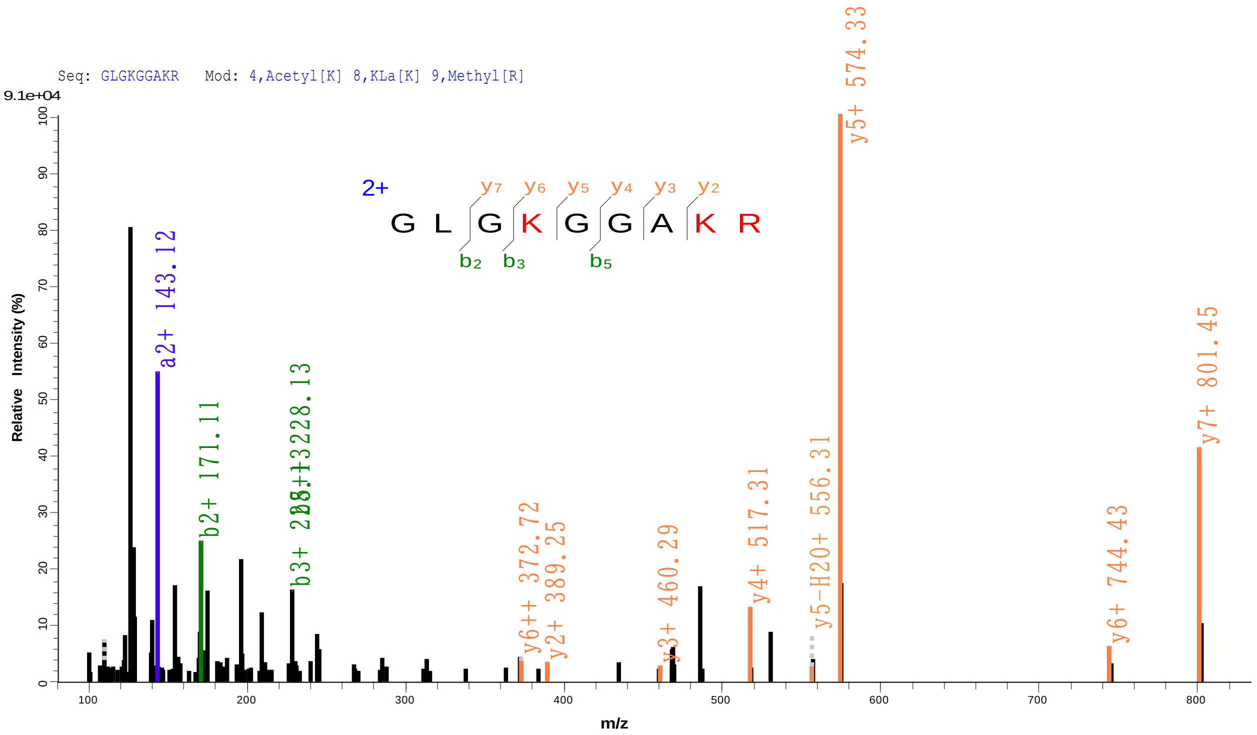
Seq: GLGKGGAKR Mod: 4,Acetyl[K] 8,KLa[K] 9,Methyl[R]
9.1e+04
Relative Intensity (%)
m/z
2+
G L G K G G A K R
y7 y6 y5 y4 y3 y2
b2 b3 b5
a2+ 143.12
b2+ 171.11
b3+ 228.13
y6++ 372.72
y2+ 389.25
y3+ 460.29
y4+ 517.31
y5-H2O+ 556.31
y5+ 574.33
y6+ 744.43
y7+ 801.45

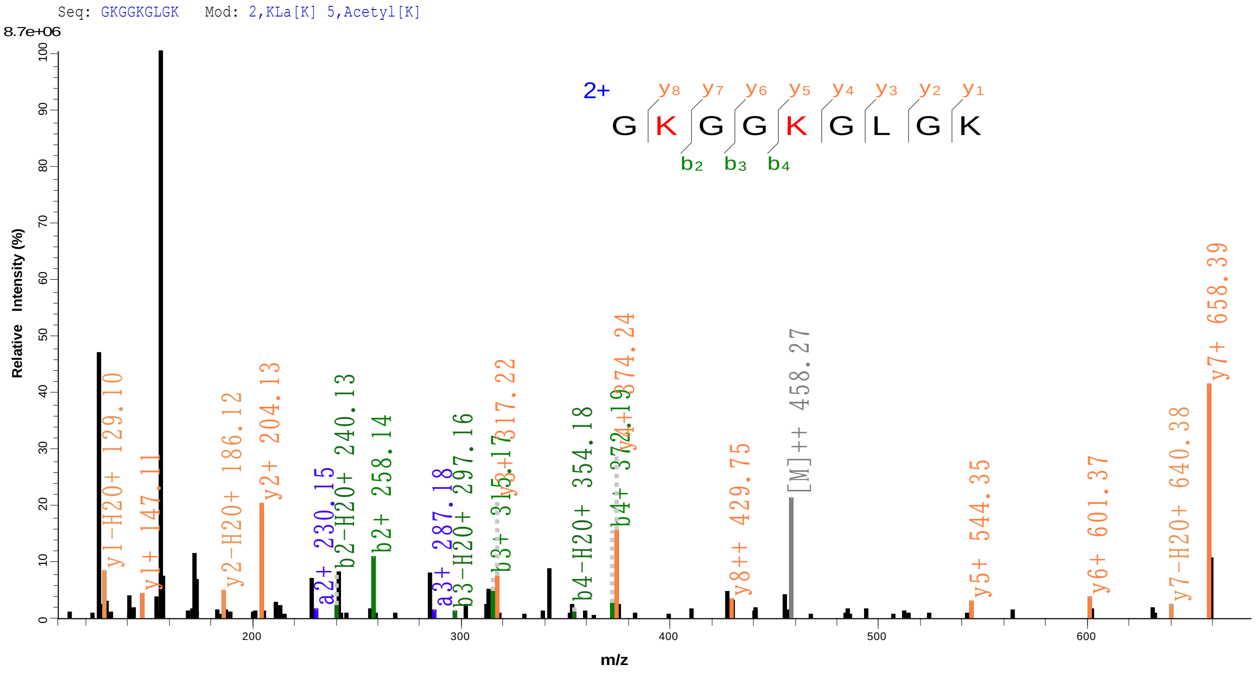
Seq: GKGGKGLGK Mod: 2,KLa[K] 5,Acetyl[K]
8.7e+06
Relative Intensity (%)
m/z
2+
G K G G K G L G K
y8 y7 y6 y5 y4 y3 y2 y1
b2 b3 b4
y1-H2O+ 129.10
y1+ 147.11
y2-H2O+ 186.12
y2+ 204.13
a2+ 230.15
b2-H2O+ 240.13
b2+ 258.14
a3+ 287.18
b3-H2O+ 297.16
b3+ 315.17
y3+ 317.22
b4-H2O+ 354.18
b4+ 372.19
y4+ 374.24
y8++ 429.75
[M]++ 458.27
y5+ 544.35
y6+ 601.37
y7-H2O+ 640.38
y7+ 658.39

Seq: GKGGKGLGK Mod: 2,Acetyl[K] 5,KLa[K]

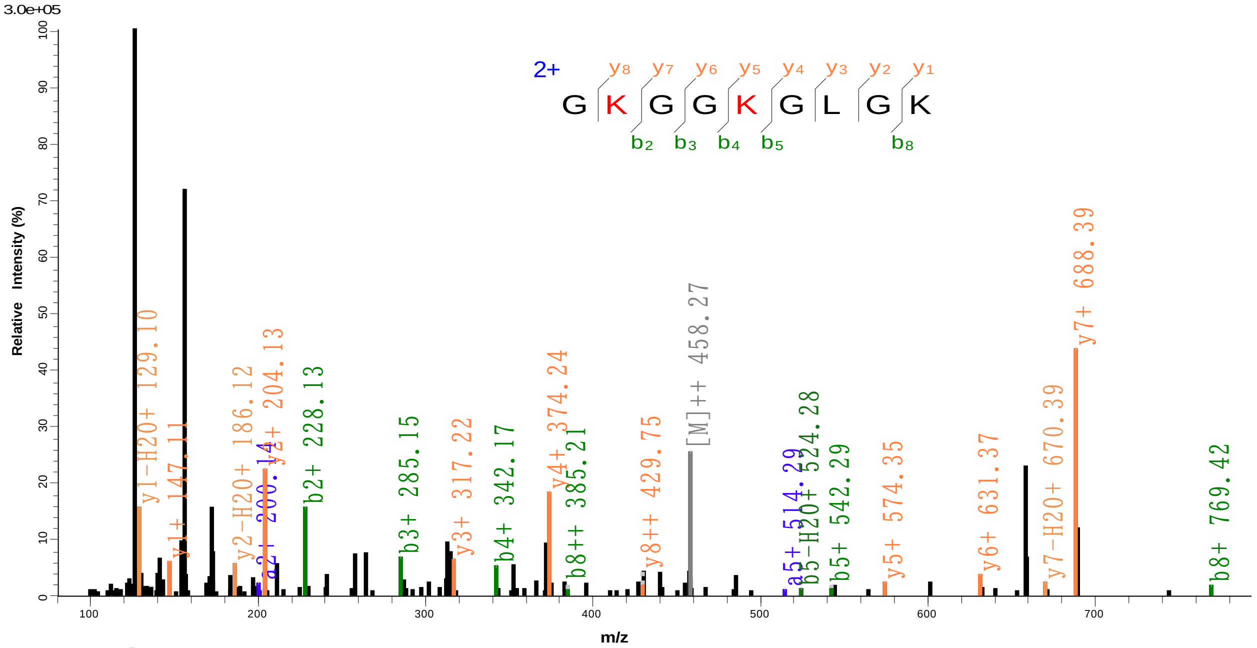

Seq: GKGGKGLGKGGAK Mod: 5,KLa[K] 9,Acetyl[K]

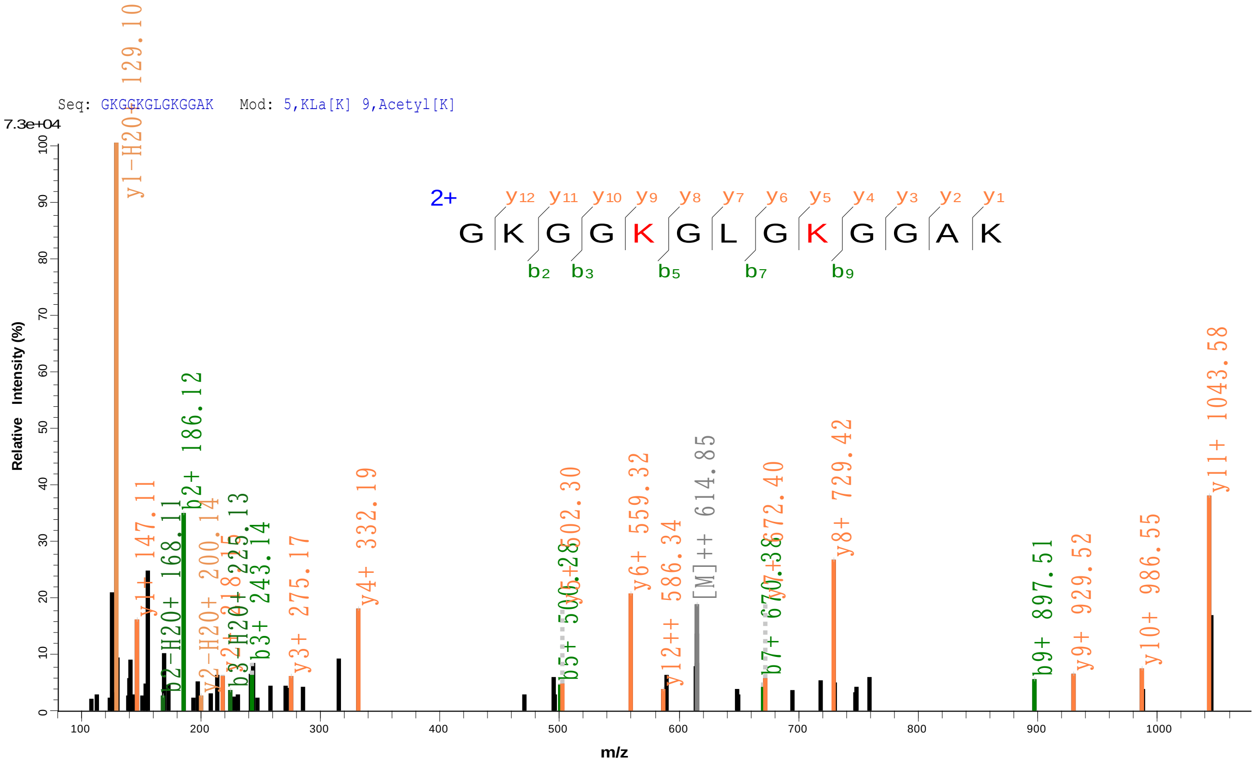

Seq: KSAPATGGVK Mod: 1,KLa[K] 10,Methyl[K]

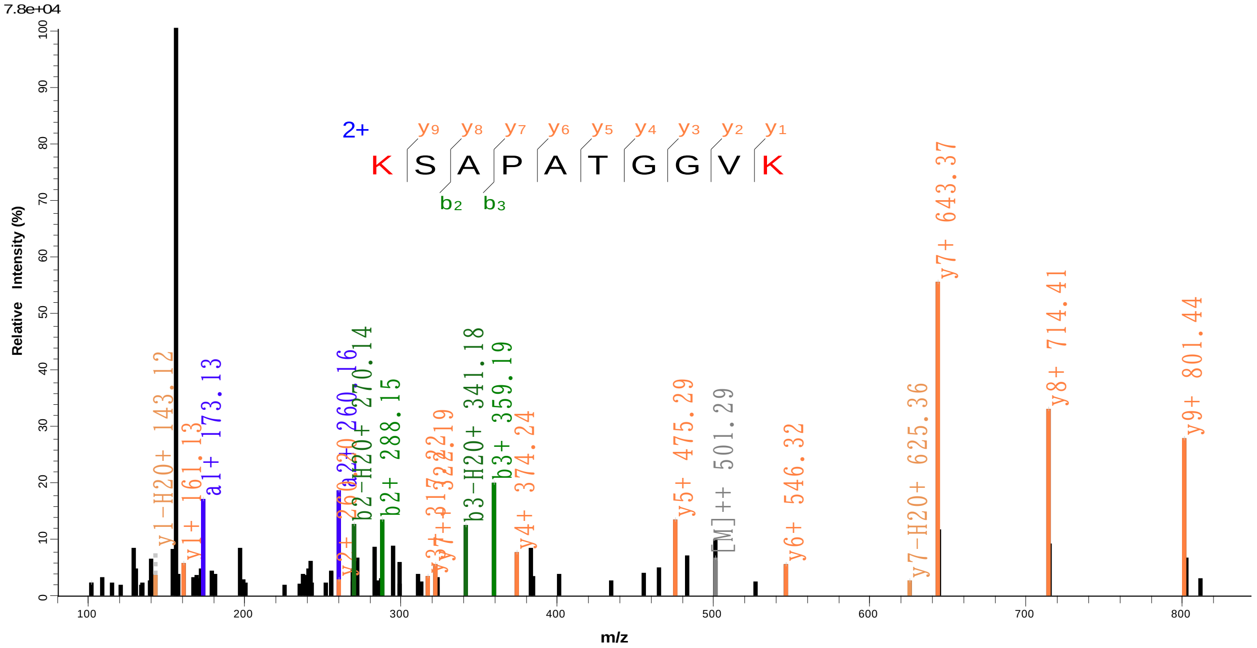

Seq: KQLATKAAR Mod: 1,KLa[K] 6,Acetyl[K]

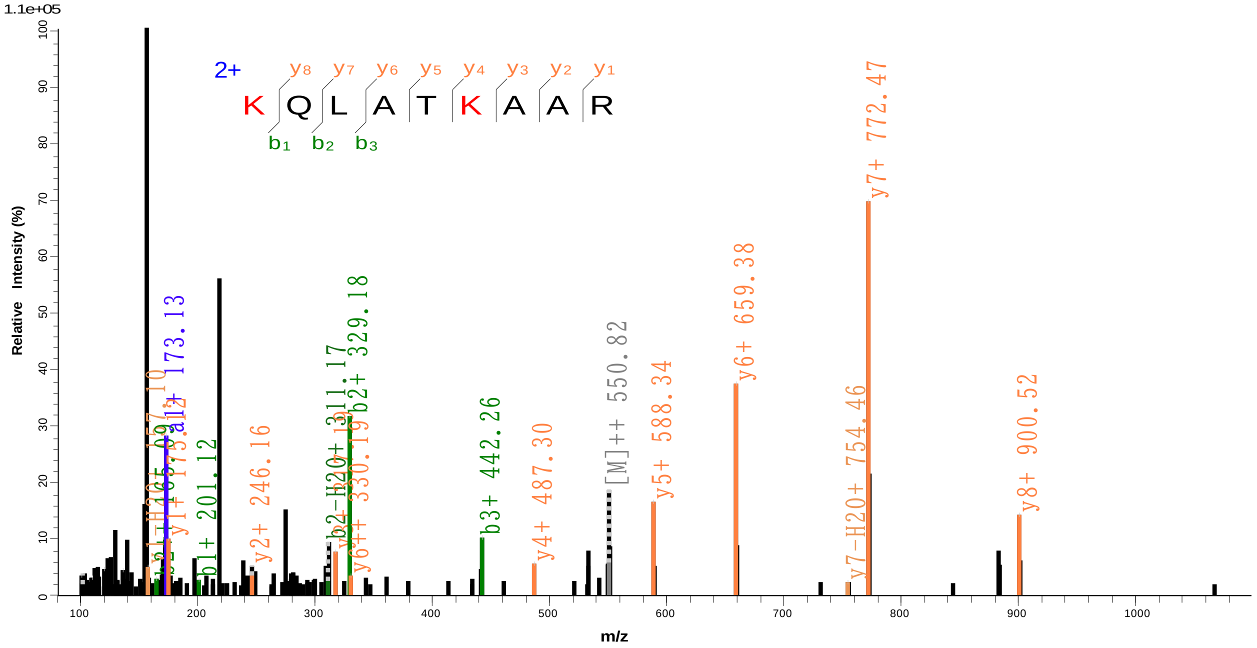

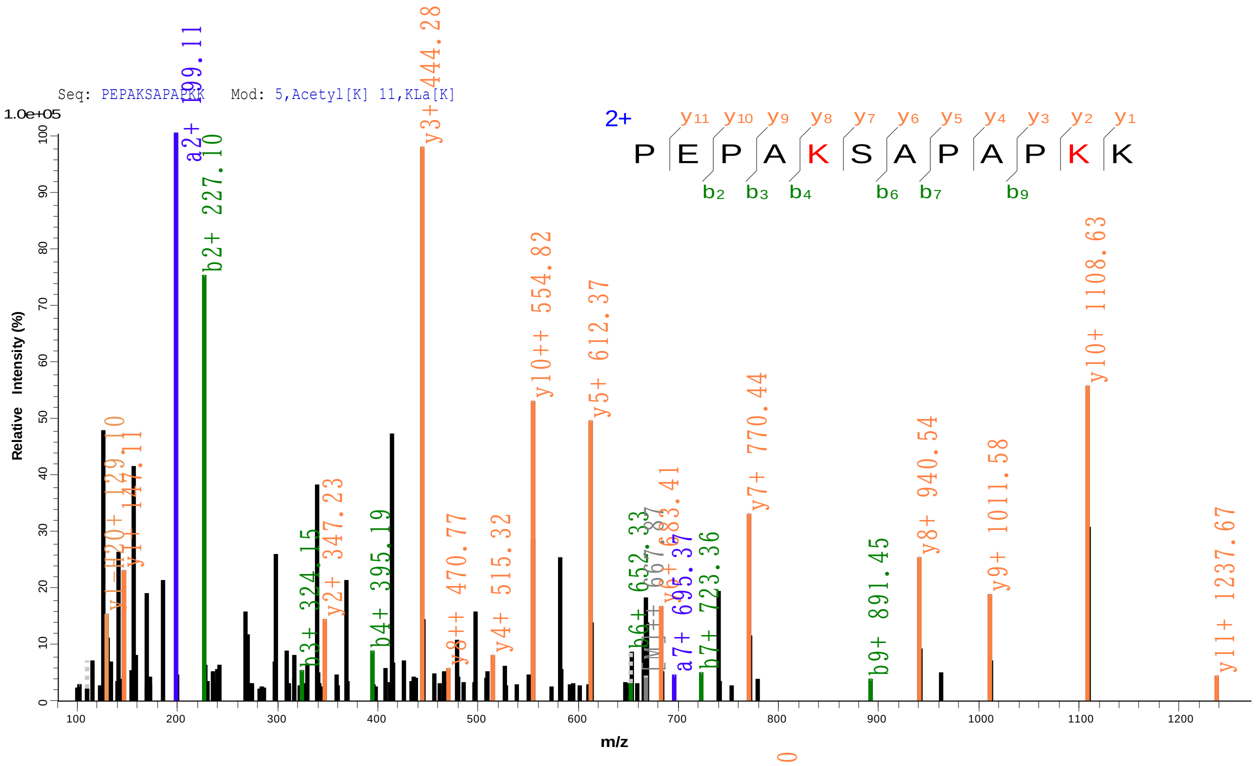

Seq: PEPAKSAPAPKK Mod: 5,Acetyl[K] 11,KLa[K]
1.0e+05
Relative Intensity (%)
m/z
2+
P E P A K S A P A P K K
y11 y10 y9 y8 y7 y6 y5 y4 y3 y2 y1
b2 b3 b4 b6 b7 b9
a2+ 199.11
b2+ 227.10
y3+ 444.28
y10++ 554.82
y5+ 612.37
y10+ 1108.63
y7+ 770.44
y8+ 940.54
y9+ 1011.58
y11+ 1237.67
b9+ 891.45
b7+ 723.36
a7+ 695.37
b4+ 395.19
b3+ 324.15
y2+ 347.23
y8++ 470.77
y4+ 515.32
b6+ 652.33

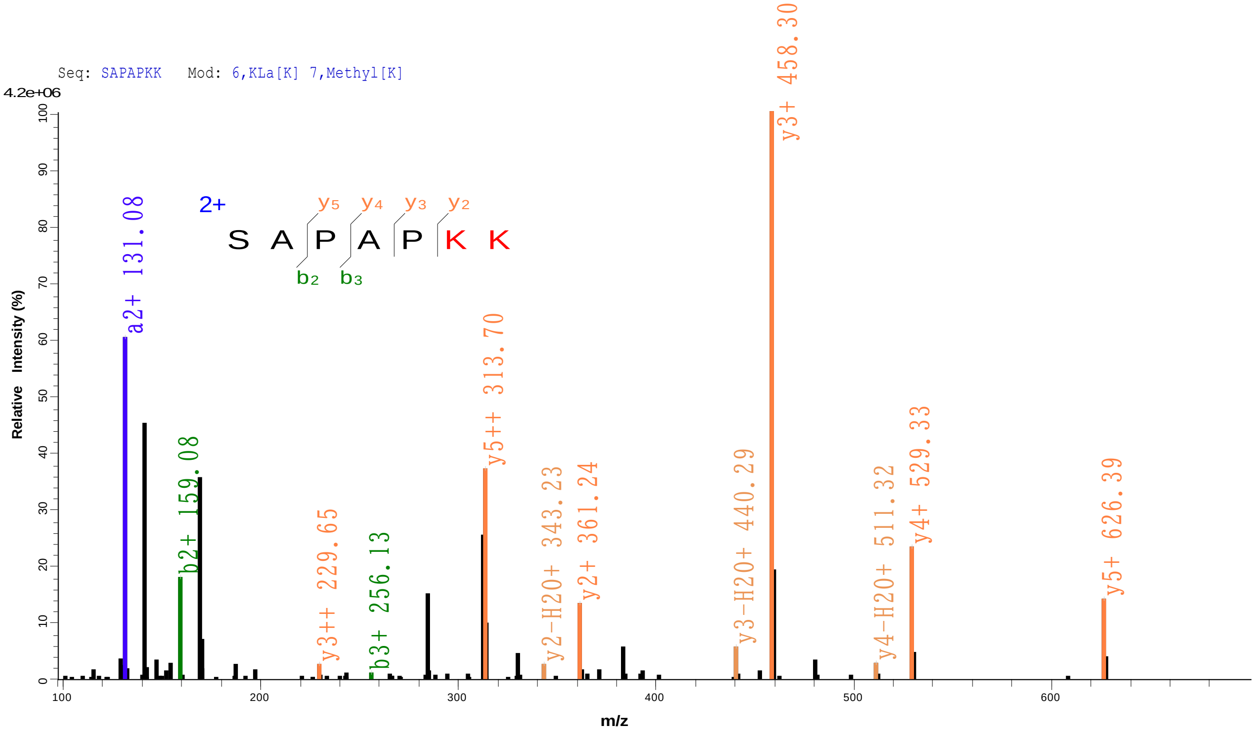

Seq: SAPAPKK Mod: 6,KLa[K] 7,Methyl[K]
4.2e+06
Relative Intensity (%)
m/z
2+
S A P A P K K
y5 y4 y3 y2
b2 b3
a2+ 131.08
b2+ 159.08
y3++ 229.65
b3+ 256.13
y5++ 313.70
y2-H2O+ 343.23
y2+ 361.24
y3-H2O+ 440.29
y3+ 458.30
y4-H2O+ 511.32
y4+ 529.33
y5+ 626.39

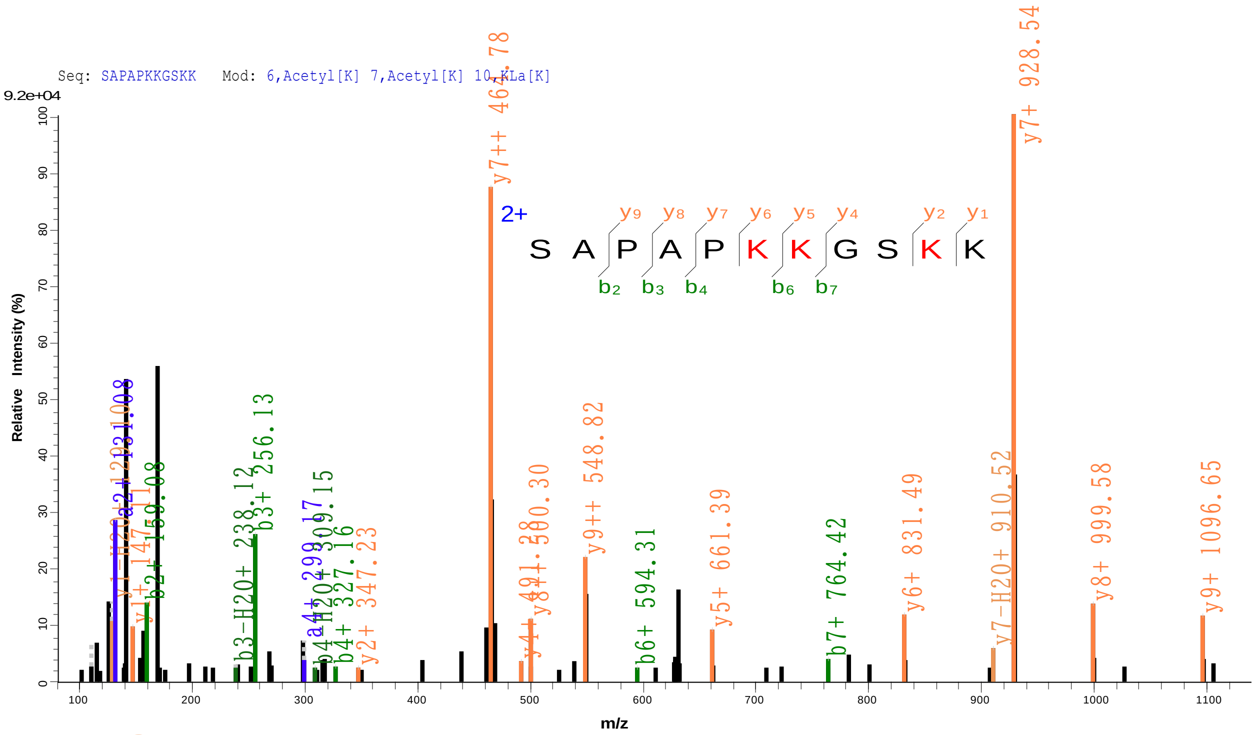

Seq: SAPAPKKGSKK Mod: 6,Acetyl[K] 7,Acetyl[K] 10,KLa[K]
9.2e+04
Relative Intensity (%)
m/z
2+
S A P A P K K G S K K
y7+ 928.54
y9++ 548.82
b3+ 256.13
b7+ 764.42
b6+ 594.31
y5+ 661.39
y6+ 831.49
y7-H2O+ 910.52
y8+ 999.58
y9+ 1096.65
b3-H2O+ 238.12

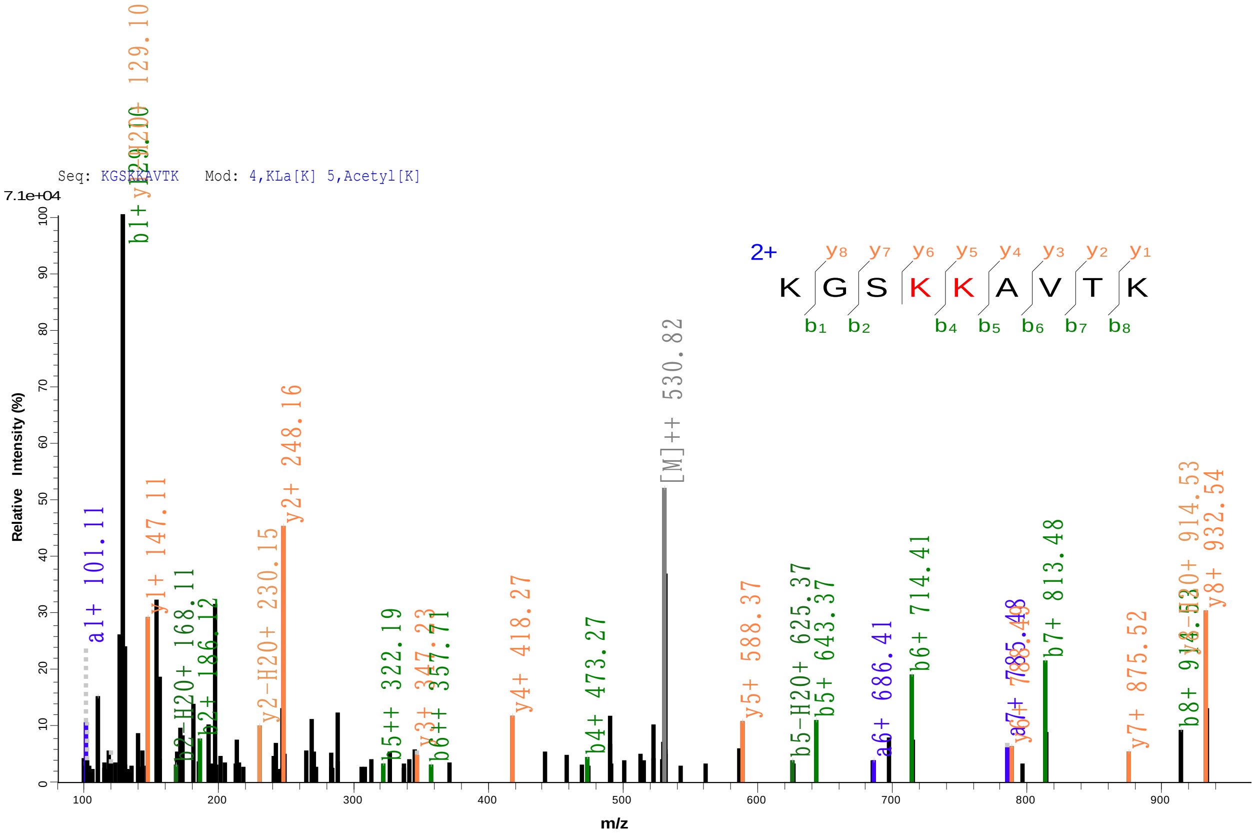

Seq: KGSKKAVTK Mod: 4,KLa[K] 5,Acetyl[K]
7.1e+04
Relative Intensity (%)
m/z
2+
K G S K K A V T K
[M]++ 530.82
a1+ 101.11
y1+ 147.11
y2+ 248.16
y2-H2O+ 230.15
b2-H2O+ 168.11
b5++ 322.19
y4+ 418.27
b4+ 473.27
y5+ 588.37
b5-H2O+ 625.37
b5+ 643.37
a6+ 686.41
b6+ 714.41
b7+ 813.48
y7+ 875.52
y8+ 932.54

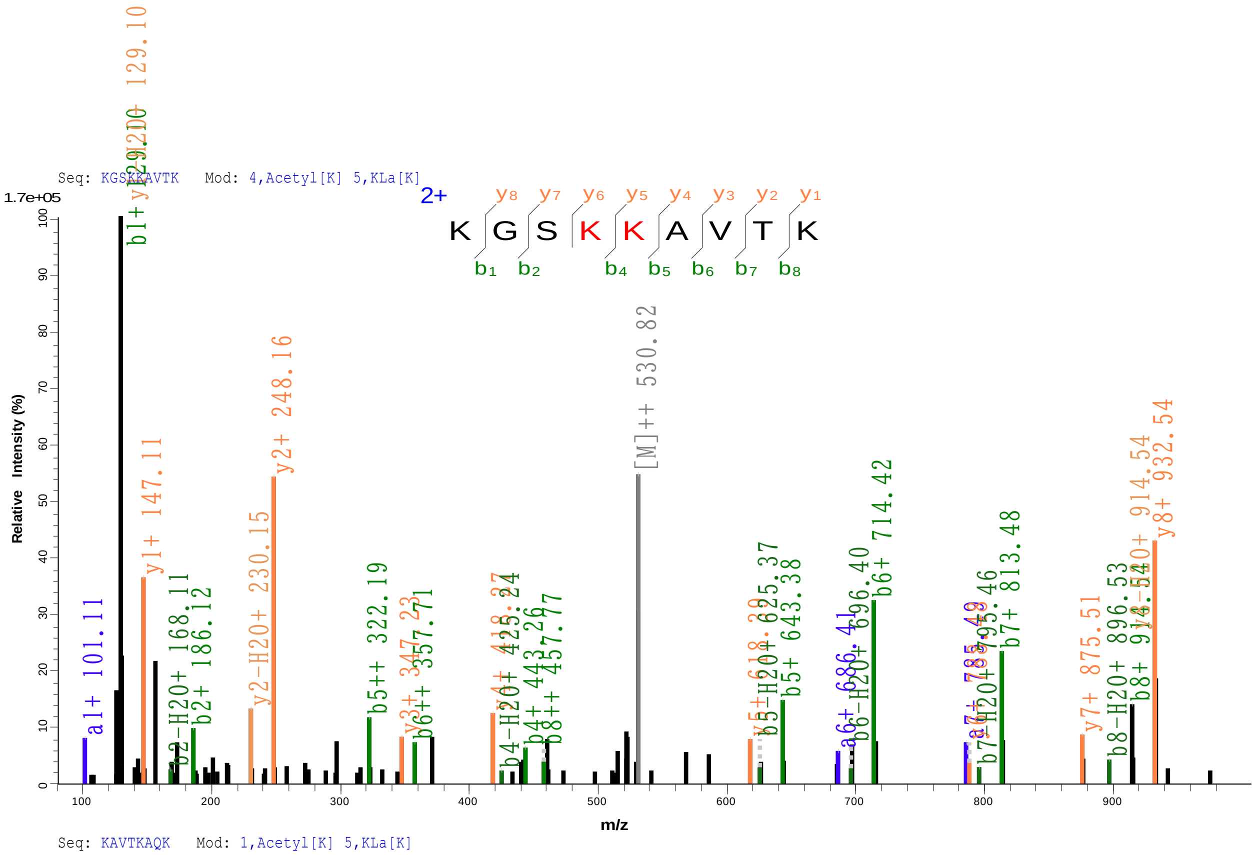
Seq: KGSKKAVTK Mod: 4,Acetyl[K] 5,KLa[K]
1.7e+05
Relative Intensity (%)
m/z
2+
K G S K K A V T K
[M]++ 530.82
y2+ 248.16
y1+ 147.11
y8+ 932.54
b6+ 714.42
b7+ 813.48

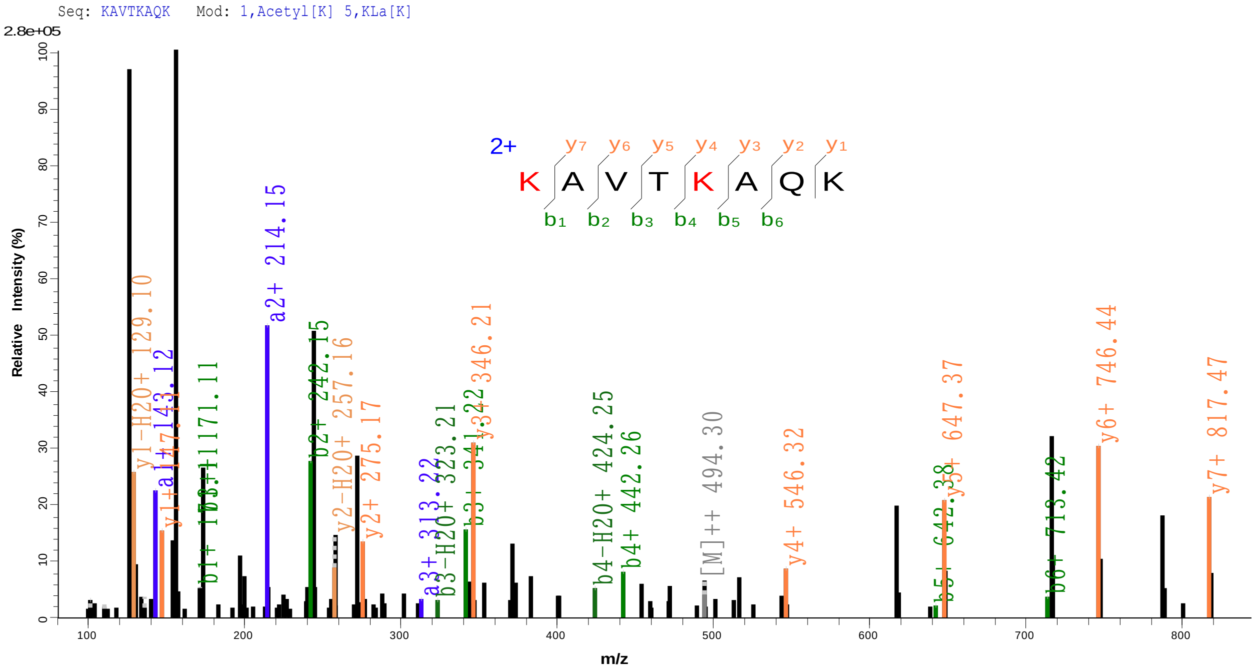
Seq: KAVTKAQK Mod: 1,Acetyl[K] 5,KLa[K]
2.8e+05
Relative Intensity (%)
m/z
2+
K A V T K A Q K
a2+ 214.15
[M]++ 494.30
y4+ 546.32
y6+ 746.44
y7+ 817.47
b4+ 442.26
b4-H2O+ 424.25

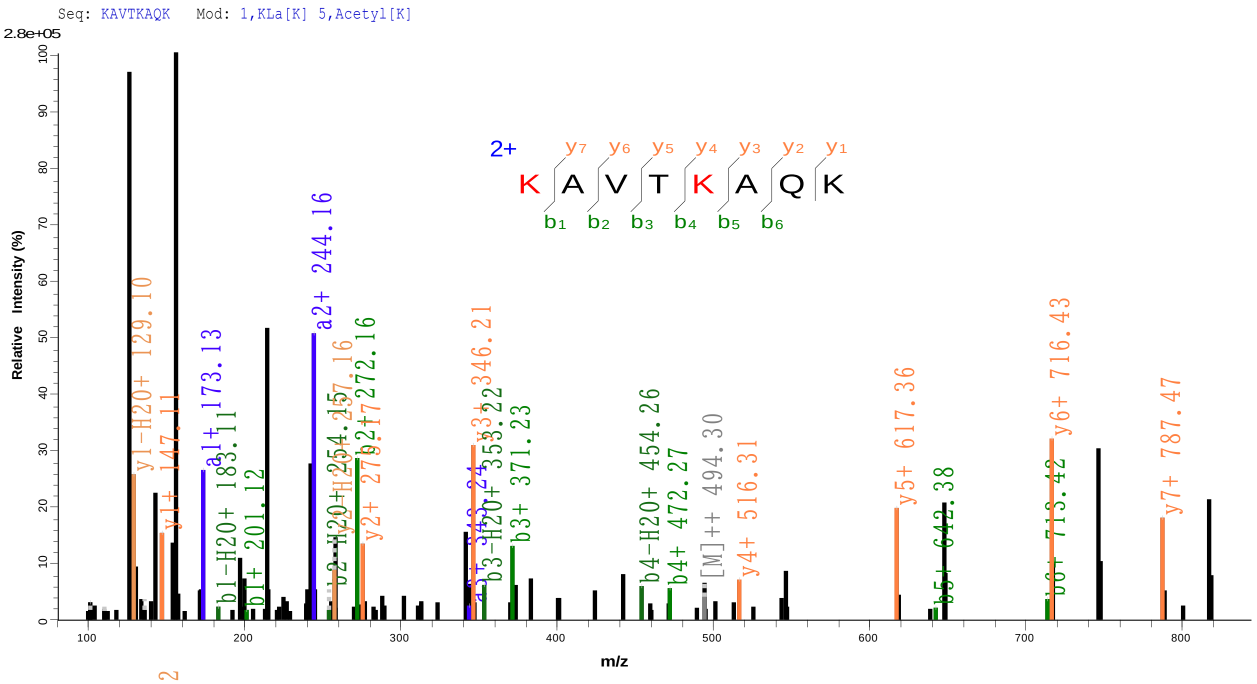
Seq: KAVTKAQK Mod: 1,KLa[K] 5,Acetyl[K]
2.8e+05
Relative Intensity (%)
m/z
2+
K A V T K A Q K

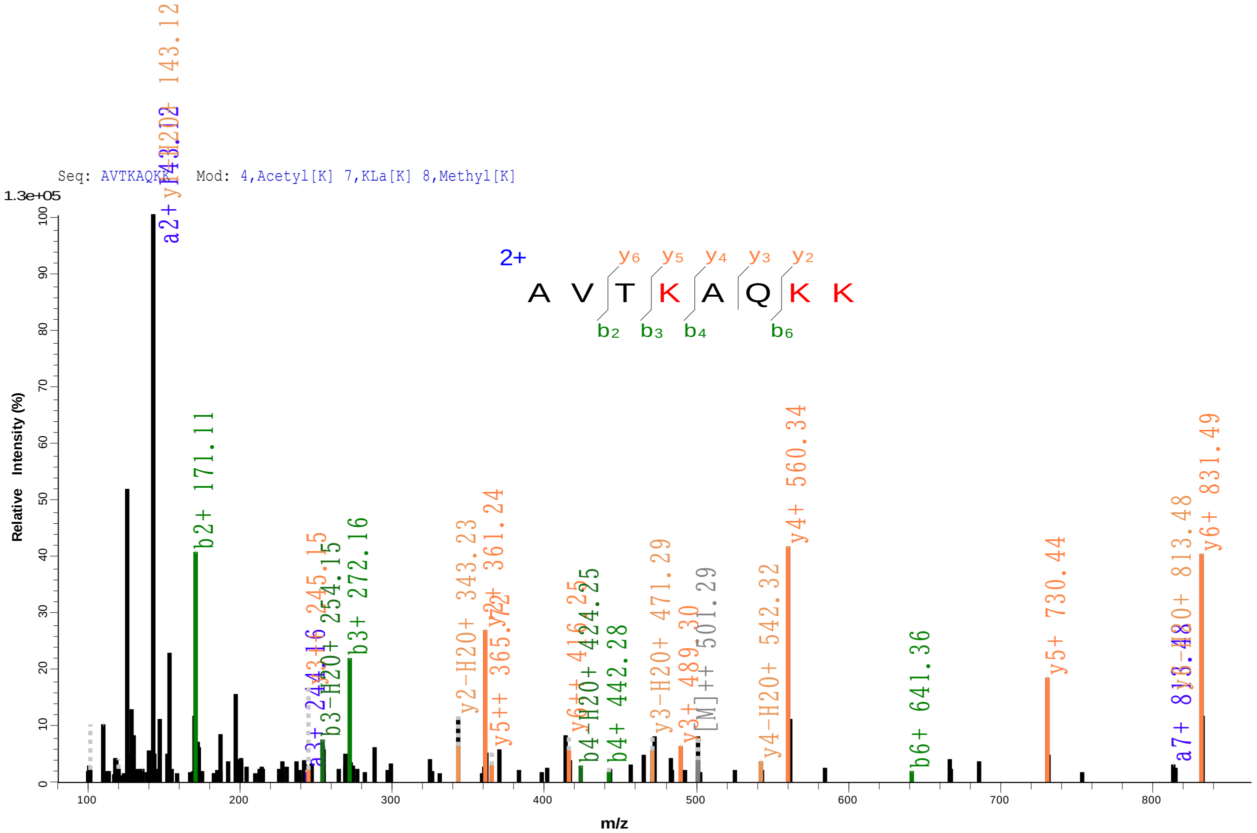
Seq: AVTKAQKK Mod: 4,Acetyl[K] 7,KLa[K] 8,Methyl[K]
1.3e+05
Relative Intensity (%)
m/z
2+
A V T K A Q K K

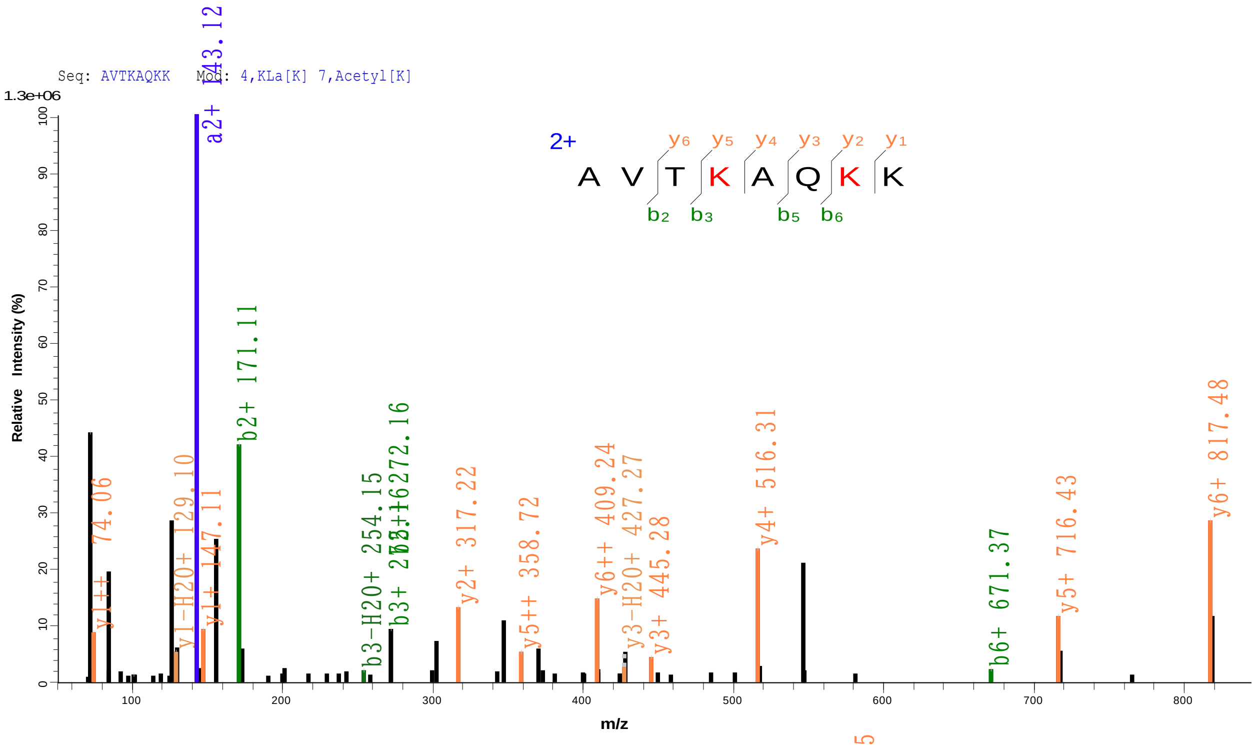

Seq: AVTKAQKK Mod: 4,KLa[K] 7,Acetyl[K]
1.3e+06
Relative Intensity (%)
m/z
2+
A V T K A Q K K
a2+ 143.12
b2+ 171.11
y1++ 74.06
y1-H2O+ 129.10
y1+ 147.11
b3-H2O+ 254.15
b3+ 272.16
y2+ 317.22
y5++ 358.72
y6++ 409.24
y3-H2O+ 427.27
y3+ 445.28
y4+ 516.31
b6+ 671.37
y5+ 716.43
y6+ 817.48

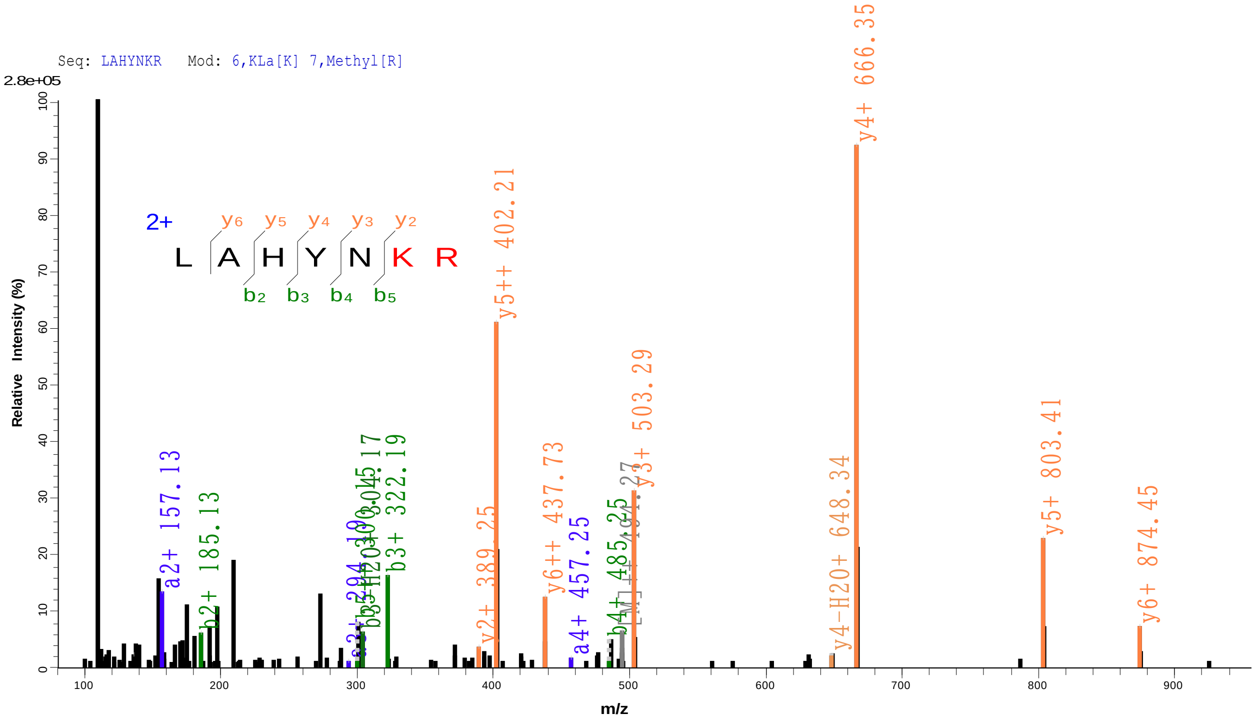

Seq: LAHYNKR Mod: 6,KLa[K] 7,Methyl[R]
2.8e+05
Relative Intensity (%)
m/z
2+
L A H Y N K R
a2+ 157.13
b2+ 185.13
b3+ 322.19
y2+ 389.25
y5++ 402.21
y6++ 437.73
a4+ 457.25
b4+ 485.25
y3+ 503.29
y4-H2O+ 648.34
y4+ 666.35
y5+ 803.41
y6+ 874.45

Seq: ESYSIYVYKVLK Mod: 9,KLa[K] 12,Methyl[K]

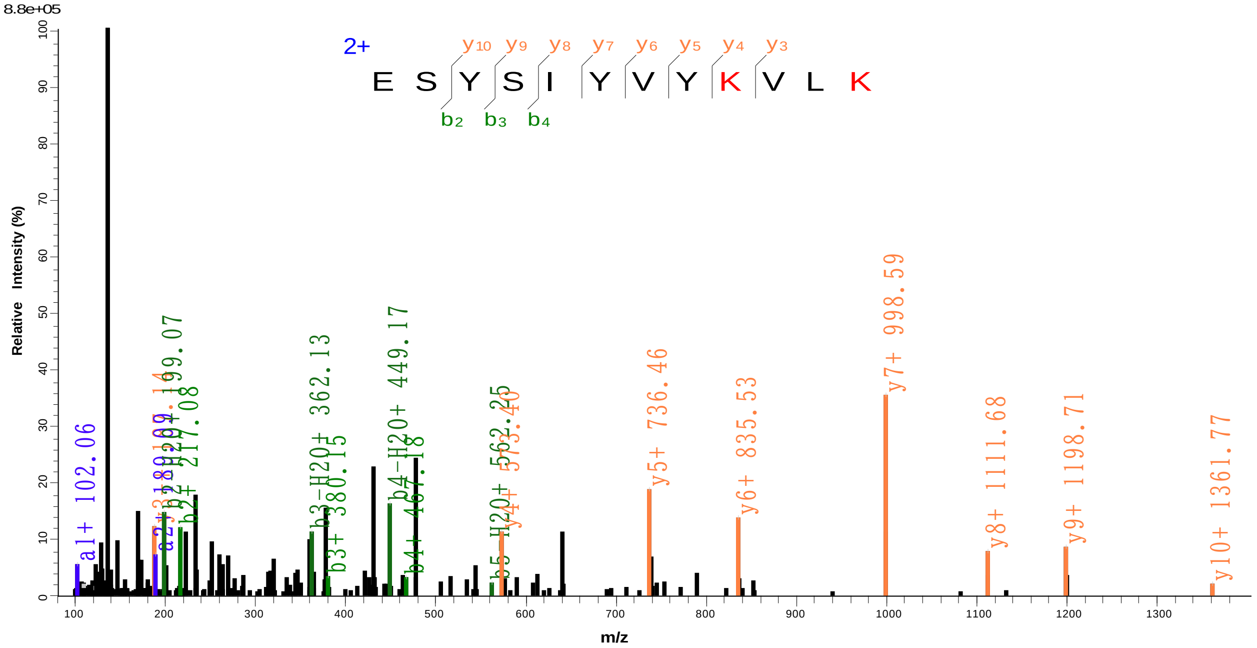

Seq: PDPAKSAPAPKKGSK Mod: 5,KLa[K] 11,Acetyl[K] 12,Acetyl[K]

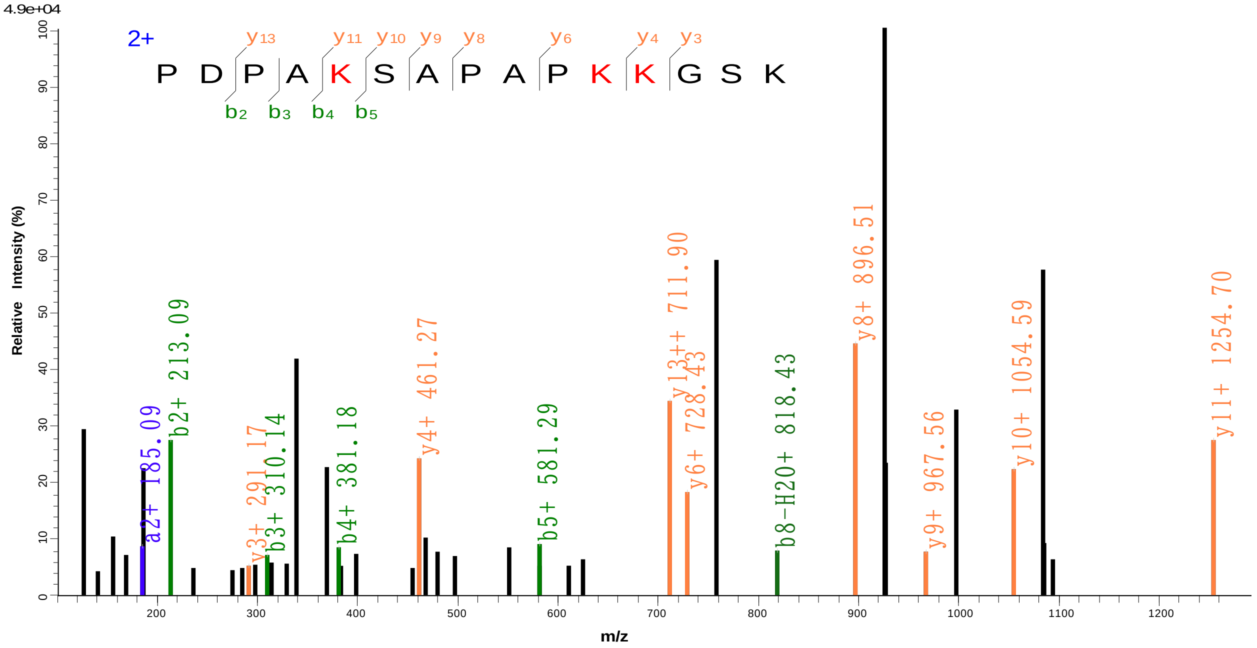

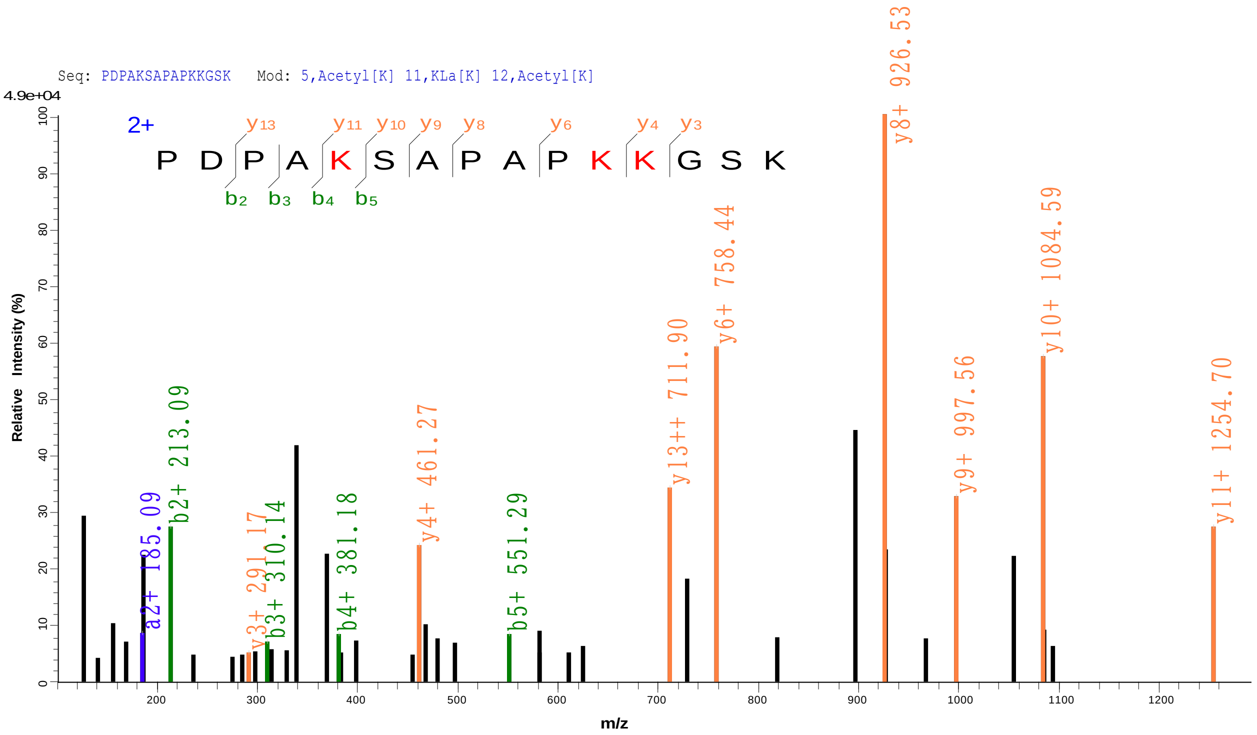

Seq: PDPAKSAPAPKKGSK Mod: 5,Acetyl[K] 11,KLa[K] 12,Acetyl[K]
4.9e+04
2+
P D P A K S A P A P K K G S K
Relative Intensity (%)
m/z
y8+ 926.53
y6+ 758.44
y10+ 1084.59
y13++ 711.90
y9+ 997.56
y11+ 1254.70
y4+ 461.27
b2+ 213.09
a2+ 185.09
b4+ 381.18
b5+ 551.29
y3+ 291.17
b3+ 310.14

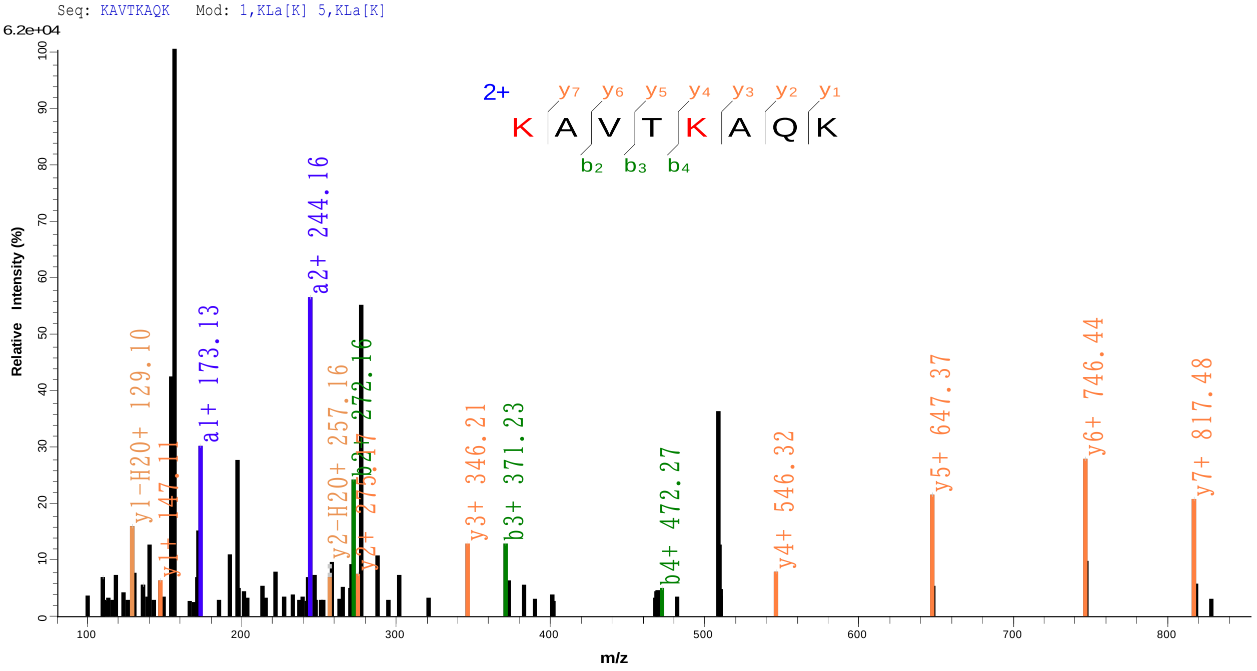

Seq: KAVTKAQK Mod: 1,KLa[K] 5,KLa[K]
6.2e+04
2+
K A V T K A Q K
Relative Intensity (%)
m/z
a2+ 244.16
a1+ 173.13
y1-H2O+ 129.10
y1+ 147.11
y2-H2O+ 257.16
b2+ 272.16
y2+ 275.17
y3+ 346.21
b3+ 371.23
b4+ 472.27
y4+ 546.32
y5+ 647.37
y6+ 746.44
y7+ 817.48

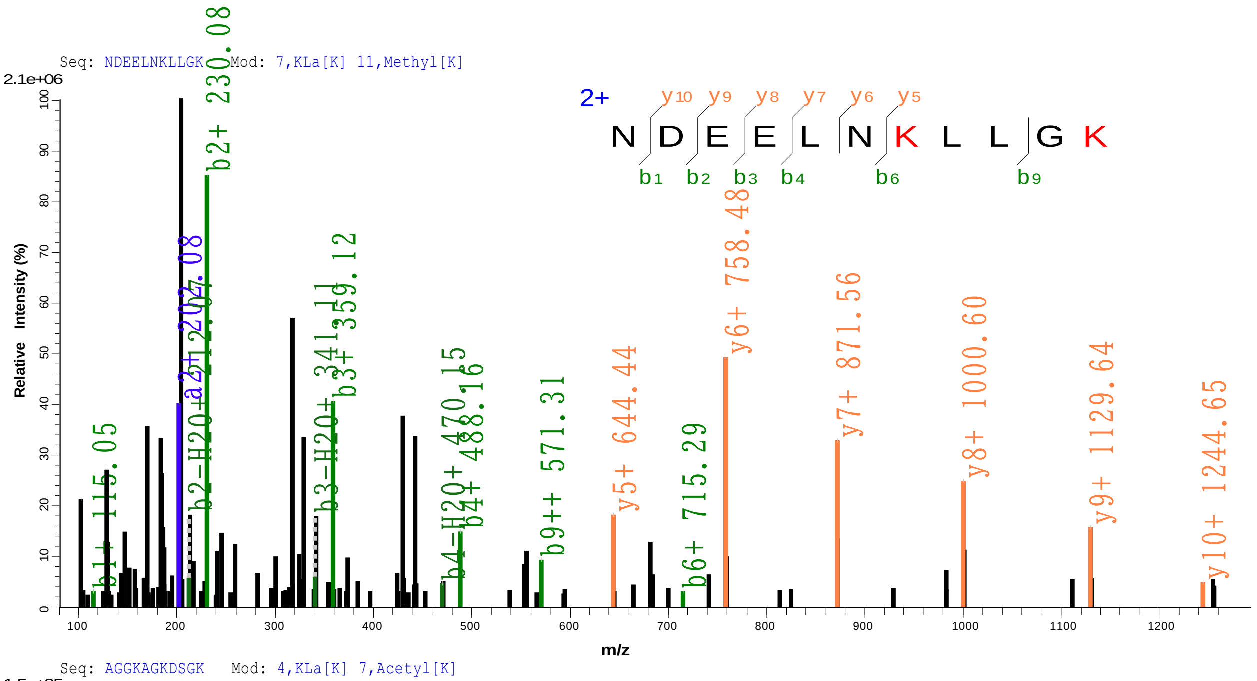
Seq: NDEELNKLLGK Mod: 7,KLa[K] 11,Methyl[K]
2.1e+06
Relative Intensity (%)
m/z
2+
N D E E L N K L L G K
y10 y9 y8 y7 y6 y5
b1 b2 b3 b4 b6 b9
b2+ 230.08
b1+ 115.05
b3+ 359.12
b4+ 488.16
b9++ 571.31
y5+ 644.44
b6+ 715.29
y6+ 758.48
y7+ 871.56
y8+ 1000.60
y9+ 1129.64
y10+ 1244.65

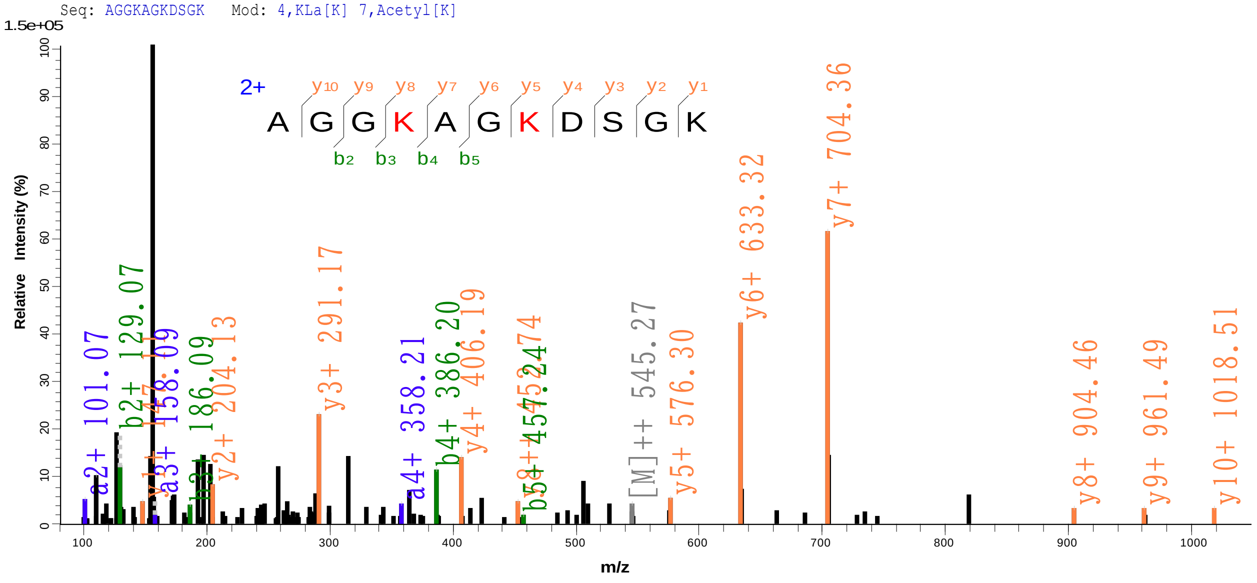
Seq: AGGKAGKDSGK Mod: 4,KLa[K] 7,Acetyl[K]
1.5e+05
Relative Intensity (%)
m/z
2+
A G G K A G K D S G K
y10 y9 y8 y7 y6 y5 y4 y3 y2 y1
b2 b3 b4 b5
a2+ 101.07
b2+ 129.07
a3+ 158.09
y2+ 204.13
y3+ 291.17
a4+ 358.21
b4+ 386.20
y4+ 406.19
[M]++ 545.27
y5+ 576.30
y6+ 633.32
y7+ 704.36
y8+ 904.46
y9+ 961.49
y10+ 1018.51

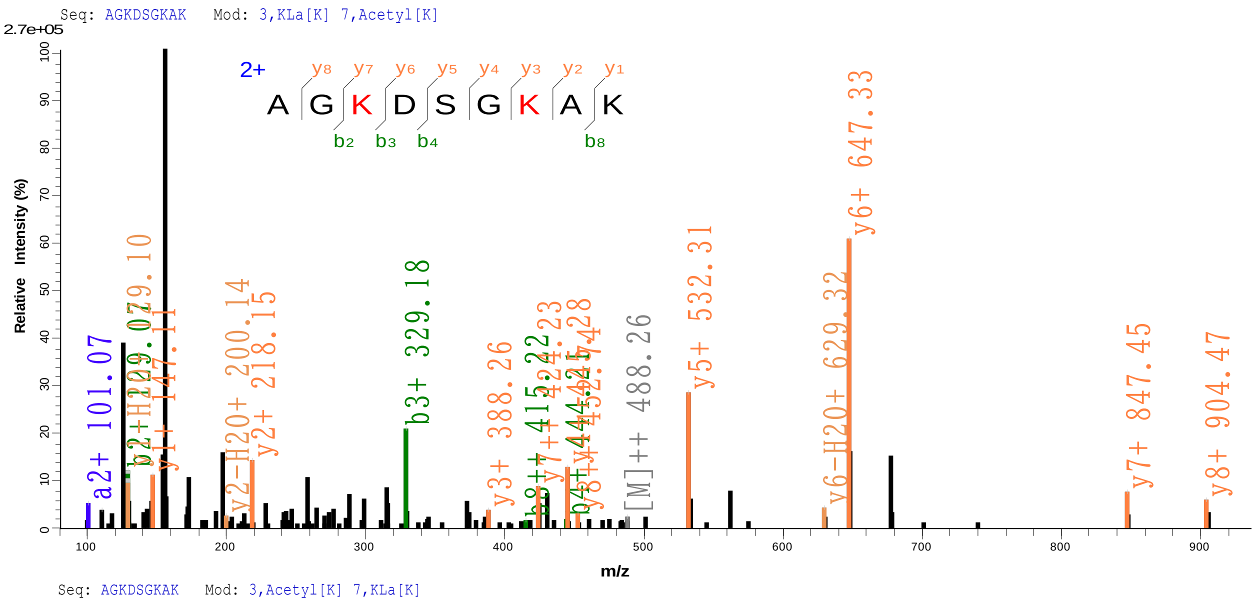
Seq: AGKDSGKAK Mod: 3,KLa[K] 7,Acetyl[K]
2.7e+05
2+
A G K D S G K A K
y8 y7 y6 y5 y4 y3 y2 y1
b2 b3 b4 b8
Relative Intensity (%)
m/z
a2+ 101.07
b3+ 329.18
y3+ 388.26
[M]++ 488.26
y5+ 532.31
y6-H2O+ 629.32
y6+ 647.33
y7+ 847.45
y8+ 904.47

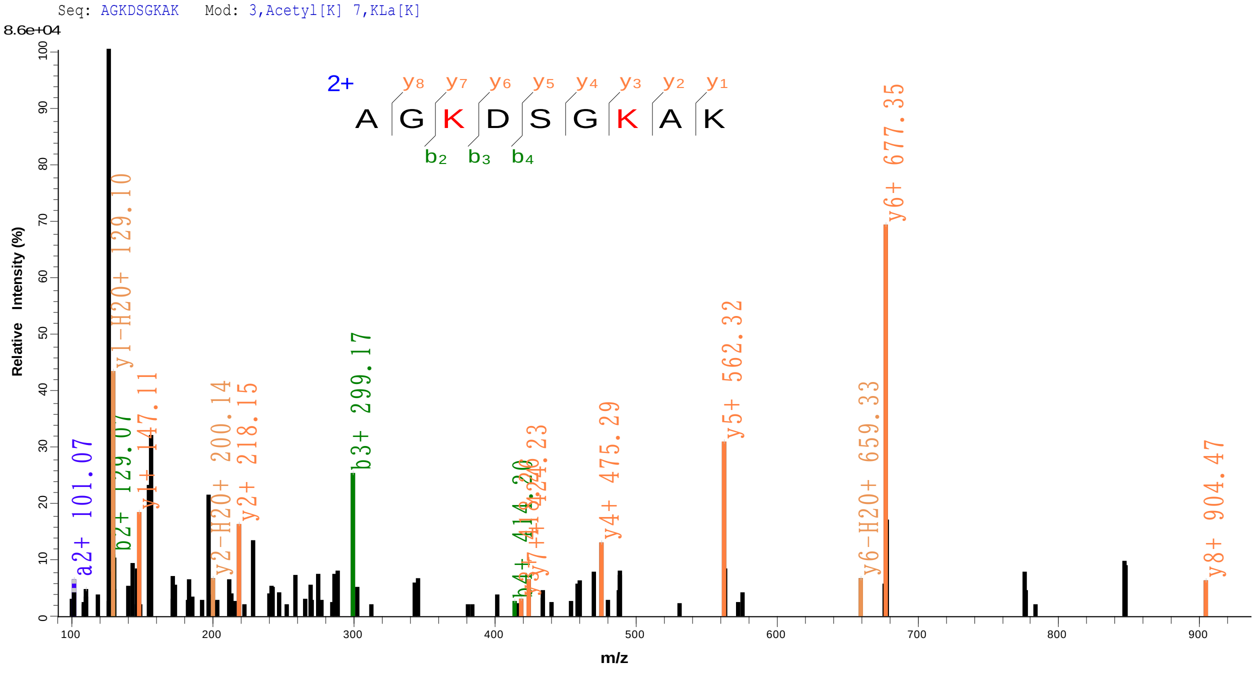
Seq: AGKDSGKAK Mod: 3,Acetyl[K] 7,KLa[K]
8.6e+04
2+
A G K D S G K A K
y8 y7 y6 y5 y4 y3 y2 y1
b2 b3 b4
Relative Intensity (%)
m/z
a2+ 101.07
y1-H2O+ 129.10
b2+ 129.07
y1+ 147.11
y2-H2O+ 200.14
y2+ 218.15
b3+ 299.17
y4+ 475.29
y5+ 562.32
y6-H2O+ 659.33
y6+ 677.35
y8+ 904.47

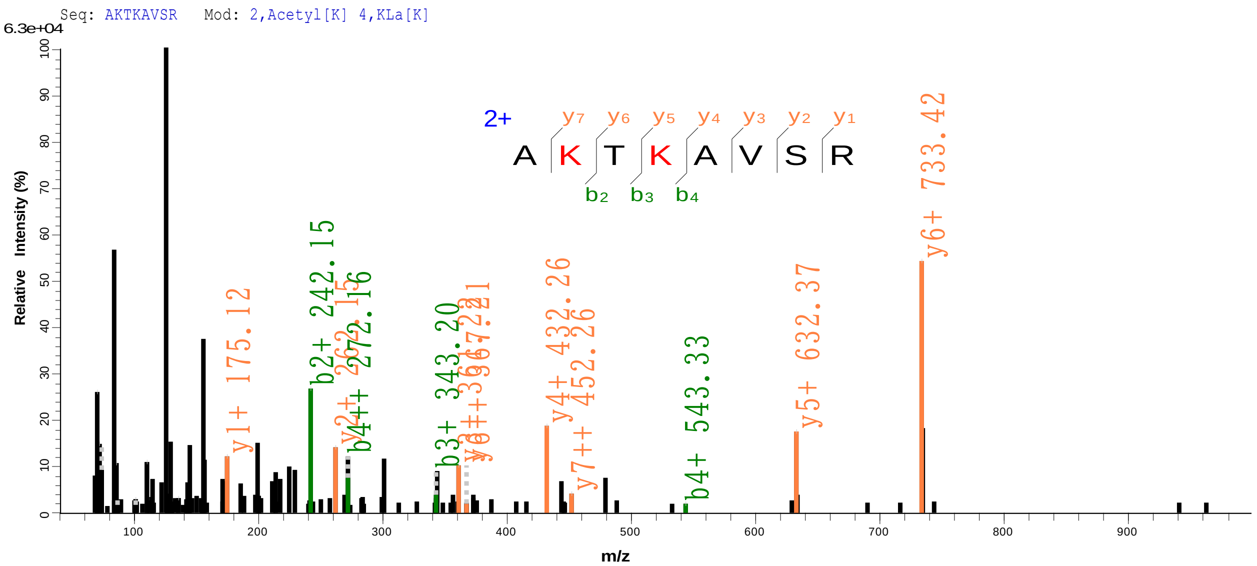
Seq: AKTKAVSR Mod: 2,Acetyl[K] 4,KLa[K]
6.3e+04
Relative Intensity (%)
2+
A K T K A V S R
y7 y6 y5 y4 y3 y2 y1
b2 b3 b4
y1+ 175.12
b2+ 242.15
b4++ 272.16
b3+ 343.20
y4+ 432.26
y7++ 452.26
b4+ 543.33
y5+ 632.37
y6+ 733.42
m/z

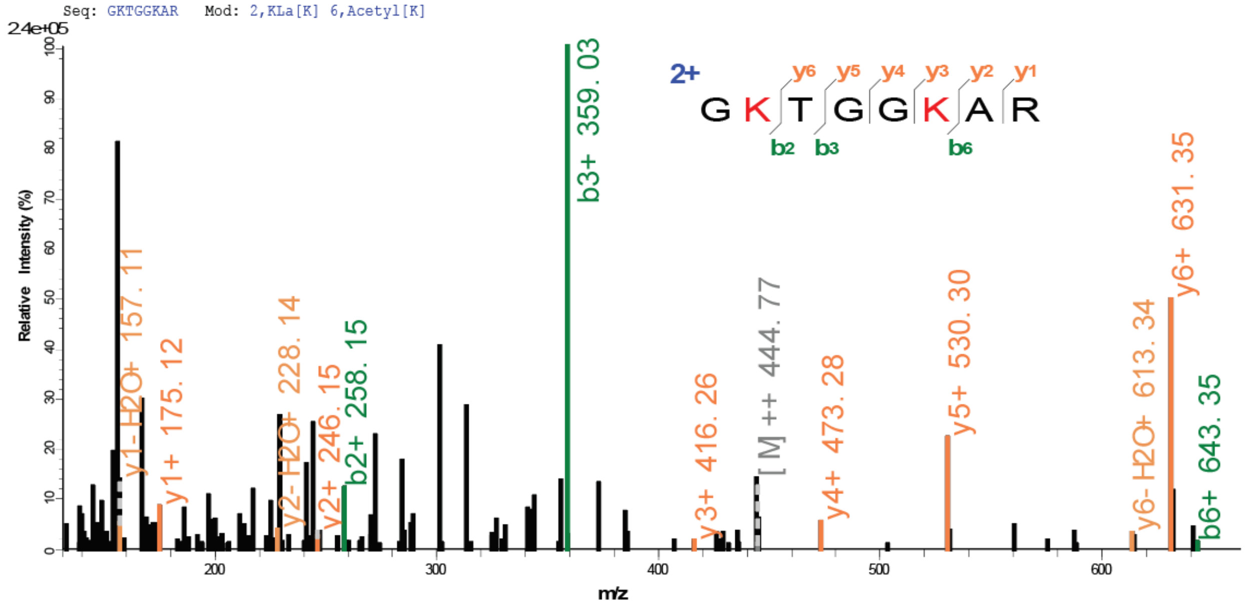
Seq: GKTGGKAR Mod: 2,KLa[K] 6,Acetyl[K]
2.4e+05
Relative Intensity (%)
2+
G K T G G K A R
y6 y5 y4 y3 y2 y1
b2 b3 b6
y1-H2O+ 157.11
y1+ 175.12
y2-H2O+ 228.14
y2+ 246.15
b2+ 258.15
b3+ 359.03
y3+ 416.26
[M]++ 444.77
y4+ 473.28
y5+ 530.30
y6-H2O+ 613.34
y6+ 631.35
b6+ 643.35
m/z

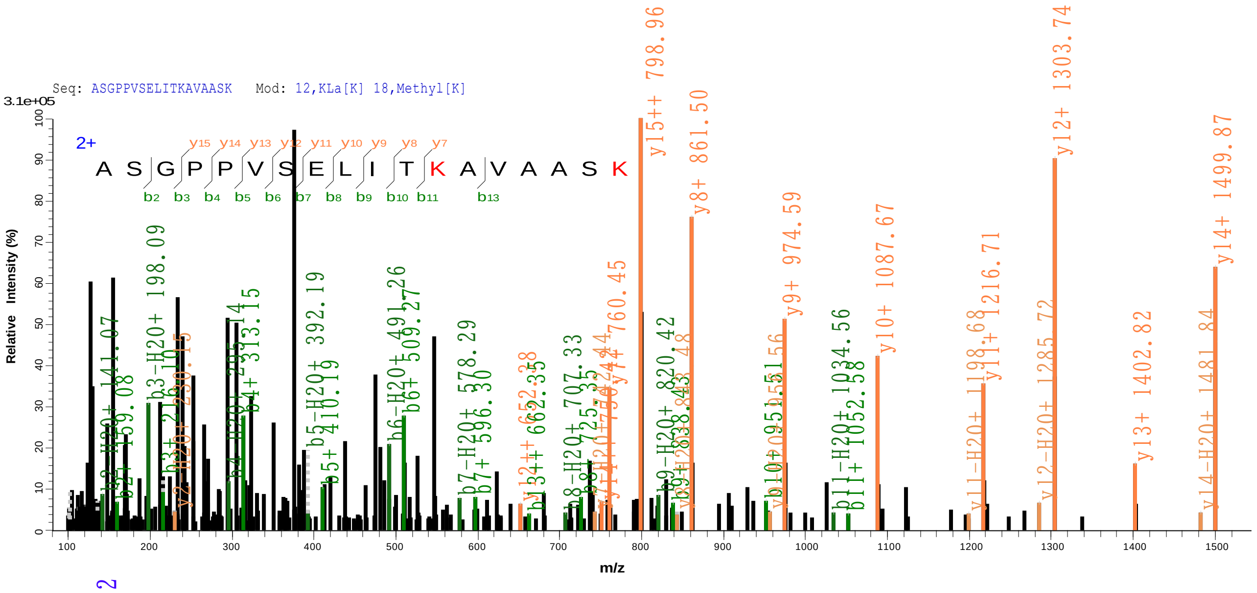

Seq: ASGPPVSELITKAVAASK Mod: 12,KLa[K] 18,Methyl[K]
3.1e+05
Relative Intensity (%)
2+
A S G P P V S E L I T K A V A A S K
y15 y14 y13 y12 y11 y10 y9 y8 y7
b2 b3 b4 b5 b6 b7 b8 b9 b10 b11 b13
y15++ 798.96
y8+ 861.50
y9+ 974.59
y10+ 1087.67
y11+ 1216.71
y12+ 1303.74
y13+ 1402.82
y14+ 1499.87
b3-H2O+ 198.09
b4+ 313.15
b5-H2O+ 392.19
b5+ 410.19
b6-H2O+ 491.26
b6+ 509.27
b7-H2O+ 578.29
b7+ 596.30
b11-H2O+ 1034.56
b11+ 1052.58
y4+ 760.45
m/z

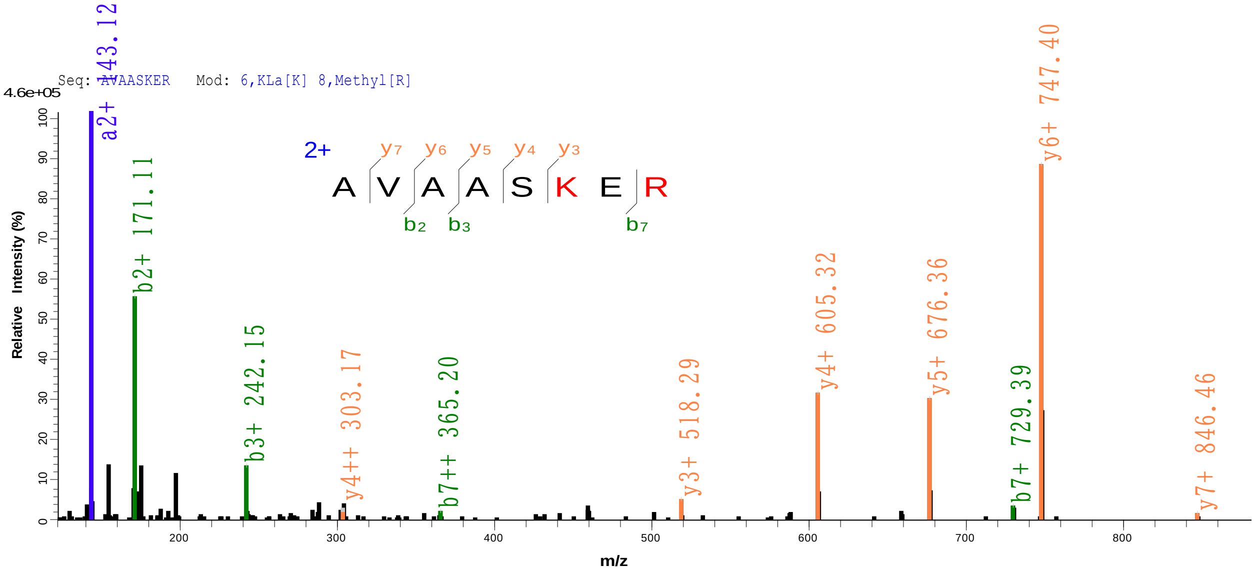

Seq: AVAASKER Mod: 6,KLa[K] 8,Methyl[R]
4.6e+05
Relative Intensity (%)
2+
A V A A S K E R
y7 y6 y5 y4 y3
b2 b3 b7
a2+ 143.12
b2+ 171.11
b3+ 242.15
y4++ 303.17
b7++ 365.20
y3+ 518.29
y4+ 605.32
y5+ 676.36
b7+ 729.39
y6+ 747.40
y7+ 846.46
m/z

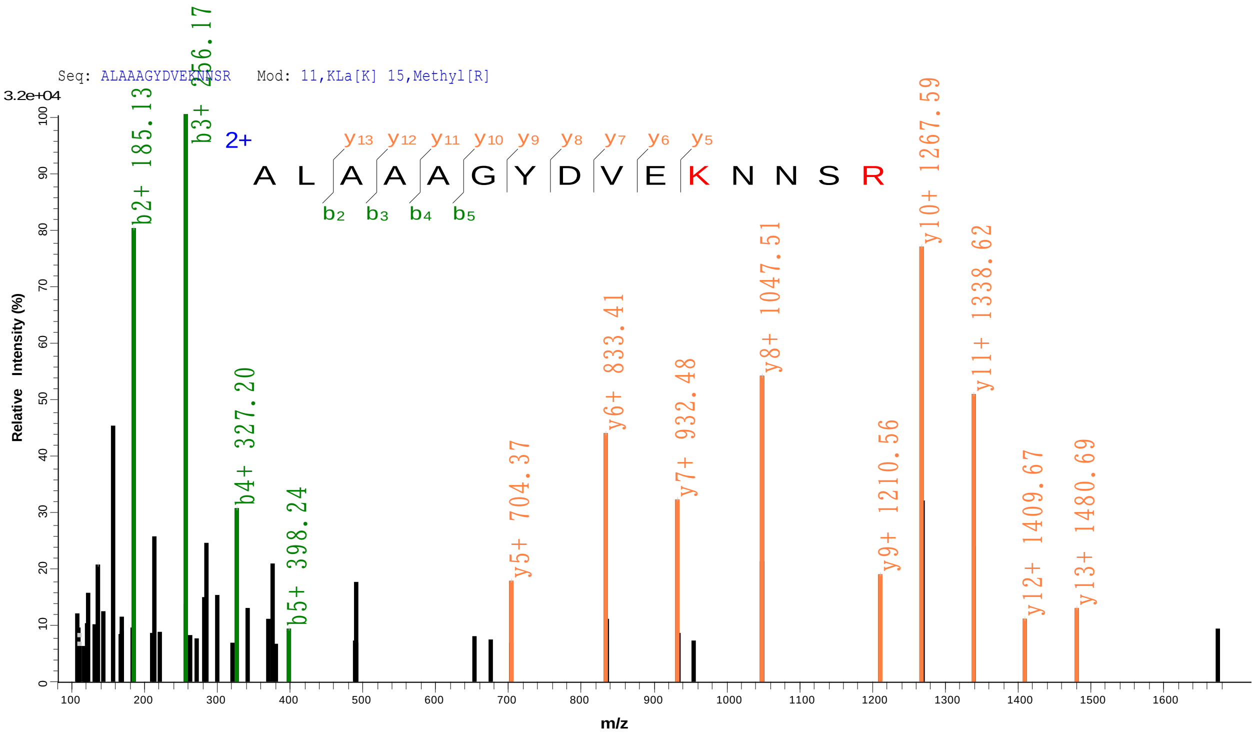
Seq: ALAAAGYDVEKNNSR Mod: 11,KLa[K] 15,Methyl[R]
3.2e+04
Relative Intensity (%)
m/z
2+
A L A A A G Y D V E K N N S R
b2+ 185.13
b3+ 256.17
b4+ 327.20
b5+ 398.24
y5+ 704.37
y6+ 833.41
y7+ 932.48
y8+ 1047.51
y9+ 1210.56
y10+ 1267.59
y11+ 1338.62
y12+ 1409.67
y13+ 1480.69

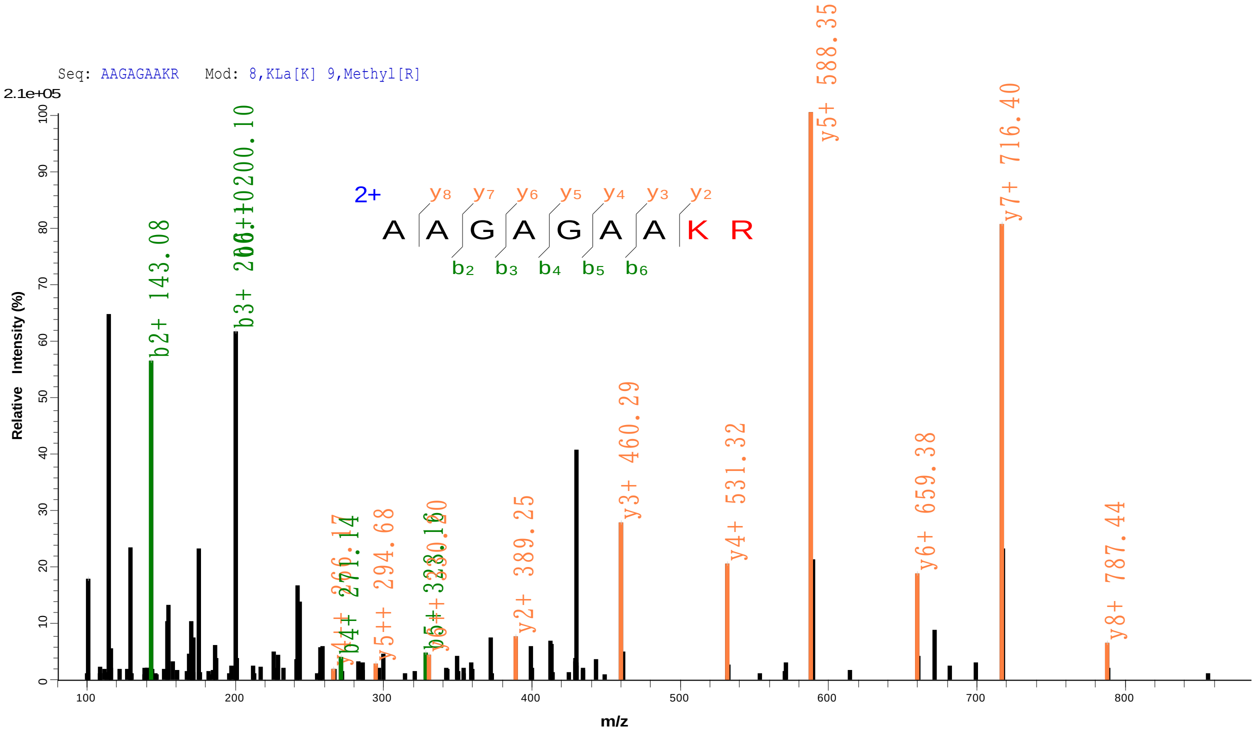
Seq: AAGAGAAKR Mod: 8,KLa[K] 9,Methyl[R]
2.1e+05
Relative Intensity (%)
m/z
2+
A A G A G A A K R
b2+ 143.08
y2+ 389.25
y3+ 460.29
y4+ 531.32
y5+ 588.35
y6+ 659.38
y7+ 716.40
y8+ 787.44
y5++ 294.68

Seq: PAAAGVKK   Mod: 7,KLa[K] 8,Methyl[K]

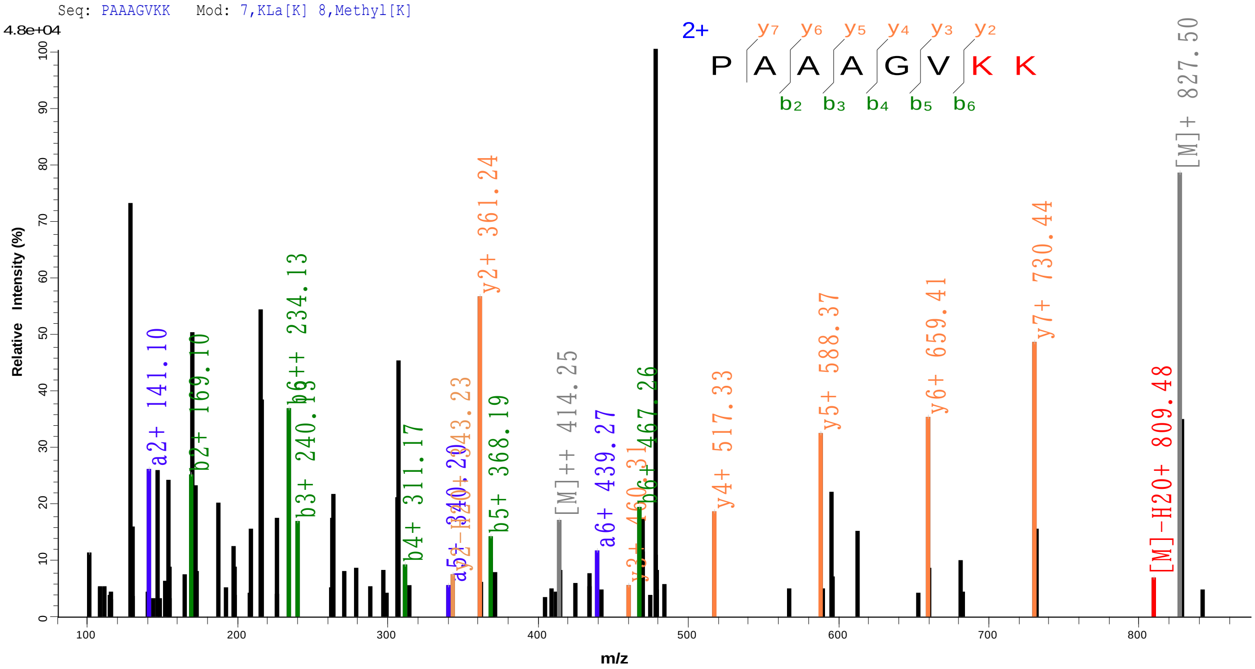

Seq: KVAGAATPKK   Mod: 9,KLa[K] 10,Methyl[K]

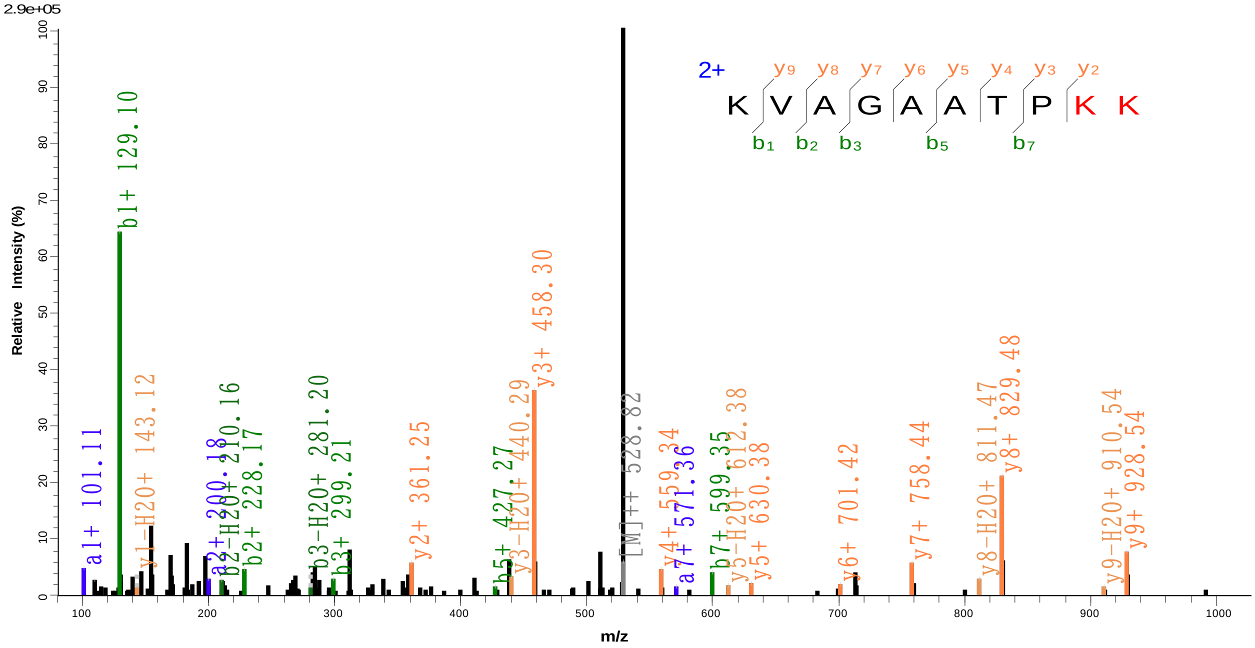

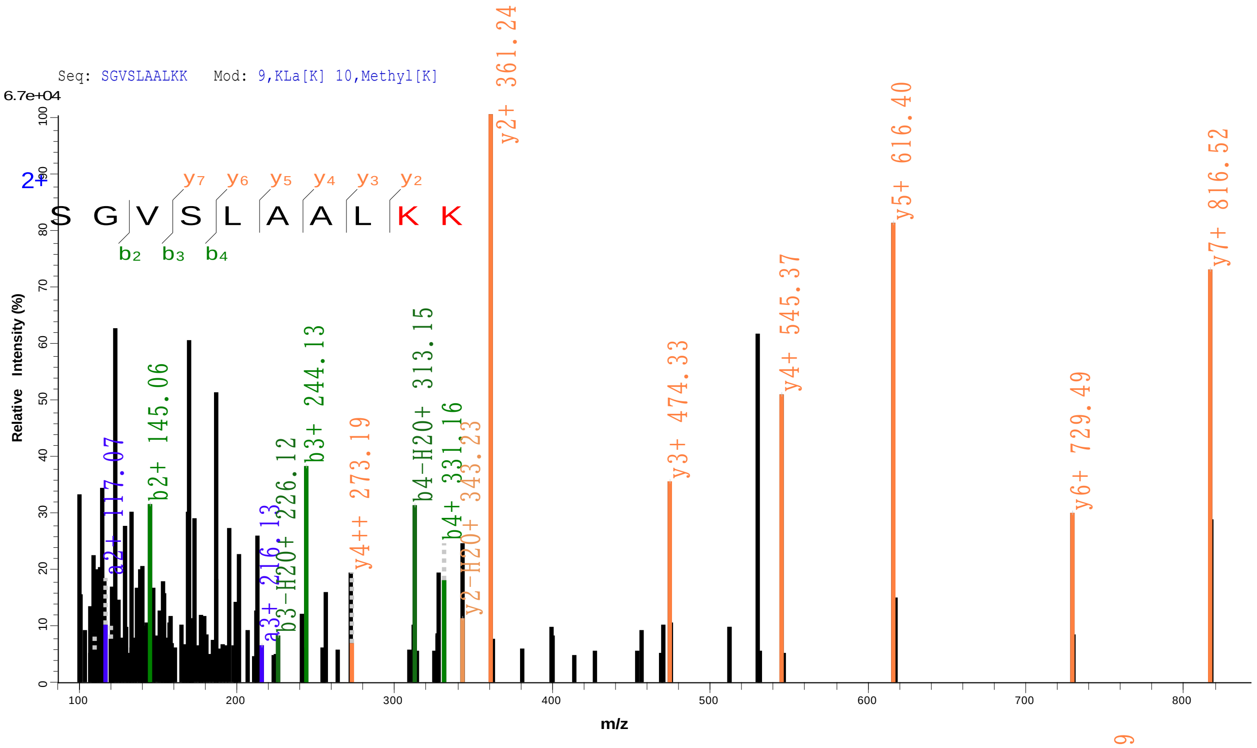
Seq: SGVSLAALKK Mod: 9,KLa[K] 10,Methyl[K]
6.7e+04
Relative Intensity (%)
m/z
y2+ 361.24
y5+ 616.40
y7+ 816.52
y4+ 545.37
y3+ 474.33
y6+ 729.49
b4-H2O+ 313.15
b3+ 244.13
b2+ 145.06
y4++ 273.19
b4+ 331.16
y2-H2O+ 343.23
a2+ 117.07
a3+ 216.13
b3-H2O+ 226.12

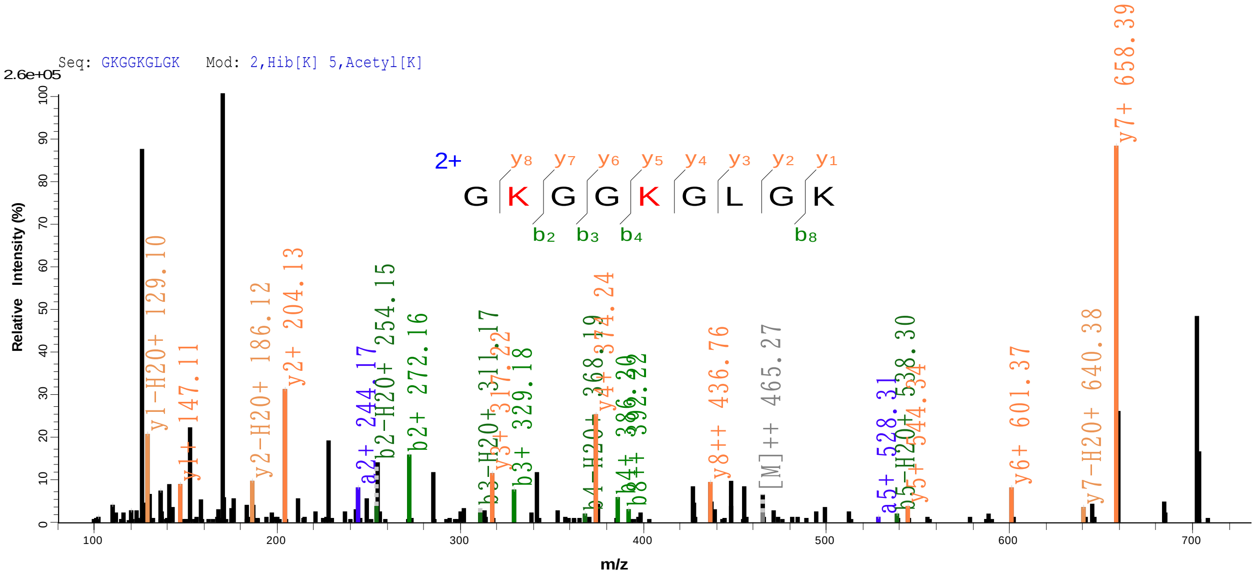
Seq: GKGGKGLGK Mod: 2,Hib[K] 5,Acetyl[K]
2.6e+05
Relative Intensity (%)
m/z
y7+ 658.38
y1-H2O+ 129.10
y1+ 147.11
y2-H2O+ 186.12
y2+ 204.13
a2+ 244.17
b2-H2O+ 254.15
b2+ 272.16
b3-H2O+ 311.17
b3+ 329.18
y8++ 436.76
[M]++ 465.27
a5+ 528.31
b5-H2O+ 538.30
y6+ 601.37
y7-H2O+ 640.38

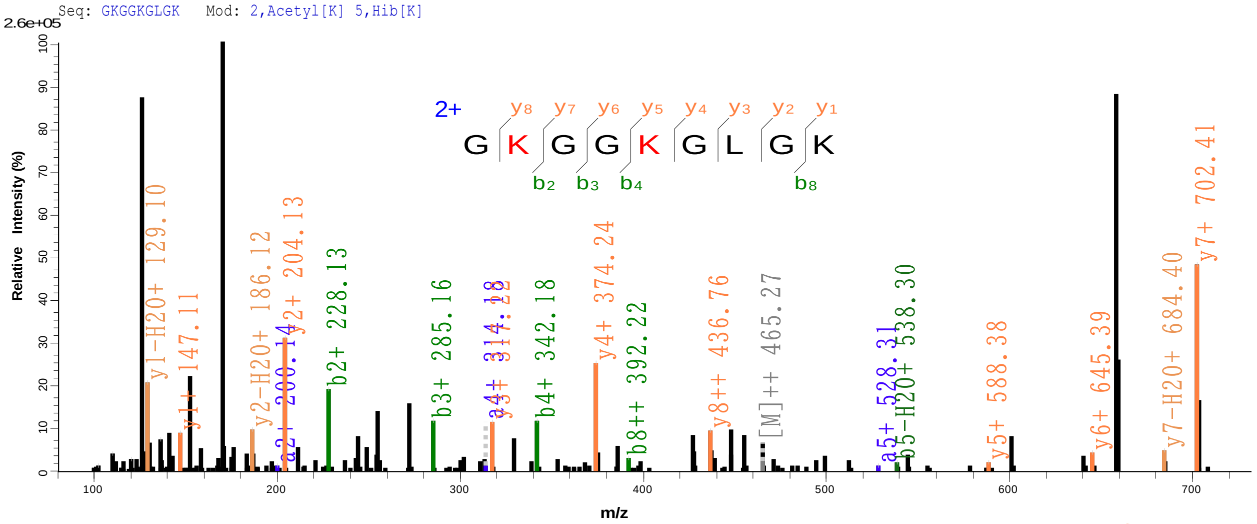
Seq: GKGGKGLGK Mod: 2,Acetyl[K] 5,Hib[K]
2.6e+05
2+
G K G G K G L G K
y8 y7 y6 y5 y4 y3 y2 y1
b2 b3 b4 b8
y1-H2O+ 129.10
y1+ 147.11
y2-H2O+ 186.12
y2+ 204.13
b2+ 228.13
b3+ 285.16
b4+ 342.18
y4+ 374.24
b8++ 392.22
y8++ 436.76
[M]++ 465.27
a5+ 528.31
b5-H2O+ 538.30
y5+ 588.38
y6+ 645.39
y7-H2O+ 684.40
y7+ 702.41
Relative Intensity (%)
m/z

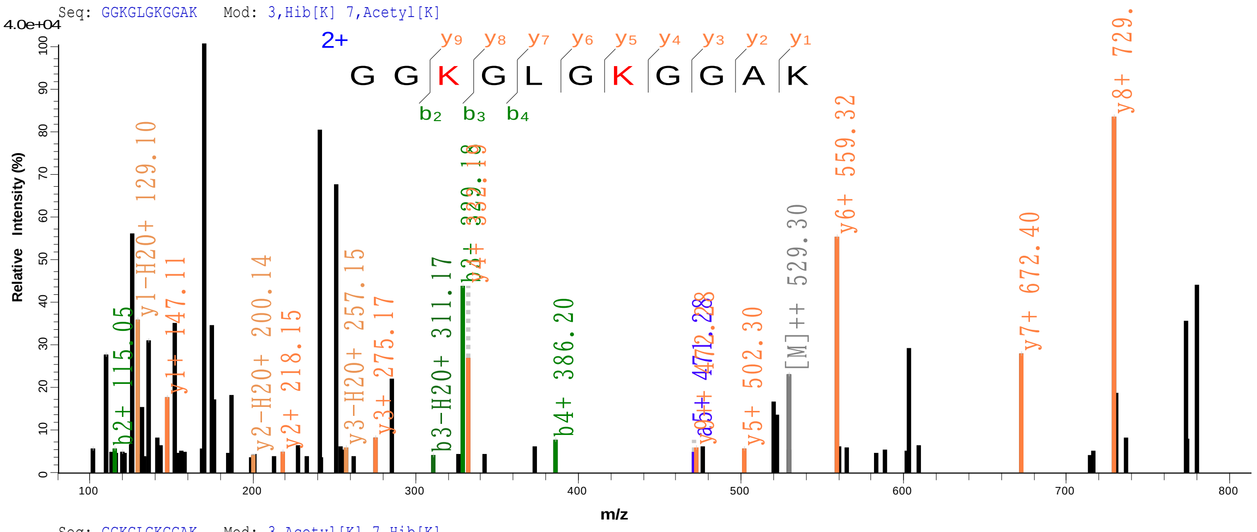
Seq: GGKGLGKGGAK Mod: 3,Hib[K] 7,Acetyl[K]
4.0e+04
2+
G G K G L G K G G A K
y9 y8 y7 y6 y5 y4 y3 y2 y1
b2 b3 b4
b2+ 115.05
y1-H2O+ 129.10
y1+ 147.11
y2-H2O+ 200.14
y2+ 218.15
y3-H2O+ 257.15
y3+ 275.17
b3-H2O+ 311.17
b4+ 386.20
y5+ 502.30
[M]++ 529.30
y6+ 559.32
y7+ 672.40
y8+ 729.42
Relative Intensity (%)
m/z

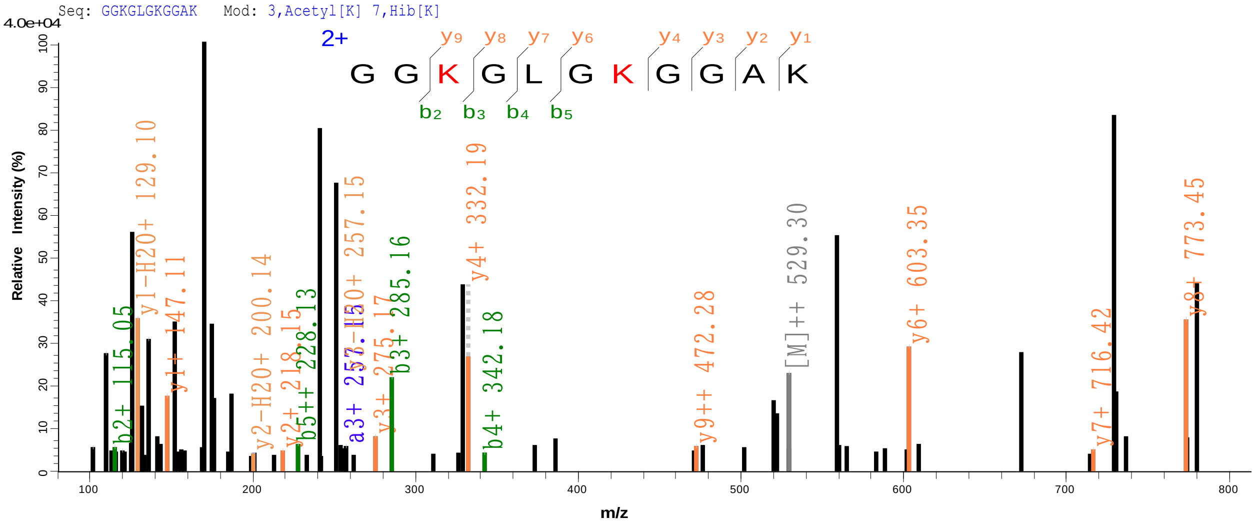
Seq: GGKGLGKGGAK Mod: 3,Acetyl[K] 7,Hib[K]
4.0e+04
2+
G G K G L G K G G A K
y9 y8 y7 y6 y4 y3 y2 y1
b2 b3 b4 b5
b2+ 115.05
y1-H2O+ 129.10
y1+ 147.11
y2-H2O+ 200.14
b5++ 228.13
y3-H2O+ 257.15
y3+ 275.17
b3+ 285.16
y4+ 332.19
b4+ 342.18
y9++ 472.28
[M]++ 529.30
y6+ 603.35
y7+ 716.42
y8+ 773.45
Relative Intensity (%)
m/z

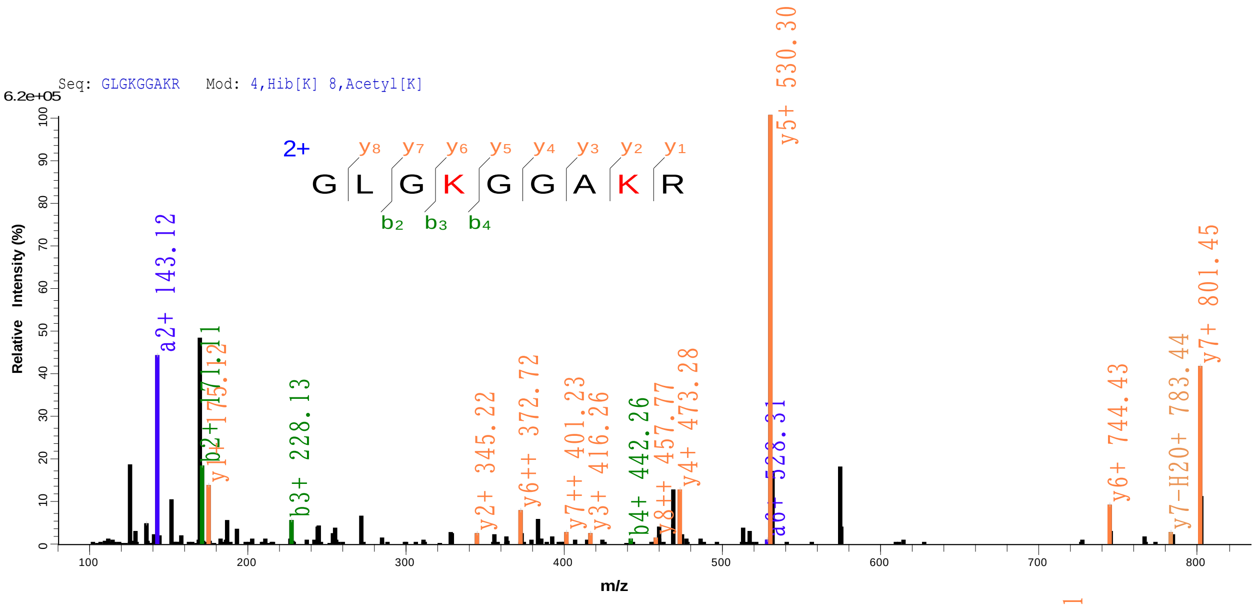

Seq: GLGKGGAKR Mod: 4,Hib[K] 8,Acetyl[K]
6.2e+05
Relative Intensity (%)
m/z
2+
G L G K G G A K R
a2+ 143.12
b3+ 228.13
y2+ 345.22
y6++ 372.72
y7++ 401.23
y3+ 416.26
b4+ 442.26
y4+ 473.28
y5+ 530.30
y6+ 744.43
y7-H2O+ 783.44
y7+ 801.45

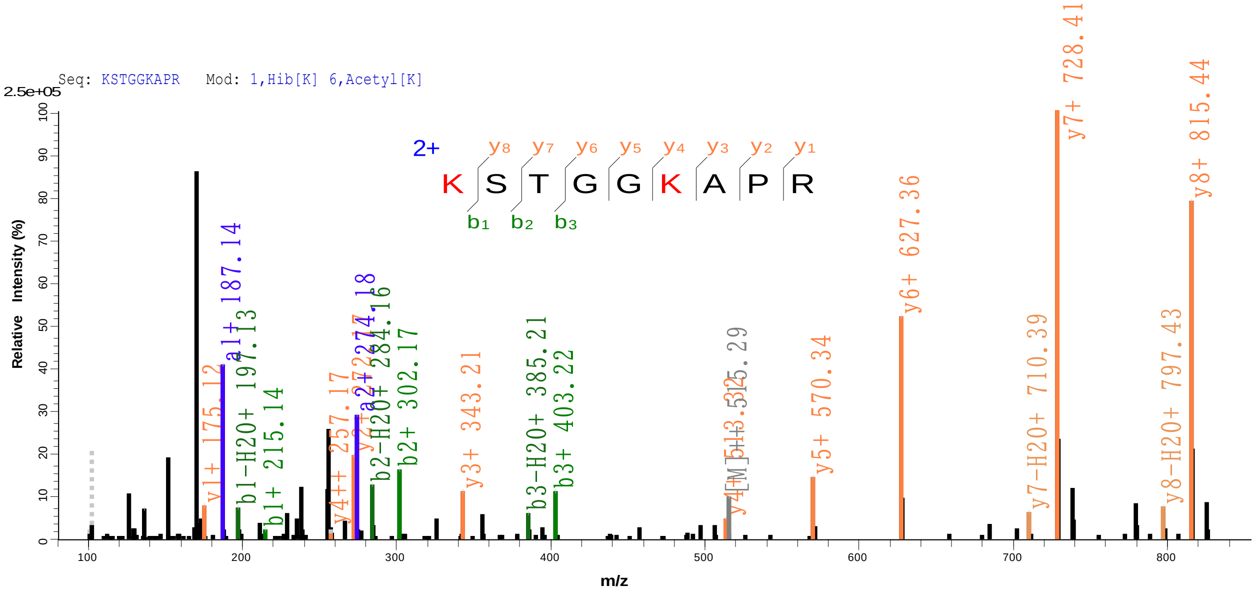

Seq: KSTGGKAPR Mod: 1,Hib[K] 6,Acetyl[K]
2.5e+05
Relative Intensity (%)
m/z
2+
K S T G G K A P R
y1+ 175.12
a1+ 187.14
b1-H2O+ 197.13
b1+ 215.14
b2-H2O+ 284.16
b2+ 302.17
y3+ 343.21
b3-H2O+ 385.21
b3+ 403.22
y5+ 570.34
y6+ 627.36
y7-H2O+ 710.39
y7+ 728.41
y8-H2O+ 797.43
y8+ 815.44

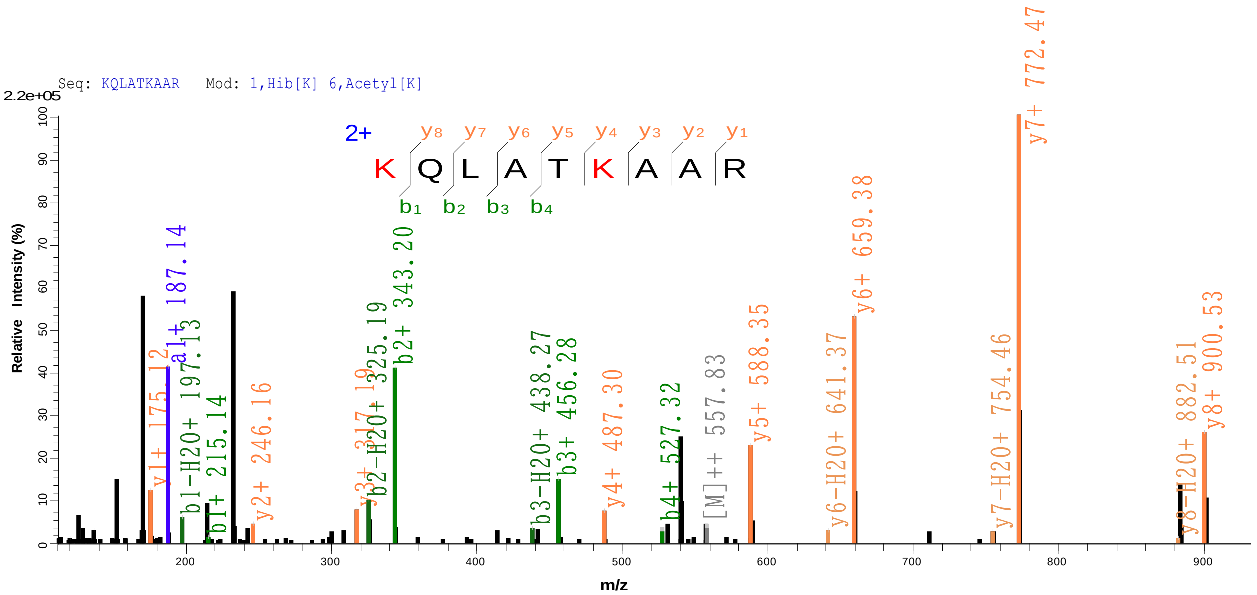
Seq: KQLATKAAR Mod: 1,Hib[K] 6,Acetyl[K]
2.2e+05
Relative Intensity (%)
m/z
2+
K Q L A T K A A R
y8 y7 y6 y5 y4 y3 y2 y1
b1 b2 b3 b4
y7+ 772.47
y6+ 659.38
y8+ 900.53
y8-H2O+ 882.51
y7-H2O+ 754.46
y6-H2O+ 641.37
y5+ 588.35
[M]++ 557.83
b4+ 527.32
y4+ 487.30
b3+ 456.28
b3-H2O+ 438.27
b2+ 343.20
b2-H2O+ 325.19
y3+ 317.19
y2+ 246.16
b1+ 215.14
b1-H2O+ 197.13
a1+ 187.14
y1+ 175.12

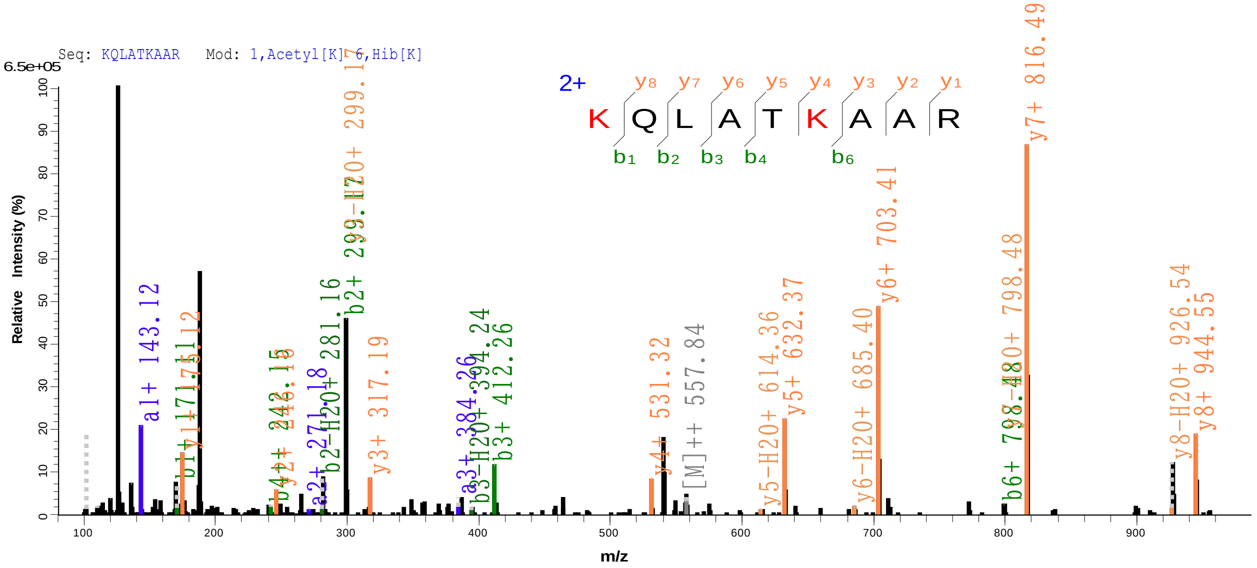
Seq: KQLATKAAR Mod: 1,Acetyl[K] 6,Hib[K]
6.5e+05
Relative Intensity (%)
m/z
2+
K Q L A T K A A R
y8 y7 y6 y5 y4 y3 y2 y1
b1 b2 b3 b4 b6
y7+ 816.49
y7-H2O+ 798.48
b6+ 798.48
y6+ 703.41
y6-H2O+ 685.40
y5+ 632.37
y5-H2O+ 614.36
y8+ 944.55
y8-H2O+ 926.54
[M]++ 557.84
y4+ 531.32
b3+ 412.26
b3-H2O+ 394.24
a3+ 384.26
y3+ 317.19
b2+ 299.17
b2-H2O+ 281.16
a2+ 271.18
y2+ 246.16
b1+ 171.12
y1+ 175.12
a1+ 143.12

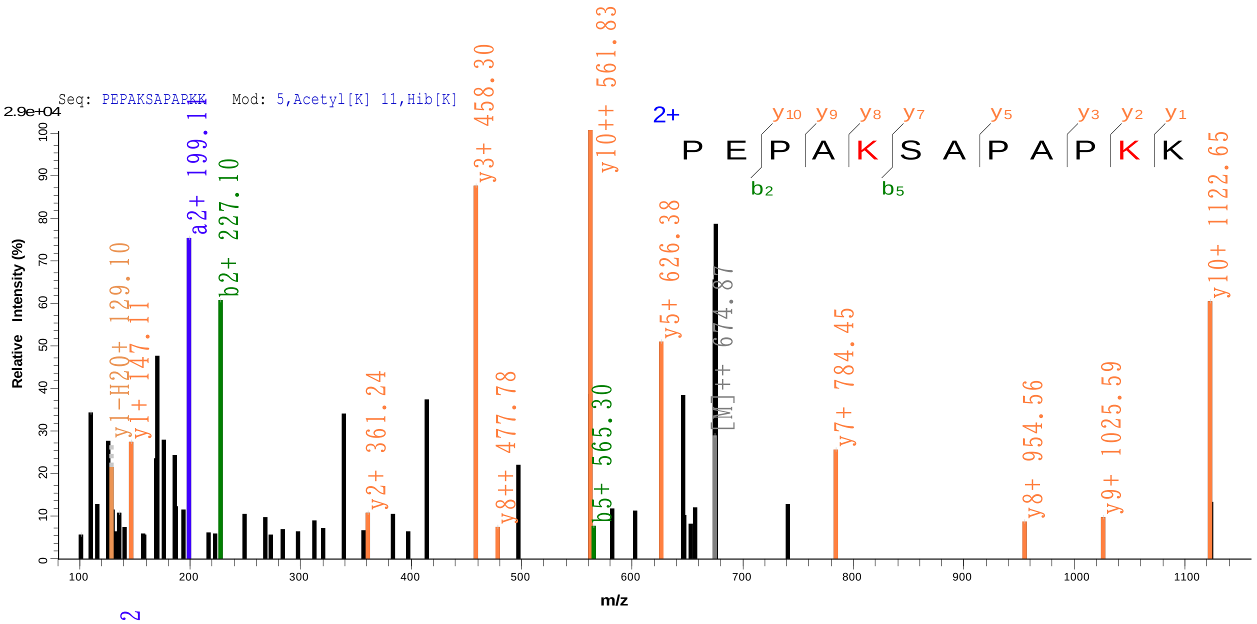
Seq: PEPAKSAPAPKK Mod: 5,Acetyl[K] 11,Hib[K]
2.9e+04
2+
P E P A K S A P A P K K
Relative Intensity (%)
m/z
a2+ 199.1
b2+ 227.10
y1-H2O+ 129.10
y1+ 147.11
y2+ 361.24
y3+ 458.30
y8++ 477.78
y10++ 561.83
b5+ 565.30
y5+ 626.38
[M]++ 674.87
y7+ 784.45
y8+ 954.56
y9+ 1025.59
y10+ 1122.65
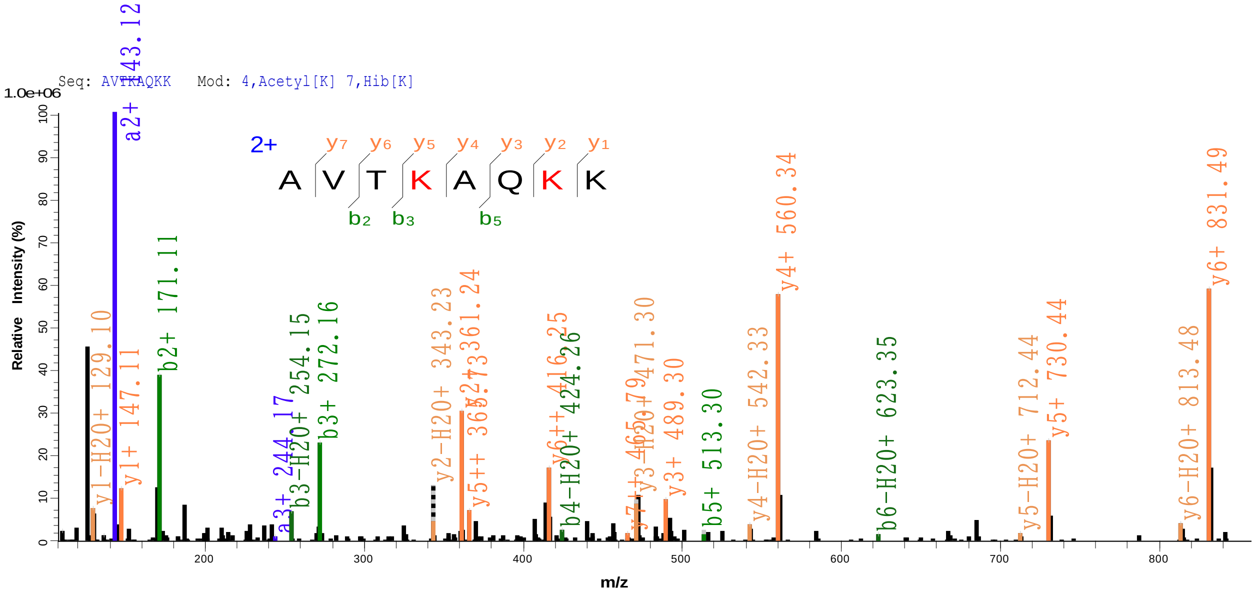
Seq: AVTKAQKK Mod: 4,Acetyl[K] 7,Hib[K]
1.0e+06
2+
A V T K A Q K K
Relative Intensity (%)
m/z
a2+ 143.12
y1-H2O+ 129.10
y1+ 147.11
b2+ 171.11
a3+ 244.17
b3-H2O+ 254.15
b3+ 272.16
y2-H2O+ 343.23
y5++ 365.73
y2+ 361.24
y6++ 416.25
b4-H2O+ 424.26
y3-H2O+ 471.30
y3+ 489.30
b5+ 513.30
y4-H2O+ 542.33
y4+ 560.34
b6-H2O+ 623.35
y5-H2O+ 712.44
y5+ 730.44
y6-H2O+ 813.48
y6+ 831.49
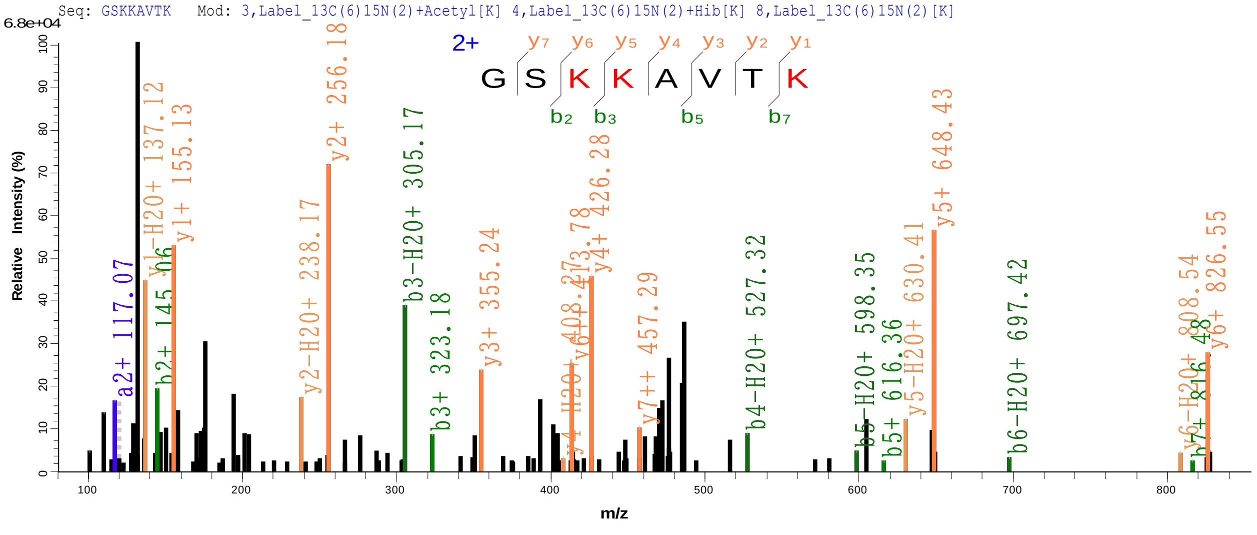
Seq: GSKKAVTK Mod: 3,Label_13C(6)15N(2)+Acetyl[K] 4,Label_13C(6)15N(2)+Hib[K] 8,Label_13C(6)15N(2)[K]
6.8e+04
2+
G S K K A V T K
Relative Intensity (%)
m/z
a2+ 117.07
y1-H2O+ 137.12
b2+ 145.06
y1+ 155.13
y2-H2O+ 238.17
y2+ 256.18
b3-H2O+ 305.17
b3+ 323.18
y3+ 355.24
y4-H2O+ 408.27
y4+ 426.28
y7++ 457.29
b4-H2O+ 527.32
b5-H2O+ 598.35
b5+ 616.36
y5-H2O+ 630.41
y5+ 648.43
b6-H2O+ 697.42
y6-H2O+ 808.54
b7+ 816.48
y6+ 826.55

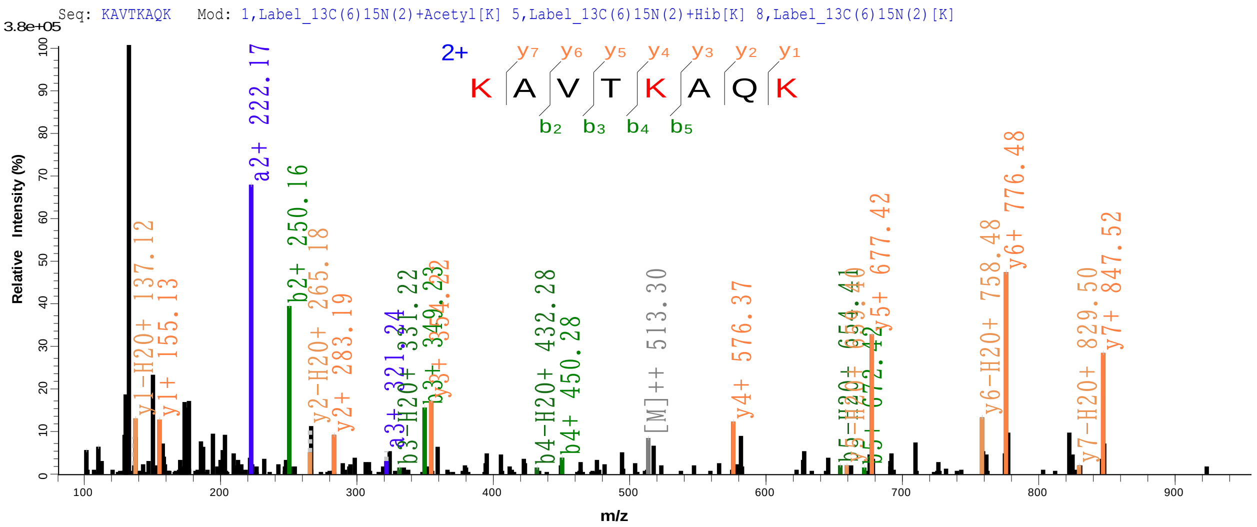
Seq: KAVTKAQK Mod: 1,Label_13C(6)15N(2)+Acetyl[K] 5,Label_13C(6)15N(2)+Hib[K] 8,Label_13C(6)15N(2)[K]
3.8e+05
Relative Intensity (%)
m/z
2+
K A V T K A Q K
a2+ 222.17
b2+ 250.16
y6+ 776.48
y6-H2O+ 758.48
y7+ 847.52
y7-H2O+ 829.50
y5+ 677.42
y4+ 576.37
[M]++ 513.30
b4+ 450.28
b4-H2O+ 432.28
y2+ 283.19
y2-H2O+ 265.18
y1+ 155.13
y1-H2O+ 137.12

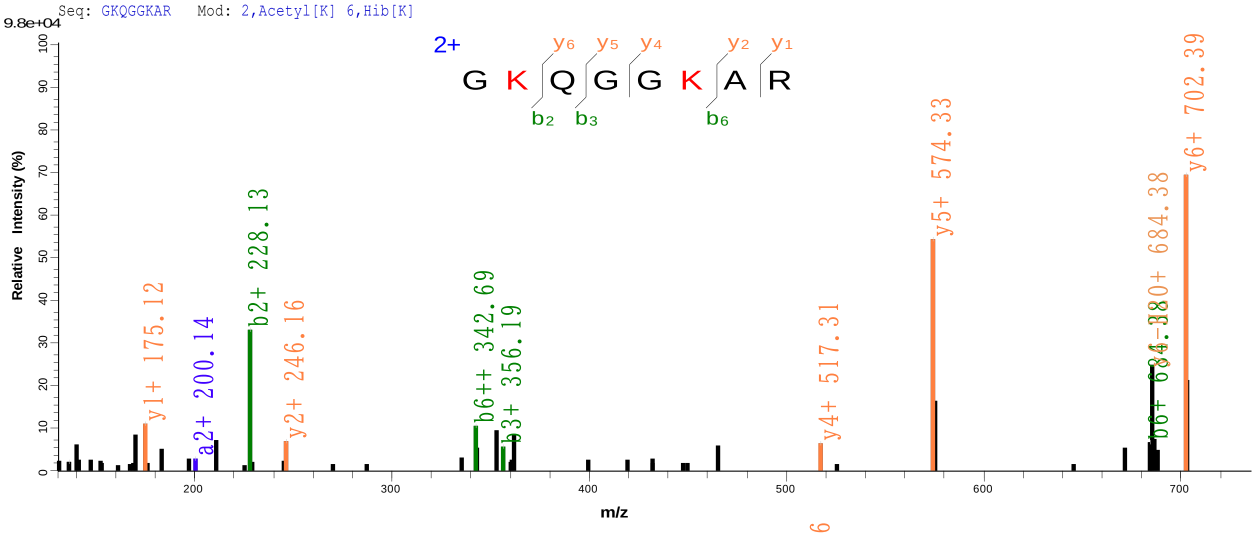
Seq: GKQGGKAR Mod: 2,Acetyl[K] 6,Hib[K]
9.8e+04
Relative Intensity (%)
m/z
2+
G K Q G G K A R
y6+ 702.39
y5+ 574.33
y4+ 517.31
b2+ 228.13
y1+ 175.12
a2+ 200.14
y2+ 246.16
b6++ 342.69
b3+ 356.19

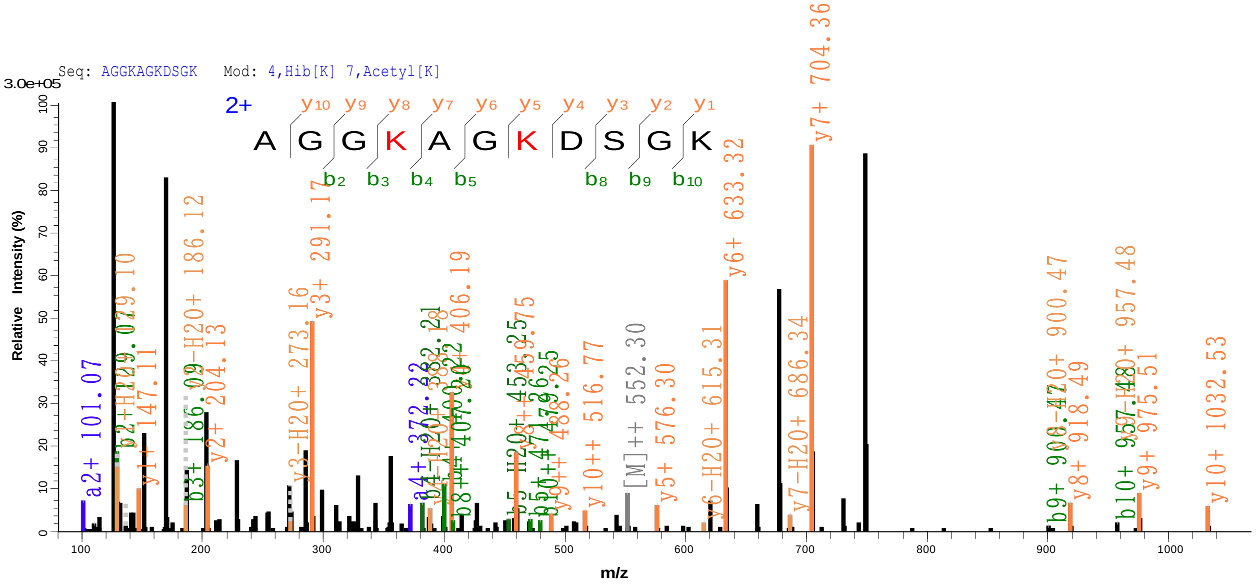
Seq: AGGKAGKDSGK Mod: 4,Hib[K] 7,Acetyl[K]
3.0e+05
Relative Intensity (%)
m/z
2+
A G G K A G K D S G K
y7+ 704.36
y6+ 633.32
y6-H2O+ 615.31
y7-H2O+ 686.34
y3+ 291.17
y3-H2O+ 273.16
a2+ 101.07
y2+ 204.13
y10+ 1032.53
y9+ 975.51
y8+ 918.49
y5+ 576.30
[M]++ 552.30
y10++ 516.77

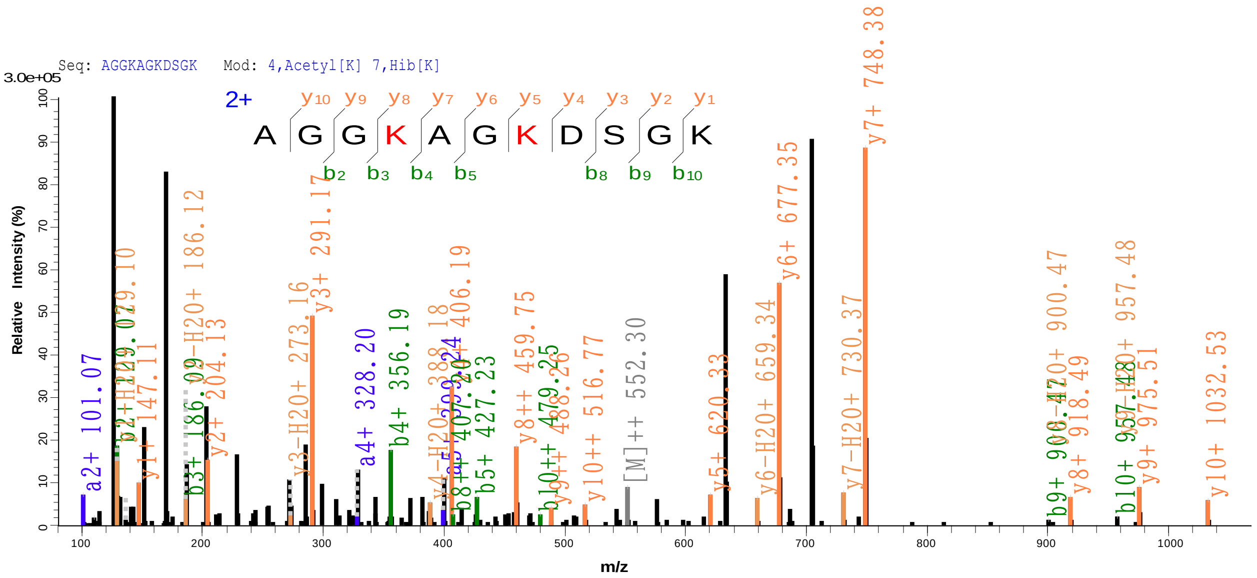
Seq: AGGKAGKDSGK Mod: 4,Acetyl[K] 7,Hib[K]
3.0e+05
Relative Intensity (%)
m/z
y7+ 748.38
y6+ 677.35

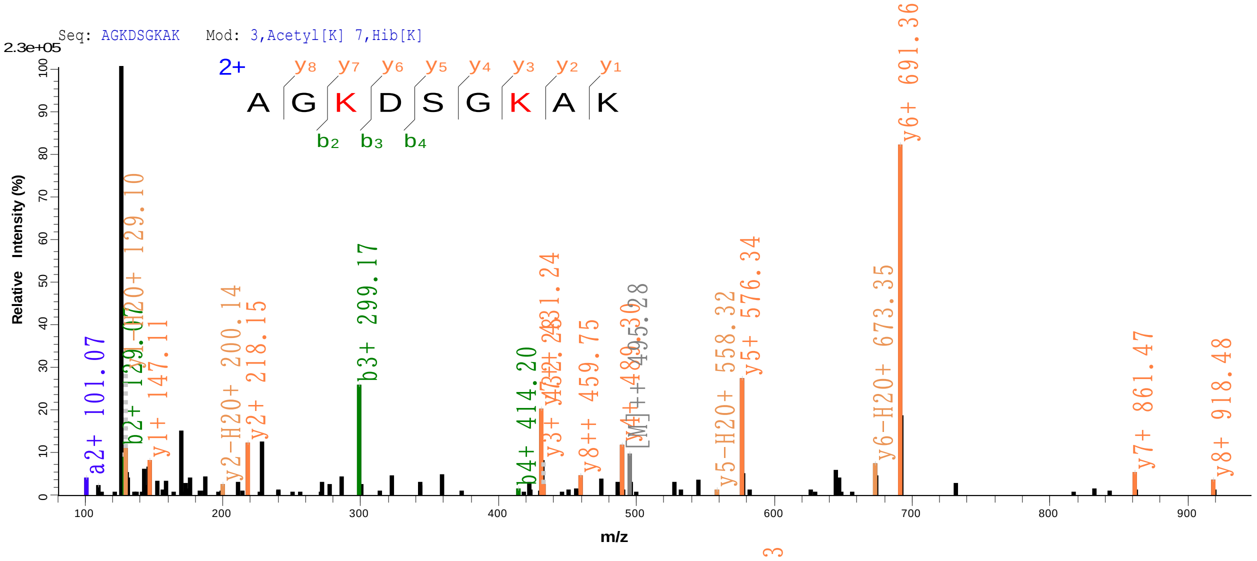
Seq: AGKDSGKAK Mod: 3,Acetyl[K] 7,Hib[K]
2.3e+05
Relative Intensity (%)
m/z
y6+ 691.36
b3+ 299.17

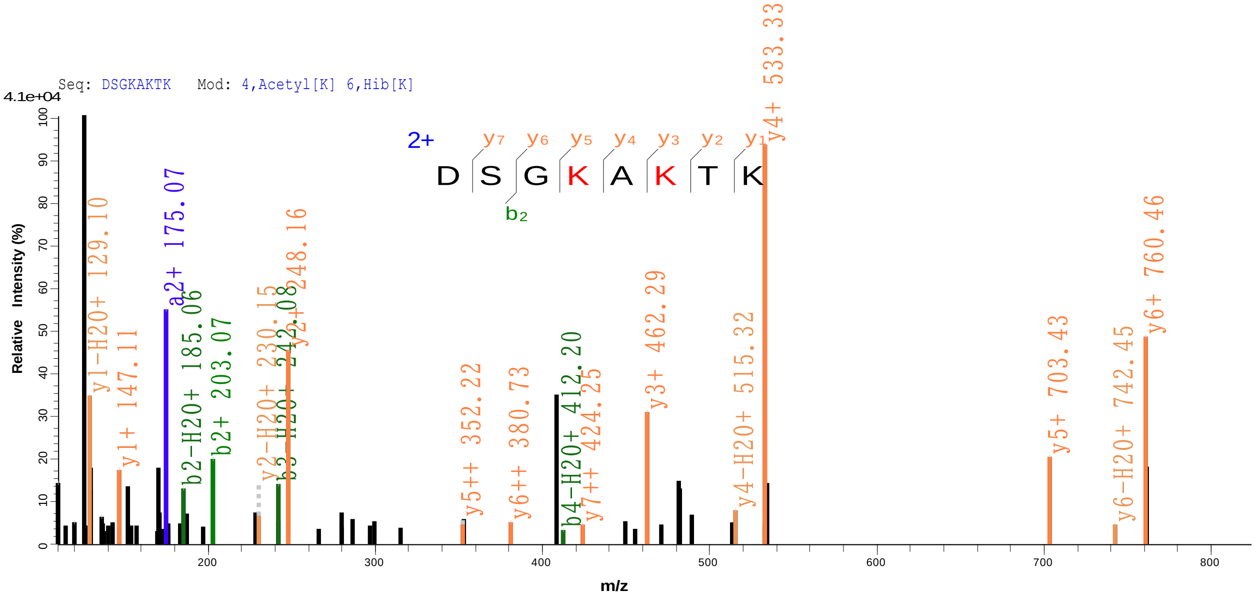
Seq: DSGKAKTK Mod: 4,Acetyl[K] 6,Hib[K]
4.1e+04
Relative Intensity (%)
m/z
y4+ 533.33
y6+ 760.46

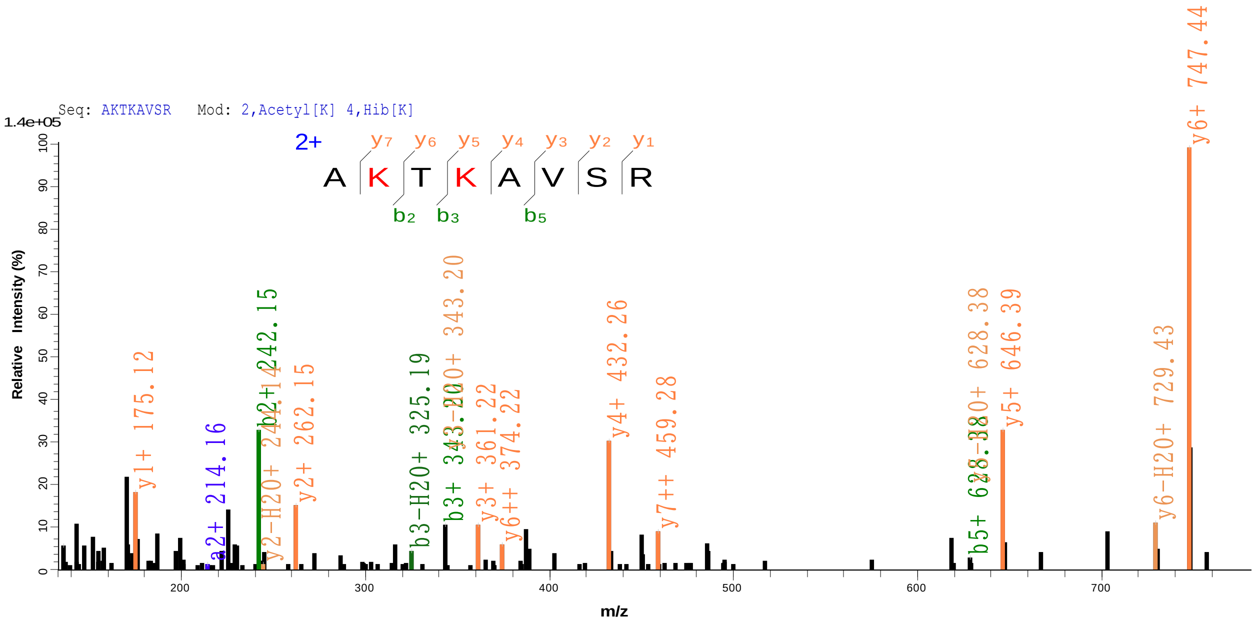
Seq: AKTKAVSR Mod: 2,Acetyl[K] 4,Hib[K]
1.4e+05
2+
A K T K A V S R
Relative Intensity (%)
m/z
y1+ 175.12
a2+ 214.16
b2+ 242.15
y2+ 262.15
b3-H2O+ 325.19
b3+ 343.20
y3+ 361.22
y6++ 374.22
y4+ 432.26
y7++ 459.28
b5+ 628.38
y5+ 646.39
y6-H2O+ 729.43
y6+ 747.44

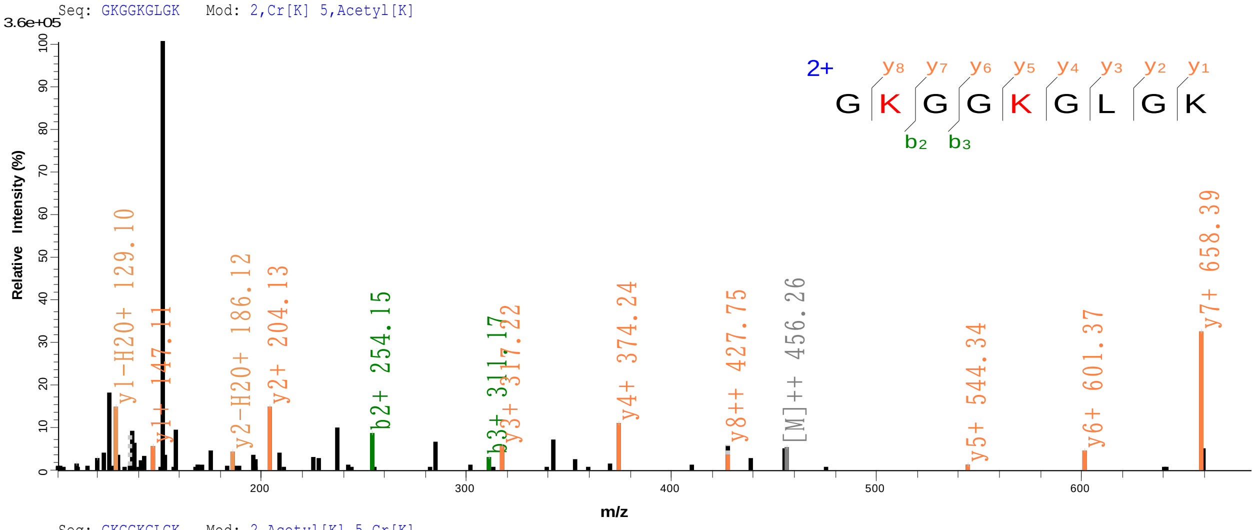
Seq: GKGGKGLGK Mod: 2,Cr[K] 5,Acetyl[K]
3.6e+05
2+
G K G G K G L G K
Relative Intensity (%)
m/z
y1-H2O+ 129.10
y2-H2O+ 186.12
y2+ 204.13
b2+ 254.15
y4+ 374.24
y8++ 427.75
[M]++ 456.26
y5+ 544.34
y6+ 601.37
y7+ 658.39

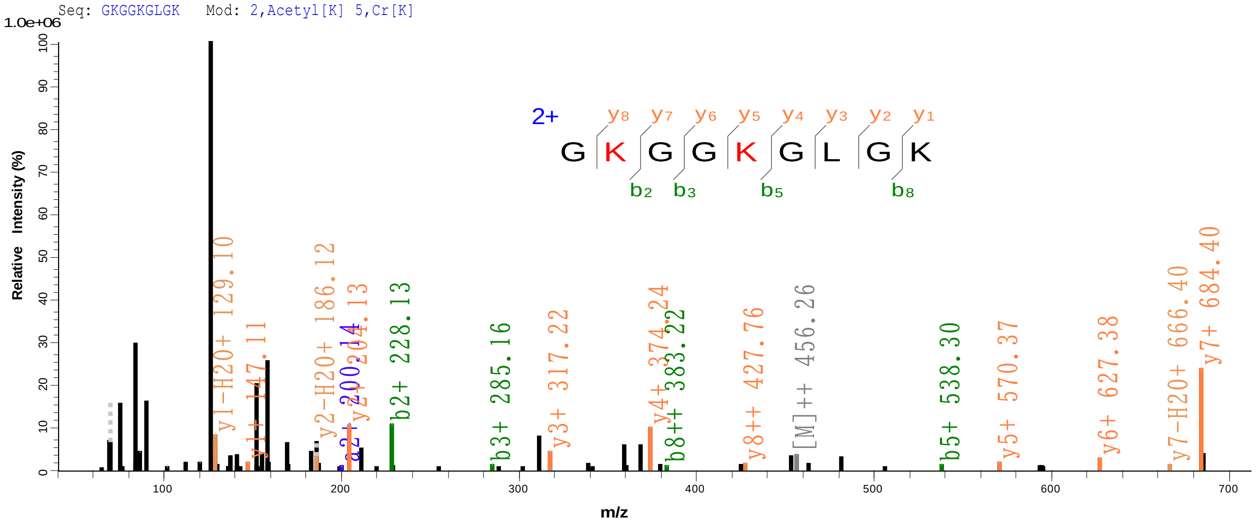
Seq: GKGGKGLGK Mod: 2,Acetyl[K] 5,Cr[K]
1.0e+06
2+
G K G G K G L G K
Relative Intensity (%)
m/z
y1-H2O+ 129.10
y1+ 147.11
y2-H2O+ 186.12
b2+ 228.13
b3+ 285.16
y3+ 317.22
y8++ 427.76
[M]++ 456.26
b5+ 538.30
y5+ 570.37
y6+ 627.38
y7-H2O+ 666.40
y7+ 684.40

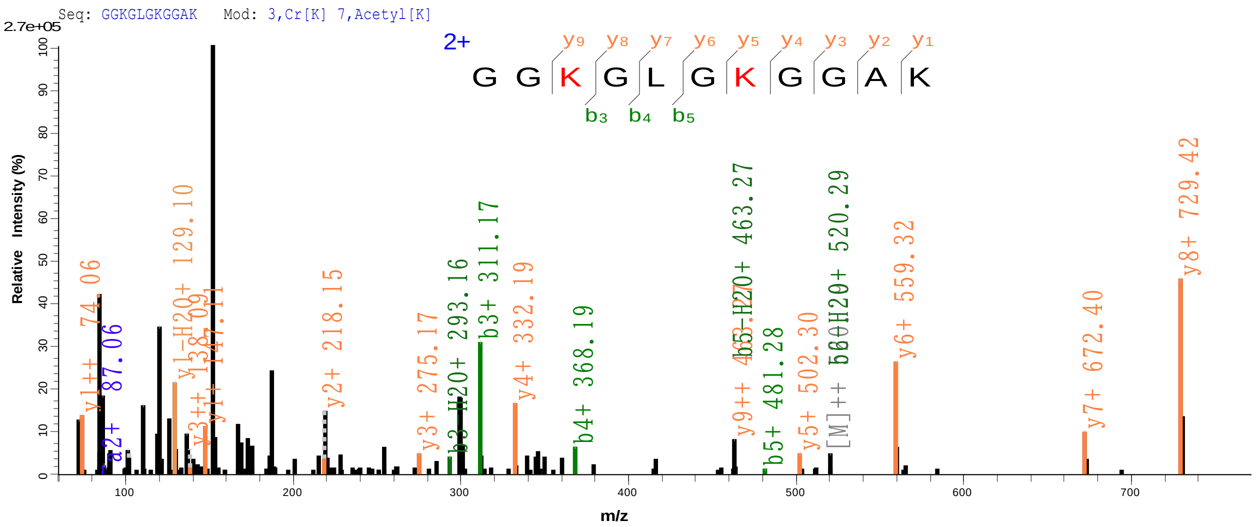
Seq: GGKGLGKGGAK Mod: 3,Cr[K] 7,Acetyl[K]
2.7e+05
2+
G G K G L G K G G A K
y9 y8 y7 y6 y5 y4 y3 y2 y1
b3 b4 b5
Relative Intensity (%)
m/z
87.06
y2+ 218.15
y3+ 275.17
b3-H2O+ 293.16
b3+ 311.17
y4+ 332.19
b4+ 368.19
b5+ 481.28
y5+ 502.30
y6+ 559.32
y7+ 672.40
y8+ 729.42

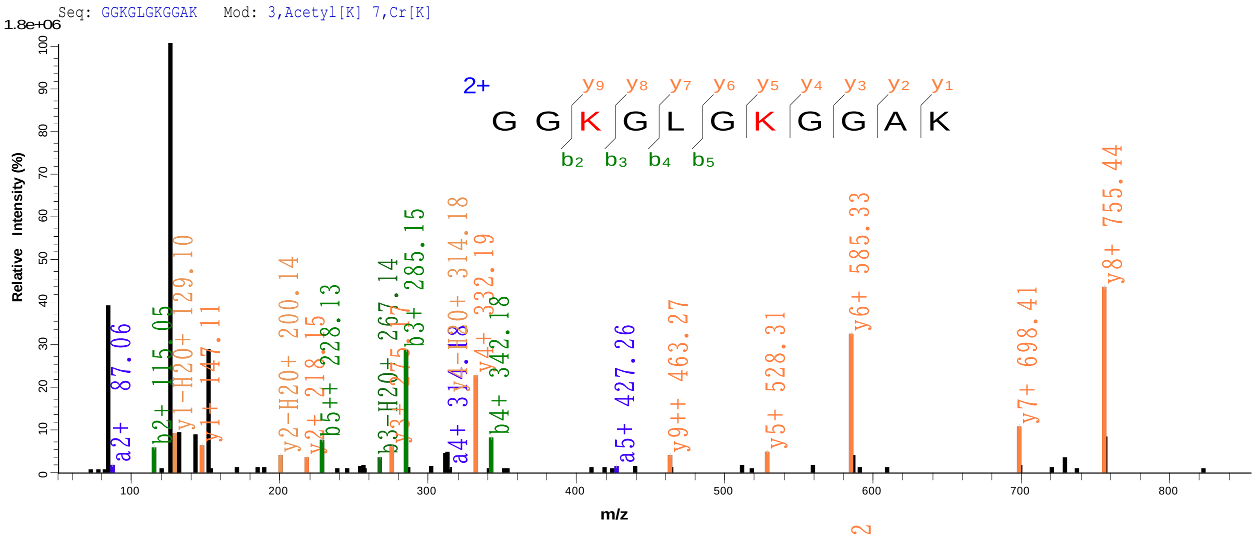
Seq: GGKGLGKGGAK Mod: 3,Acetyl[K] 7,Cr[K]
1.8e+06
2+
G G K G L G K G G A K
y9 y8 y7 y6 y5 y4 y3 y2 y1
b2 b3 b4 b5
Relative Intensity (%)
m/z
87.06
b2+ 115.05
y1-H2O+ 129.10
y1+ 147.11
y2-H2O+ 200.14
b5++ 228.13
b3+ 285.15
b4+ 342.18
a5+ 427.26
y9++ 463.27
y5+ 528.31
y6+ 585.33
y7+ 698.41
y8+ 755.44

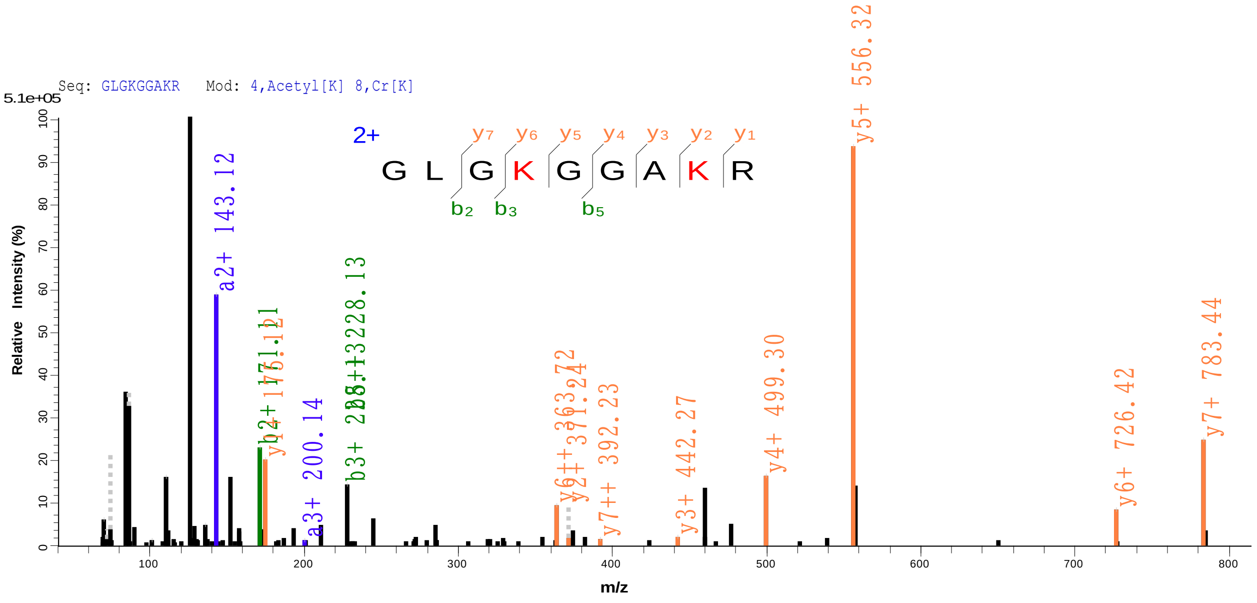
Seq: GLGKGGAKR Mod: 4,Acetyl[K] 8,Cr[K]
5.1e+05
2+
G L G K G G A K R
y7 y6 y5 y4 y3 y2 y1
b2 b3 b5
Relative Intensity (%)
m/z
a2+ 143.12
a3+ 200.14
y7++ 392.23
y3+ 442.27
y4+ 499.30
y5+ 556.32
y6+ 726.42
y7+ 783.44

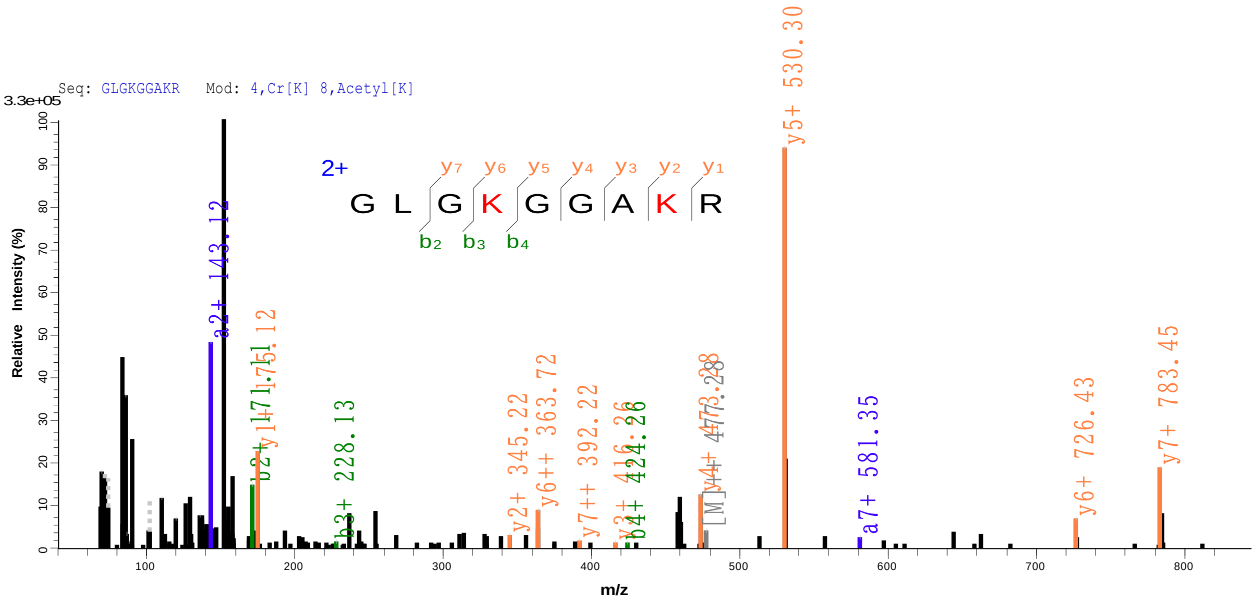

Seq: GLGKGGAKR Mod: 4,Cr[K] 8,Acetyl[K]
3.3e+05
Relative Intensity (%)
m/z
2+
G L G K G G A K R
y7 y6 y5 y4 y3 y2 y1
b2 b3 b4
y5+ 530.30
a2+ 143.12
y1+ 175.12
b3+ 228.13
y2+ 345.22
y6++ 363.72
y7++ 392.22
a7+ 581.35
y6+ 726.43
y7+ 783.45

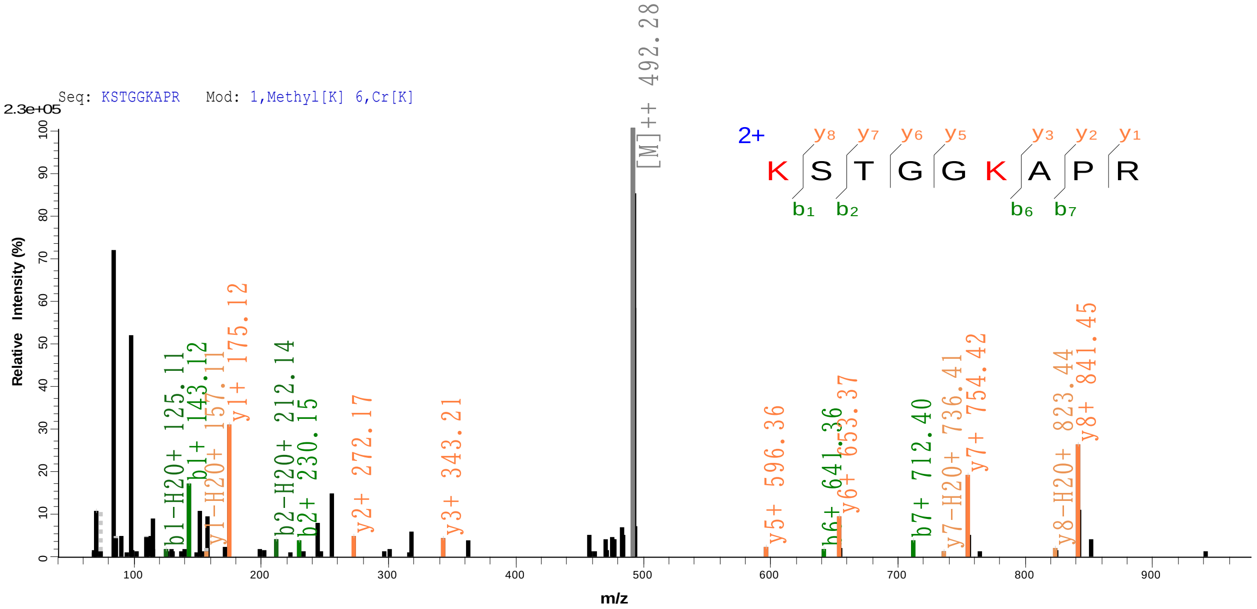

Seq: KSTGGKAPR Mod: 1,Methyl[K] 6,Cr[K]
2.3e+05
Relative Intensity (%)
m/z
[M]++ 492.28
2+
K S T G G K A P R
y8 y7 y6 y5 y3 y2 y1
b1 b2 b6 b7
b1-H2O+ 125.11
b1+ 143.12
y1-H2O+ 157.11
y1+ 175.12
b2-H2O+ 212.14
b2+ 230.15
y2+ 272.17
y3+ 343.21
y5+ 596.36
b6+ 641.36
y6+ 653.37
b7+ 712.40
y7-H2O+ 736.41
y7+ 754.42
y8-H2O+ 823.44
y8+ 841.45

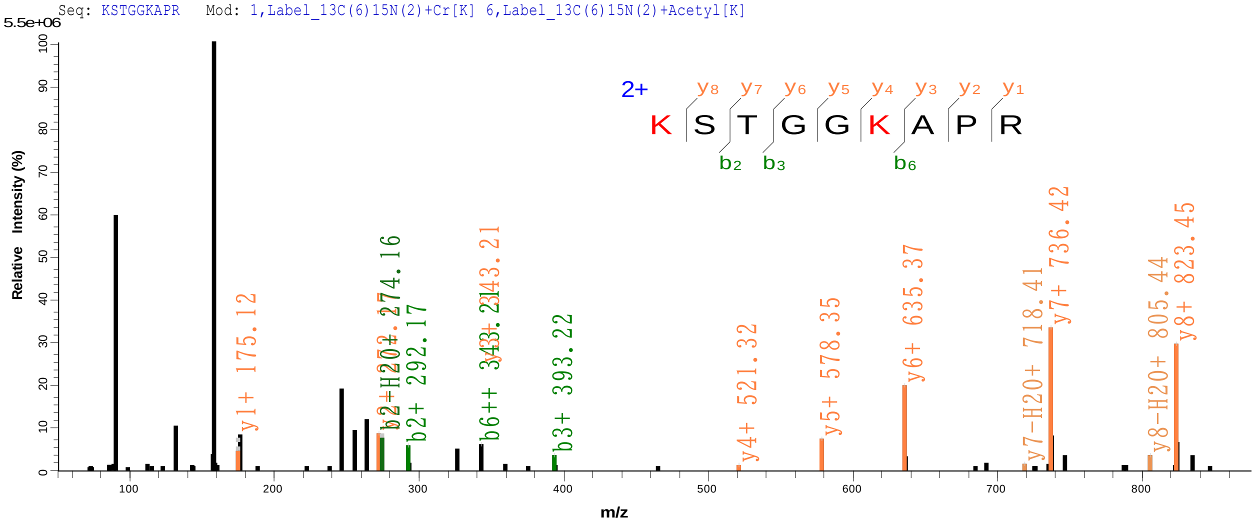

Seq: KSTGGKAPR Mod: 1,Label_13C(6)15N(2)+Cr[K] 6,Label_13C(6)15N(2)+Acetyl[K]
5.5e+06
Relative Intensity (%)
m/z
2+
K S T G G K A P R
y8 y7 y6 y5 y4 y3 y2 y1
b2 b3 b6
y1+ 175.12
b2+ 292.17
b3+ 393.22
y4+ 521.32
y5+ 578.35
y6+ 635.37
y7-H2O+ 718.41
y7+ 736.42
y8-H2O+ 805.44
y8+ 823.45

Seq: KSTGGKAPR   Mod: 1,Acetyl[K] 6,Cr[K]

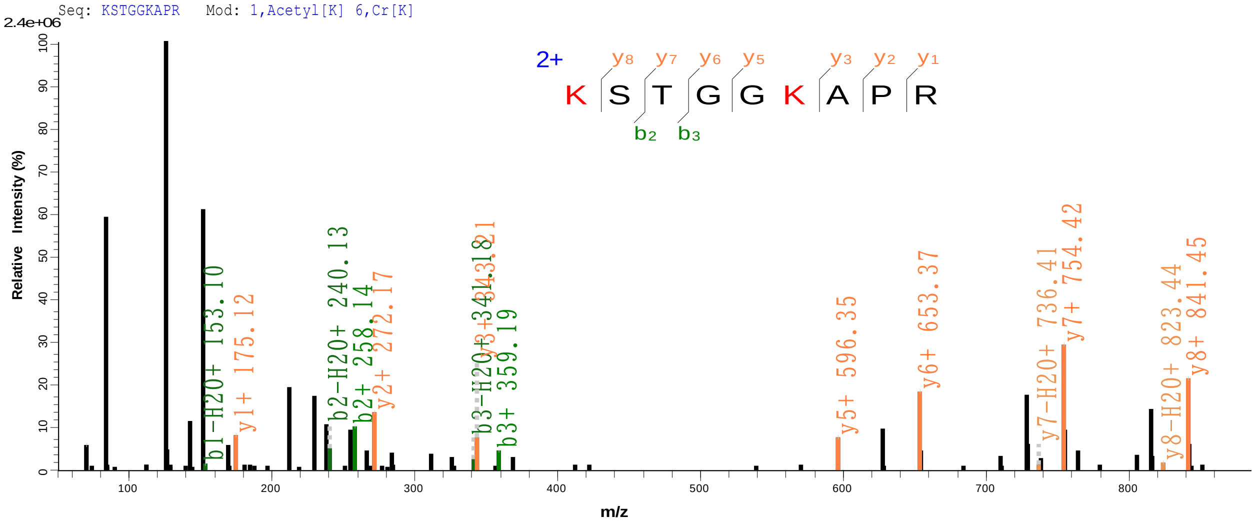

Seq: KQLATKAAR   Mod: 1,Acetyl[K] 6,Cr[K]

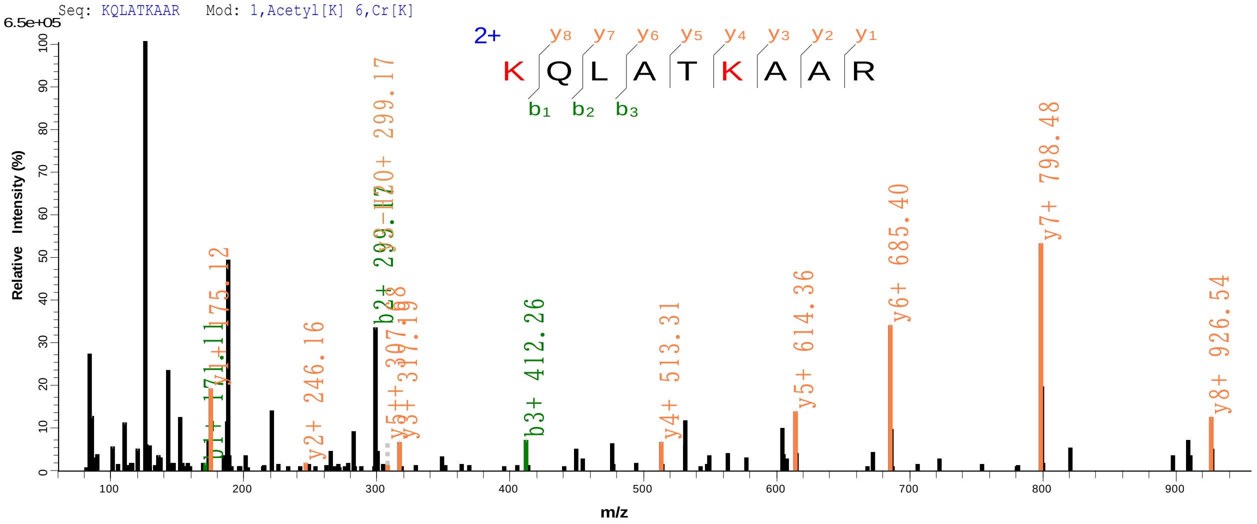

Seq: KQLATKAAR   Mod: 1,Label_13C(6)15N(2)+Cr[K] 6,Label_13C(6)15N(2)+Acetyl[K]

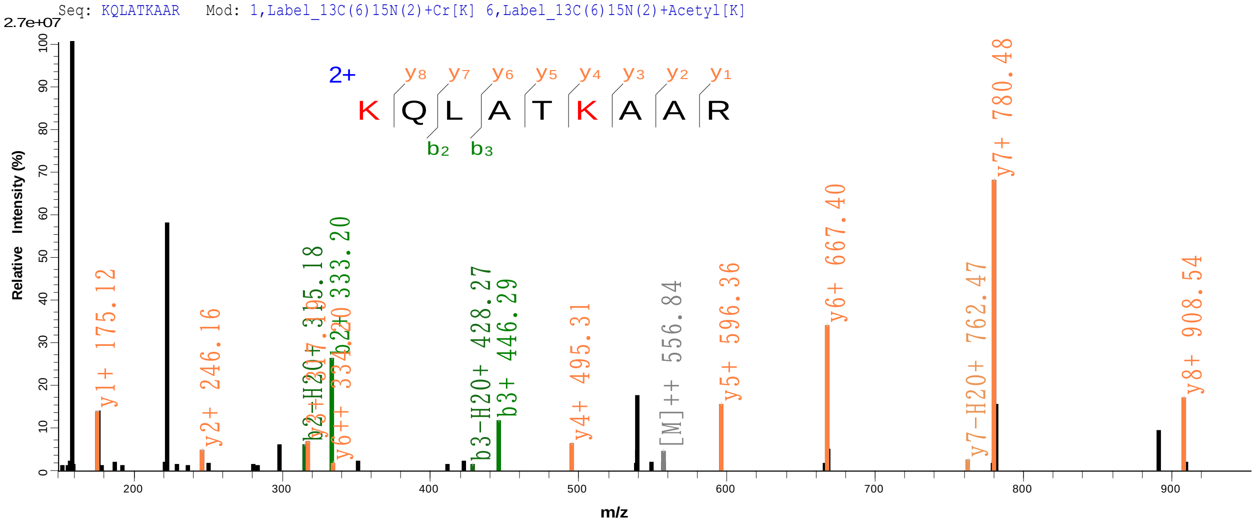

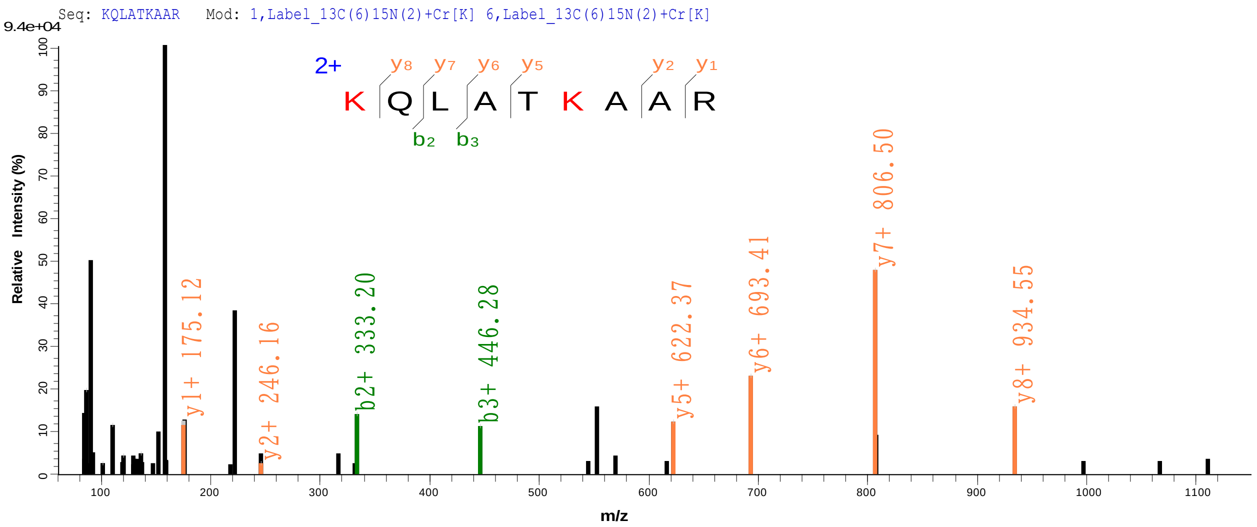

Seq: KQLATKAAR Mod: 1,Label_13C(6)15N(2)+Cr[K] 6,Label_13C(6)15N(2)+Cr[K]
9.4e+04
Relative Intensity (%)
2+
K Q L A T K A A R
y1+ 175.12
y2+ 246.16
b2+ 333.20
b3+ 446.28
y5+ 622.37
y6+ 693.41
y7+ 806.50
y8+ 934.55
m/z

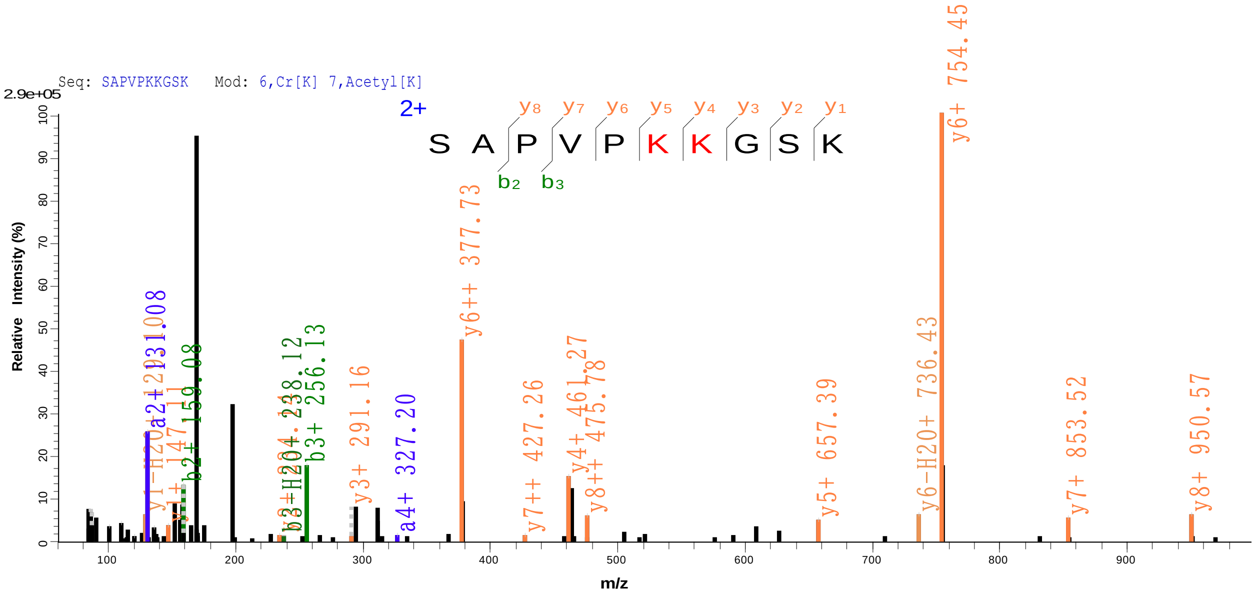

Seq: SAPVPKKGSK Mod: 6,Cr[K] 7,Acetyl[K]
2.9e+05
Relative Intensity (%)
2+
S A P V P K K G S K
b3+ 256.13
y3+ 291.16
a4+ 327.20
y6++ 377.73
y7++ 427.26
y6-H2O+ 736.43
y6+ 754.45
y5+ 657.39
y7+ 853.52
y8+ 950.57
m/z

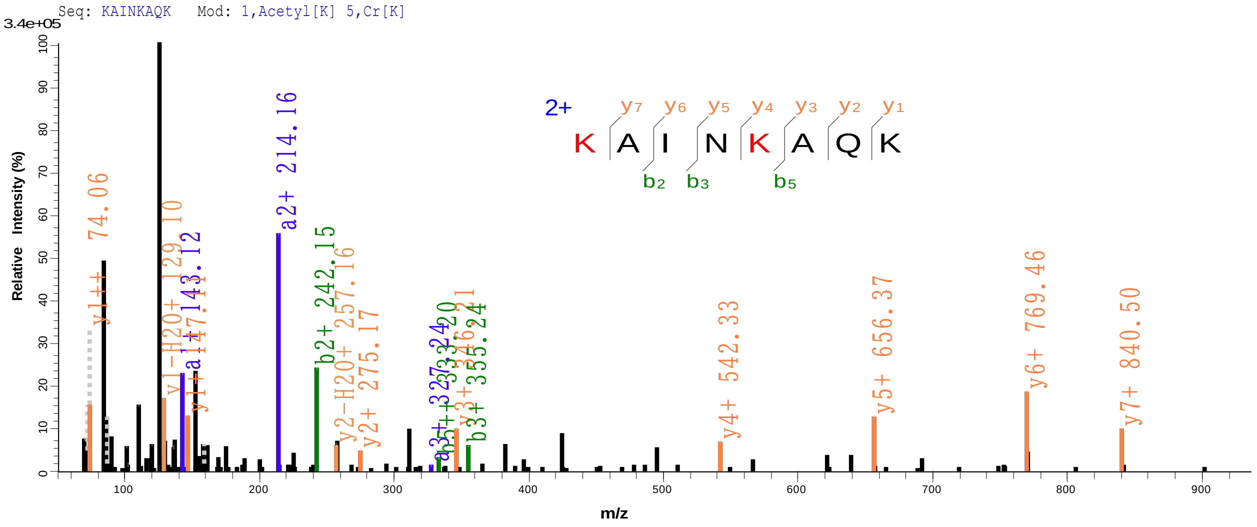

Seq: KAINKAQK Mod: 1,Acetyl[K] 5,Cr[K]
3.4e+05
Relative Intensity (%)
2+
K A I N K A Q K
y1++ 74.06
a2+ 214.16
b2+ 242.15
y2+ 275.17
b3+ 355.24
y4+ 542.33
y5+ 656.37
y6+ 769.46
y7+ 840.50
m/z

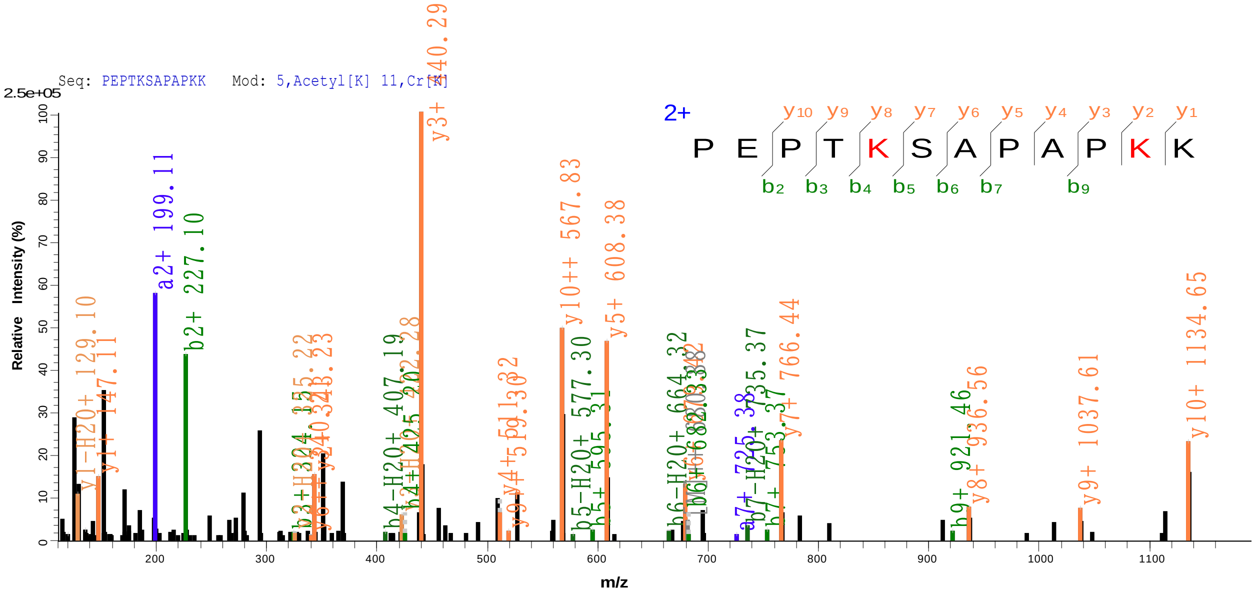
Seq: PEPTKSAPAPKK Mod: 5,Acetyl[K] 11,Cr[K]
2.5e+05
Relative Intensity (%)
m/z
2+
P E P T K S A P A P K K
y3+ 440.29
a2+ 199.11
b2+ 227.10
y10++ 567.83
y5+ 608.38
y10+ 1134.65
y9+ 1037.61
y7+ 766.44

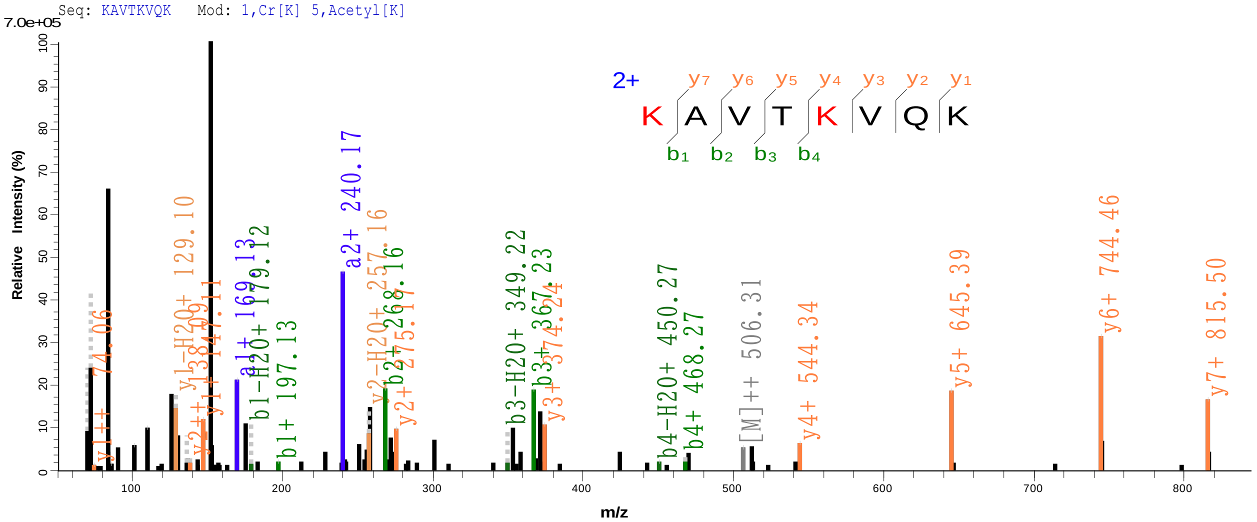
Seq: KAVTKVQK Mod: 1,Cr[K] 5,Acetyl[K]
7.0e+05
Relative Intensity (%)
m/z
2+
K A V T K V Q K
a2+ 240.17
b3-H2O+ 349.22
b4-H2O+ 450.27
b4+ 468.27
[M]++ 506.31
y4+ 544.34
y5+ 645.39
y6+ 744.46
y7+ 815.50

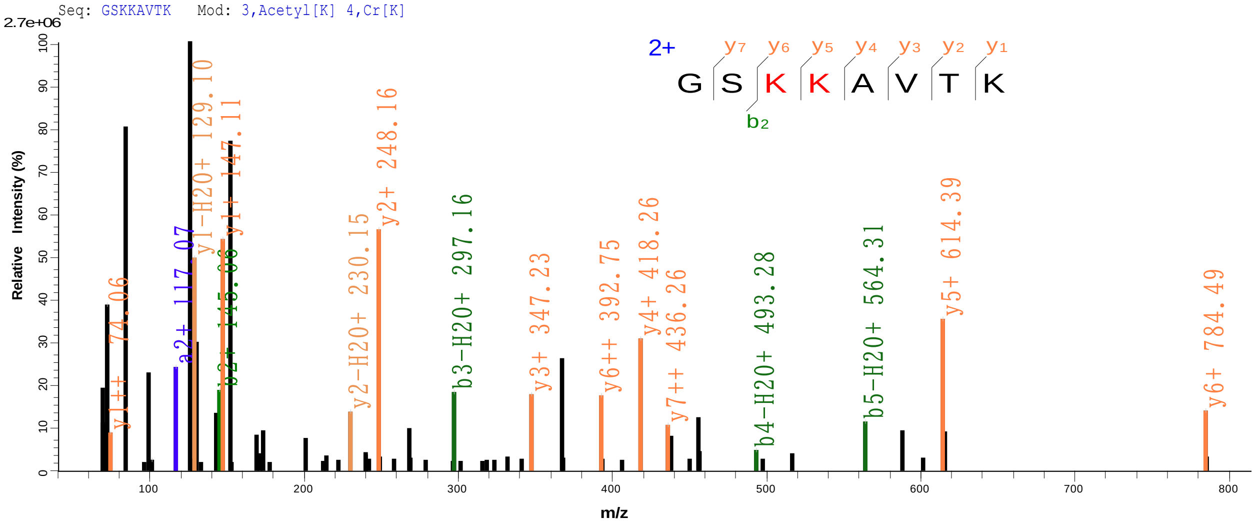
Seq: GSKKAVTK Mod: 3,Acetyl[K] 4,Cr[K]
2.7e+06
Relative Intensity (%)
m/z
2+
G S K K A V T K
y2+ 248.16
y2-H2O+ 230.15
b3-H2O+ 297.16
y3+ 347.23
y6++ 392.75
y4+ 418.26
y7++ 436.26
b4-H2O+ 493.28
b5-H2O+ 564.31
y5+ 614.39
y6+ 784.49

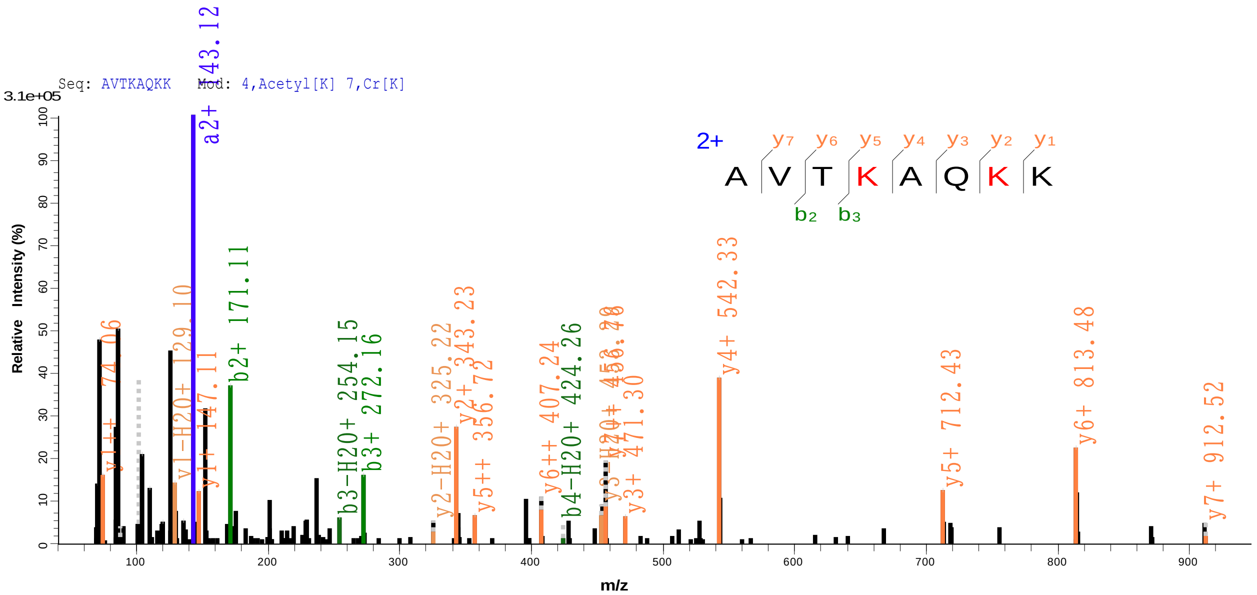
Seq: AVTKAQKK   Mod: 4,Acetyl[K] 7,Cr[K]
3.1e+05
Relative Intensity (%)
m/z
2+
A V T K A Q K K
y7 y6 y5 y4 y3 y2 y1
b2 b3
a2+ 143.12
b2+ 171.11
b3-H2O+ 254.15
b3+ 272.16
y2-H2O+ 325.22
y2+ 343.23
y5++ 356.72
y6++ 407.24
b4-H2O+ 424.26
y3+ 471.30
y4+ 542.33
y5+ 712.43
y6+ 813.48
y7+ 912.52

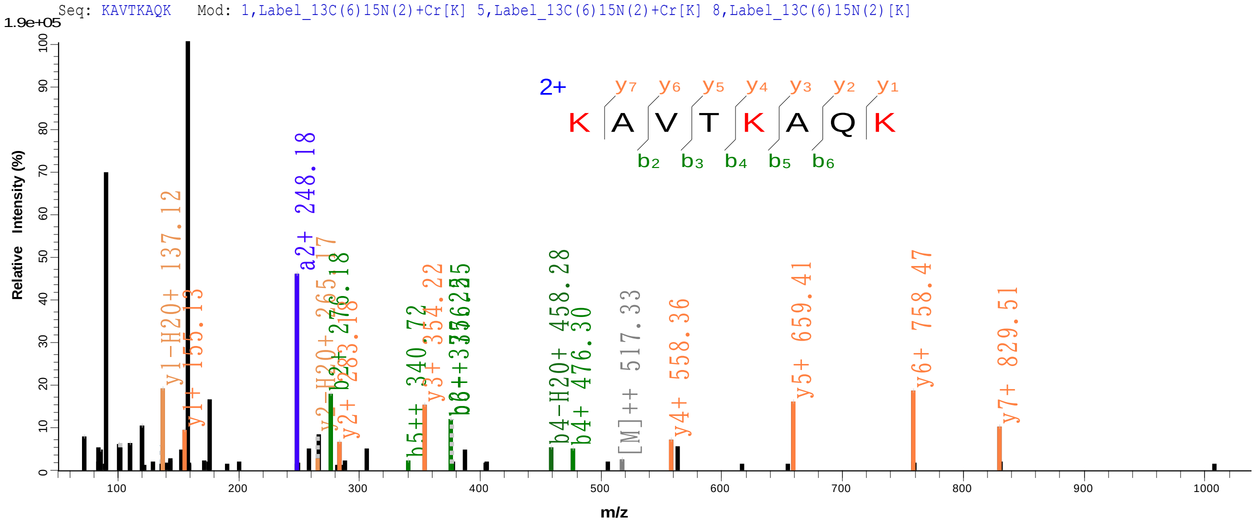
Seq: KAVTKAQK   Mod: 1,Label_13C(6)15N(2)+Cr[K] 5,Label_13C(6)15N(2)+Cr[K] 8,Label_13C(6)15N(2)[K]
1.9e+05
Relative Intensity (%)
m/z
2+
K A V T K A Q K
y7 y6 y5 y4 y3 y2 y1
b2 b3 b4 b5 b6
y1-H2O+ 137.12
y1+ 155.13
a2+ 248.18
b2+ 276.18
y2+ 283.18
b5++ 340.72
y3+ 354.22
b4-H2O+ 458.28
b4+ 476.30
[M]++ 517.33
y4+ 558.36
y5+ 659.41
y6+ 758.47
y7+ 829.51

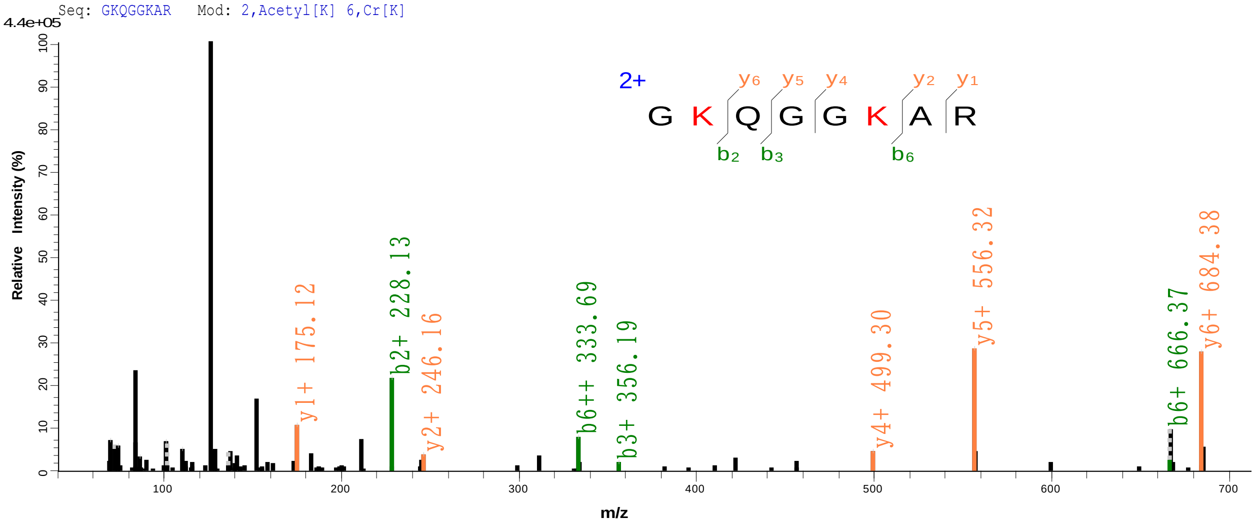
Seq: GKQGGKAR   Mod: 2,Acetyl[K] 6,Cr[K]
4.4e+05
Relative Intensity (%)
m/z
2+
G K Q G G K A R
y6 y5 y4 y2 y1
b2 b3 b6
y1+ 175.12
b2+ 228.13
y2+ 246.16
b6++ 333.69
b3+ 356.19
y4+ 499.30
y5+ 556.32
b6+ 666.37
y6+ 684.38

Seq: GKQGGKAR   Mod: 2,Cr[K] 6,Acetyl[K]

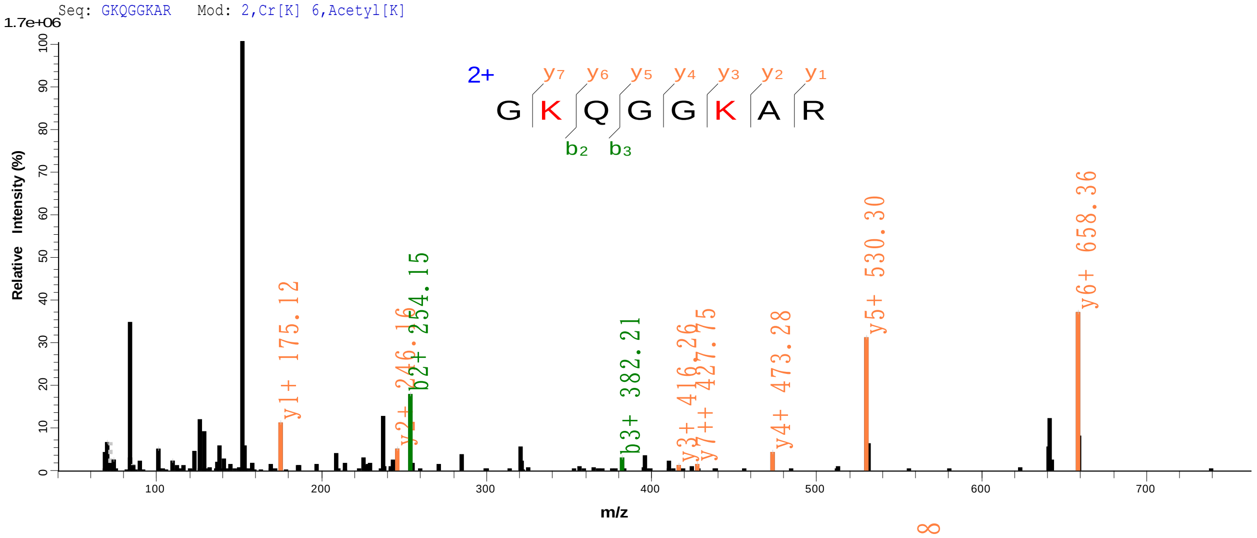

Seq: AGGKAGKDSGK   Mod: 4,Label_13C(6)15N(2)+Cr[K] 7,Label_13C(6)15N(2)+Acetyl[K] 11,Label_13C(6)15N(2)[K]

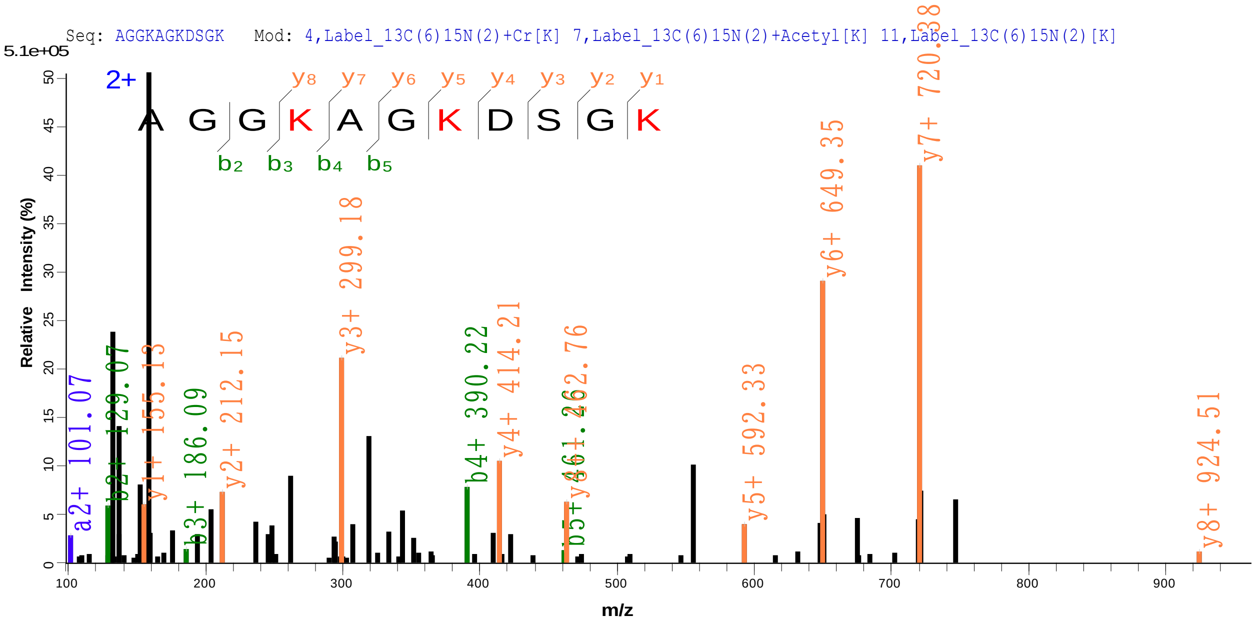

Seq: AGKDSGKAK   Mod: 3,Label_13C(6)15N(2)+Acetyl[K] 7,Label_13C(6)15N(2)+Cr[K] 9,Label_13C(6)15N(2)[K]

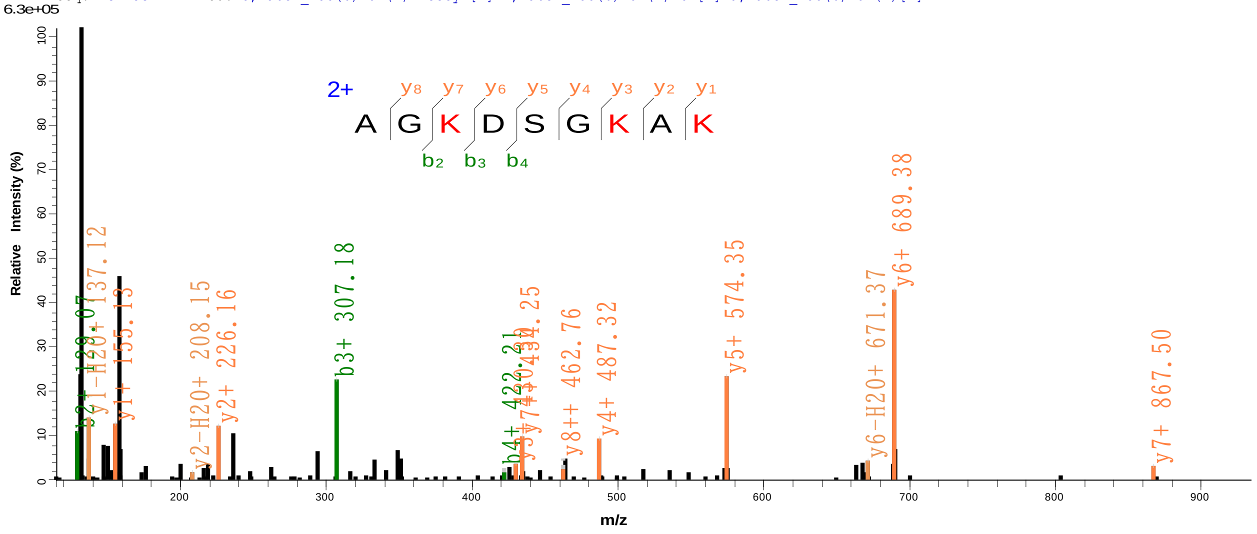

Seq: DSGKAKTK Mod: 4,Acetyl[K] 6,Crotonyl[K]

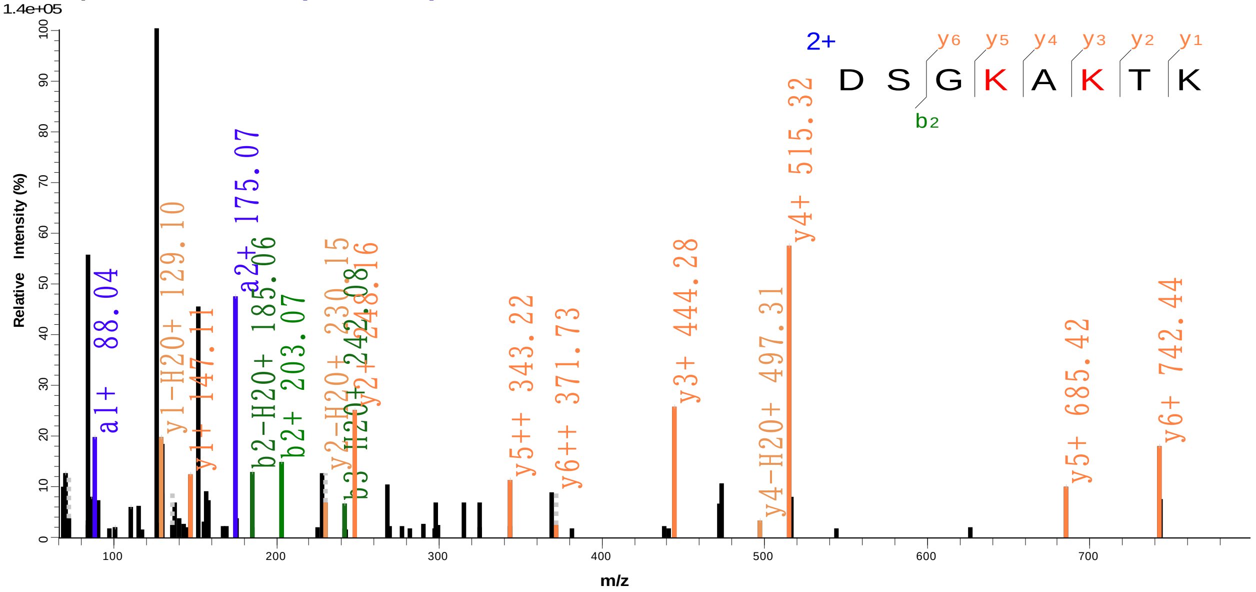

Seq: KSTGGKAPR Mod: 1,Benzoyl[K] 6,Acetyl[K]

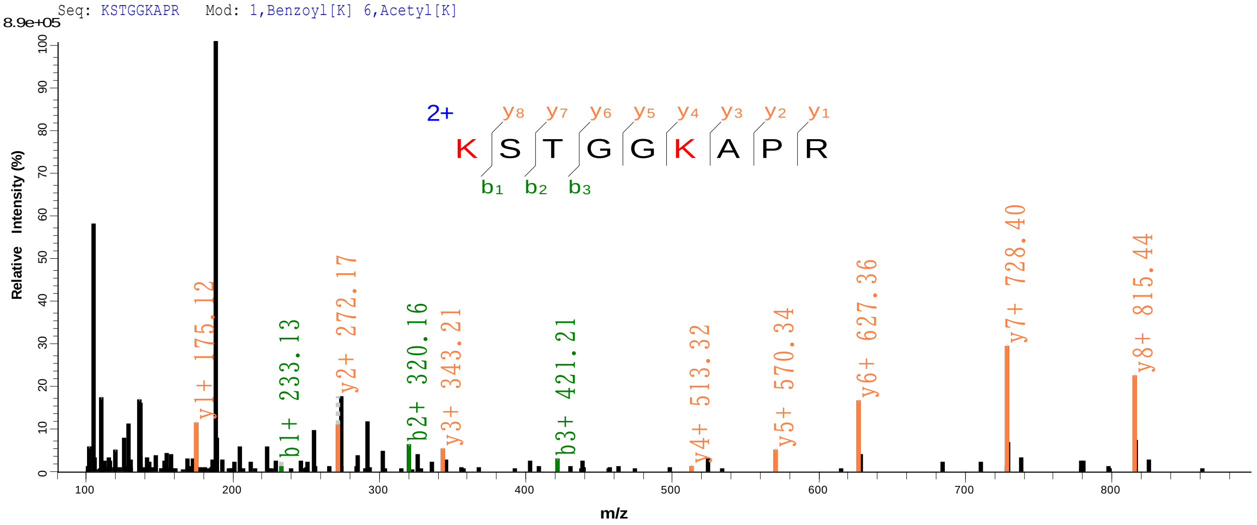

Seq: KQLATKAAR Mod: 1,Benzoyl[K] 6,Acetyl[K]

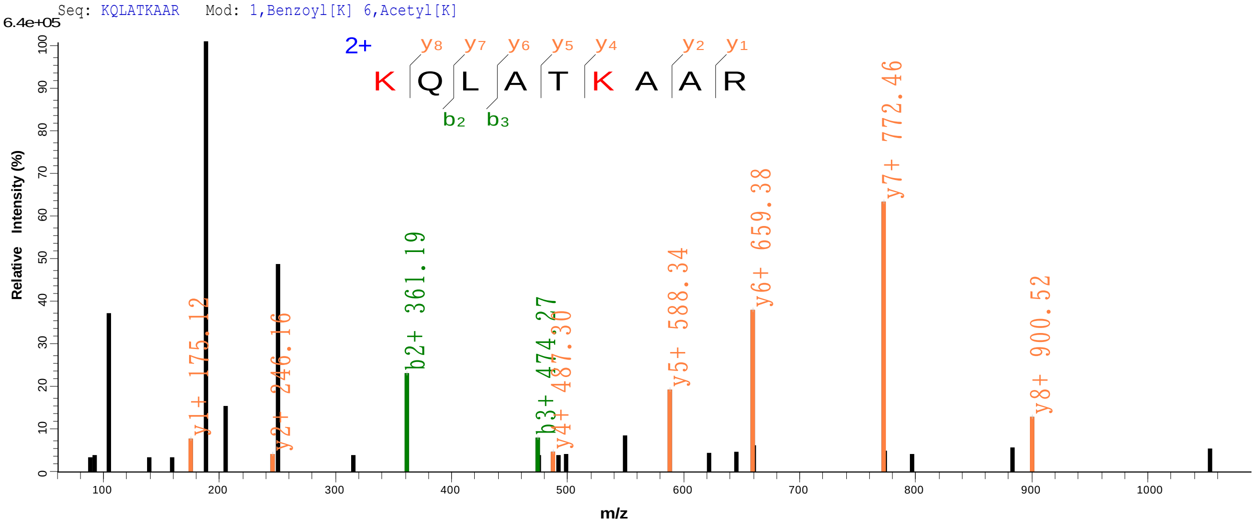

Data S6. PSMs for all newly identified histone marks.

A. Kla

H1

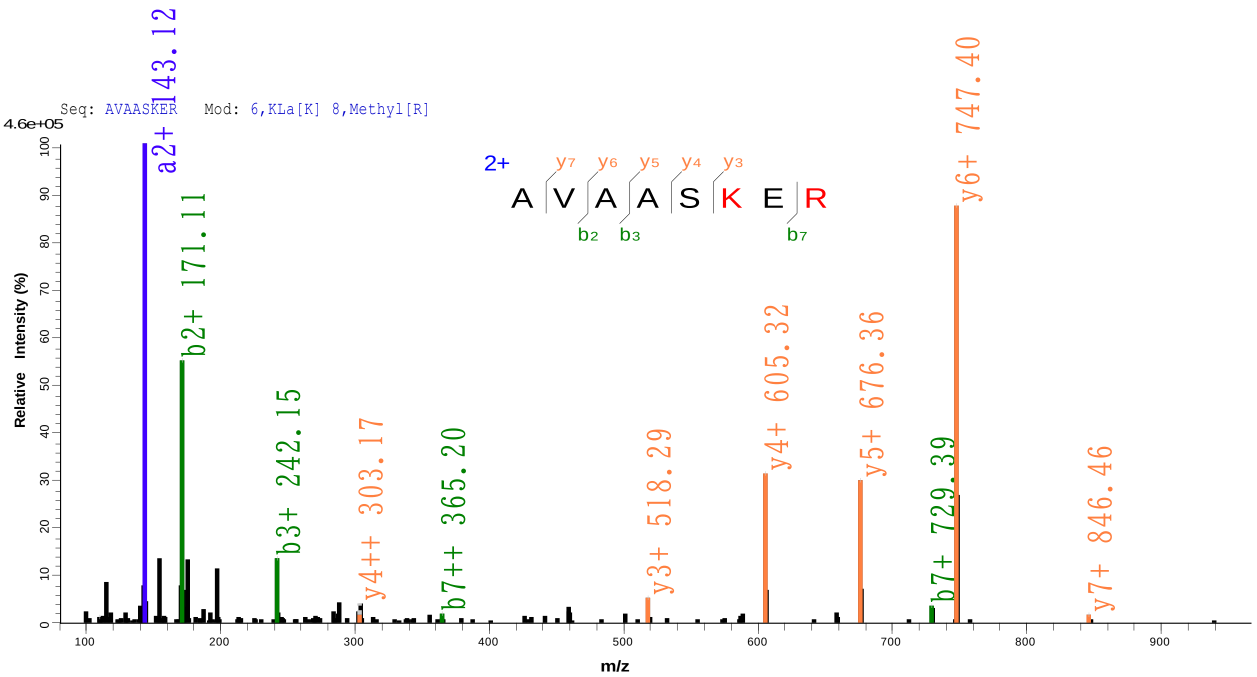

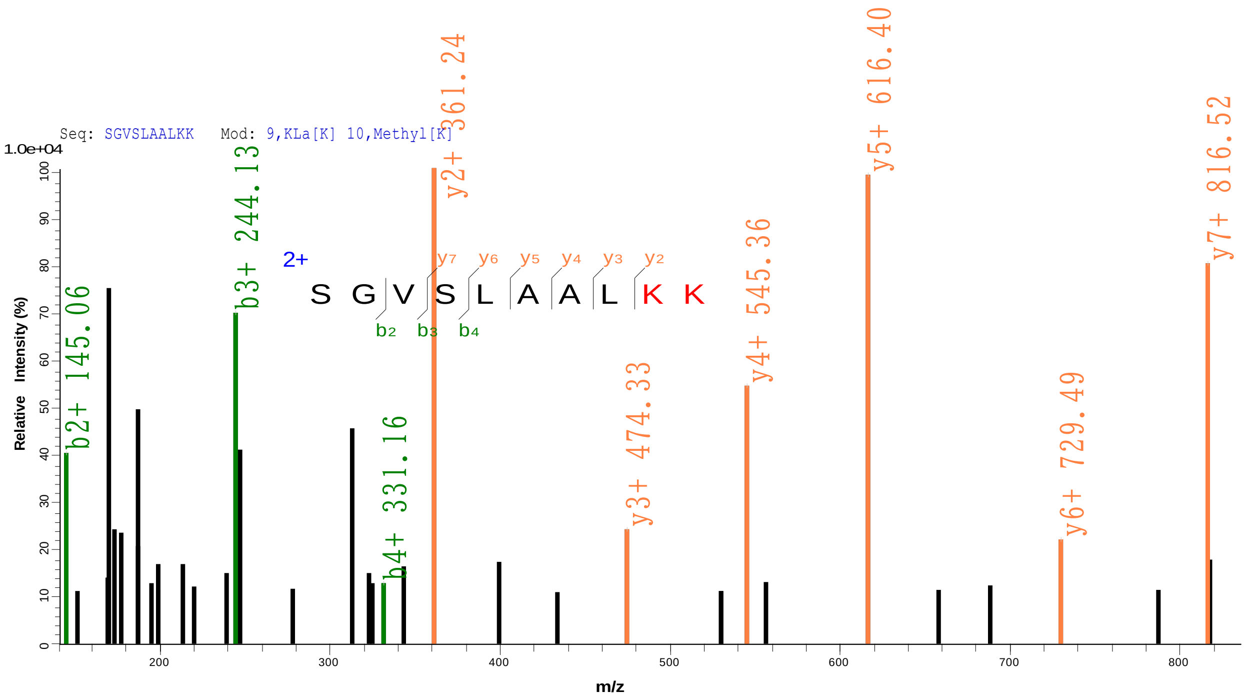

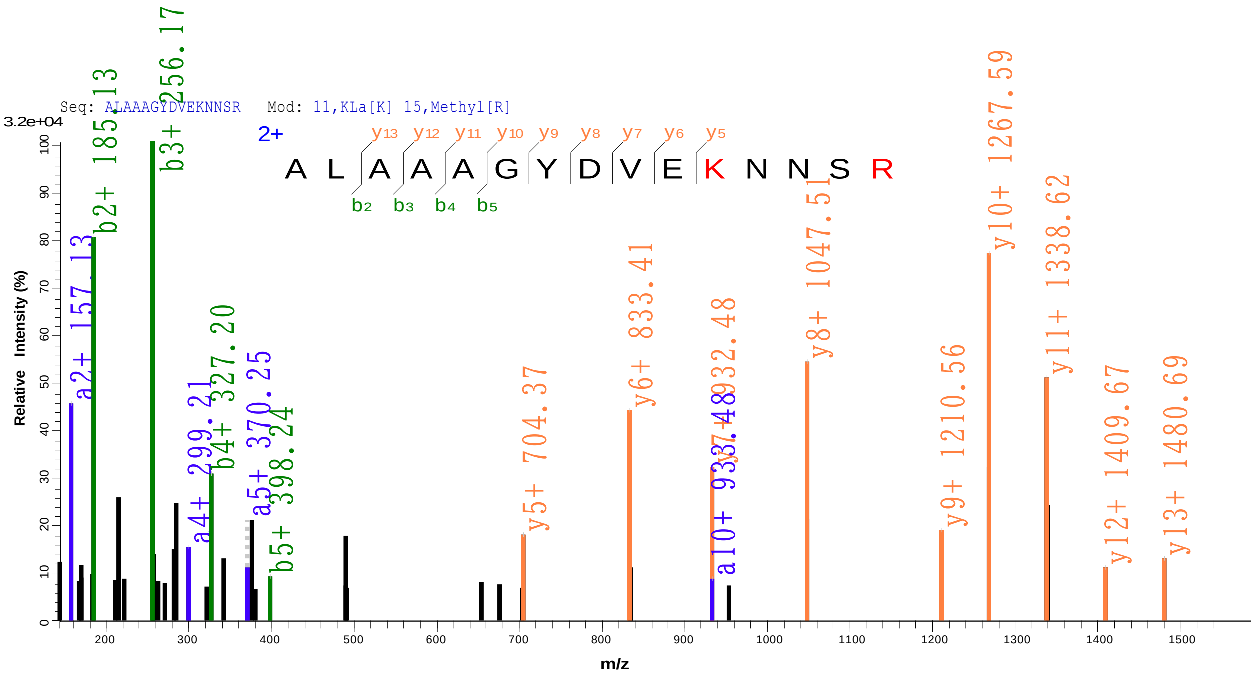
Seq: ALAAAGYDVEKNNSR Mod: 11,KLa[K] 15,Methyl[R]
3.2e+04
2+
A L A A A G Y D V E K N N S R
Relative Intensity (%)
m/z
a2+ 157.13
b2+ 185.13
b3+ 256.17
a4+ 299.21
b4+ 327.20
a5+ 370.25
b5+ 398.24
y5+ 704.37
y6+ 833.41
a10+ 933.48
y8+ 1047.51
y9+ 1210.56
y10+ 1267.59
y11+ 1338.62
y12+ 1409.67
y13+ 1480.69

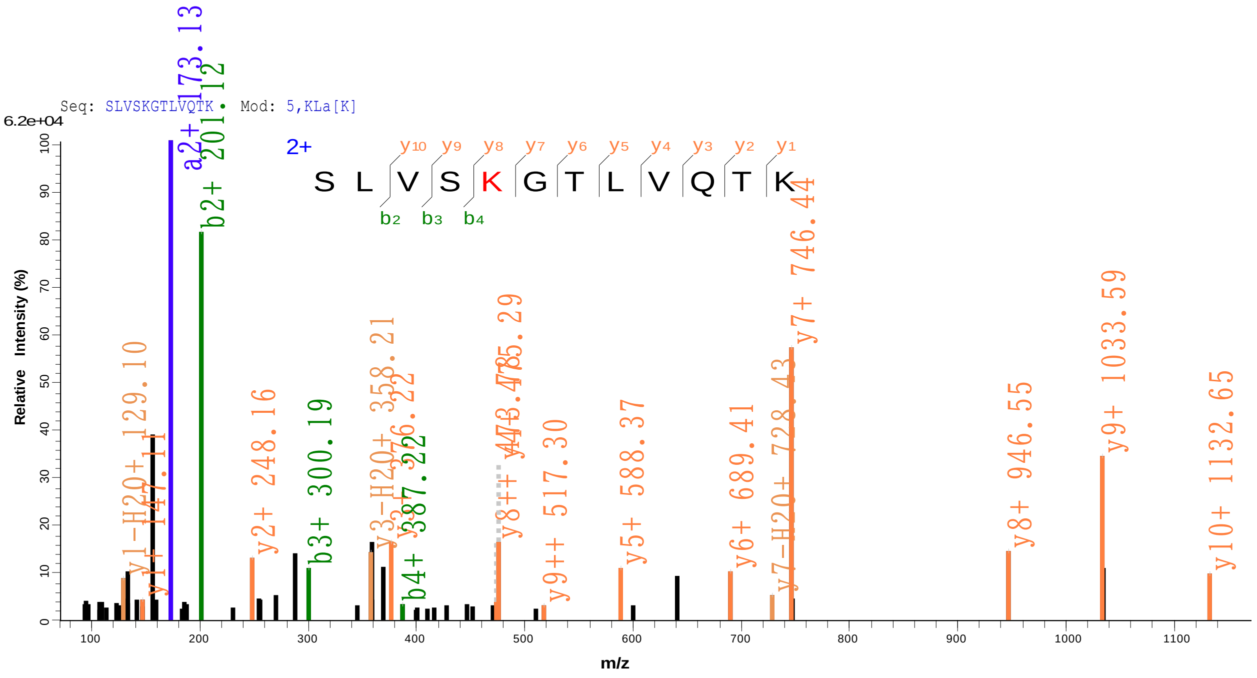
Seq: SLVSKGTLVQTK Mod: 5,KLa[K]
6.2e+04
2+
S L V S K G T L V Q T K
Relative Intensity (%)
m/z
a2+ 173.13
b2+ 201.12
y1-H2O+ 129.10
y1+ 147.11
y2+ 248.16
b3+ 300.19
y3-H2O+ 358.21
b4+ 387.22
y9++ 517.30
y5+ 588.37
y6+ 689.41
y7-H2O+ 728.43
y7+ 746.44
y8+ 946.55
y9+ 1033.59
y10+ 1132.65

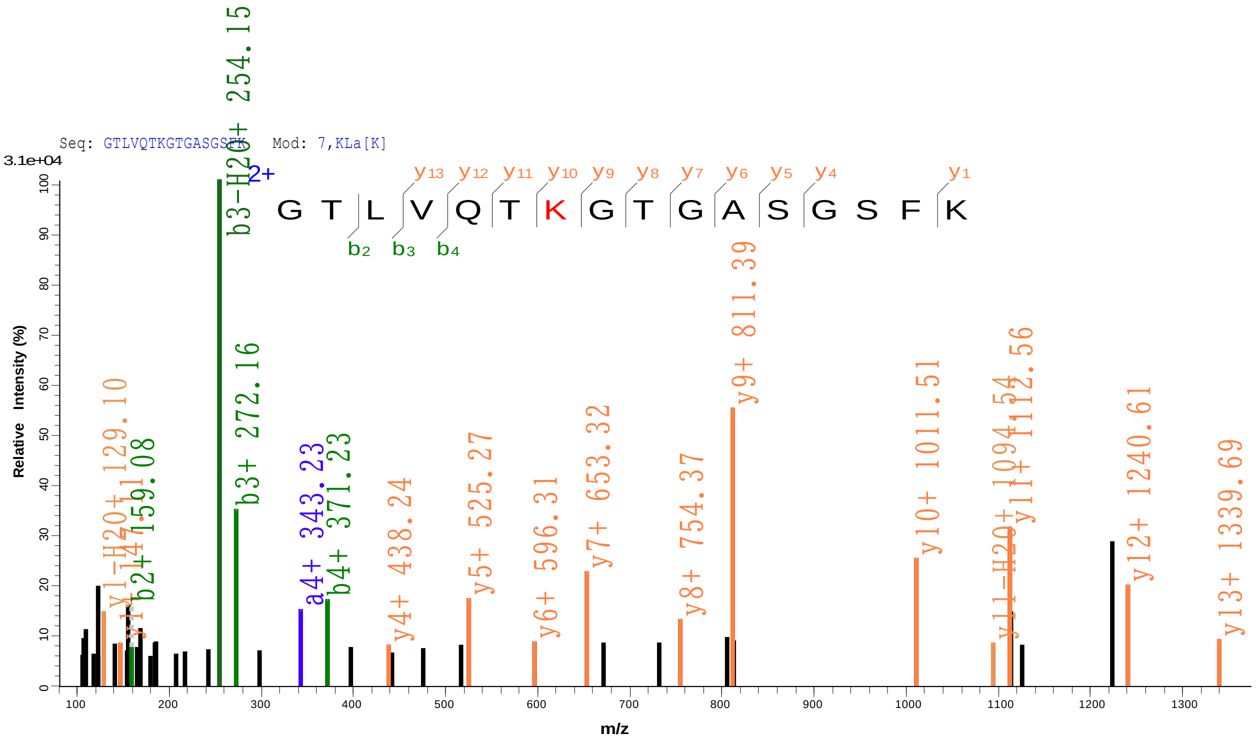

Seq: GTLVQTKGTGASGSFK Mod: 7,KLa[K]
3.1e+04
2+
G T L V Q T K G T G A S G S F K
Relative Intensity (%)
m/z

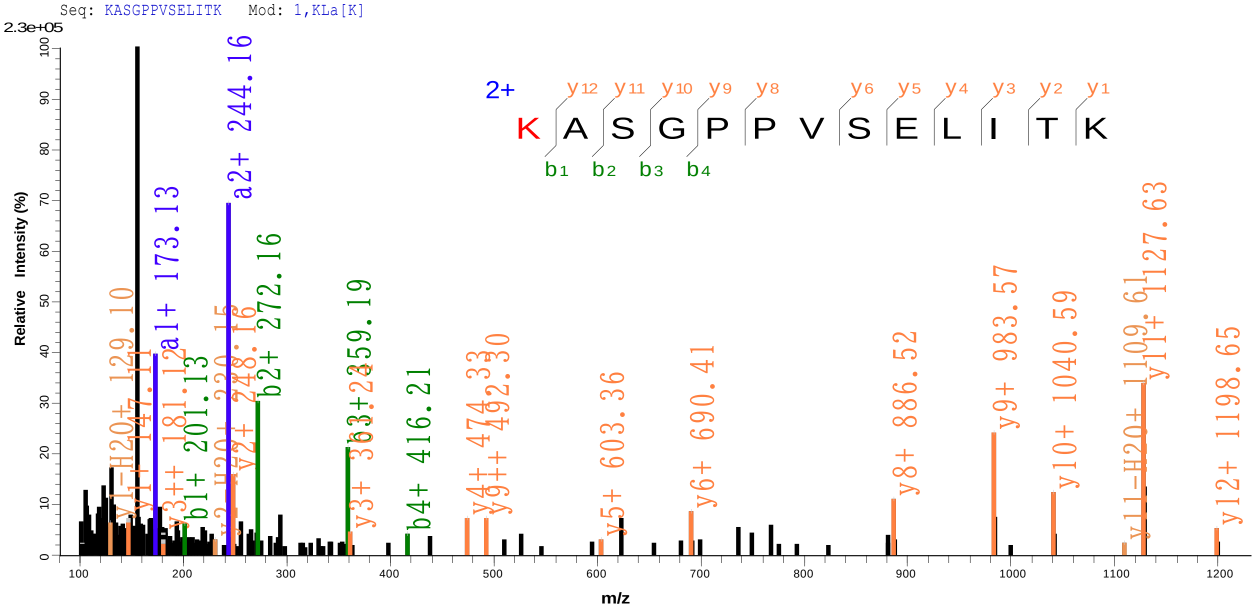

Seq: KASGPPVSELITK Mod: 1,KLa[K]
2.3e+05
2+
K A S G P P V S E L I T K
Relative Intensity (%)
m/z

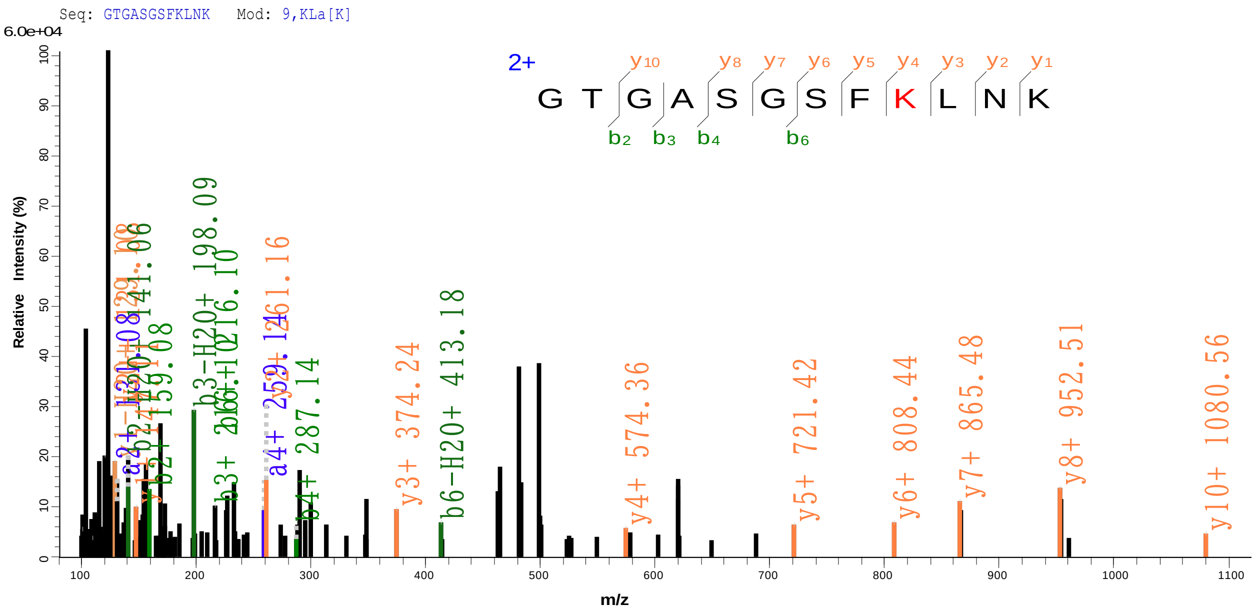
Seq: GTGASGSFKLNK Mod: 9,KLa[K]
6.0e+04
2+
G T G A S G S F K L N K
y10 y8 y7 y6 y5 y4 y3 y2 y1
b2 b3 b4 b6
Relative Intensity (%)
m/z
b3-H2O+ 198.09
a4+ 259.14
b4+ 287.14
y3+ 374.24
b6-H2O+ 413.18
y4+ 574.36
y5+ 721.42
y6+ 808.44
y7+ 865.48
y8+ 952.51
y10+ 1080.56

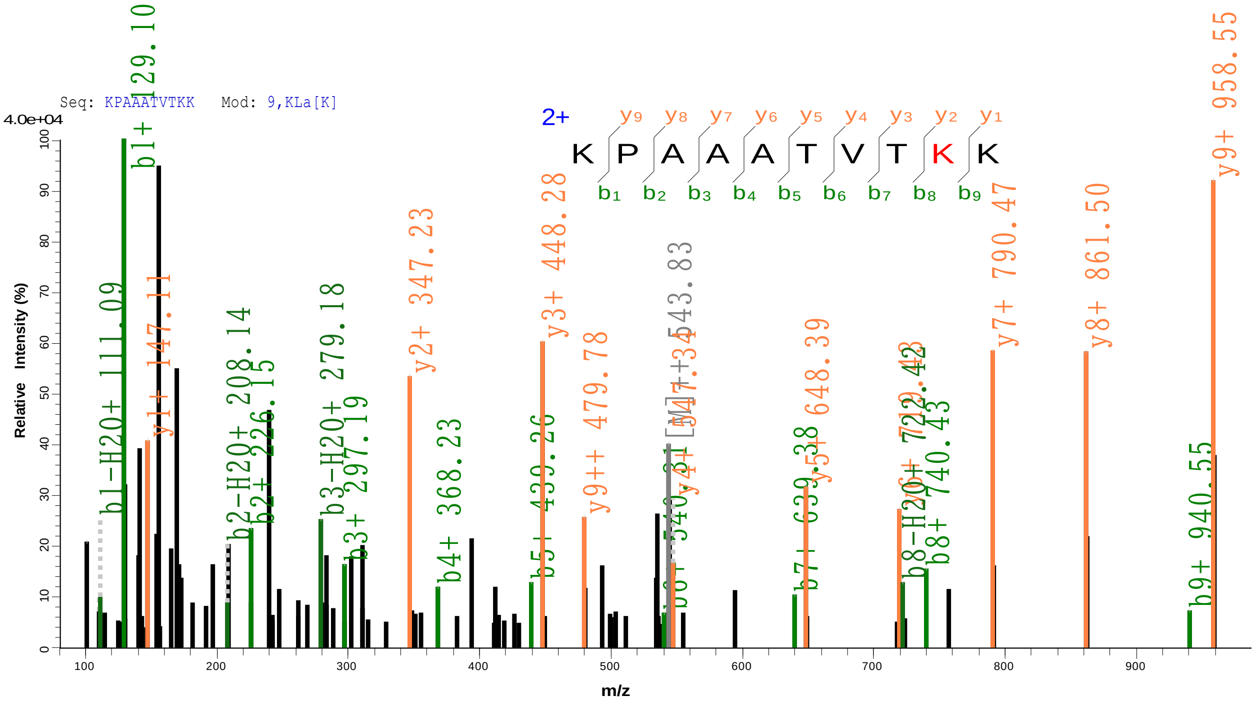
Seq: KPAAATVTKK Mod: 9,KLa[K]
4.0e+04
2+
K P A A A T V T K K
y9 y8 y7 y6 y5 y4 y3 y2 y1
b1 b2 b3 b4 b5 b6 b7 b8 b9
Relative Intensity (%)
m/z
b1+ 129.10
b1-H2O+ 111.09
y1+ 147.11
b2-H2O+ 208.14
b2+ 226.15
b3-H2O+ 279.18
b3+ 297.19
y2+ 347.23
b4+ 368.23
y3+ 448.28
y9++ 479.78
y7+ 790.47
y8+ 861.50
b8+ 740.43
b9+ 940.55
y9+ 958.55

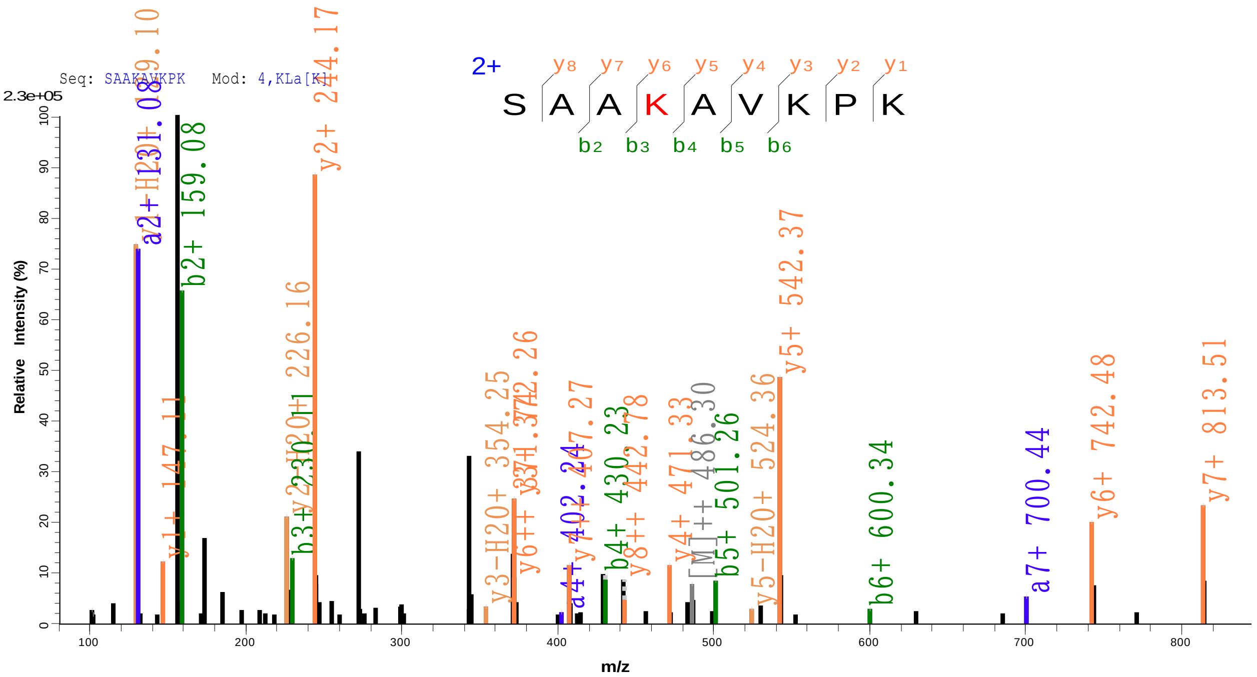
Seq: SAAKAVKPK Mod: 4,KLa[K]
2.3e+05
2+
y8 y7 y6 y5 y4 y3 y2 y1
S A A K A V K P K
b2 b3 b4 b5 b6
Relative Intensity (%)
m/z
a2+ 131.08
b2+ 159.08
y1+ 147.11
y2+ 244.17
b3+ 230.1
y3-H2O+ 354.25
a4+ 402.24
b4+ 430.23
b5+ 501.26
[M]++ 486.30
y5-H2O+ 524.36
y5+ 542.37
b6+ 600.34
a7+ 700.44
y6+ 742.48
y7+ 813.51
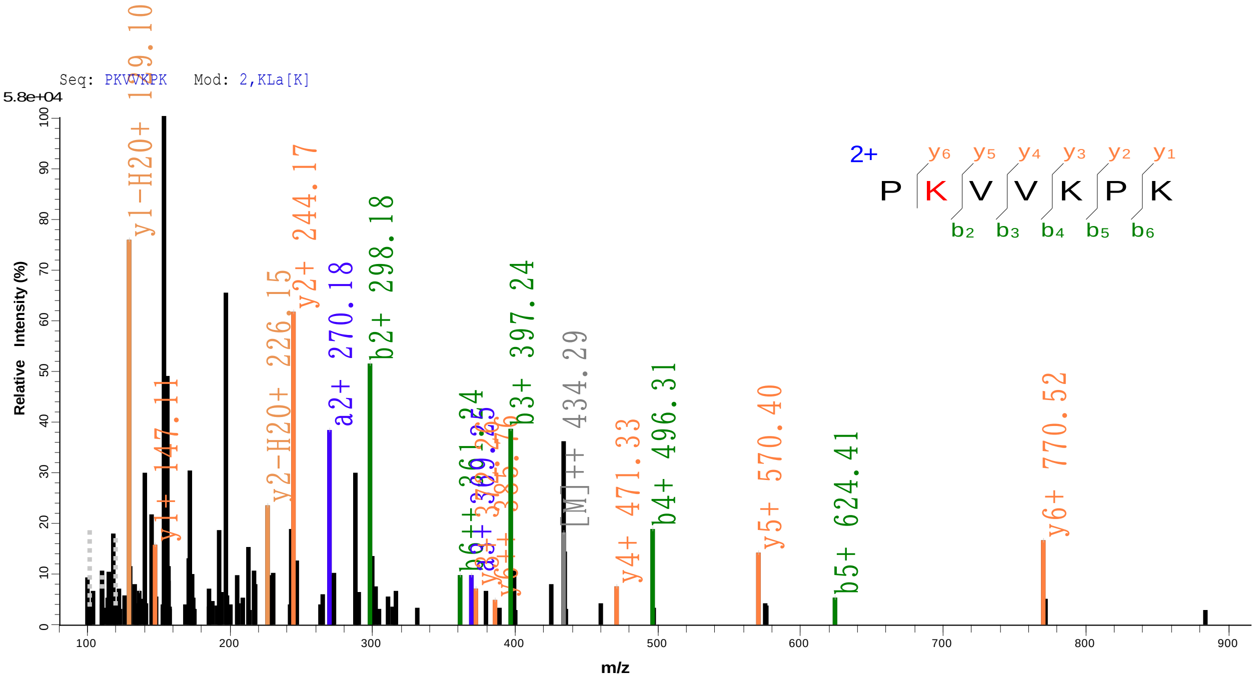
Seq: PKVVKPK Mod: 2,KLa[K]
5.8e+04
2+
y6 y5 y4 y3 y2 y1
P K V V K P K
b2 b3 b4 b5 b6
Relative Intensity (%)
m/z
y1-H2O+ 129.10
y1+ 147.11
y2-H2O+ 226.15
y2+ 244.17
a2+ 270.18
b2+ 298.18
b3+ 397.24
[M]++ 434.29
y4+ 471.33
b4+ 496.31
y5+ 570.40
b5+ 624.41
y6+ 770.52

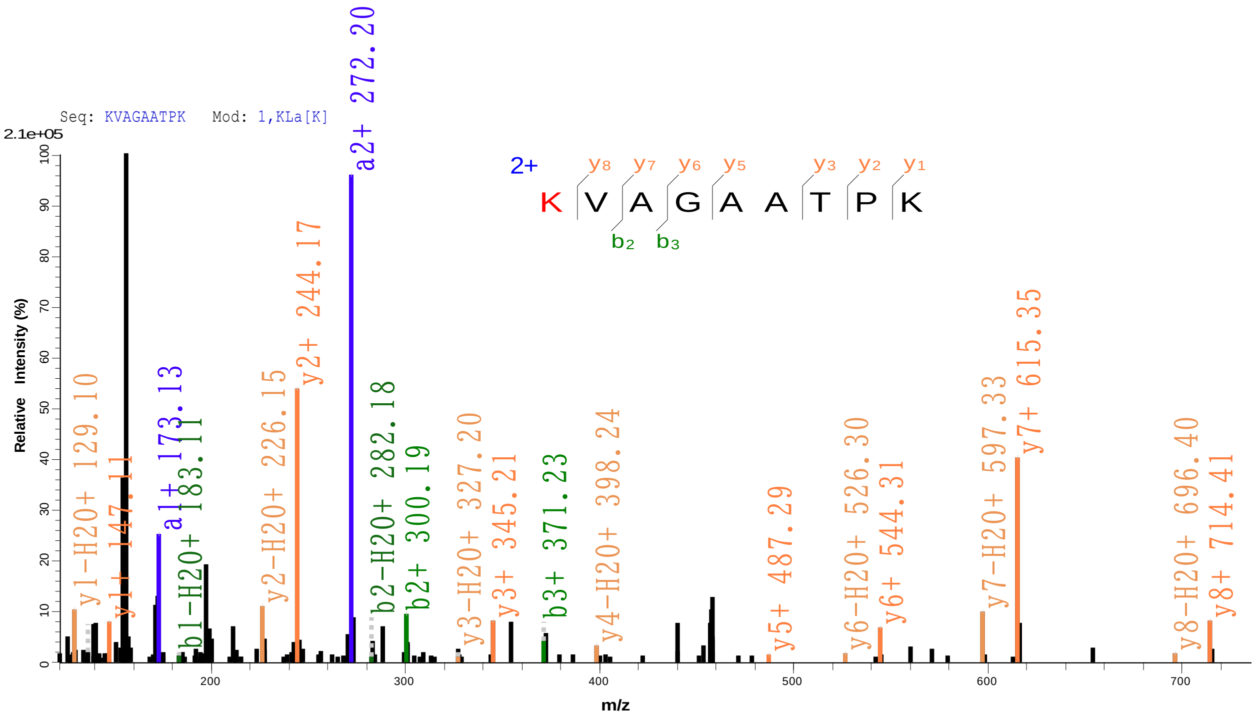
Seq: KVAGAATPK Mod: 1,KLa[K]
2.1e+05
Relative Intensity (%)
m/z
2+
K V A G A A T P K
y8 y7 y6 y5 y3 y2 y1
b2 b3
y1-H2O+ 129.10
y1+ 147.11
a1+ 173.13
b1-H2O+ 183.11
y2-H2O+ 226.15
y2+ 244.17
a2+ 272.20
b2-H2O+ 282.18
b2+ 300.19
y3-H2O+ 327.20
y3+ 345.21
b3+ 371.23
y4-H2O+ 398.24
y5+ 487.29
y6-H2O+ 526.30
y6+ 544.31
y7-H2O+ 597.33
y7+ 615.35
y8-H2O+ 696.40
y8+ 714.41

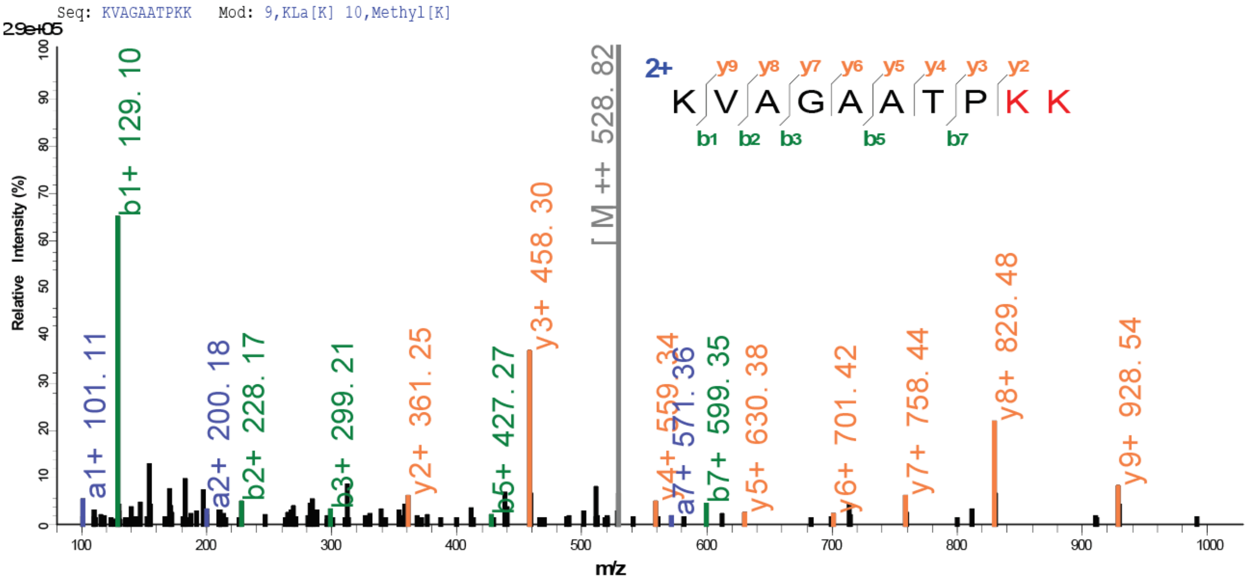
Seq: KVAGAATPKK Mod: 9,KLa[K] 10,Methyl[K]
2.9e+05
Relative Intensity (%)
m/z
2+
K V A G A A T P K K
y9 y8 y7 y6 y5 y4 y3 y2
b1 b2 b3 b5 b7
a1+ 101.11
b1+ 129.10
a2+ 200.18
b2+ 228.17
b3+ 299.21
y2+ 361.25
b5+ 427.27
y3+ 458.30
[M]++ 528.82
y4+ 559.34
a7+ 571.36
b7+ 599.35
y5+ 630.38
y6+ 701.42
y7+ 758.44
y8+ 829.48
y9+ 928.54

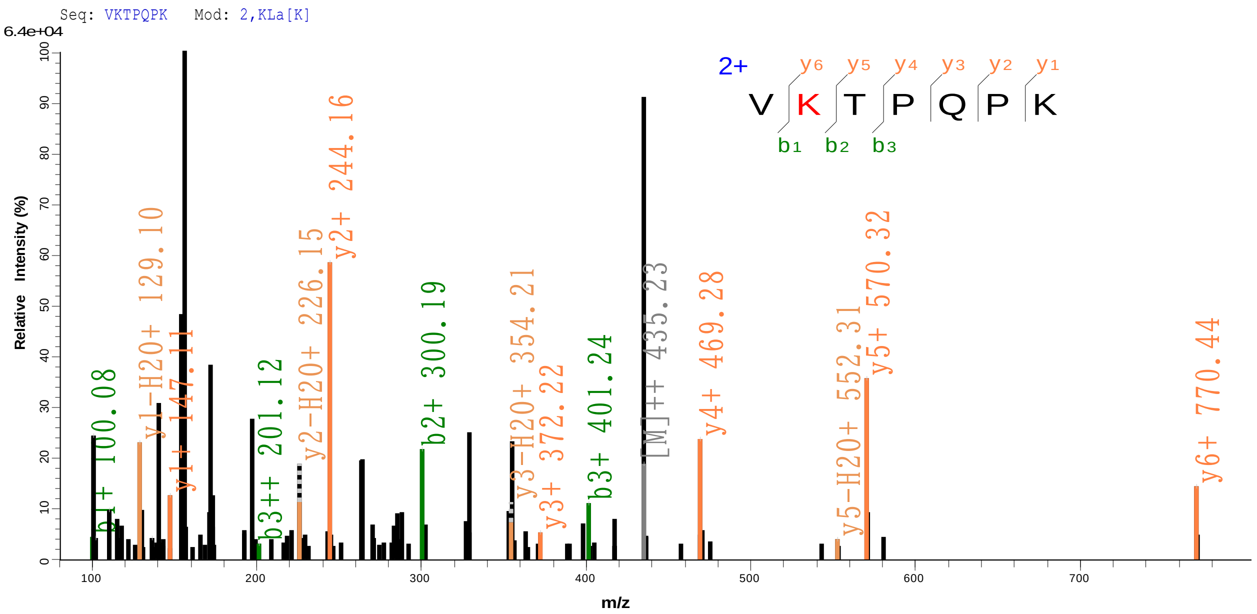

Seq: VKTPQPK Mod: 2,KLa[K]
6.4e+04
Relative Intensity (%)
m/z
2+
V K T P Q P K
y6 y5 y4 y3 y2 y1
b1 b2 b3
b1+ 100.08
y1-H2O+ 129.10
y1+ 147.11
b3++ 201.12
y2-H2O+ 226.15
y2+ 244.16
b2+ 300.19
y3-H2O+ 354.21
y3+ 372.22
b3+ 401.24
[M]++ 435.23
y4+ 469.28
y5-H2O+ 552.31
y5+ 570.32
y6+ 770.44

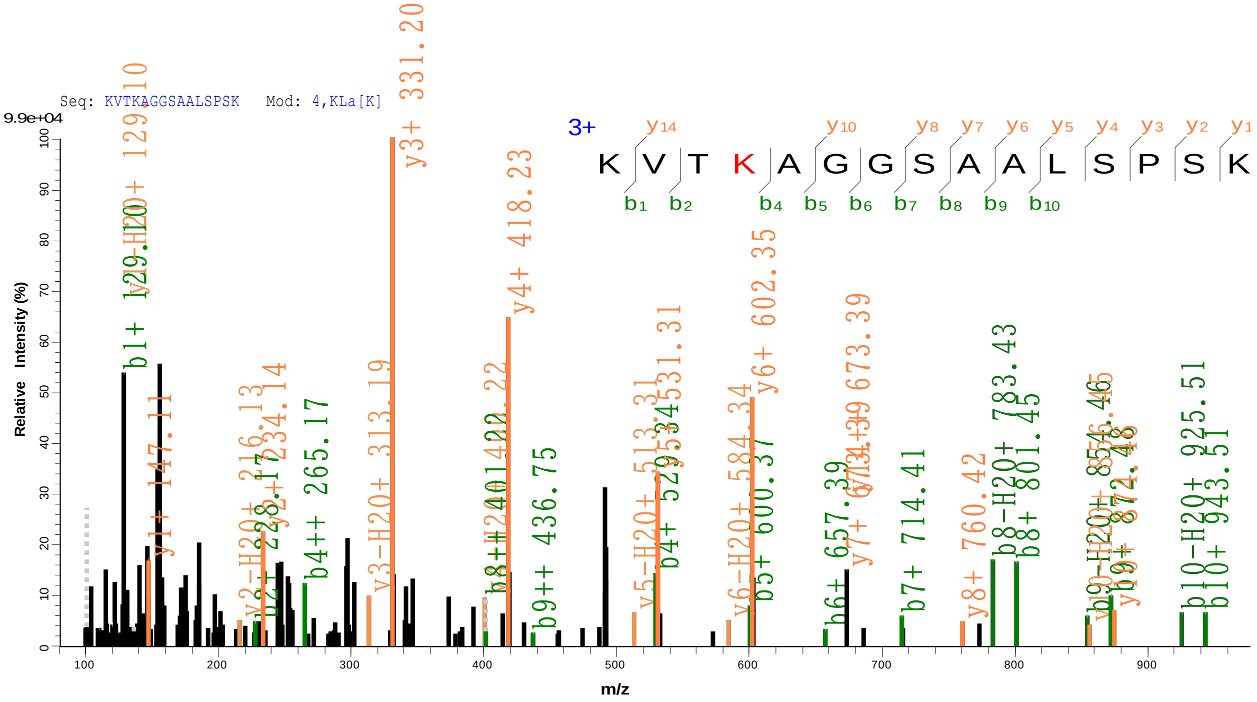

Seq: KVTKAGGSAALSPSK Mod: 4,KLa[K]
9.9e+04
Relative Intensity (%)
m/z
3+
K V T K A G G S A A L S P S K
y14 y10 y8 y7 y6 y5 y4 y3 y2 y1
b1 b2 b4 b5 b6 b7 b8 b9 b10
b1+ 129.10
y1+ 147.11
y2-H2O+ 216.13
y2+ 234.14
b4++ 265.17
y3-H2O+ 313.19
y3+ 331.20
y4+ 418.23
b9++ 436.75
y5-H2O+ 513.31
b4+ 529.34
y6-H2O+ 584.34
b5+ 600.37
y6+ 602.35
b6+ 657.39
y7+ 673.39
b7+ 714.41
y8+ 760.42
b8-H2O+ 783.43
b8+ 801.45
b10-H2O+ 925.51
b10+ 943.51

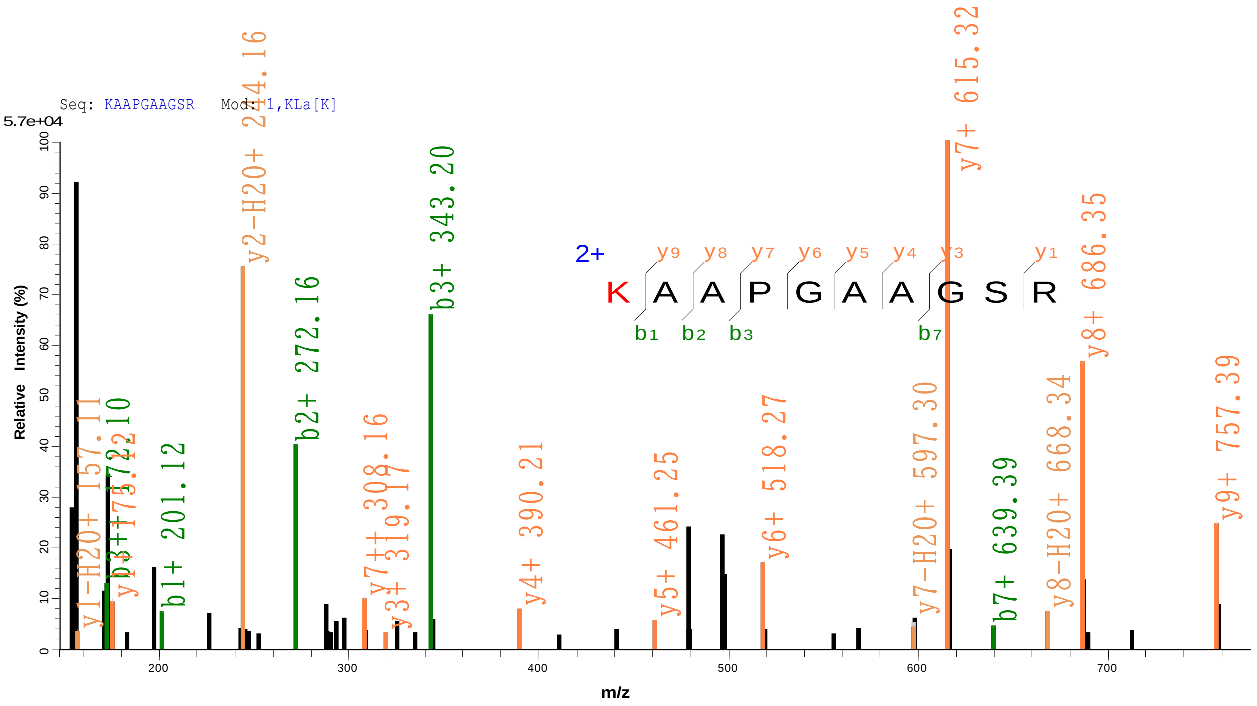
Seq: KAAPGAAGSR Mod: 1,KLa[K]
5.7e+04
Relative Intensity (%)
m/z
2+
K A A P G A A G S R
y9 y8 y7 y6 y5 y4 y3 y1
b1 b2 b3 b7
y7+ 615.32
y2-H2O+ 244.16
b3+ 343.20
b2+ 272.16
y8+ 686.35
b1+ 201.12
y9+ 757.39
y6+ 518.27
y4+ 390.21
y5+ 461.25
y7-H2O+ 597.30
b7+ 639.39
y8-H2O+ 668.34
y1-H2O+ 157.11

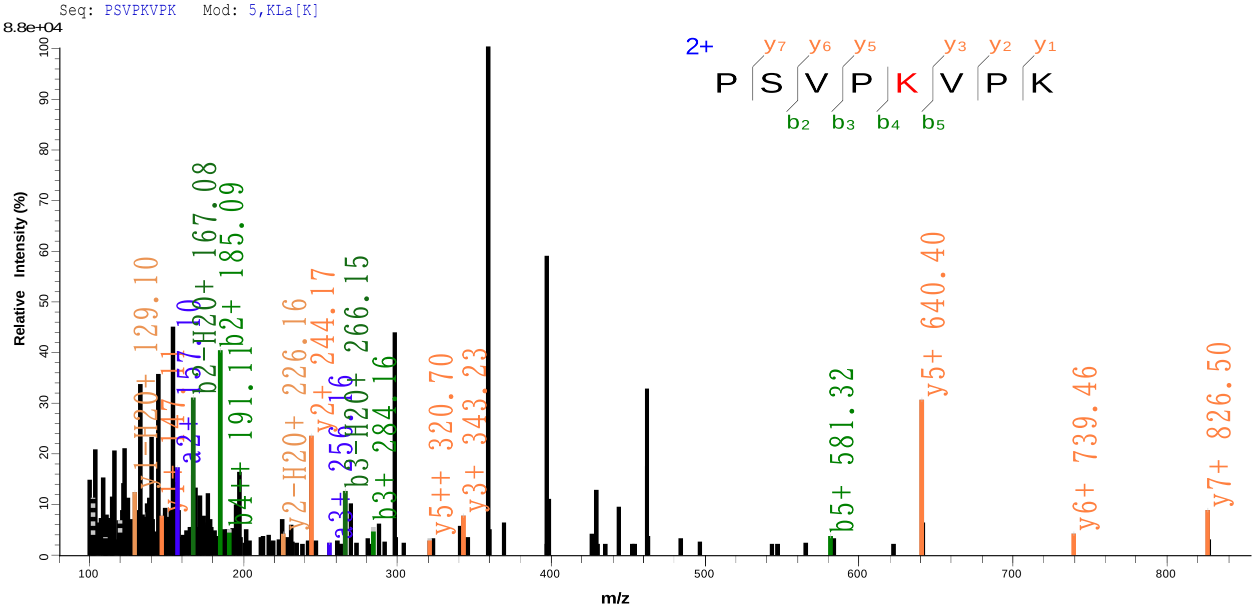
Seq: PSVPKVPK Mod: 5,KLa[K]
8.8e+04
Relative Intensity (%)
m/z
2+
P S V P K V P K
y7 y6 y5 y3 y2 y1
b2 b3 b4 b5
y5+ 640.40
b2-H2O+ 167.08
b2+ 185.09
b3-H2O+ 266.15
y2+ 244.17
y2-H2O+ 226.16
b3+ 284.16
b4+ 191.11
y5++ 320.70
y3+ 343.23
b5+ 581.32
y6+ 739.46
y7+ 826.50
129.10

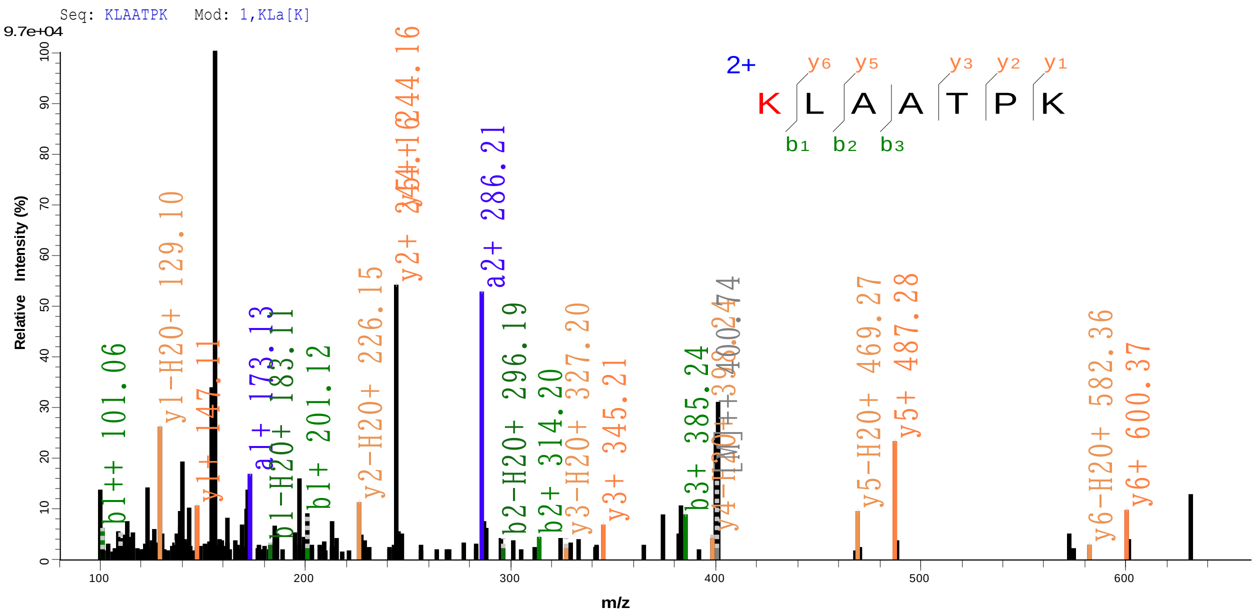
Seq: KLAATPK Mod: 1,KLa[K]
9.7e+04
Relative Intensity (%)
m/z
2+
K L A A T P K
y6 y5 y3 y2 y1
b1 b2 b3
y1-H2O+ 129.10
y1+ 147.11
a1+ 173.13
b1-H2O+ 183.11
b1+ 201.12
y2-H2O+ 226.15
a2+ 286.21
b2-H2O+ 296.19
b2+ 314.20
y3-H2O+ 327.20
y3+ 345.21
b3+ 385.24
y5-H2O+ 469.27
y5+ 487.28
y6-H2O+ 582.36
y6+ 600.37

H2A

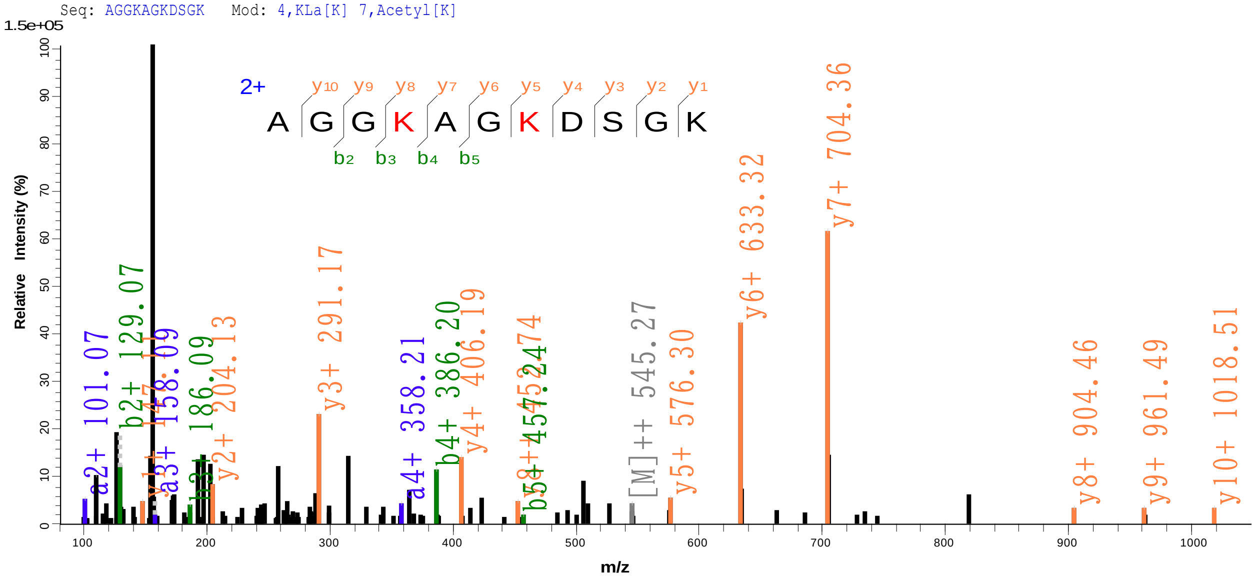
Seq: AGGKAGKDSGK Mod: 4,KLa[K] 7,Acetyl[K]
1.5e+05
Relative Intensity (%)
m/z
2+
A G G K A G K D S G K
y10 y9 y8 y7 y6 y5 y4 y3 y2 y1
b2 b3 b4 b5
a2+ 101.07
b2+ 129.07
a3+ 158.09
b3+ 186.09
y2+ 204.13
y3+ 291.17
a4+ 358.21
b4+ 386.20
y4+ 406.19
[M]++ 545.27
y5+ 576.30
y6+ 633.32
y7+ 704.36
y8+ 904.46
y9+ 961.49
y10+ 1018.51

Seq: AGKDSGKAK   Mod: 3,KLa[K] 7,Acetyl[K]

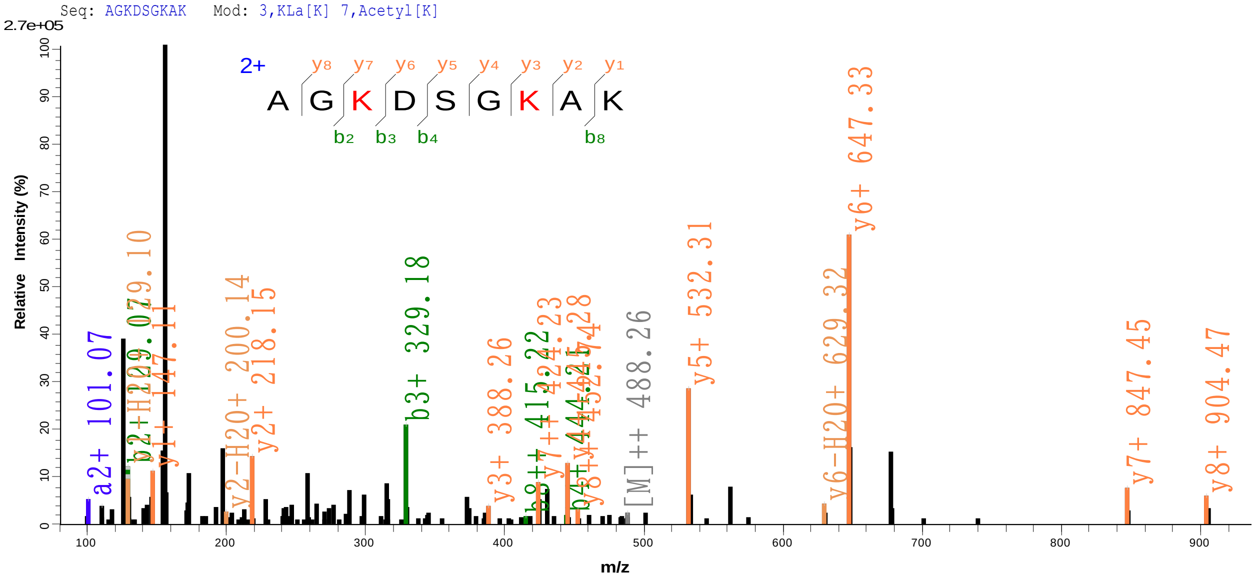

Seq: AKTKAVSR   Mod: 2,Acetyl[K] 4,KLa[K]

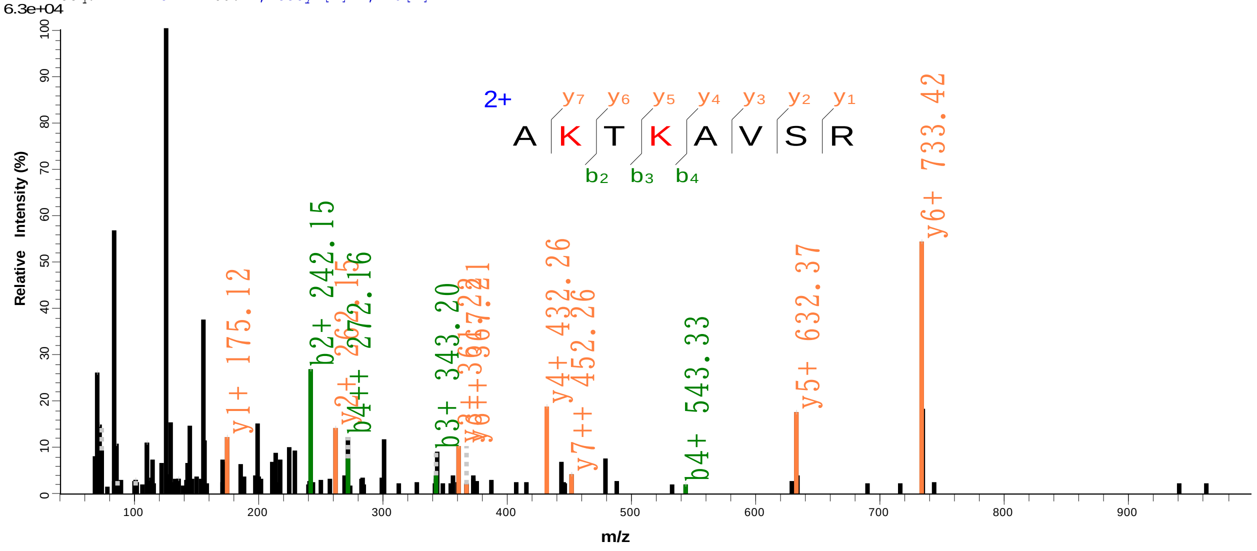

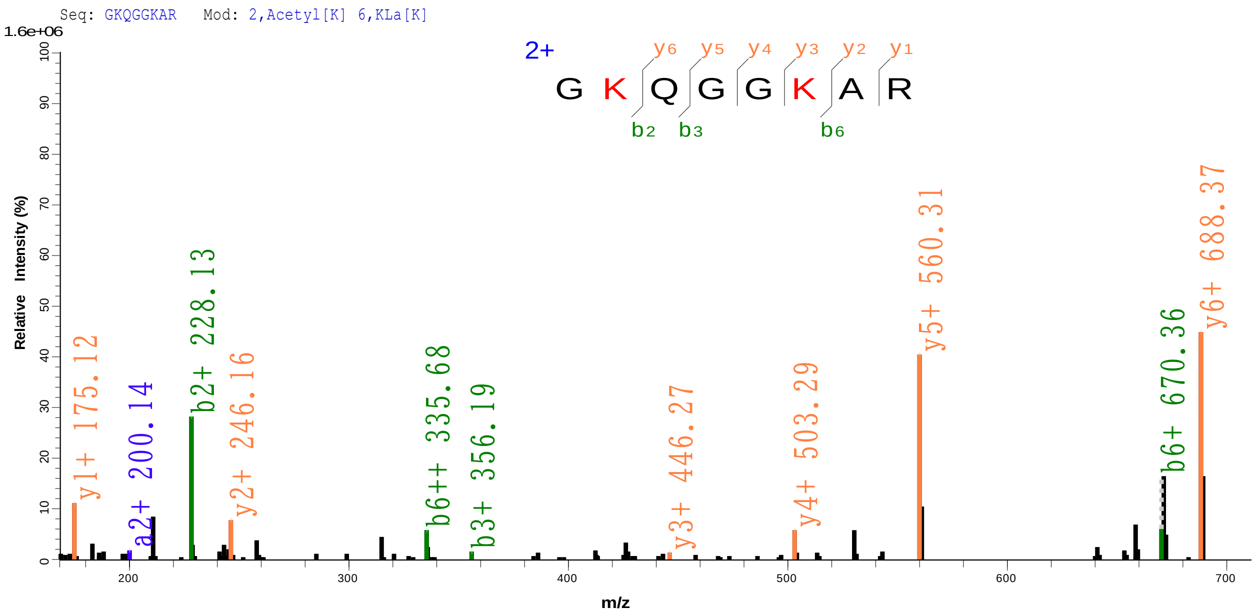
Seq: GKQGGKAR Mod: 2,Acetyl[K] 6,KLa[K]
1.6e+06
2+
G K Q G G K A R
y6 y5 y4 y3 y2 y1
b2 b3 b6
Relative Intensity (%)
m/z
y1+ 175.12
a2+ 200.14
b2+ 228.13
y2+ 246.16
b6++ 335.68
b3+ 356.19
y3+ 446.27
y4+ 503.29
y5+ 560.31
b6+ 670.36
y6+ 688.37

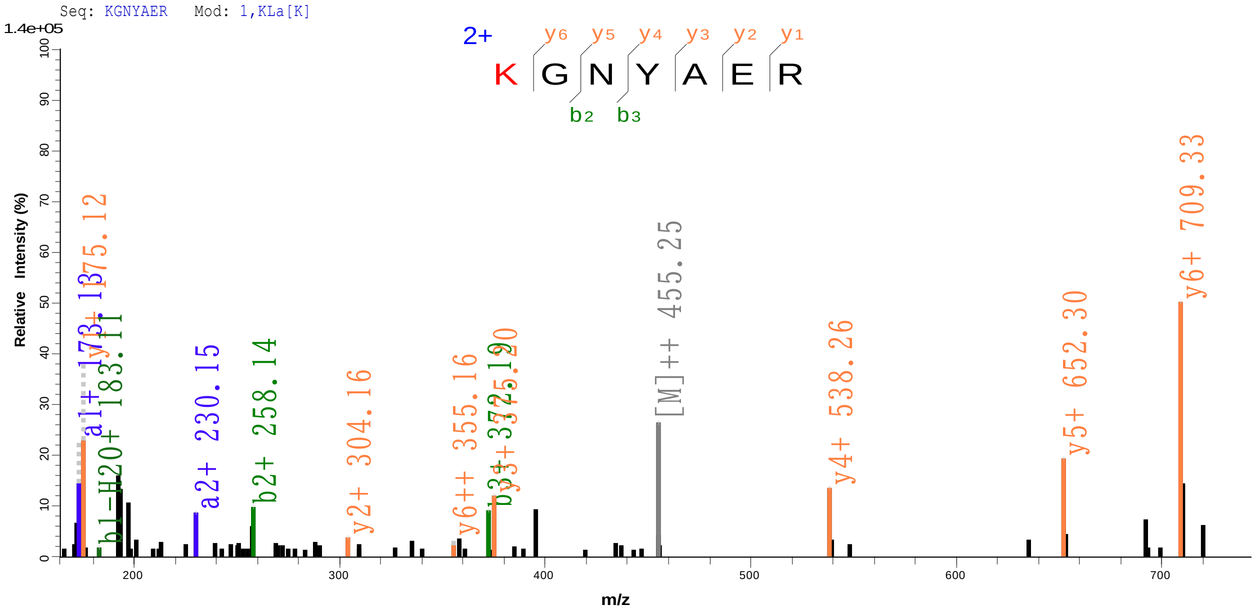
Seq: KGNYAER Mod: 1,KLa[K]
1.4e+05
2+
K G N Y A E R
y6 y5 y4 y3 y2 y1
b2 b3
Relative Intensity (%)
m/z
a2+ 230.15
b2+ 258.14
y2+ 304.16
y6++ 355.16
[M]++ 455.25
y4+ 538.26
y5+ 652.30
y6+ 709.33

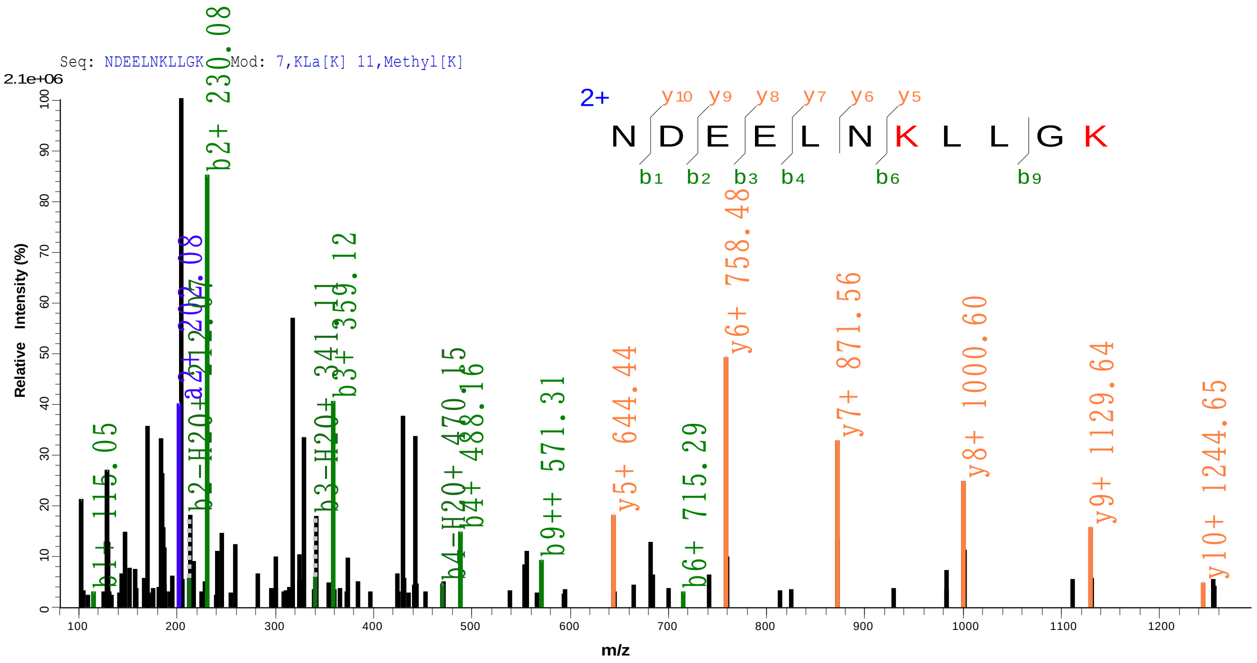
Seq: NDEELNKLLGK Mod: 7,KLa[K] 11,Methyl[K]
2.1e+06
Relative Intensity (%)
m/z
2+
N D E E L N K L L G K

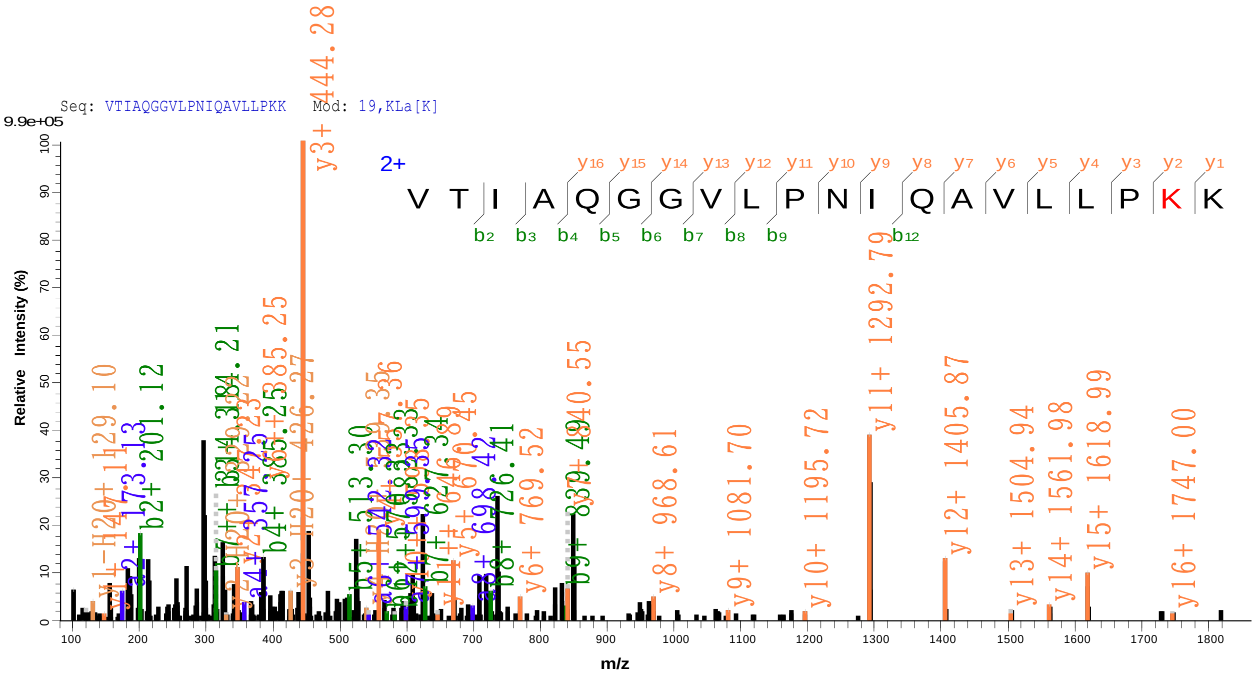
Seq: VTIAQGGVLPNIQAVLLPKK Mod: 19,KLa[K]
9.9e+05
Relative Intensity (%)
m/z
2+
V T I A Q G G V L P N I Q A V L L P K K

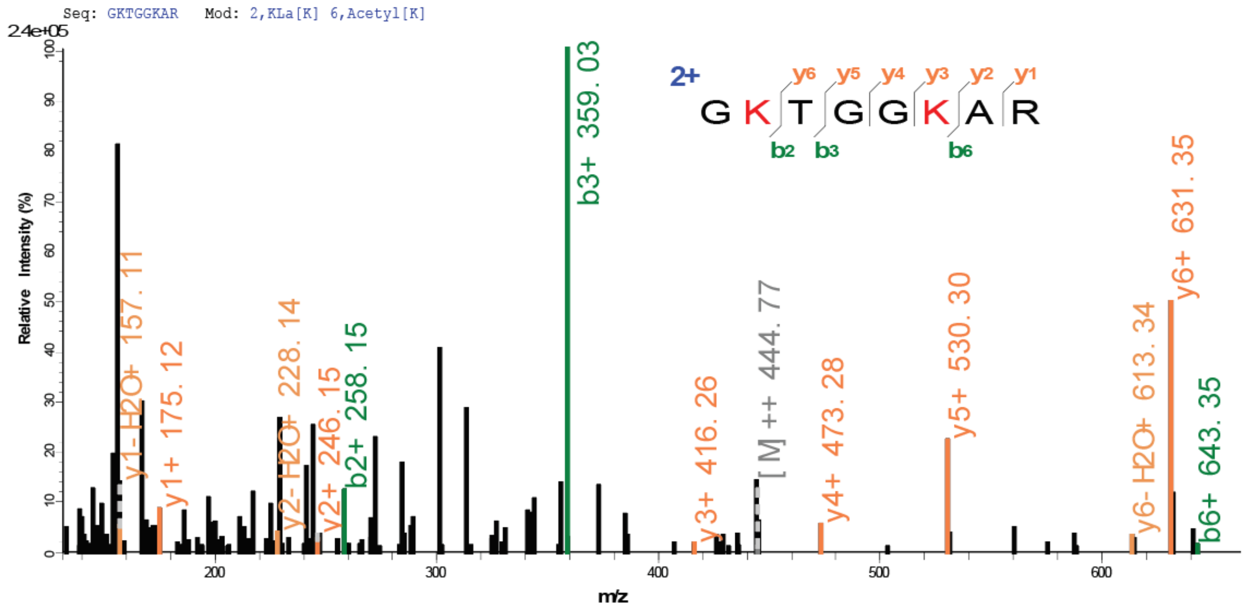

Seq: GKTGGKAR Mod: 2,KLa[K] 6,Acetyl[K]
2.4e+05
Relative Intensity (%)
m/z
2+
G K T G G K A R
y6 y5 y4 y3 y2 y1
b2 b3 b6
b3+ 359. 03
y1-H2O+ 157. 11
y1+ 175. 12
y2-H2O+ 228. 14
y2+ 246. 15
b2+ 258. 15
y3+ 416. 26
[M]++ 444. 77
y4+ 473. 28
y5+ 530. 30
y6-H2O+ 613. 34
y6+ 631. 35
b6+ 643. 35

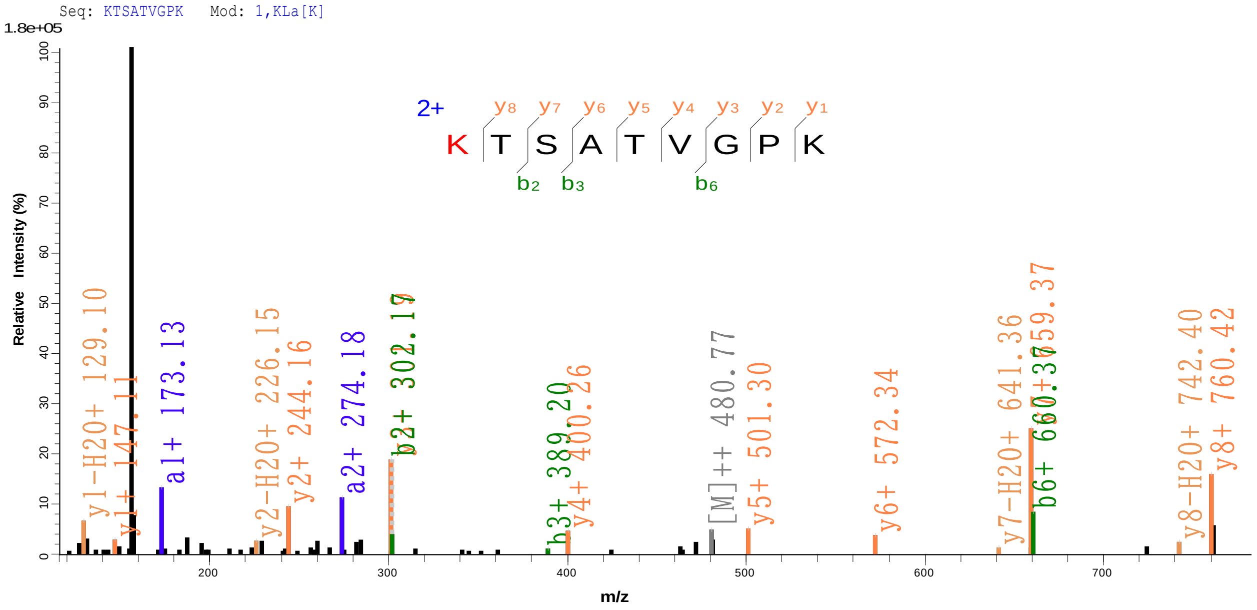

Seq: KTSATVGPK Mod: 1,KLa[K]
1.8e+05
Relative Intensity (%)
m/z
2+
K T S A T V G P K
y8 y7 y6 y5 y4 y3 y2 y1
b2 b3 b6
y1-H2O+ 129.10
y1+ 147.11
a1+ 173.13
y2-H2O+ 226.15
y2+ 244.16
a2+ 274.18
b2+ 302.19
b3+ 389.20
y4+ 400.26
[M]++ 480.77
y5+ 501.30
y6+ 572.34
y7-H2O+ 641.36
b6+ 660.37
y8-H2O+ 742.40
y8+ 760.42

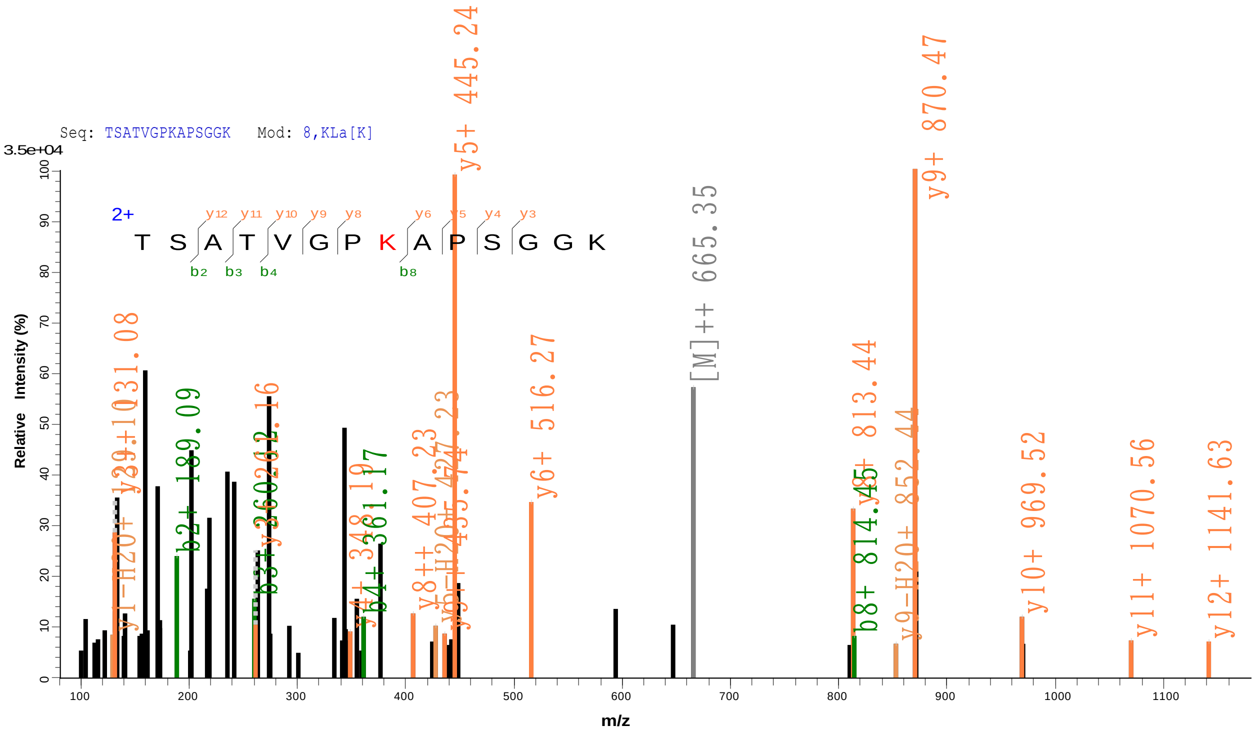

Seq: TSATVGPKAPSGGK Mod: 8,KLa[K]
3.5e+04
Relative Intensity (%)
2+
T S A T V G P K A P S G G K
y5+ 445.24
y9+ 870.47
[M]++ 665.35
y6+ 516.27
b2+ 189.09
y8+ 813.44
b8+ 814.45
y9-H2O+ 852.44
y10+ 969.52
y11+ 1070.56
y12+ 1141.63
b4+ 361.17
y8++ 407.23
m/z

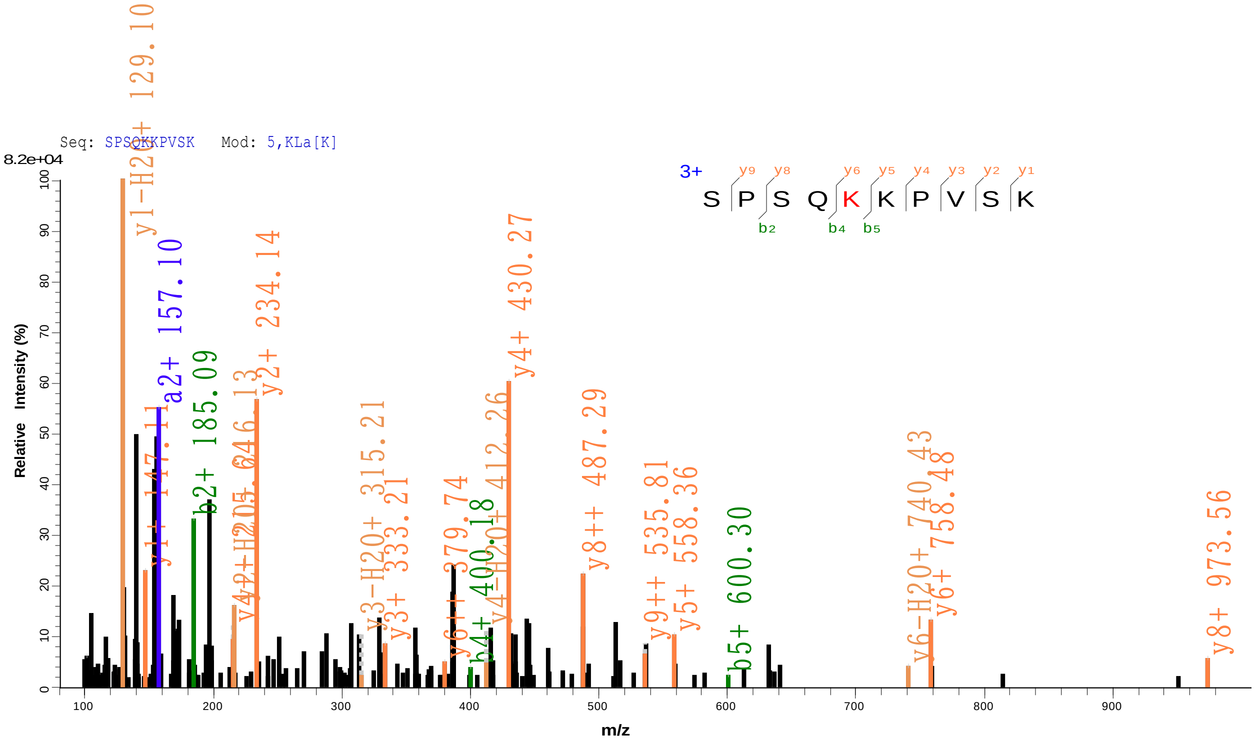

Seq: SPSQKKPVSK Mod: 5,KLa[K]
8.2e+04
Relative Intensity (%)
3+
S P S Q K K P V S K
y1-H2O+ 129.10
a2+ 157.10
b2+ 185.09
y2+ 234.14
y4+ 430.27
y8++ 487.29
y9++ 535.81
y5+ 558.36
b5+ 600.30
y6-H2O+ 740.43
y6+ 758.48
y8+ 973.56
y3-H2O+ 315.21
y3+ 333.21
b4+ 400.18
m/z

H2B

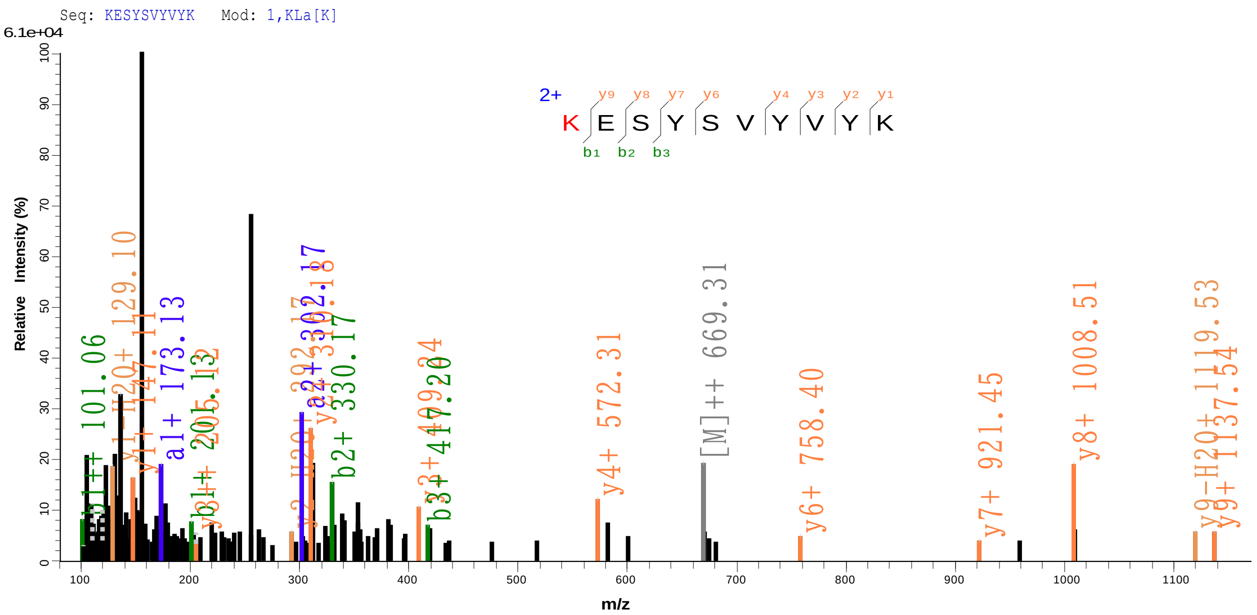
Seq: KESYSVYVYK Mod: 1,KLa[K]
6.1e+04
Relative Intensity (%)
m/z
2+
K E S Y S V Y V Y K
y9 y8 y7 y6 y4 y3 y2 y1
b1 b2 b3
101.06
a1+ 173.13
b2+ 330.17
y4+ 572.31
[M]++ 669.31
y6+ 758.40
y7+ 921.45
y8+ 1008.51

H3

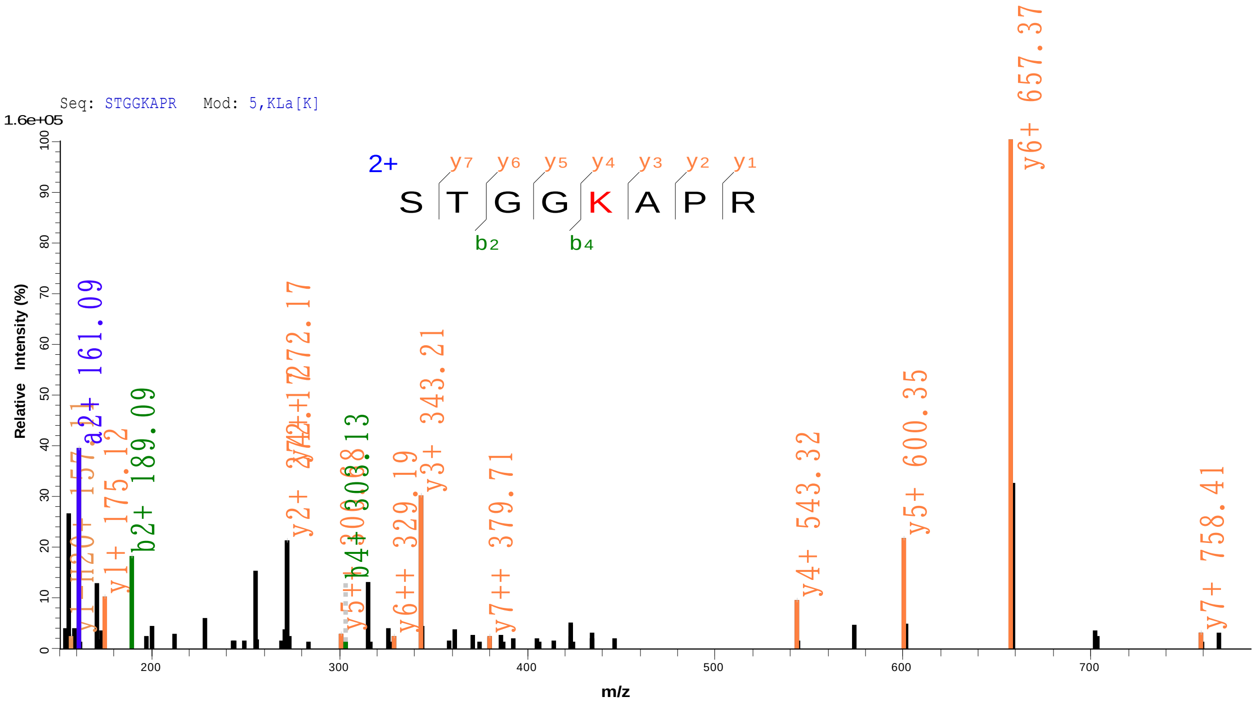
Seq: STGGKAPR Mod: 5,KLa[K]
1.6e+05
Relative Intensity (%)
m/z
2+
S T G G K A P R
y7 y6 y5 y4 y3 y2 y1
b2 b4
a2+ 161.09
y1+ 175.12
b2+ 189.09
y3+ 343.21
y7++ 379.71
y4+ 543.32
y5+ 600.35
y6+ 657.37
y7+ 758.41

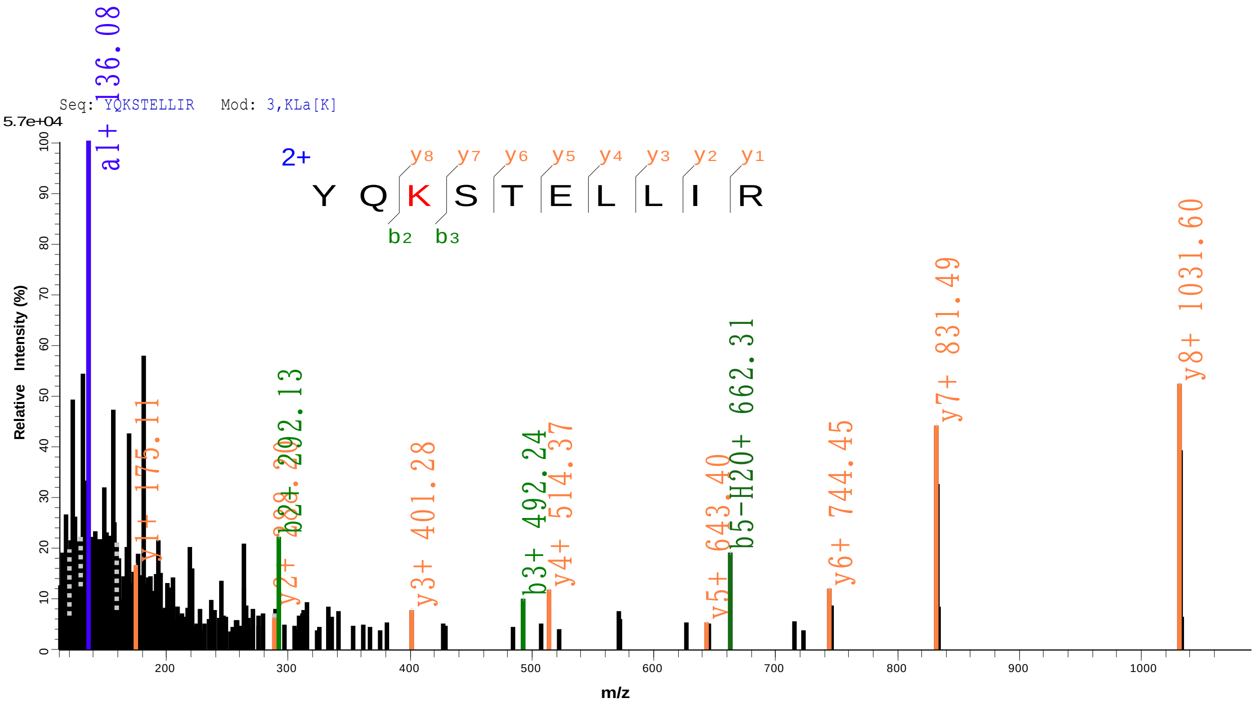
Seq: YQKSTELLIR Mod: 3,KLa[K]
5.7e+04
Relative Intensity (%)
m/z
2+
Y Q K S T E L L I R
a1+ 136.08
y1+ 175.11
y2+ 289.20
b2+ 292.13
y3+ 401.28
b3+ 492.24
y4+ 514.37
y5+ 643.40
b5-H2O+ 662.31
y6+ 744.45
y7+ 831.49
y8+ 1031.60

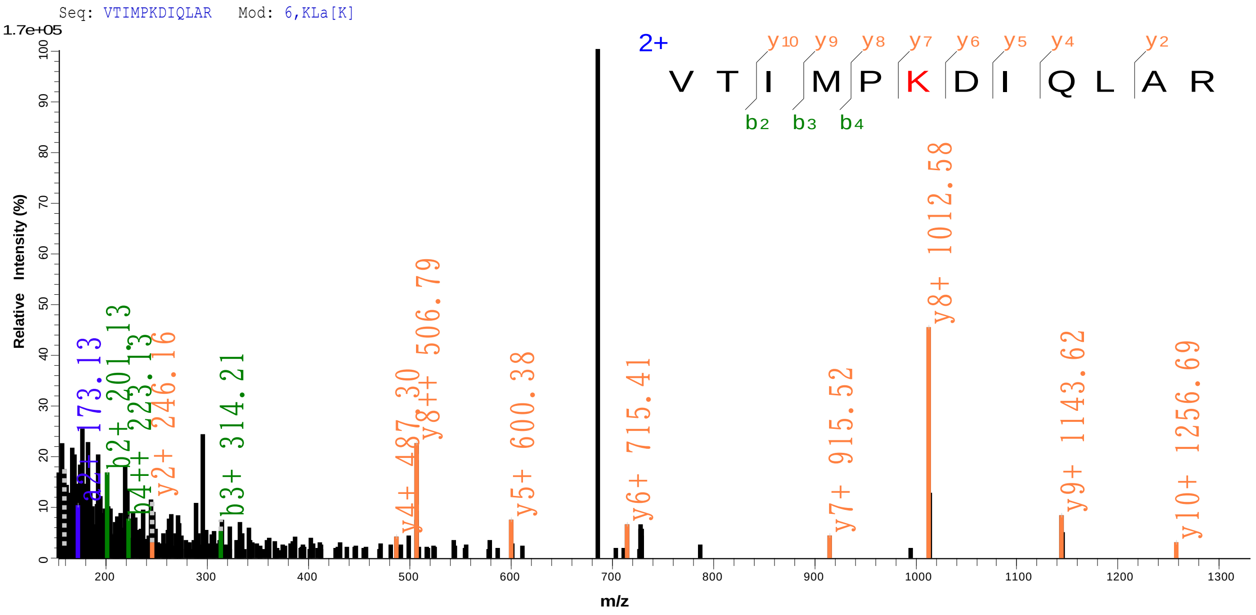
Seq: VTIMPKDIQLAR Mod: 6,KLa[K]
1.7e+05
Relative Intensity (%)
m/z
2+
V T I M P K D I Q L A R
a2+ 173.13
b2+ 201.13
b4++ 223.13
y2+ 246.16
b3+ 314.21
y4+ 487.30
y8++ 506.79
y5+ 600.38
y6+ 715.41
y7+ 915.52
y8+ 1012.58
y9+ 1143.62
y10+ 1256.69

H4

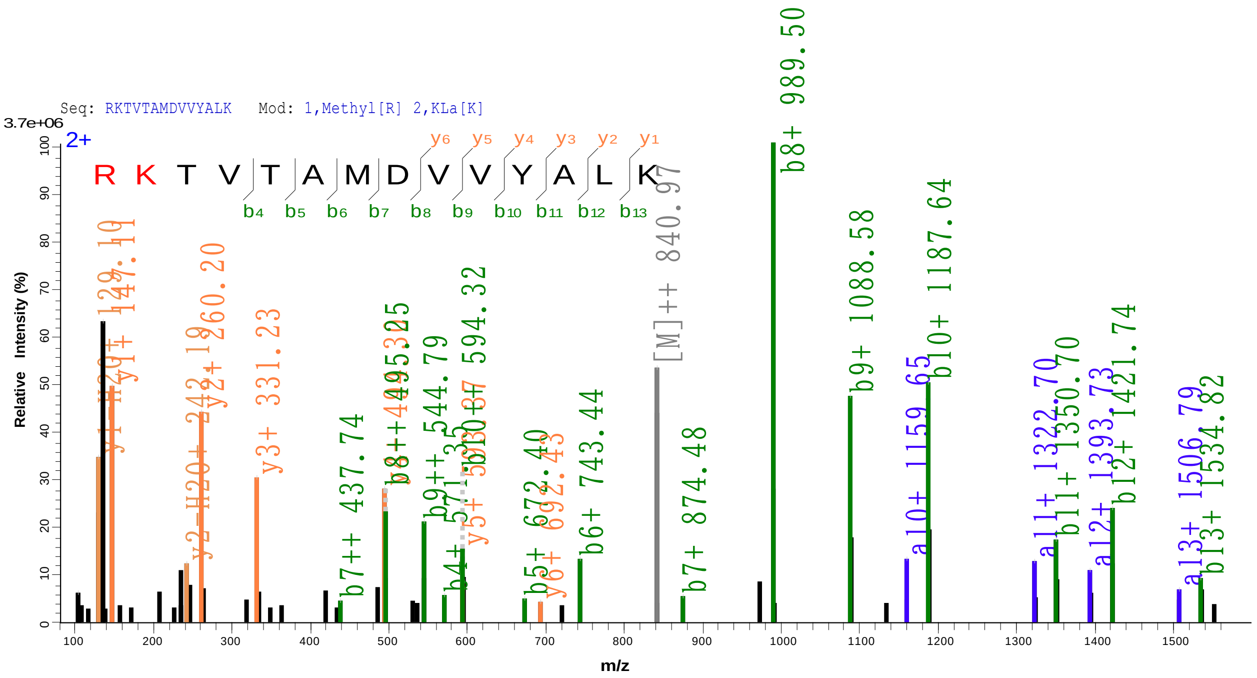

Seq: RKTVTAMDVVYALK Mod: 1,Methyl[R] 2,KLa[K]
3.7e+06
2+
R K T V T A M D V V Y A L K
b8+ 989.50
b9+ 1088.58
b10+ 1187.64
[M]++ 840.97
b6+ 743.44
b7+ 874.48
b7++ 437.74
y3+ 331.23
y2+ 260.20
b12+ 1421.74
b13+ 1534.82
a10+ 1159.65
a11+ 1322.70
a12+ 1393.73
a13+ 1506.79
Relative Intensity (%)
m/z

B. Kcr

H1

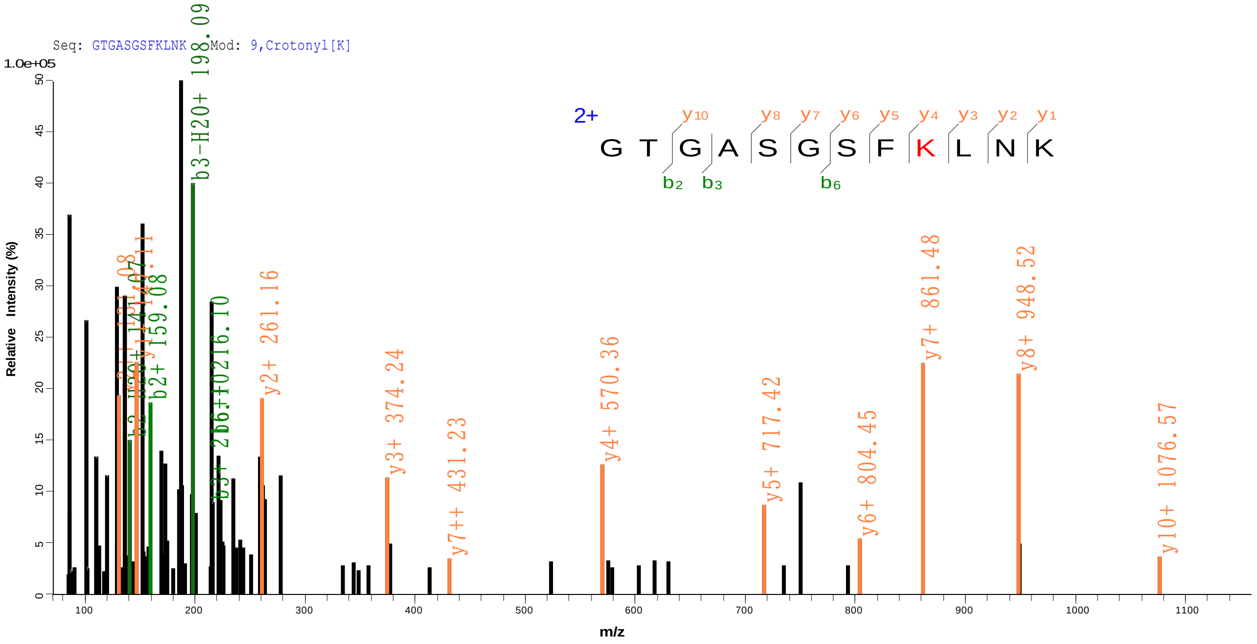

Seq: GTGASGSFKLNK Mod: 9,Crotonyl[K]
1.0e+05
2+
G T G A S G S F K L N K
b3-H2O+ 198.09
b2+ 159.08
y2+ 261.16
y3+ 374.24
y7++ 431.23
y4+ 570.36
y5+ 717.42
y6+ 804.45
y7+ 861.48
y8+ 948.52
y10+ 1076.57
Relative Intensity (%)
m/z

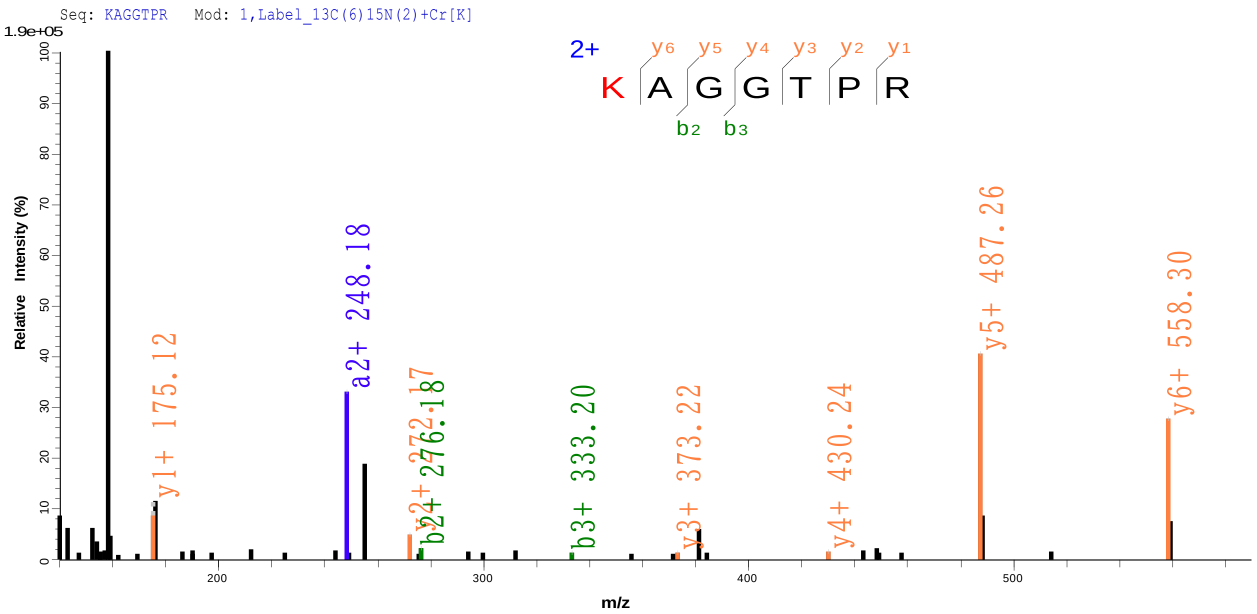
Seq: KAGGTPR Mod: 1,Label_13C(6)15N(2)+Cr[K]
2+
K A G G T P R
y6 y5 y4 y3 y2 y1
b2 b3
y1+ 175.12
a2+ 248.18
b2+ 276.18
b3+ 333.20
y3+ 373.22
y4+ 430.24
y5+ 487.26
y6+ 558.30
Relative Intensity (%)
m/z

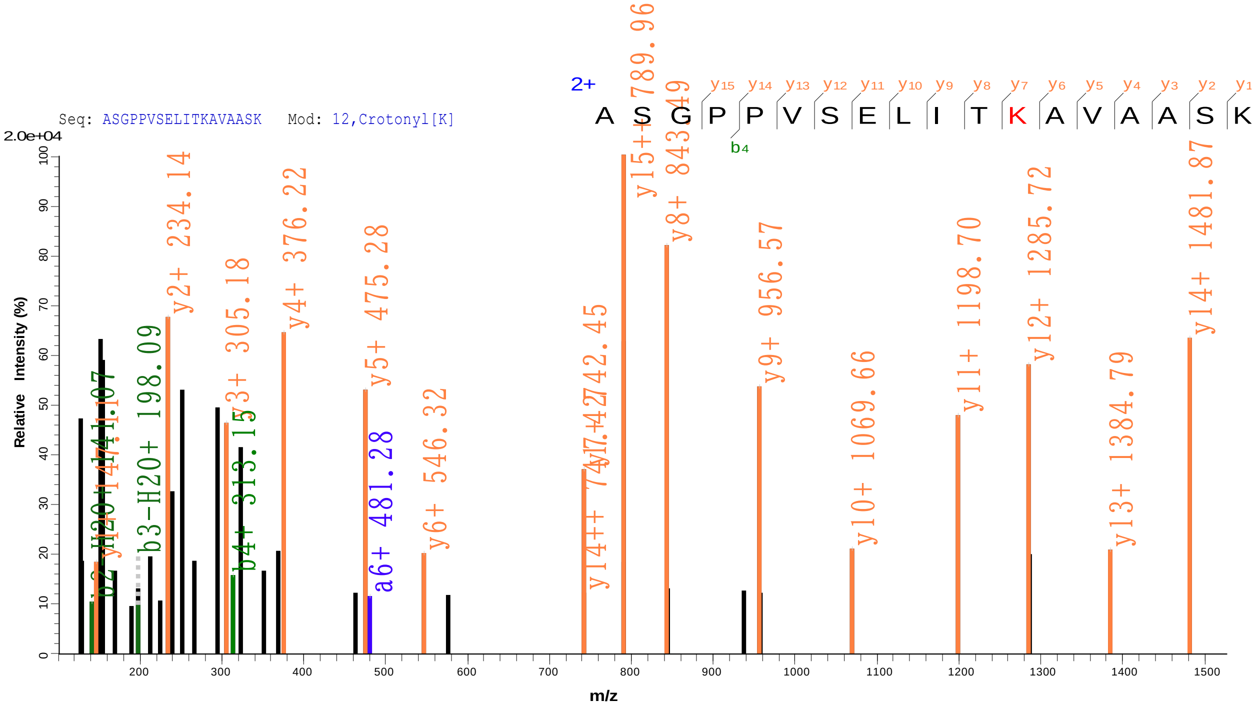
Seq: ASGPPVSELITKAVAASK Mod: 12,Crotonyl[K]
2+
A S G P P V S E L I T K A V A A S K
y15 y14 y13 y12 y11 y10 y9 y8 y7 y6 y5 y4 y3 y2 y1
b4
b3-H2O+ 198.09
y2+ 234.14
y3+ 305.18
b4+ 313.15
y4+ 376.22
y5+ 475.28
a6+ 481.28
y6+ 546.32
y15+ 789.96
y9+ 956.57
y10+ 1069.66
y11+ 1198.70
y12+ 1285.72
y13+ 1384.79
y14+ 1481.87
Relative Intensity (%)
m/z

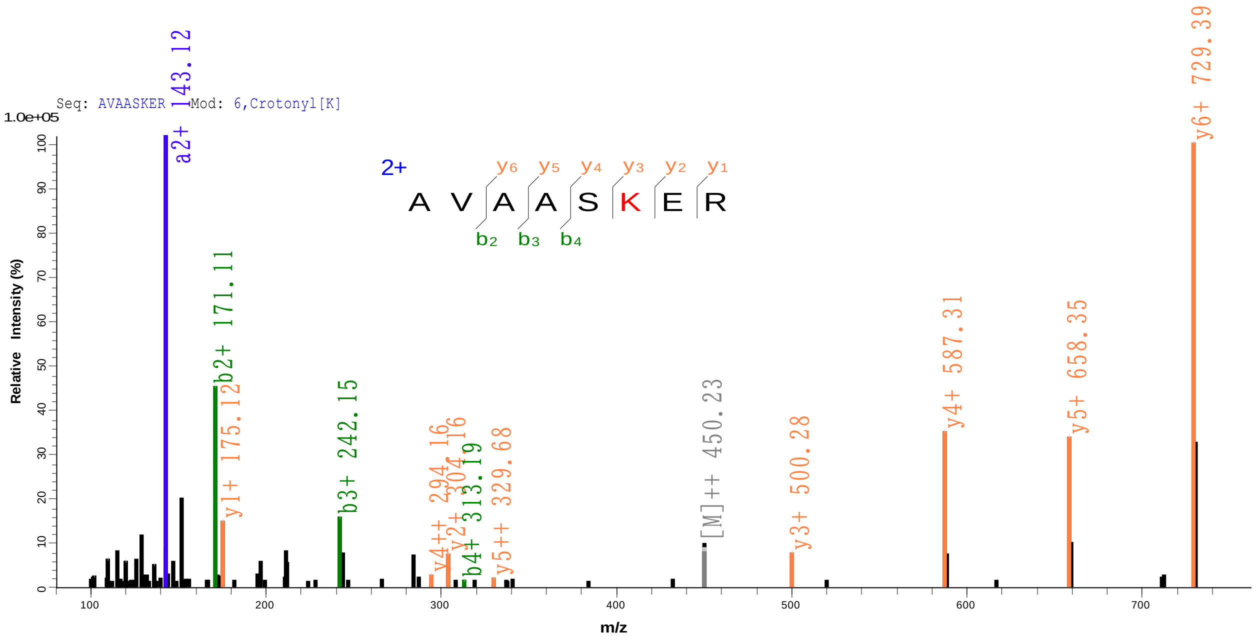
Seq: AVAASKER Mod: 6,Crotonyl[K]
1.0e+05
2+
A V A A S K E R
y6 y5 y4 y3 y2 y1
b2 b3 b4
a2+ 143.12
b2+ 171.11
y1+ 175.12
b3+ 242.15
y4++ 294.16
y2+ 304.16
b4+ 313.19
y5++ 329.68
[M]++ 450.23
y3+ 500.28
y4+ 587.31
y5+ 658.35
y6+ 729.39
Relative Intensity (%)
m/z

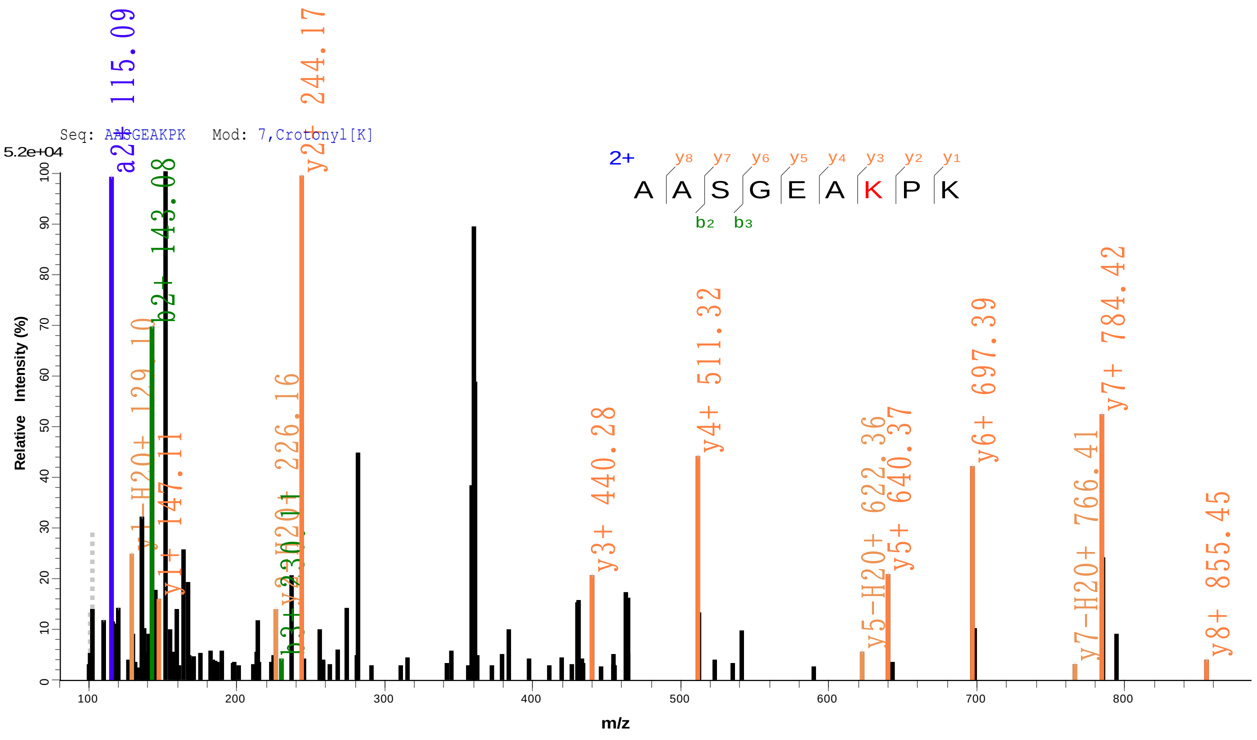
Seq: AASGEAKPK Mod: 7,Crotonyl[K]
5.2e+04
2+
A A S G E A K P K
y8 y7 y6 y5 y4 y3 y2 y1
b2 b3
a2+ 115.09
b2+ 143.08
y1-H2O+ 129.10
y1+ 147.11
y2+ 244.17
y3+ 440.28
y4+ 511.32
y5-H2O+ 622.36
y5+ 640.37
y6+ 697.39
y7-H2O+ 766.41
y7+ 784.42
y8+ 855.45
Relative Intensity (%)
m/z

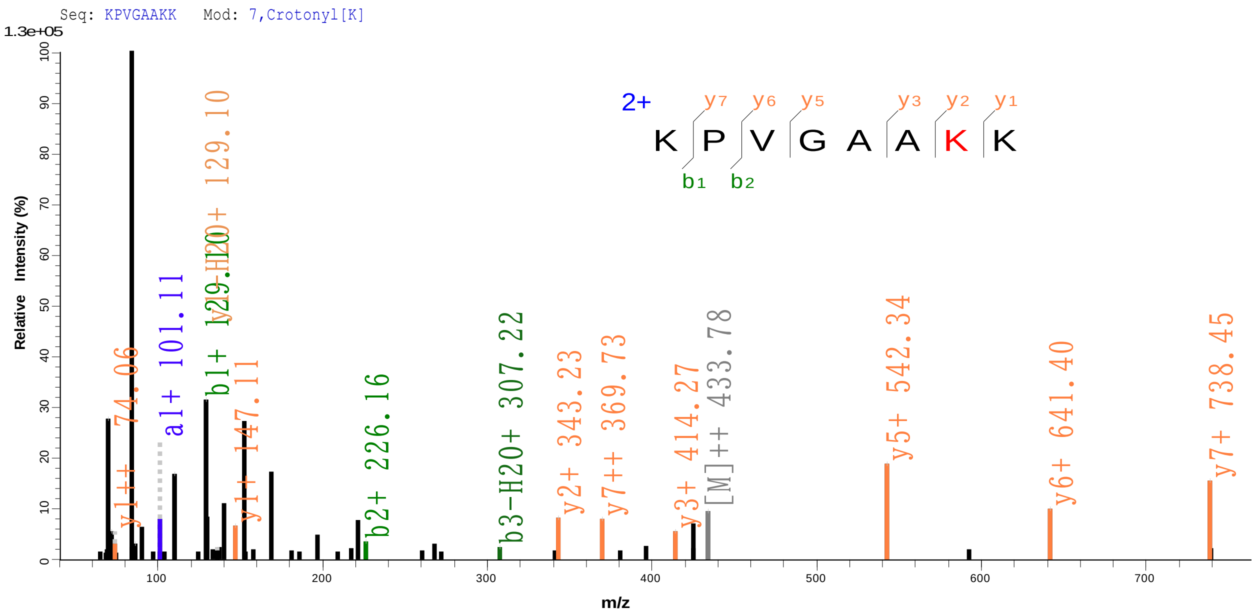
Seq: KPVGAAKK Mod: 7,Crotonyl[K]
1.3e+05
Relative Intensity (%)
m/z
2+
K P V G A A K K
y7 y6 y5 y3 y2 y1
b1 b2
y1++ 74.06
a1+ 101.11
b1+ 129.10
y1-H2O+ 129.10
y1+ 147.11
b2+ 226.16
b3-H2O+ 307.22
y2+ 343.23
y7++ 369.73
y3+ 414.27
[M]++ 433.78
y5+ 542.34
y6+ 641.40
y7+ 738.45

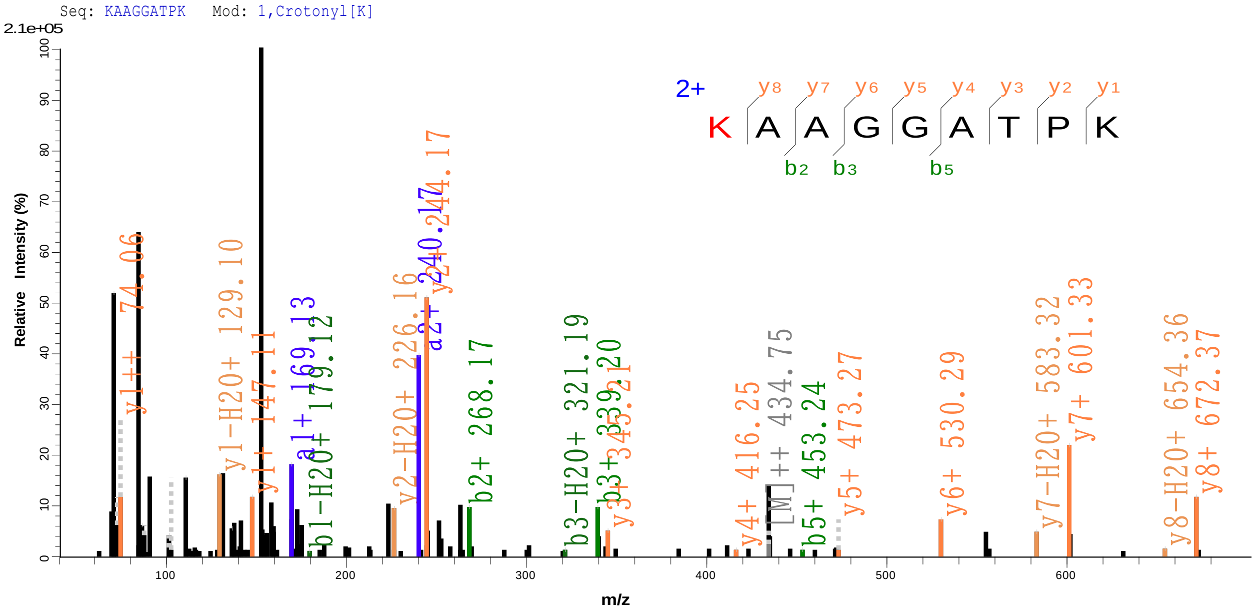
Seq: KAAGGATPK Mod: 1,Crotonyl[K]
2.1e+05
Relative Intensity (%)
m/z
2+
K A A G G A T P K
y8 y7 y6 y5 y4 y3 y2 y1
b2 b3 b5
y1++ 74.06
y1-H2O+ 129.10
y1+ 147.11
a1+ 169.13
b1-H2O+ 179.12
y2-H2O+ 226.16
a2+ 240.17
y2+ 244.17
b2+ 268.17
b3-H2O+ 321.19
b3+ 339.20
y3+ 345.21
y4+ 416.25
[M]++ 434.75
b5+ 453.24
y5+ 473.27
y6+ 530.29
y7-H2O+ 583.32
y7+ 601.33
y8-H2O+ 654.36
y8+ 672.37

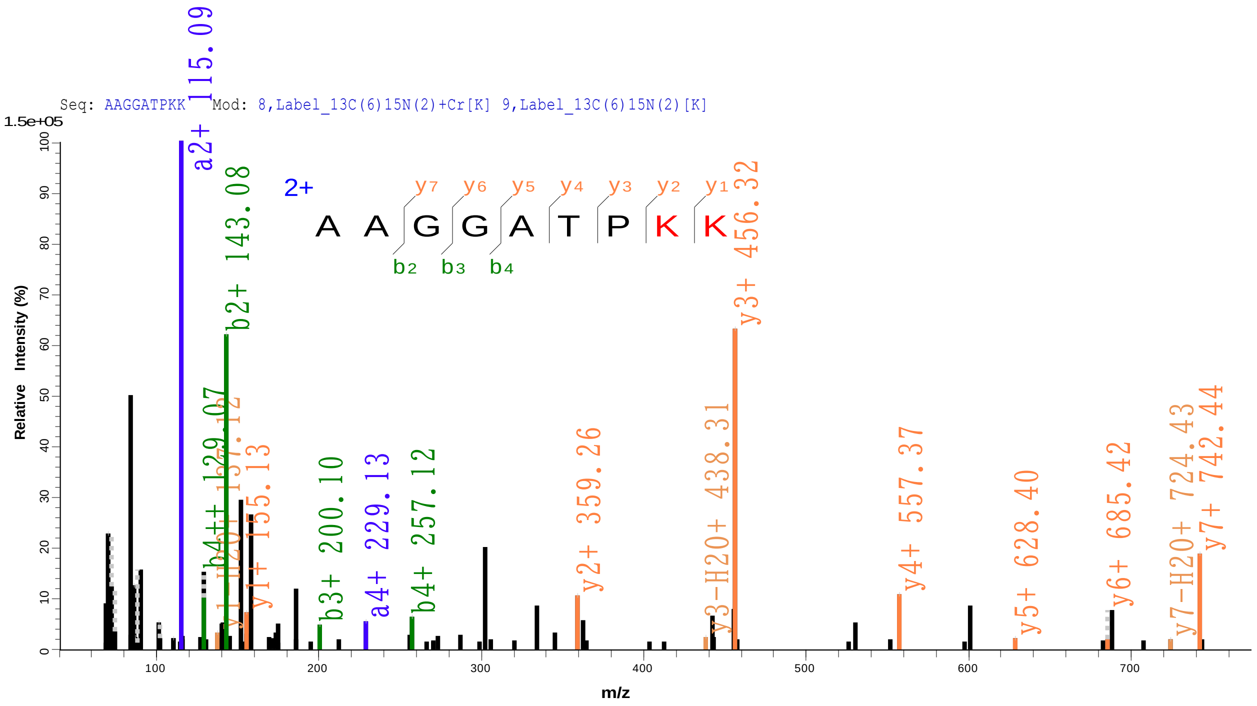
Seq: AAGGATPKK Mod: 8,Label_13C(6)15N(2)+Cr[K] 9,Label_13C(6)15N(2)[K]
1.5e+05
Relative Intensity (%)
m/z
2+
A A G G A T P K K
y7 y6 y5 y4 y3 y2 y1
b2 b3 b4
a2+ 115.09
b2+ 143.08
b4++ 129.07
y3+ 456.32
y2+ 359.26
y3-H2O+ 438.31
y4+ 557.37
y5+ 628.40
y6+ 685.42
y7-H2O+ 724.43
y7+ 742.44
b3+ 200.10
a4+ 229.13
b4+ 257.12
y1+ 155.13

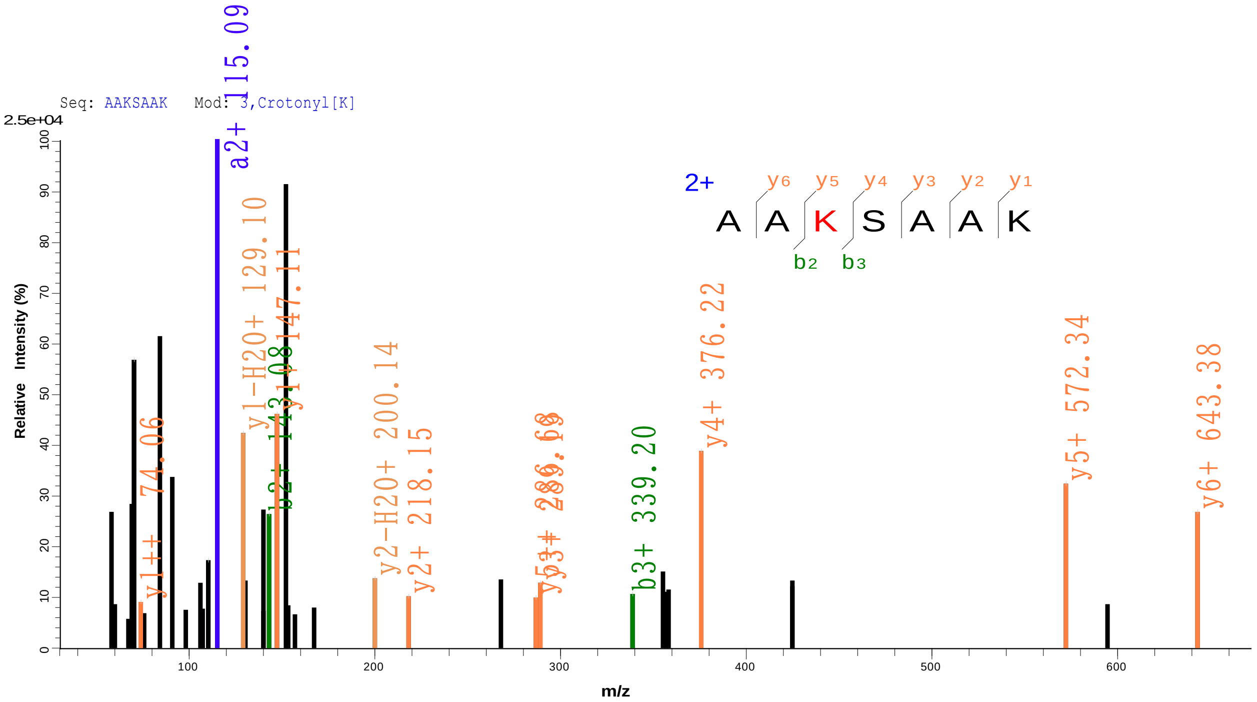
Seq: AAKSAAK Mod: 3,Crotonyl[K]
2.5e+04
Relative Intensity (%)
m/z
2+
A A K S A A K
y6 y5 y4 y3 y2 y1
b2 b3
a2+ 115.09
y1-H2O+ 129.10
y1+ 147.11
b2+ 143.08
y1++ 74.06
y2-H2O+ 200.14
y2+ 218.15
b3+ 339.20
y4+ 376.22
y5+ 572.34
y6+ 643.38

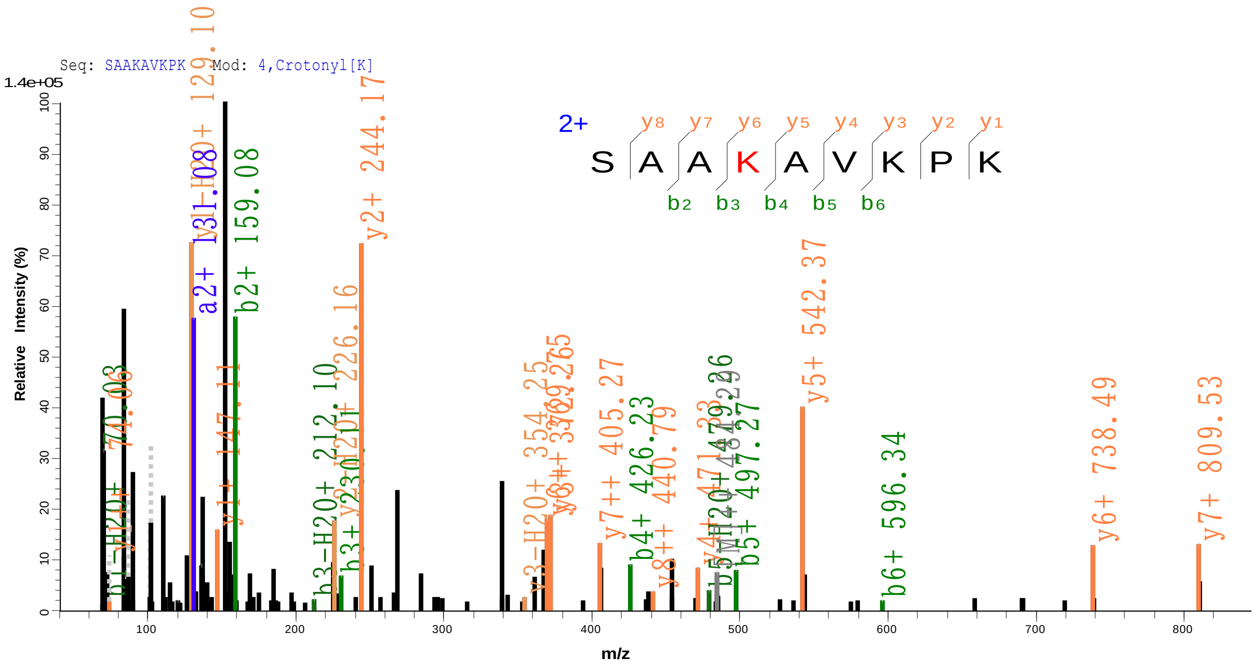

Seq: SAAKAVKPK Mod: 4,Crotonyl[K]
1.4e+05
Relative Intensity (%)
m/z
2+
S A A K A V K P K
y8 y7 y6 y5 y4 y3 y2 y1
b2 b3 b4 b5 b6
y1-H2O+ 129.10
a2+ 131.08
b2+ 159.08
y2+ 244.17
y5+ 542.37
b4+ 426.23
b5+ 497.27
b6+ 596.34
y6+ 738.49
y7+ 809.53
y7++ 405.27

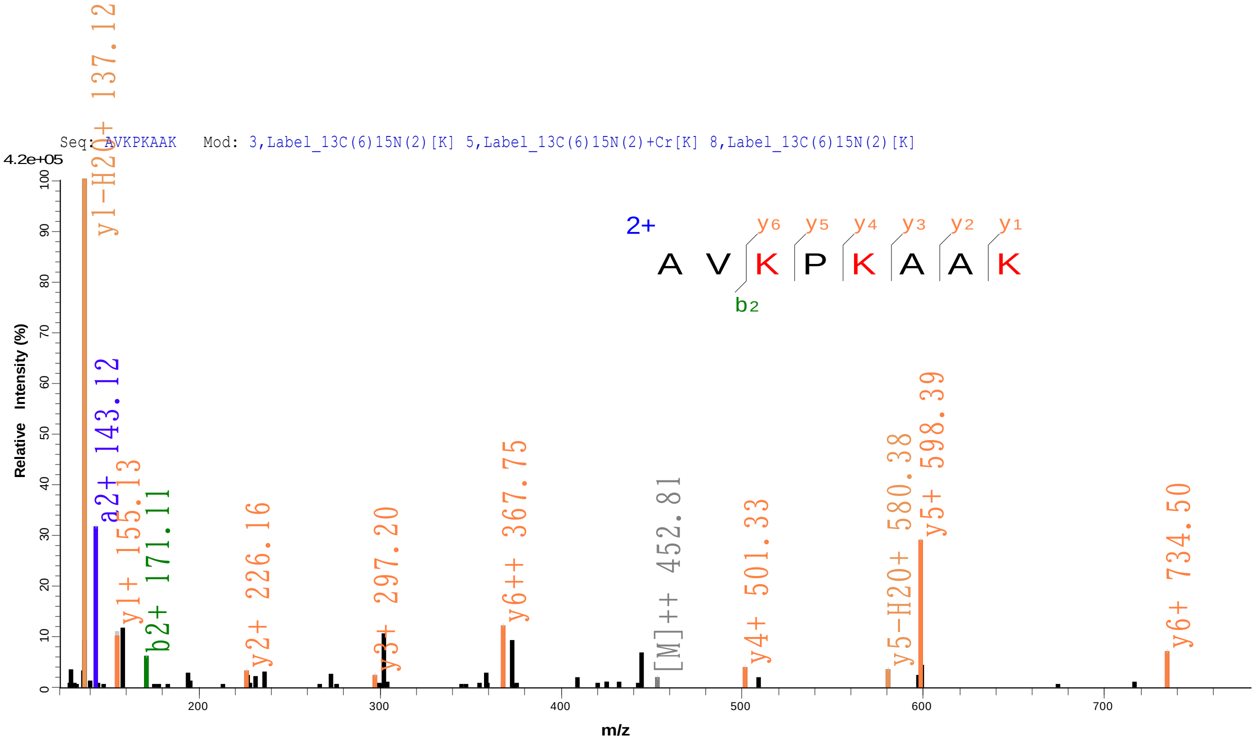

Seq: AVKPKAAK Mod: 3,Label_13C(6)15N(2)[K] 5,Label_13C(6)15N(2)+Cr[K] 8,Label_13C(6)15N(2)[K]
4.2e+05
Relative Intensity (%)
m/z
2+
A V K P K A A K
y6 y5 y4 y3 y2 y1
b2
y1-H2O+ 137.12
a2+ 143.12
y1+ 155.13
b2+ 171.11
y2+ 226.16
y3+ 297.20
y6++ 367.75
[M]++ 452.81
y4+ 501.33
y5-H2O+ 580.38
y5+ 598.39
y6+ 734.50

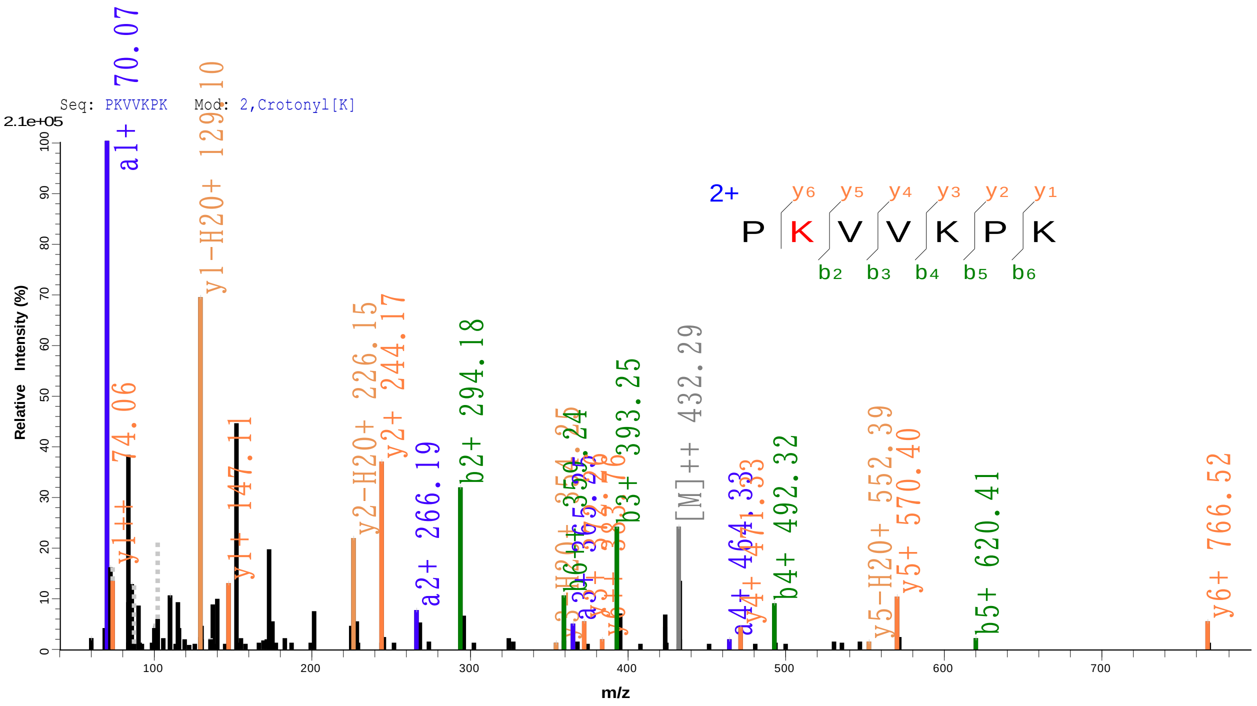

Seq: PKVVKPK Mod: 2,Crotonyl[K]
2.1e+05
Relative Intensity (%)
m/z
2+
P K V V K P K
y6 y5 y4 y3 y2 y1
b2 b3 b4 b5 b6
a1+ 70.07
y1-H2O+ 129.10
y1++ 74.06
y1+ 147.11
y2-H2O+ 226.15
y2+ 244.17
a2+ 266.19
b2+ 294.18
b3+ 393.25
[M]++ 432.29
a4+ 464.33
y4+ 471.33
b4+ 492.32
y5-H2O+ 552.39
y5+ 570.40
b5+ 620.41
y6+ 766.52

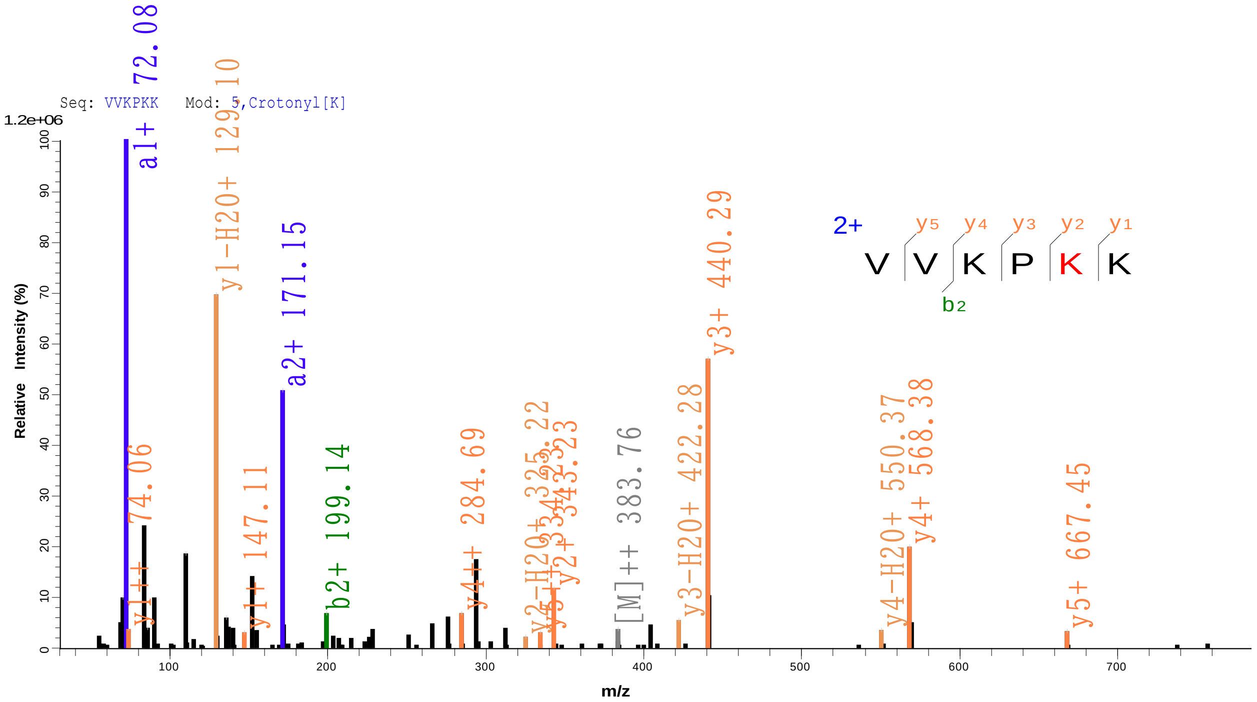

Seq: VVKPKK Mod: 5,Crotonyl[K]
1.2e+06
Relative Intensity (%)
m/z
2+
V V K P K K
y5 y4 y3 y2 y1
b2
a1+ 72.08
y1-H2O+ 129.10
y1++ 74.06
y1+ 147.11
a2+ 171.15
b2+ 199.14
y4++ 284.69
y2-H2O+ 325.22
y2+ 343.23
[M]++ 383.76
y3-H2O+ 422.28
y3+ 440.29
y4-H2O+ 550.37
y4+ 568.38
y5+ 667.45

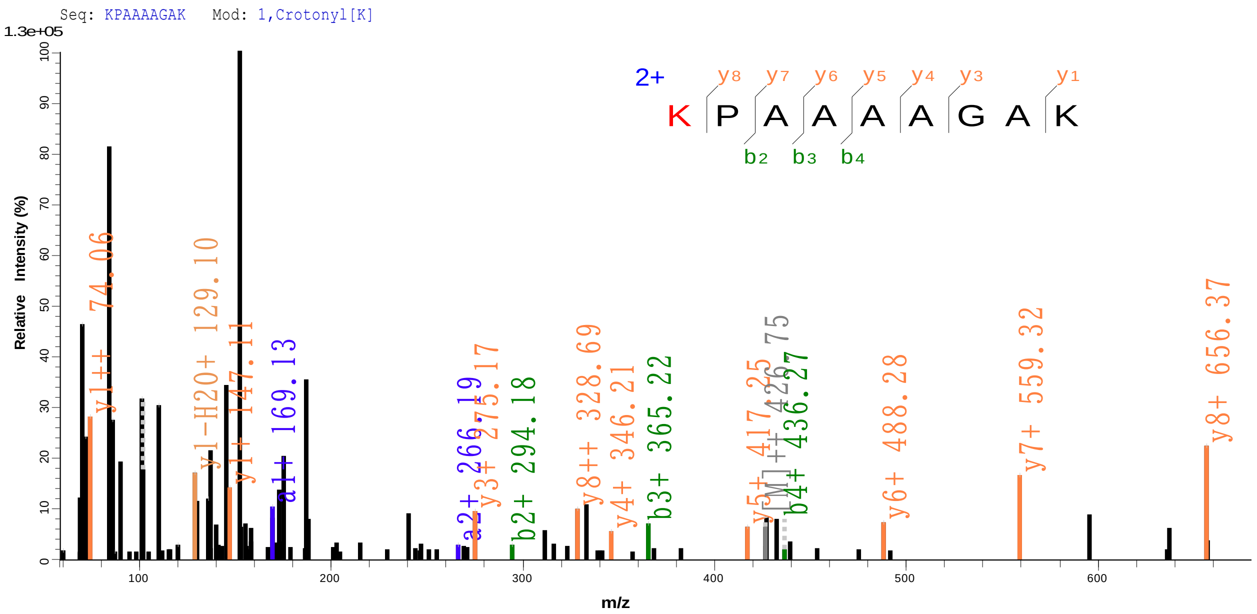
Seq: KPAAAAGAK Mod: 1,Crotonyl[K]
1.3e+05
Relative Intensity (%)
m/z
2+
K P A A A A G A K
y1++ 74.06
y1-H2O+ 129.10
y1+ 147.11
a1+ 169.13
a2+ 266.19
y3+ 275.17
b2+ 294.18
y8++ 328.69
y4+ 346.21
b3+ 365.22
b4+ 436.27
y6+ 488.28
y7+ 559.32
y8+ 656.37

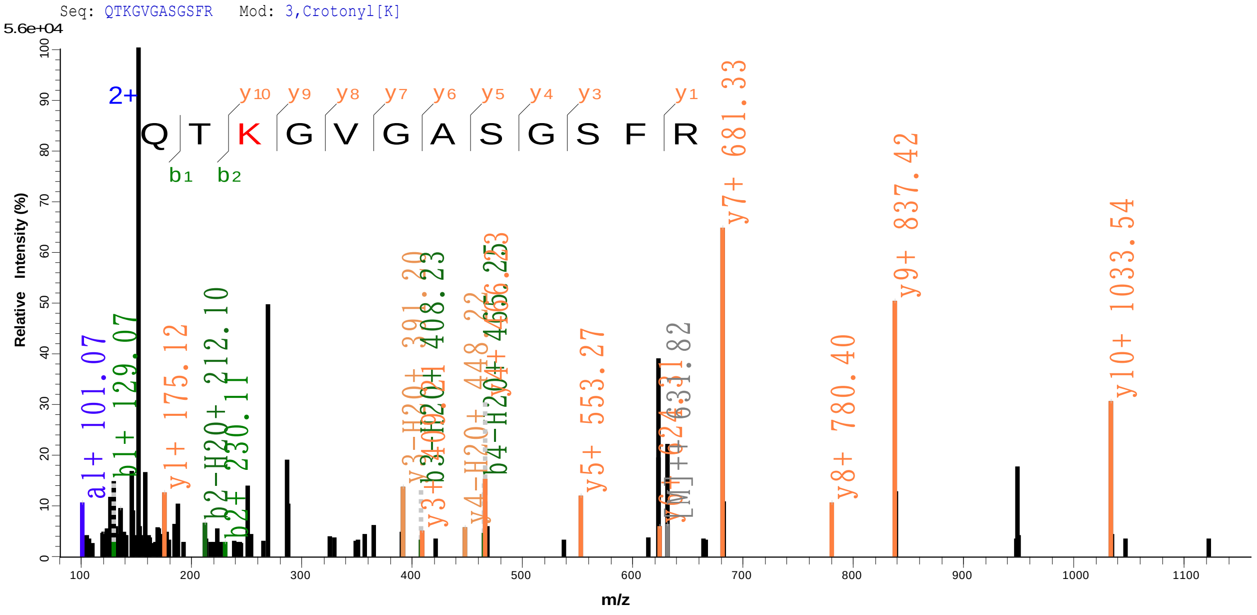
Seq: QTKGVGASGSFR Mod: 3,Crotonyl[K]
5.6e+04
Relative Intensity (%)
m/z
2+
Q T K G V G A S G S F R
a1+ 101.07
b1+ 129.07
y1+ 175.12
b2-H2O+ 212.10
b2+ 230.11
y5+ 553.27
y7+ 681.33
y8+ 780.40
y9+ 837.42
y10+ 1033.54

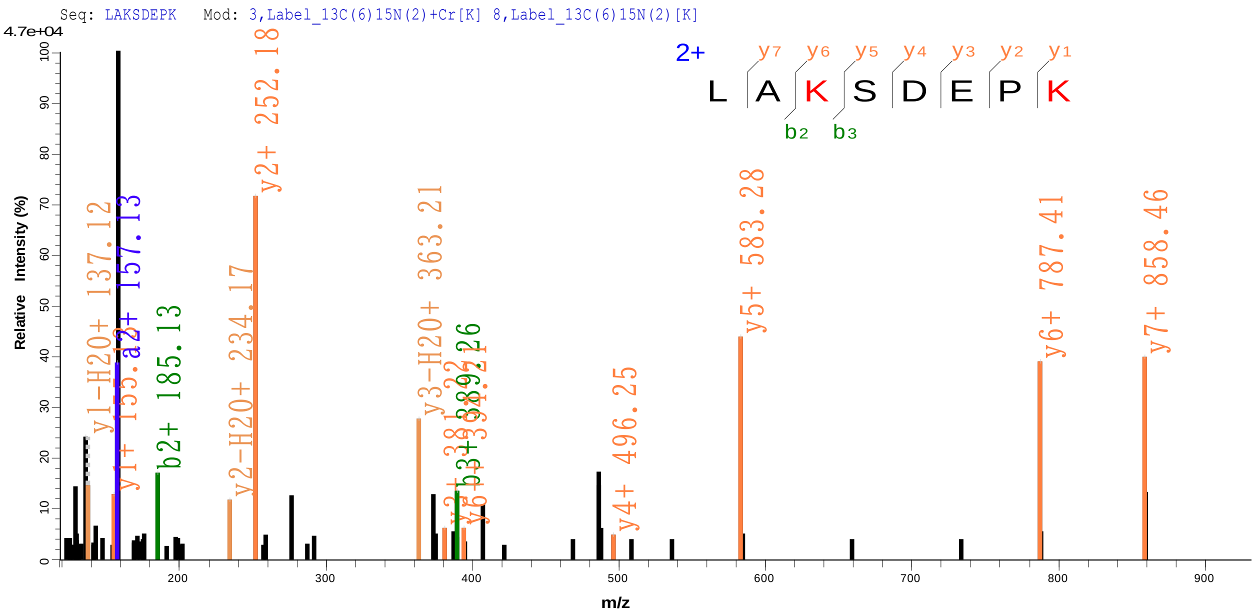
Seq: LAKSDEPK Mod: 3,Label_13C(6)15N(2)+Cr[K] 8,Label_13C(6)15N(2)[K]
4.7e+04
Relative Intensity (%)
2+
L A K S D E P K
y7 y6 y5 y4 y3 y2 y1
b2 b3
y1-H2O+ 137.12
y1+ 155.a2+ 157.13
b2+ 185.13
y2-H2O+ 234.17
y2+ 252.18
y3-H2O+ 363.21
y4+ 496.25
y5+ 583.28
y6+ 787.41
y7+ 858.46
m/z

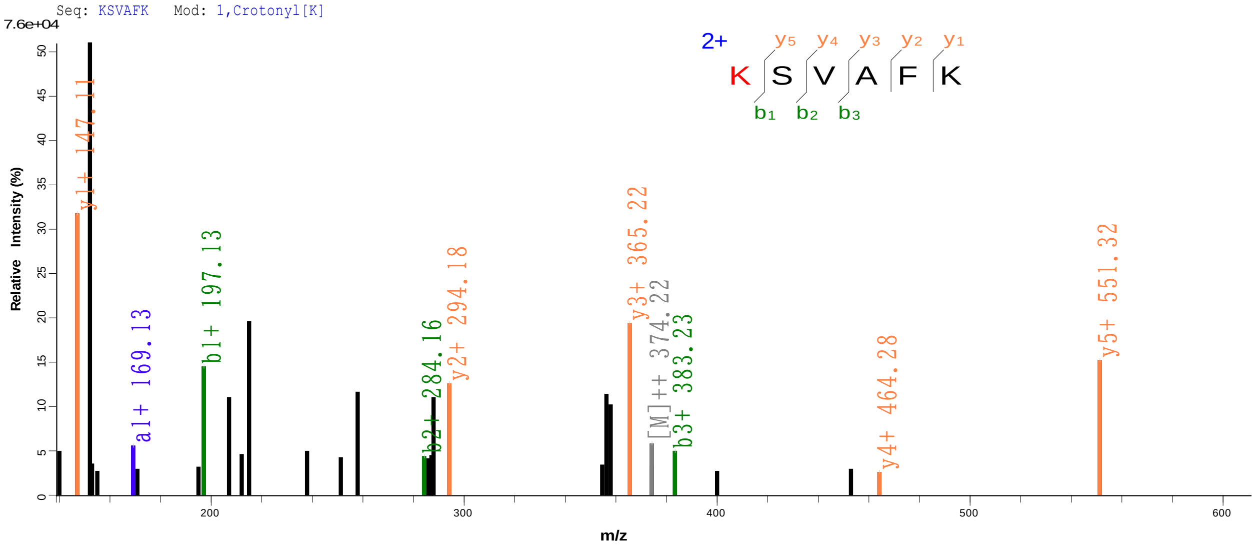
Seq: KSVAFK Mod: 1,Crotonyl[K]
7.6e+04
Relative Intensity (%)
2+
K S V A F K
y5 y4 y3 y2 y1
b1 b2 b3
y1+ 147.11
a1+ 169.13
b1+ 197.13
b2+ 284.16
y2+ 294.18
y3+ 365.22
[M]++ 374.22
b3+ 383.23
y4+ 464.28
y5+ 551.32
m/z

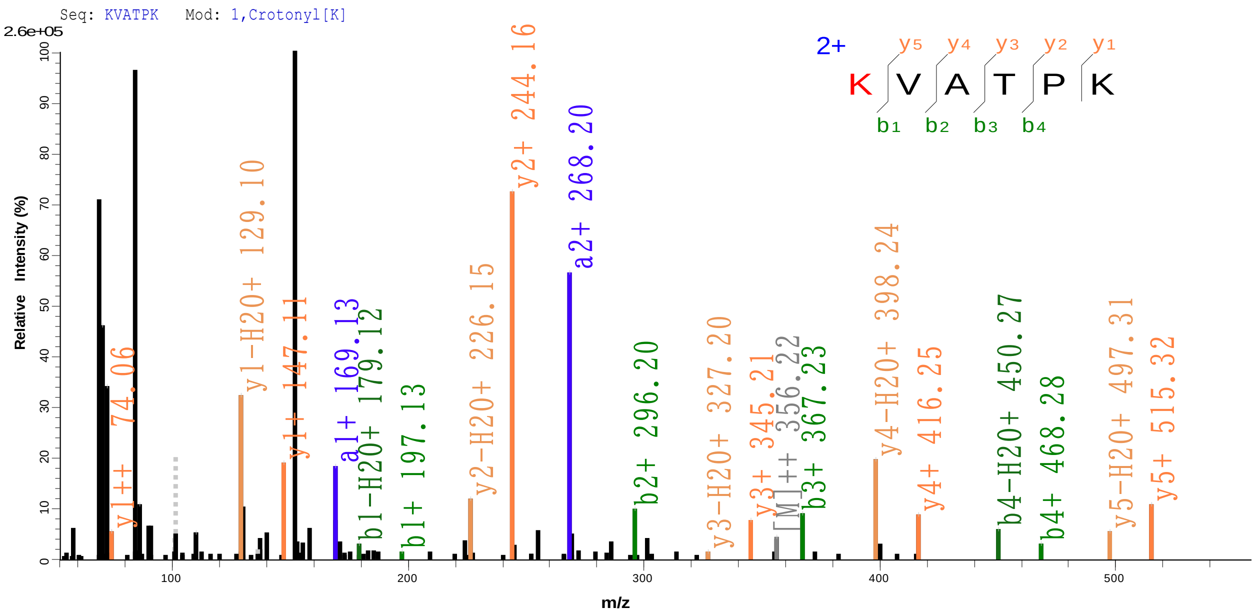

Seq: KVATPK Mod: 1,Crotonyl[K]
2.6e+05
2+
K V A T P K
y5 y4 y3 y2 y1
b1 b2 b3 b4
Relative Intensity (%)
m/z
y1++ 74.06
y1-H2O+ 129.10
y1+ 147.11
a1+ 169.13
b1-H2O+ 179.12
b1+ 197.13
y2-H2O+ 226.15
y2+ 244.16
a2+ 268.20
b2+ 296.20
y3-H2O+ 327.20
y3+ 345.21
[M]++ 356.22
b3+ 367.23
y4-H2O+ 398.24
y4+ 416.25
b4-H2O+ 450.27
b4+ 468.28
y5-H2O+ 497.31
y5+ 515.32

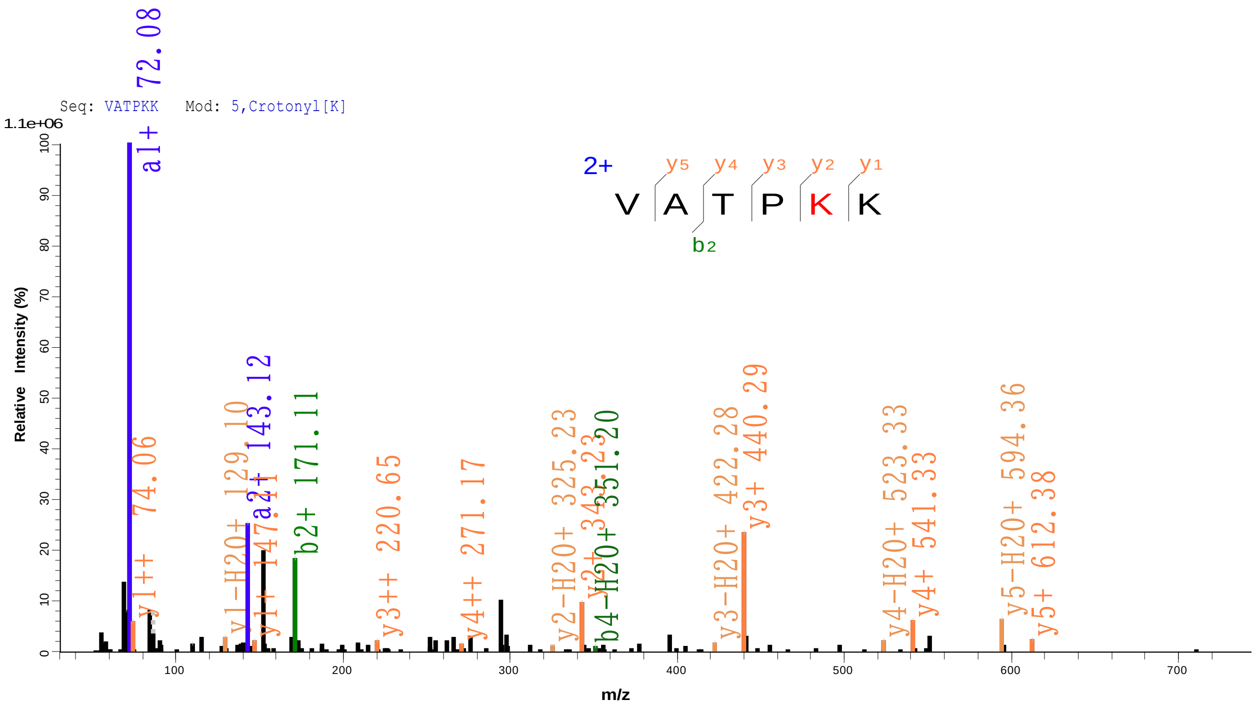

Seq: VATPKK Mod: 5,Crotonyl[K]
1.1e+06
2+
V A T P K K
y5 y4 y3 y2 y1
b2
Relative Intensity (%)
m/z
a1+ 72.08
y1++ 74.06
y1-H2O+ 129.10
a2+ 143.12
y1+ 147.11
b2+ 171.11
y3++ 220.65
y4++ 271.17
y2-H2O+ 325.23
y2+ 343.23
b4-H2O+ 351.20
y3-H2O+ 422.28
y3+ 440.29
y4-H2O+ 523.33
y4+ 541.33
y5-H2O+ 594.36
y5+ 612.38

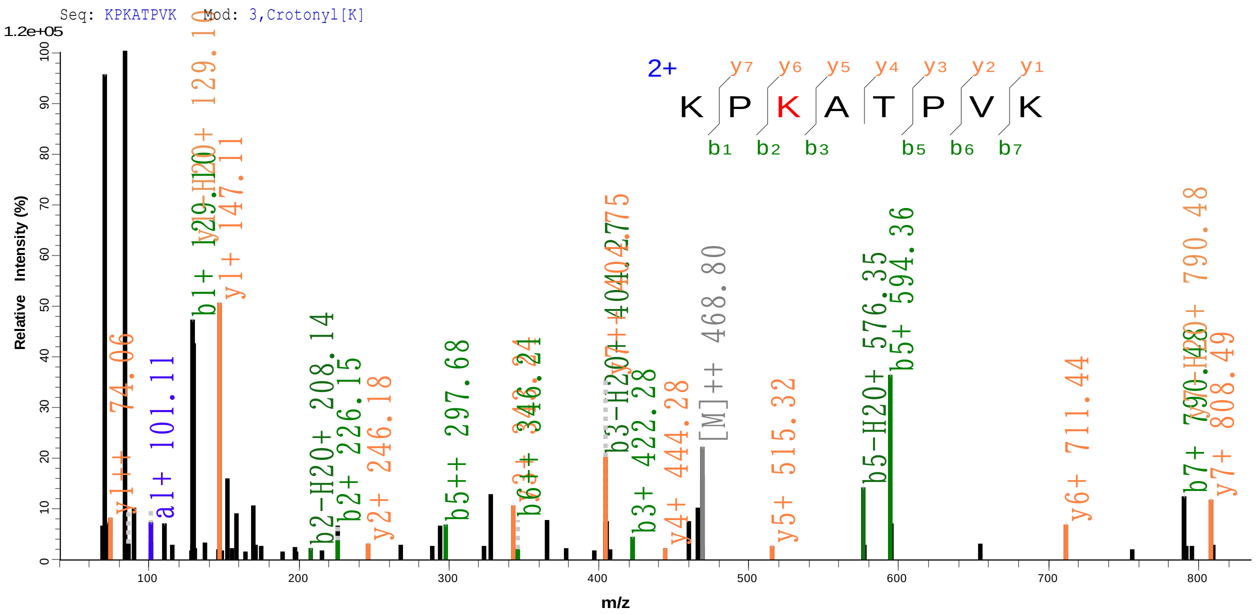
Seq: KPKATPVK
Mod: 3,Crotonyl[K]
1.2e+05
Relative Intensity (%)
m/z
2+
K P K A T P V K
y7 y6 y5 y4 y3 y2 y1
b1 b2 b3 b5 b6 b7
y1++ 74.06
a1+ 101.11
b1+ 129.10
y1+ 147.11
b2-H2O+ 208.14
b2+ 226.15
y2+ 246.18
b5++ 297.68
b3-H2O+ 404.27
b3+ 422.28
y4+ 444.28
[M]++ 468.80
y5+ 515.32
b5-H2O+ 576.35
b5+ 594.36
y6+ 711.44
y7+ 808.49

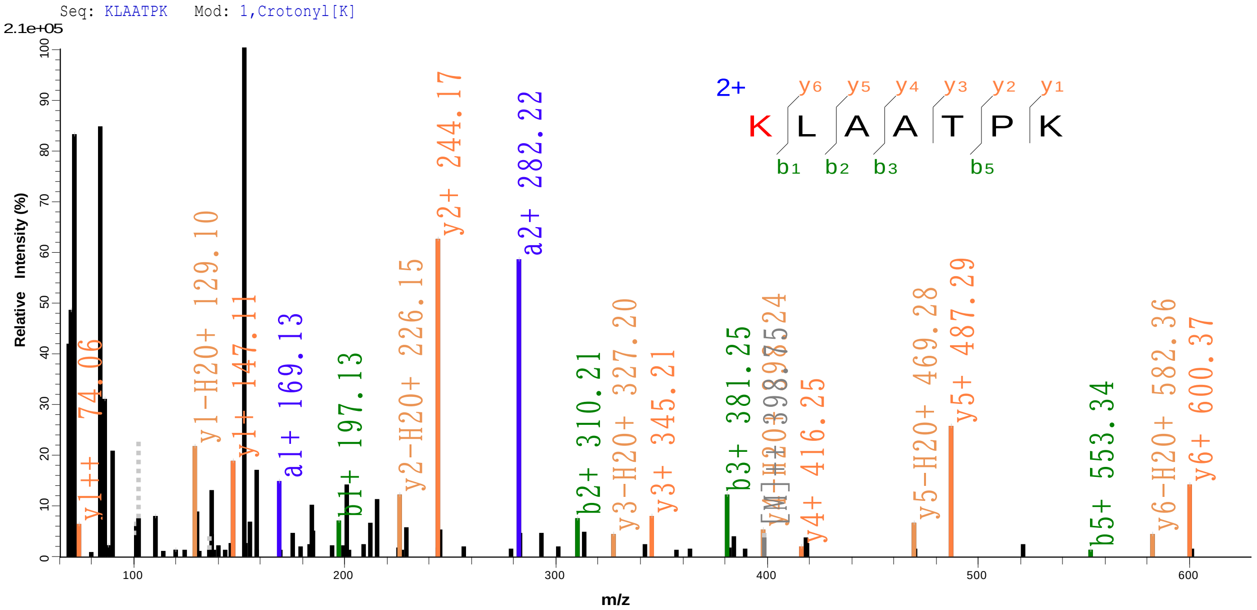
Seq: KLAATPK
Mod: 1,Crotonyl[K]
2.1e+05
Relative Intensity (%)
m/z
2+
K L A A T P K
y6 y5 y4 y3 y2 y1
b1 b2 b3 b5
y1++ 74.06
y1-H2O+ 129.10
y1+ 147.11
a1+ 169.13
b1+ 197.13
y2-H2O+ 226.15
y2+ 244.17
a2+ 282.22
b2+ 310.21
y3-H2O+ 327.20
y3+ 345.21
b3+ 381.25
y4+ 416.25
y5-H2O+ 469.28
y5+ 487.29
b5+ 553.34
y6-H2O+ 582.36
y6+ 600.37

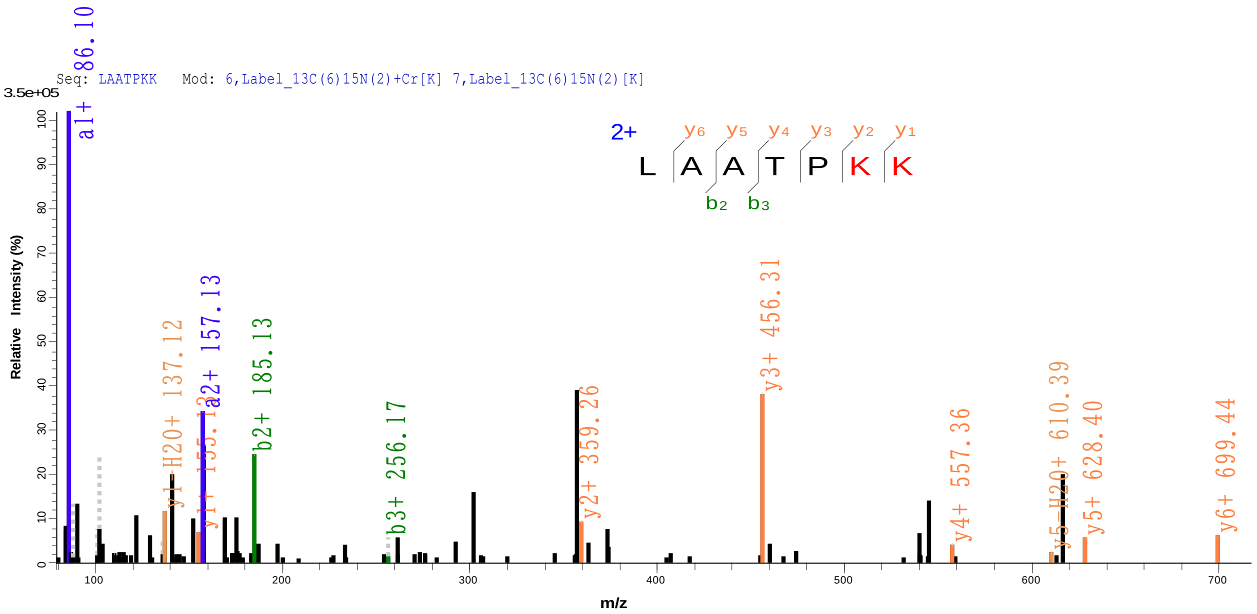
Seq: LAATPKK Mod: 6,Label_13C(6)15N(2)+Cr[K] 7,Label_13C(6)15N(2)[K]
3.5e+05
Relative Intensity (%)
m/z
2+
L A A T P K K
y6 y5 y4 y3 y2 y1
b2 b3
a1+ 86.10
a2+ 157.13
y1-H2O+ 137.12
b2+ 185.13
b3+ 256.17
y2+ 359.26
y3+ 456.31
y4+ 557.36
y5-H2O+ 610.39
y5+ 628.40
y6+ 699.44

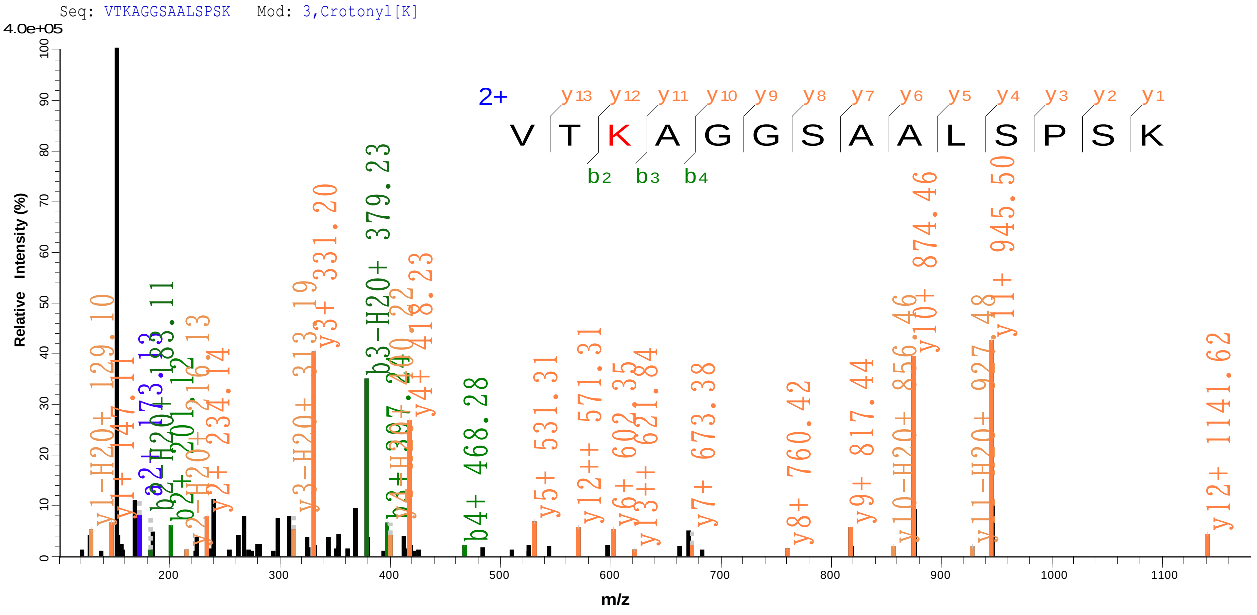
Seq: VTKAGGSAALSPSK Mod: 3,Crotonyl[K]
4.0e+05
Relative Intensity (%)
m/z
2+
V T K A G G S A A L S P S K
y13 y12 y11 y10 y9 y8 y7 y6 y5 y4 y3 y2 y1
b2 b3 b4
y1-H2O+ 129.10
y1+ 147.11
a2+ 173.13
y2+ 234.14
y3-H2O+ 313.19
y3+ 331.20
b3-H2O+ 379.23
y4+ 418.23
b4+ 468.28
y5+ 531.31
y12++ 571.31
y6+ 602.35
y13++ 621.84
y7+ 673.38
y8+ 760.42
y9+ 817.44
y10-H2O+ 856.46
y10+ 874.46
y11-H2O+ 927.48
y11+ 945.50
y12+ 1141.62

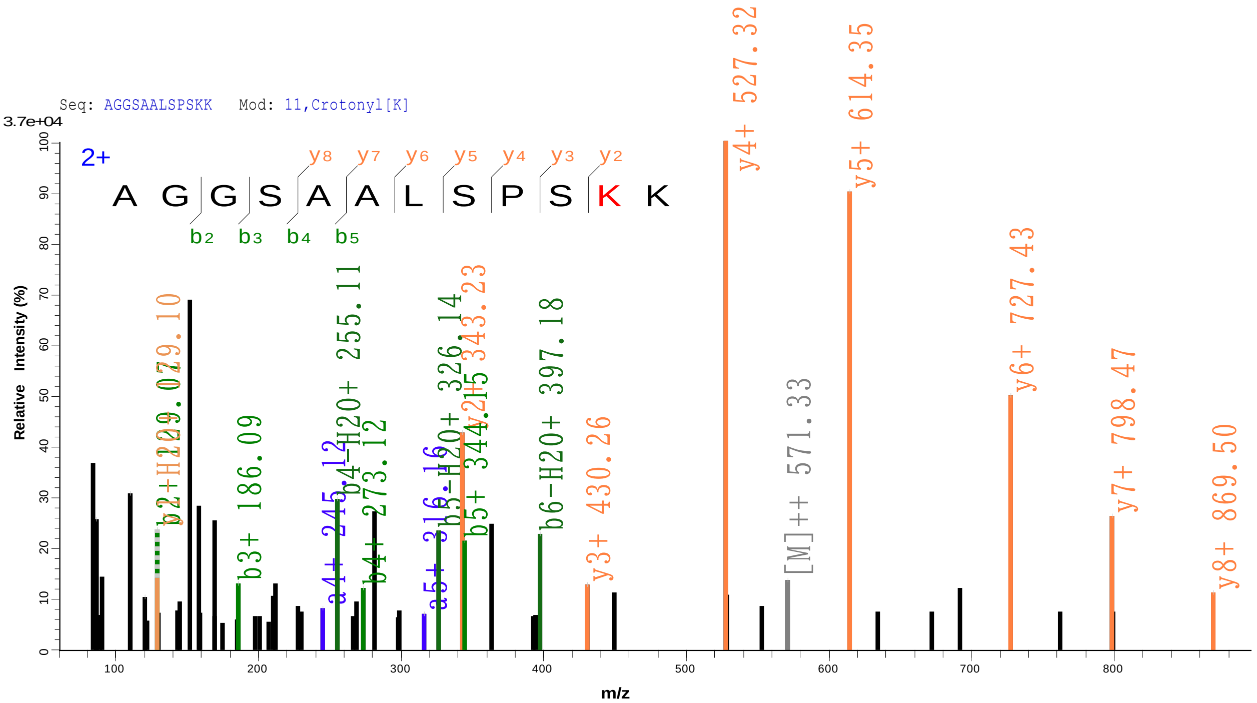
Seq: AGGSAALSPSKK Mod: 11,Crotonyl[K]
3.7e+04
2+
A G G S A A L S P S K K
y8 y7 y6 y5 y4 y3 y2
b2 b3 b4 b5
Relative Intensity (%)
m/z
y4+ 527.32
y5+ 614.35
y6+ 727.43
y7+ 798.47
y8+ 869.50
[M]++ 571.33
y3+ 430.26
b6-H2O+ 397.18
b3+ 186.09
b4-H2O+ 255.11
b4+ 273.12
a4+ 245.12
a5+ 316.16
b5+ 344.15
y3+ 343.23

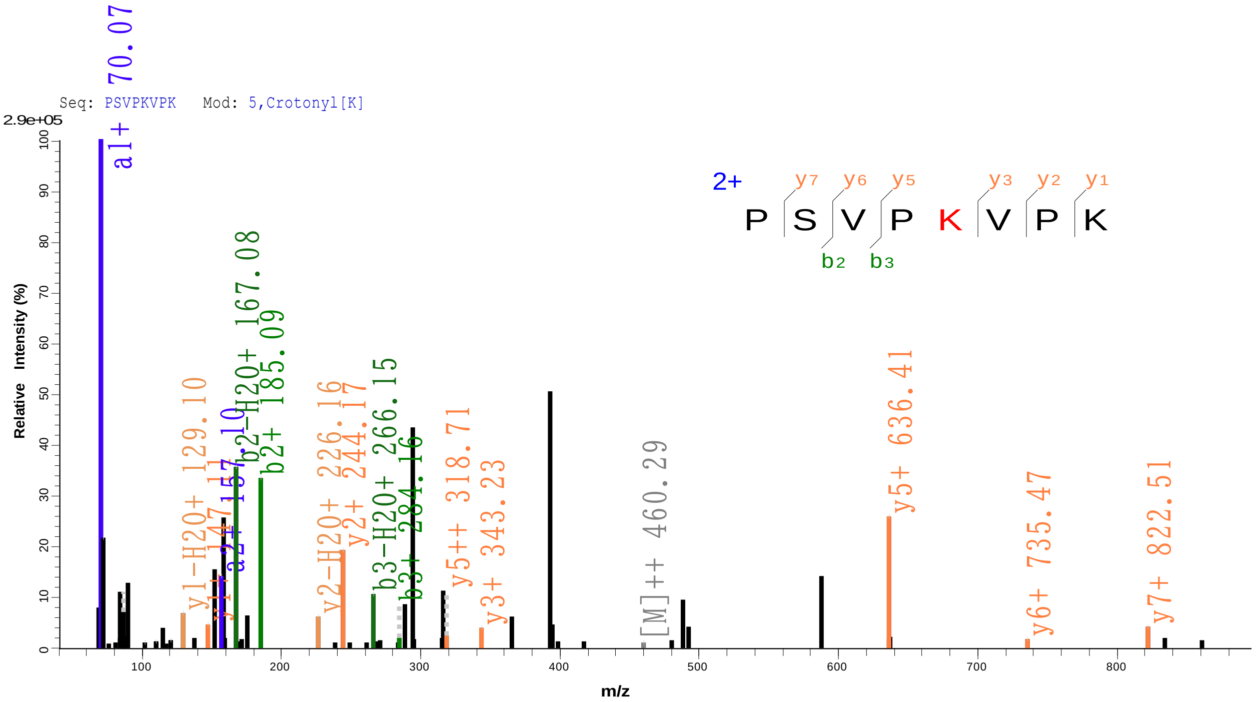
Seq: PSVPKVPK Mod: 5,Crotonyl[K]
2.9e+05
2+
P S V P K V P K
y7 y6 y5 y3 y2 y1
b2 b3
Relative Intensity (%)
m/z
a1+ 70.07
y1-H2O+ 129.10
b2-H2O+ 167.08
b2+ 185.09
y2-H2O+ 226.16
y2+ 244.17
b3-H2O+ 266.15
b3+ 284.16
y5++ 318.71
y3+ 343.23
[M]++ 460.29
y5+ 636.41
y6+ 735.47
y7+ 822.51

H2A

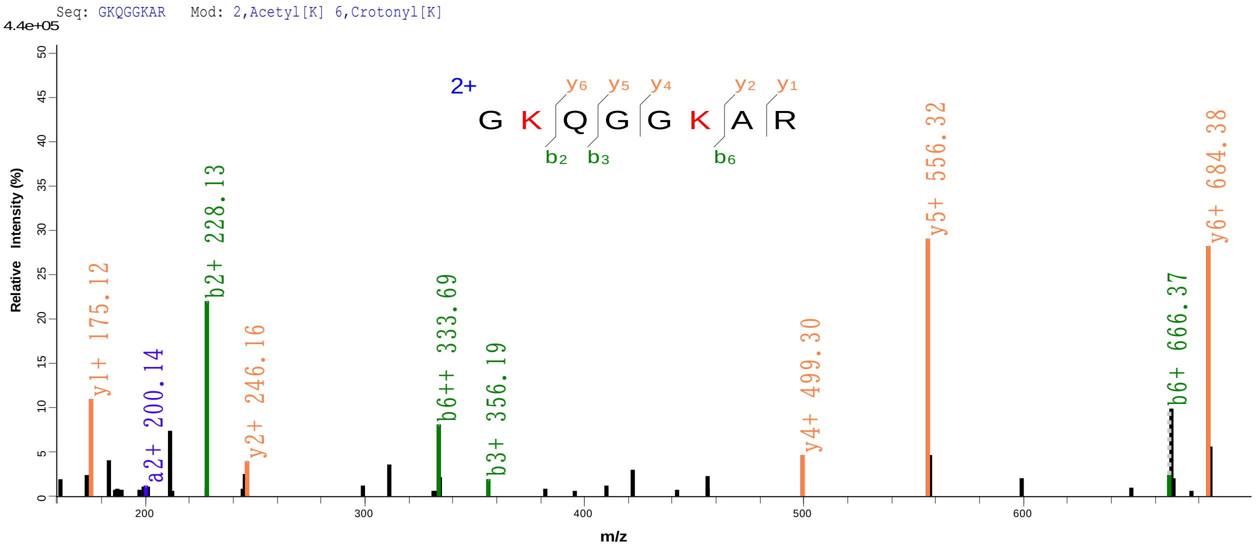
Seq: GKQGGKAR Mod: 2,Acetyl[K] 6,Crotonyl[K]
4.4e+05
Relative Intensity (%)
m/z
2+
G K Q G G K A R
y6 y5 y4 y2 y1
b2 b3 b6
y1+ 175.12
a2+ 200.14
b2+ 228.13
y2+ 246.16
b6++ 333.69
b3+ 356.19
y4+ 499.30
y5+ 556.32
b6+ 666.37
y6+ 684.38

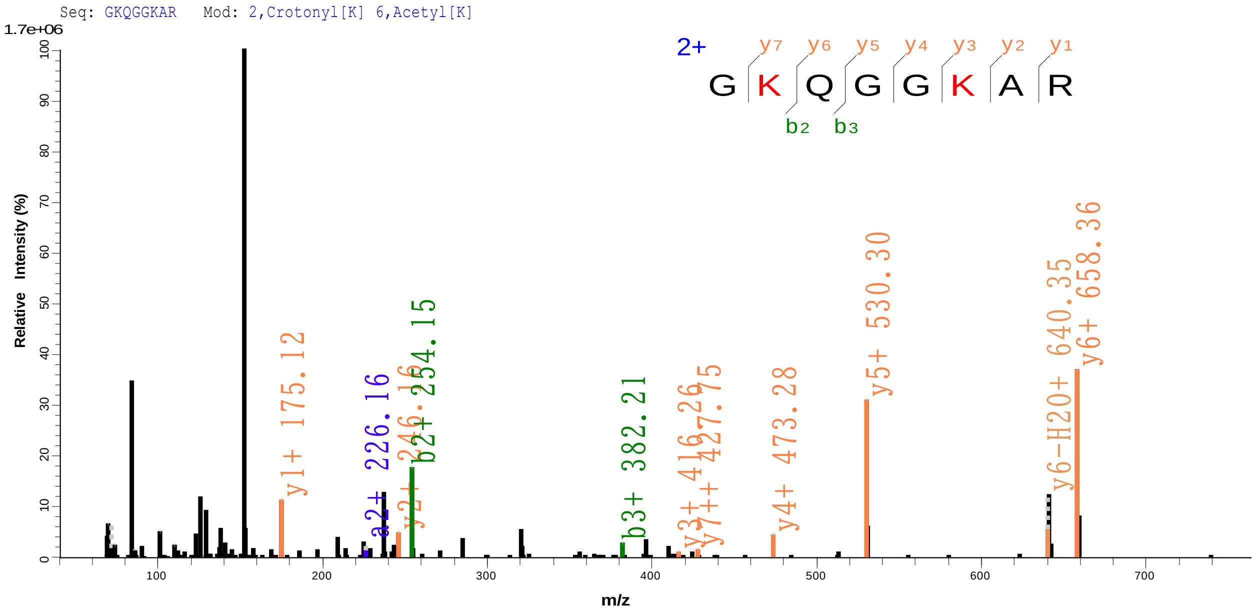
Seq: GKQGGKAR Mod: 2,Crotonyl[K] 6,Acetyl[K]
1.7e+06
Relative Intensity (%)
m/z
2+
G K Q G G K A R
y7 y6 y5 y4 y3 y2 y1
b2 b3
y1+ 175.12
a2+ 226.16
b2+ 254.15
b3+ 382.21
y4+ 473.28
y5+ 530.30
y6-H2O+ 640.35
y6+ 658.36

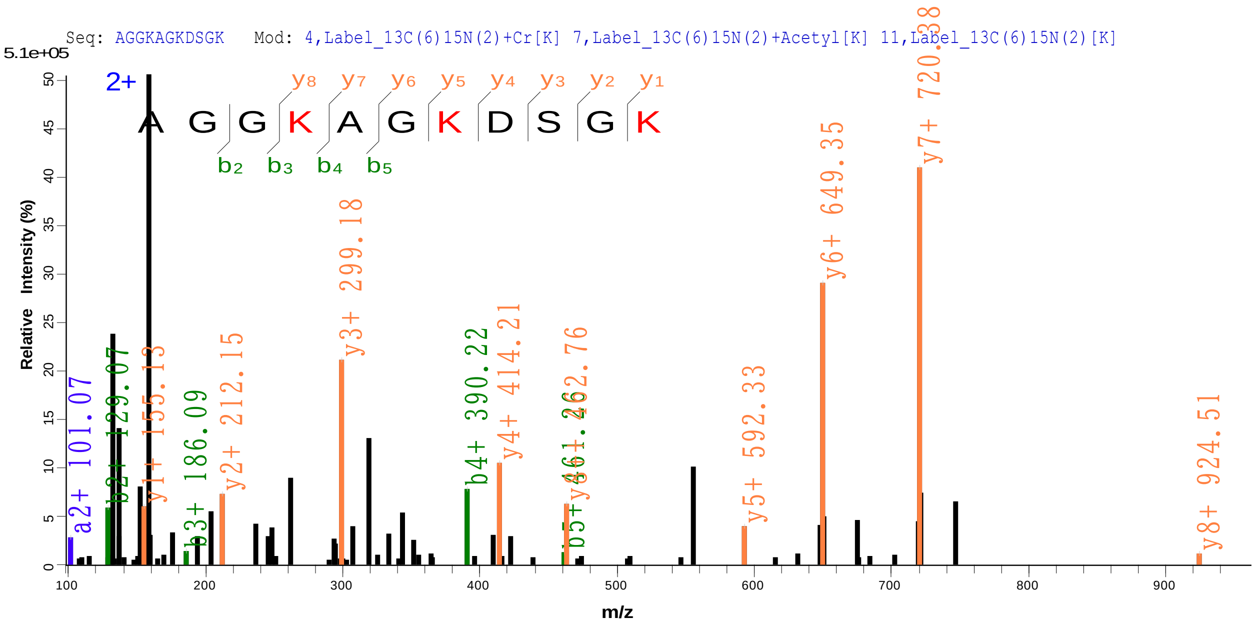

Seq: AGGKAGKDSGK Mod: 4,Label_13C(6)15N(2)+Cr[K] 7,Label_13C(6)15N(2)+Acetyl[K] 11,Label_13C(6)15N(2)[K]
5.1e+05
2+
A G G K A G K D S G K
Relative Intensity (%)
m/z
a2+ 101.07
b2+ 129.07
y1+ 155.13
b3+ 186.09
y2+ 212.15
y3+ 299.18
b4+ 390.22
y4+ 414.21
y5+ 592.33
y6+ 649.35
y7+ 720.38
y8+ 924.51

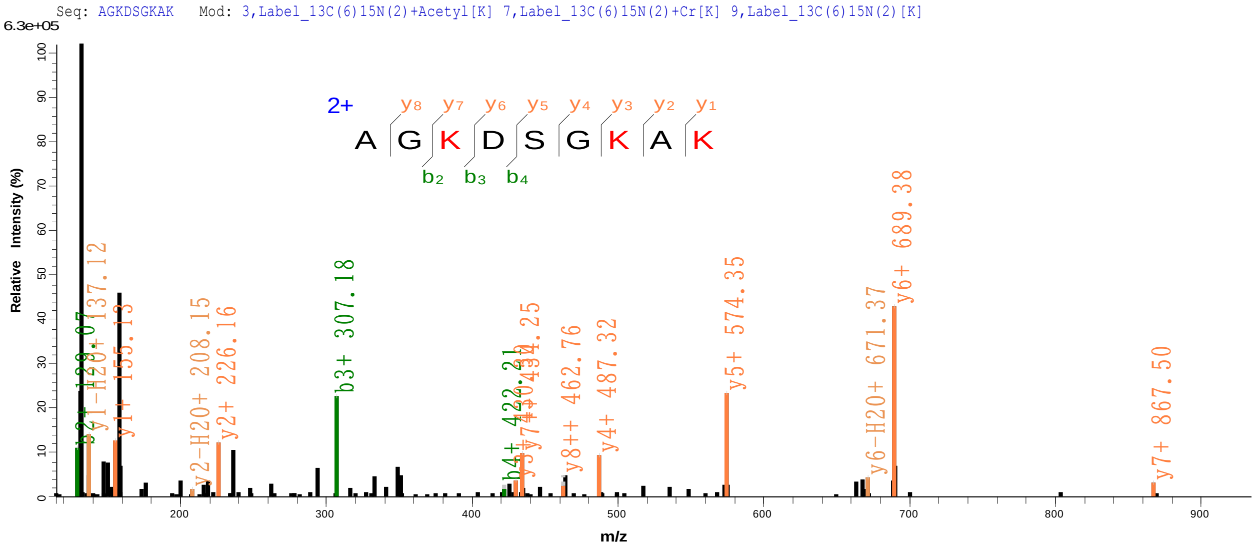

Seq: AGKDSGKAK Mod: 3,Label_13C(6)15N(2)+Acetyl[K] 7,Label_13C(6)15N(2)+Cr[K] 9,Label_13C(6)15N(2)[K]
6.3e+05
2+
A G K D S G K A K
Relative Intensity (%)
m/z
y1-H2O+ 137.12
y1+ 155.13
y2-H2O+ 208.15
y2+ 226.16
b3+ 307.18
y8++ 462.76
y4+ 487.32
y5+ 574.35
y6-H2O+ 671.37
y6+ 689.38
y7+ 867.50

Seq: DSGKAKTK Mod: 4,Acetyl[K] 6,Crotonyl[K]

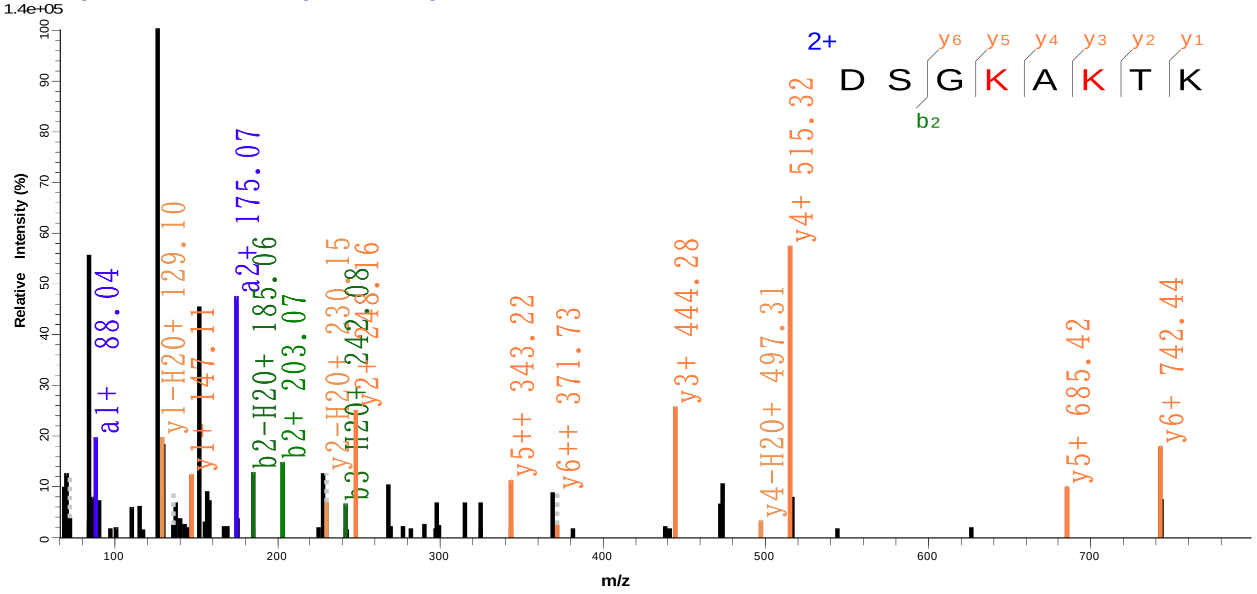

Seq: TKAVSR Mod: 2,Crotonyl[K]

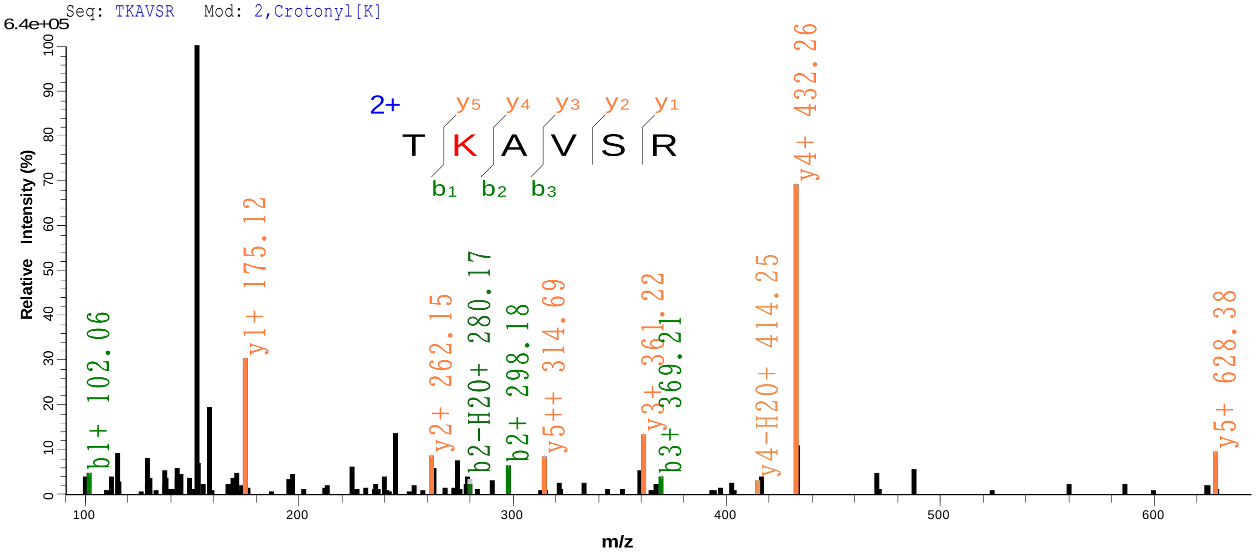

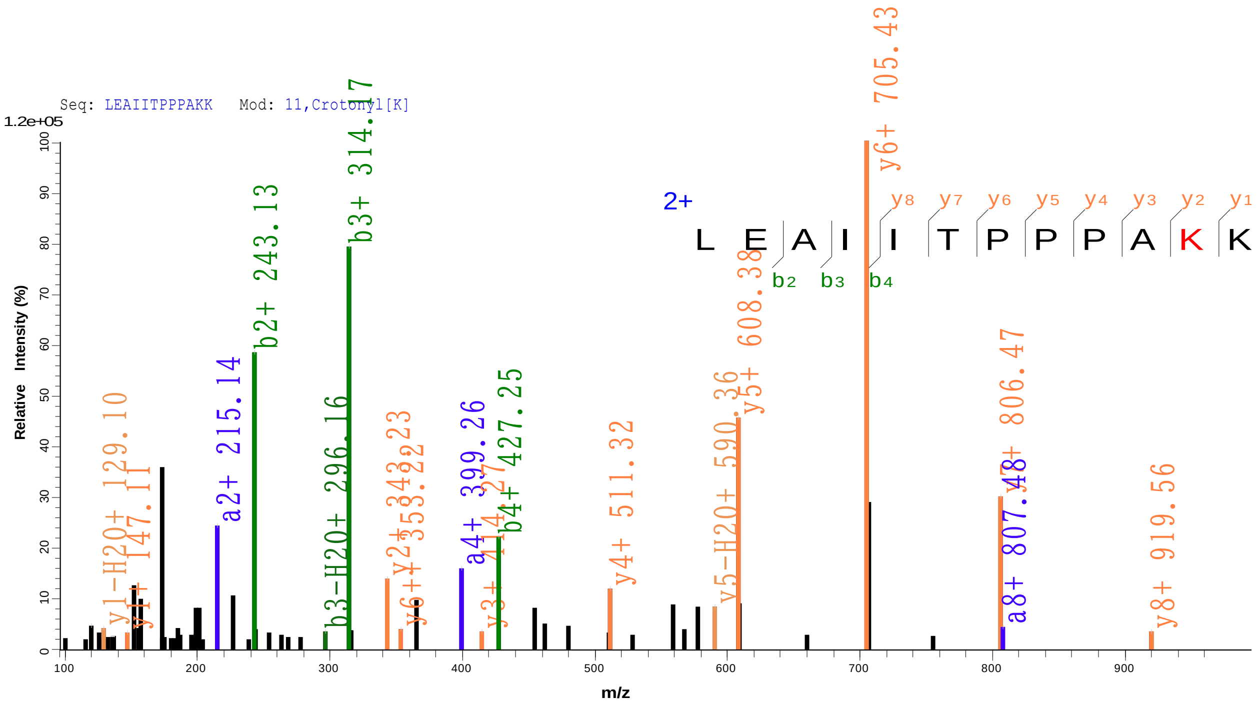
Seq: LEAIITPPPAKK Mod: 11,Crotonyl[K]
1.2e+05
Relative Intensity (%)
m/z
2+
L E A I I T P P P A K K
y8 y7 y6 y5 y4 y3 y2 y1
b2 b3 b4
y6+ 705.43
b3+ 314.17
b2+ 243.13
y5+ 608.38
a2+ 215.14
y1-H2O+ 129.10
y1+ 147.11
b3-H2O+ 296.16
a4+ 399.26
b4+ 427.25
y4+ 511.32
y5-H2O+ 590.36
y7+ 806.47
a8+ 807.48
y8+ 919.56

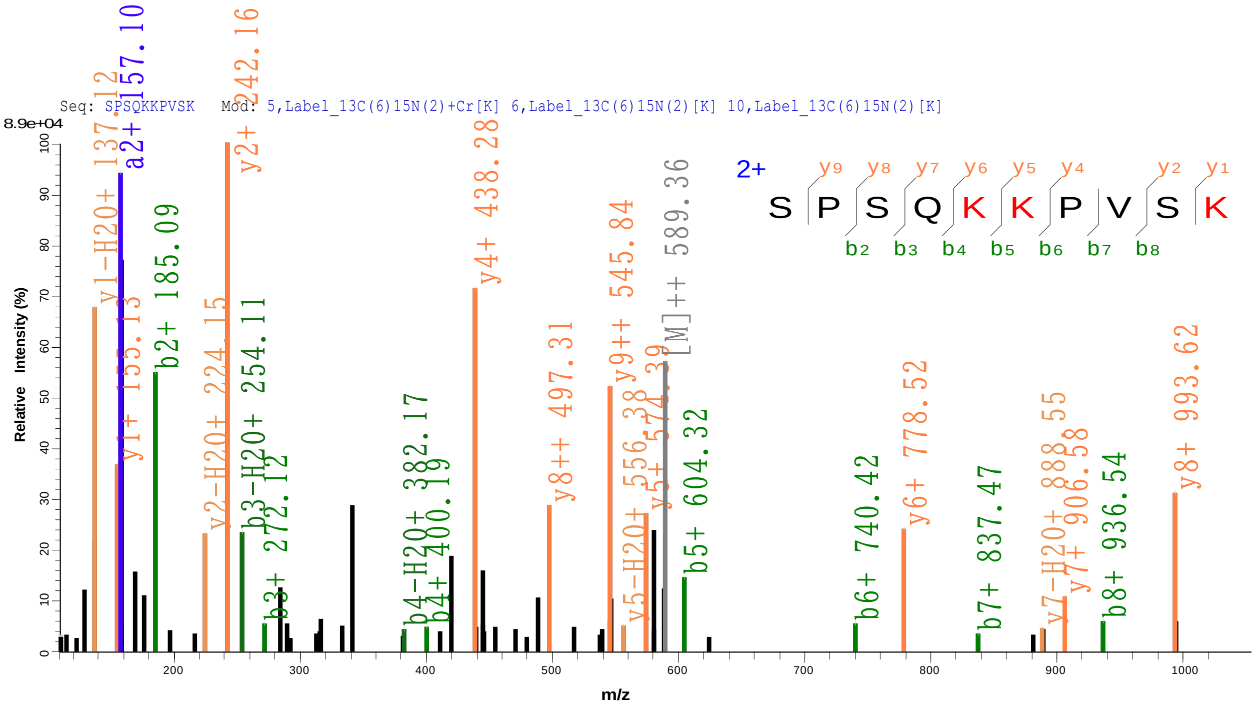
Seq: SPSQKKPVSK Mod: 5,Label_13C(6)15N(2)+Cr[K] 6,Label_13C(6)15N(2)[K] 10,Label_13C(6)15N(2)[K]
8.9e+04
Relative Intensity (%)
m/z
2+
S P S Q K K P V S K
y9 y8 y7 y6 y5 y4 y2 y1
b2 b3 b4 b5 b6 b7 b8
y2+ 242.16
a2+ 157.10
y1-H2O+ 137.12
y1+ 155.13
b2+ 185.09
y2-H2O+ 224.15
b3-H2O+ 254.11
b3+ 272.12
b4-H2O+ 382.17
b4+ 400.19
y4+ 438.28
y8++ 497.31
y9++ 545.84
[M]++ 589.36
b5+ 604.32
b6+ 740.42
y6+ 778.52
b7+ 837.47
y7-H2O+ 888.55
y7+ 906.58
b8+ 936.54
y8+ 993.62

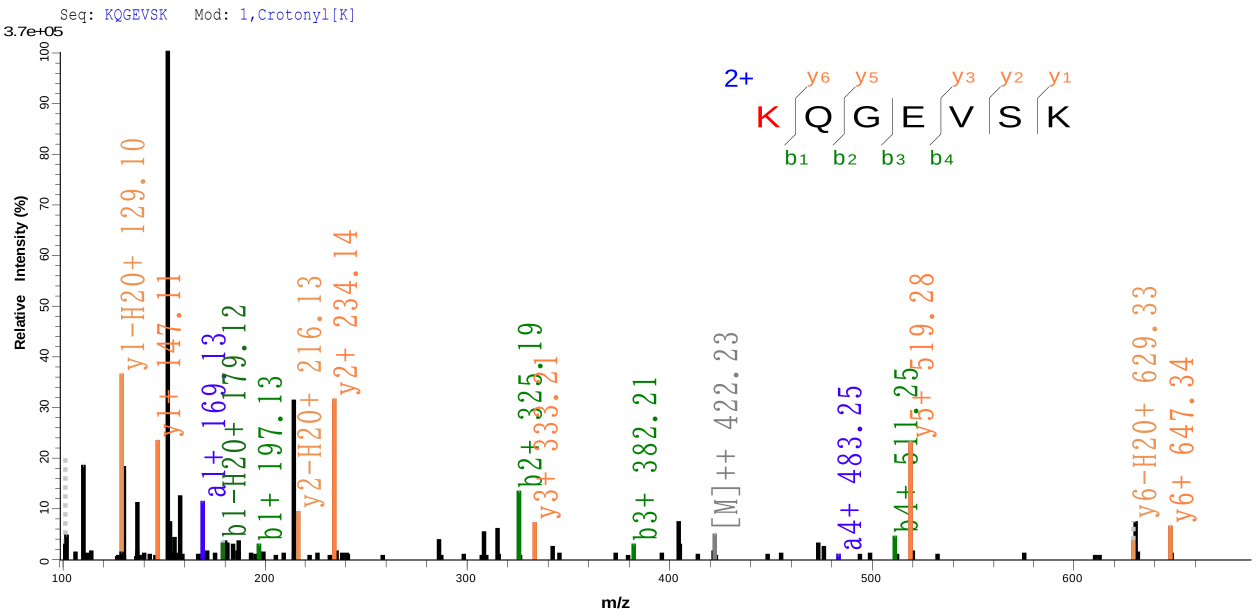
Seq: KQGEVSK Mod: 1,Crotonyl[K]
3.7e+05
Relative Intensity (%)
m/z
2+
K Q G E V S K
y6 y5 y3 y2 y1
b1 b2 b3 b4
y1-H2O+ 129.10
y1+ 147.11
a1+ 169.13
b1-H2O+ 179.12
b1+ 197.13
y2-H2O+ 216.13
y2+ 234.14
b2+ 325.19
y3+ 333.21
b3+ 382.21
[M]++ 422.23
a4+ 483.25
b4+ 511.25
y5+ 519.28
y6-H2O+ 629.33
y6+ 647.34

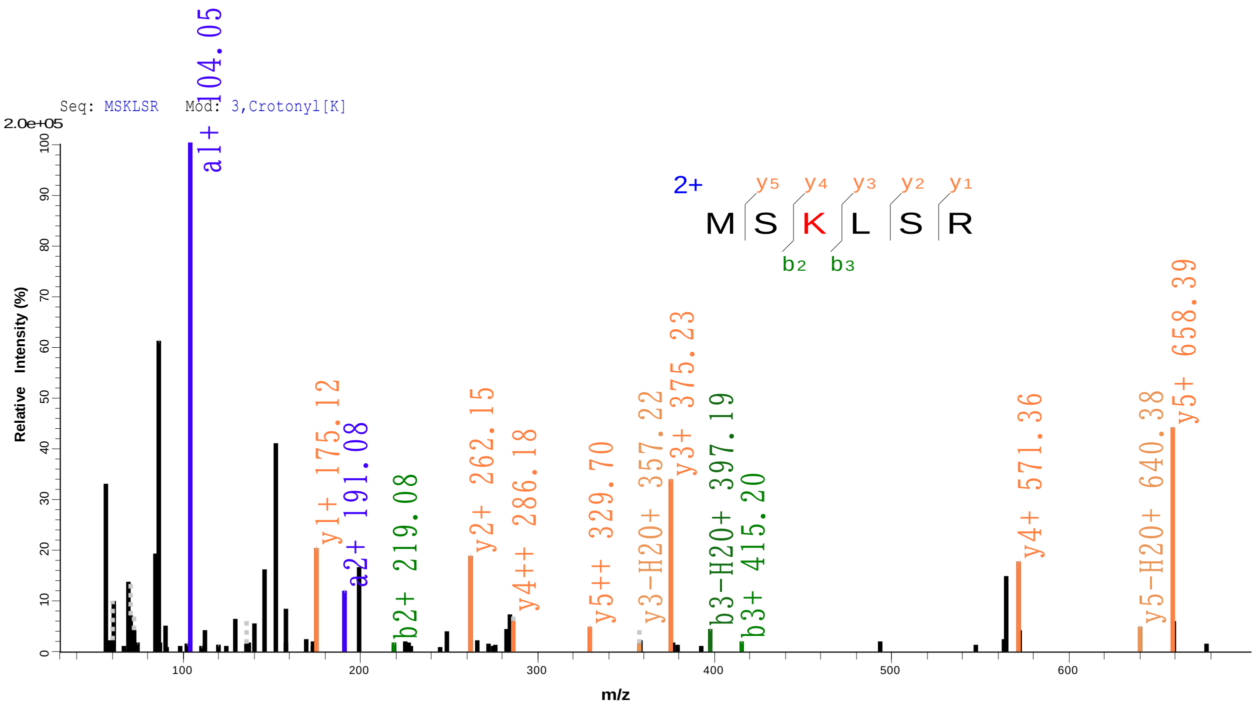
Seq: MSKLSR Mod: 3,Crotonyl[K]
2.0e+05
Relative Intensity (%)
m/z
2+
M S K L S R
y5 y4 y3 y2 y1
b2 b3
a1+ 104.05
y1+ 175.12
a2+ 191.08
b2+ 219.08
y2+ 262.15
y4++ 286.18
y5++ 329.70
y3-H2O+ 357.22
y3+ 375.23
b3-H2O+ 397.19
b3+ 415.20
y4+ 571.36
y5-H2O+ 640.38
y5+ 658.39

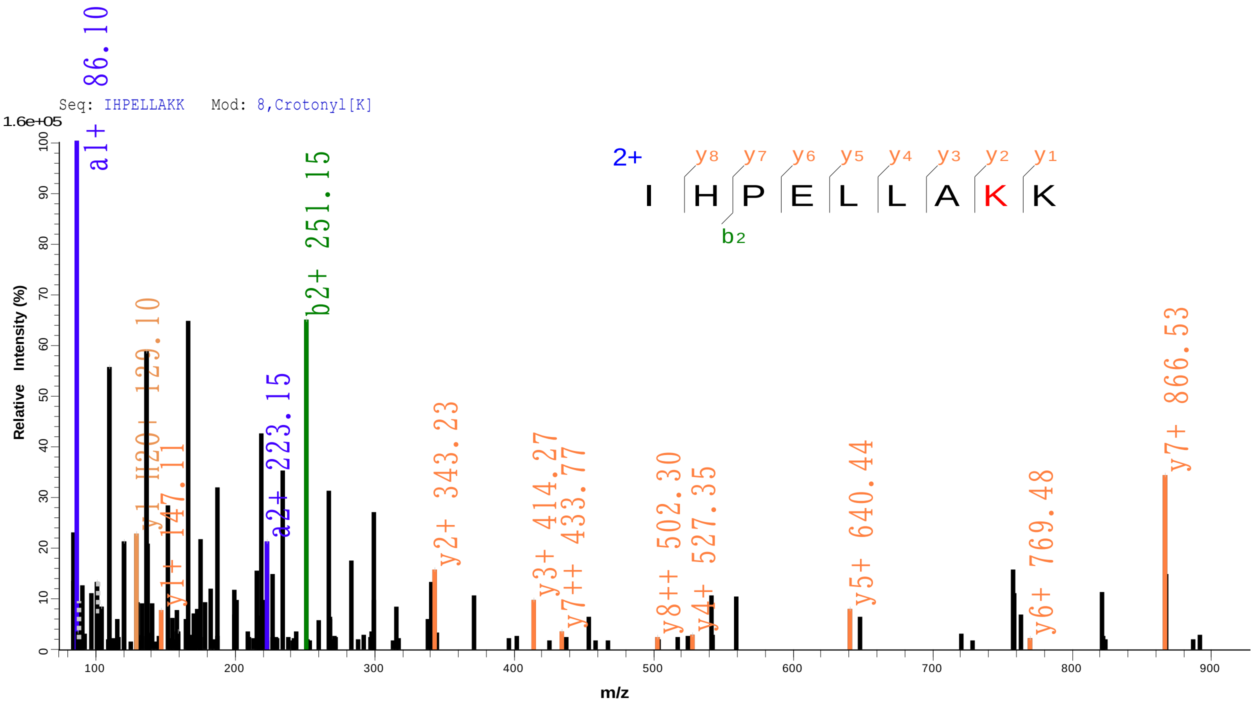
Seq: IHPELLAKK Mod: 8,Crotonyl[K]
1.6e+05
Relative Intensity (%)
m/z
2+
I H P E L L A K K
y8 y7 y6 y5 y4 y3 y2 y1
b2
a1+ 86.10
b2+ 251.15
a2+ 223.15
y1+ 147.11
y2+ 343.23
y3+ 414.27
y7++ 433.77
y8++ 502.30
y4+ 527.35
y5+ 640.44
y6+ 769.48
y7+ 866.53

H2B

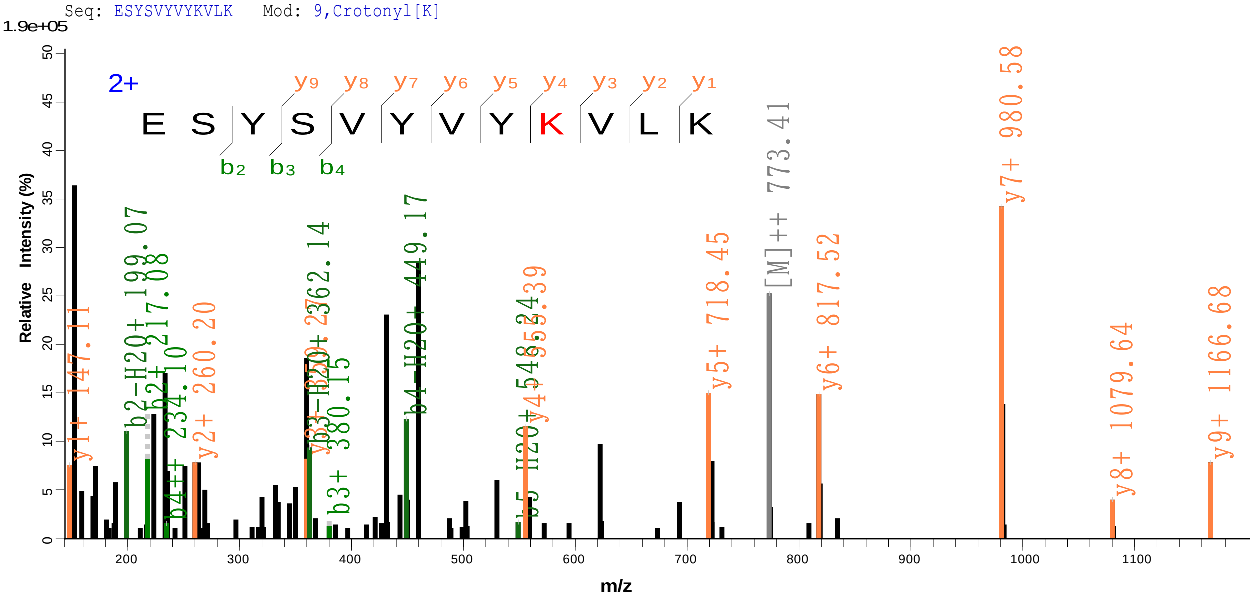
Seq: ESYSVYVYKVLK Mod: 9,Crotonyl[K]
1.9e+05
Relative Intensity (%)
m/z
2+
E S Y S V Y V Y K V L K
y9 y8 y7 y6 y5 y4 y3 y2 y1
b2 b3 b4
y1+ 147.11
b2-H2O+ 199.07
b2+ 217.08
b4++ 234.10
y2+ 260.20
b3-H2O+ 362.14
b3+ 380.15
b4-H2O+ 449.17
y5+ 718.45
[M]++ 773.41
y6+ 817.52
y7+ 980.58
y8+ 1079.64
y9+ 1166.68

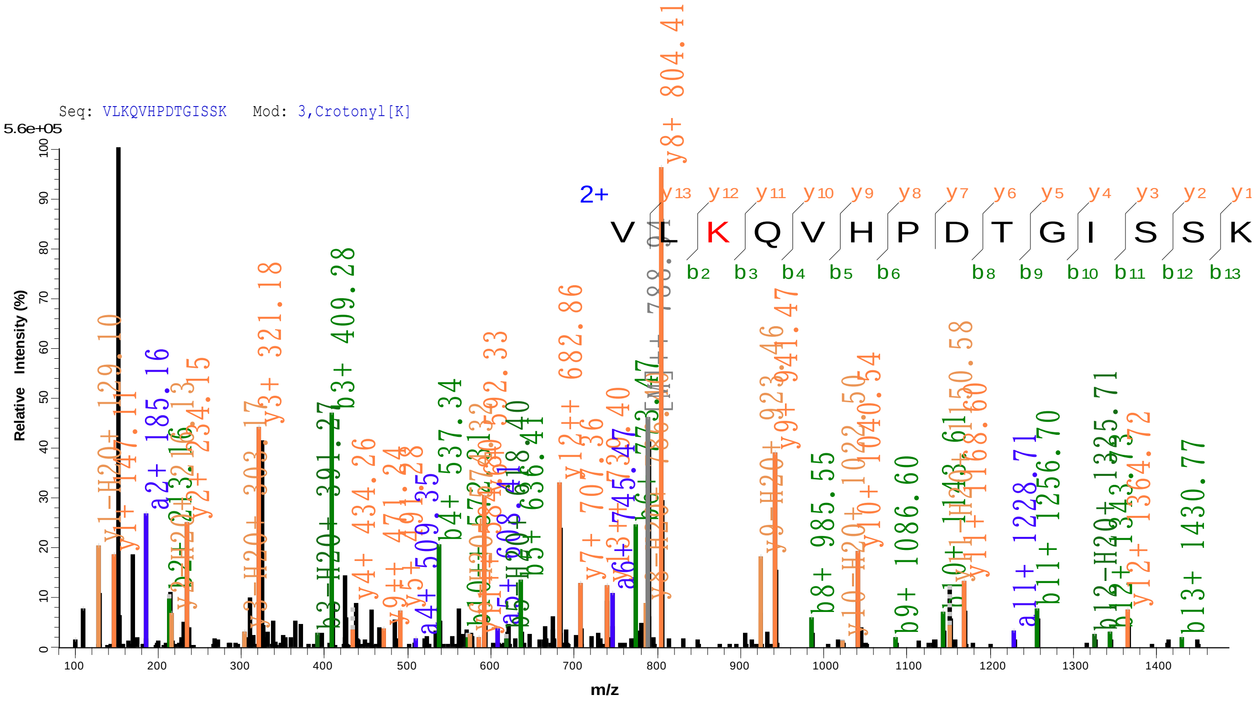
Seq: VLKQVHPDTGISSK Mod: 3,Crotonyl[K]
5.6e+05
Relative Intensity (%)
m/z
2+
V L K Q V H P D T G I S S K
y8+ 804.41
b3+ 409.28
y3+ 321.18
a2+ 185.16
b4+ 537.34
y12++ 682.86
b8+ 985.55
b9+ 1086.60
a11+ 1228.71
b11+ 1256.70
b13+ 1430.77

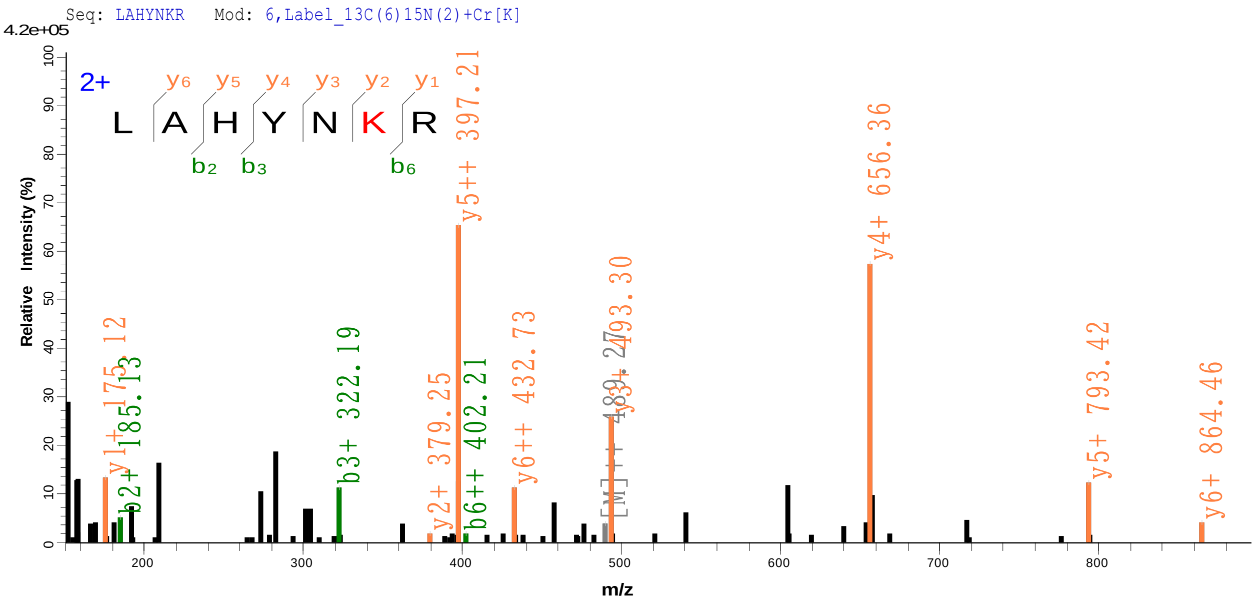
Seq: LAHYNKR Mod: 6,Label_13C(6)15N(2)+Cr[K]
4.2e+05
Relative Intensity (%)
m/z
2+
L A H Y N K R
y5++ 397.21
y4+ 656.36
b3+ 322.19
y2+ 379.25
b6++ 402.21
y6++ 432.73
y5+ 793.42
y6+ 864.46

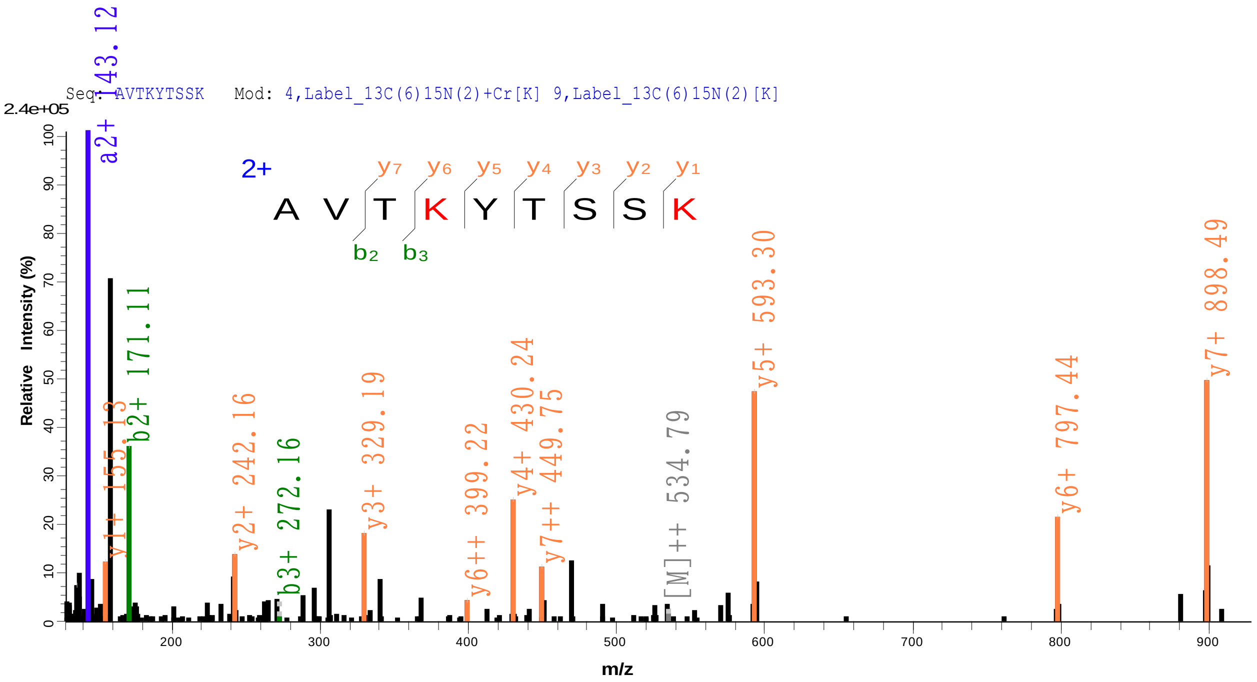
Seq: AVTKYTSSK Mod: 4,Label_13C(6)15N(2)+Cr[K] 9,Label_13C(6)15N(2)[K]
2.4e+05
Relative Intensity (%)
2+
A V T K Y T S S K
y7 y6 y5 y4 y3 y2 y1
b2 b3
a2+ 143.12
b2+ 171.11
y2+ 242.16
b3+ 272.16
y3+ 329.19
y6++ 399.22
y4+ 430.24
y7++ 449.75
[M]++ 534.79
y5+ 593.30
y6+ 797.44
y7+ 898.49
m/z

H3

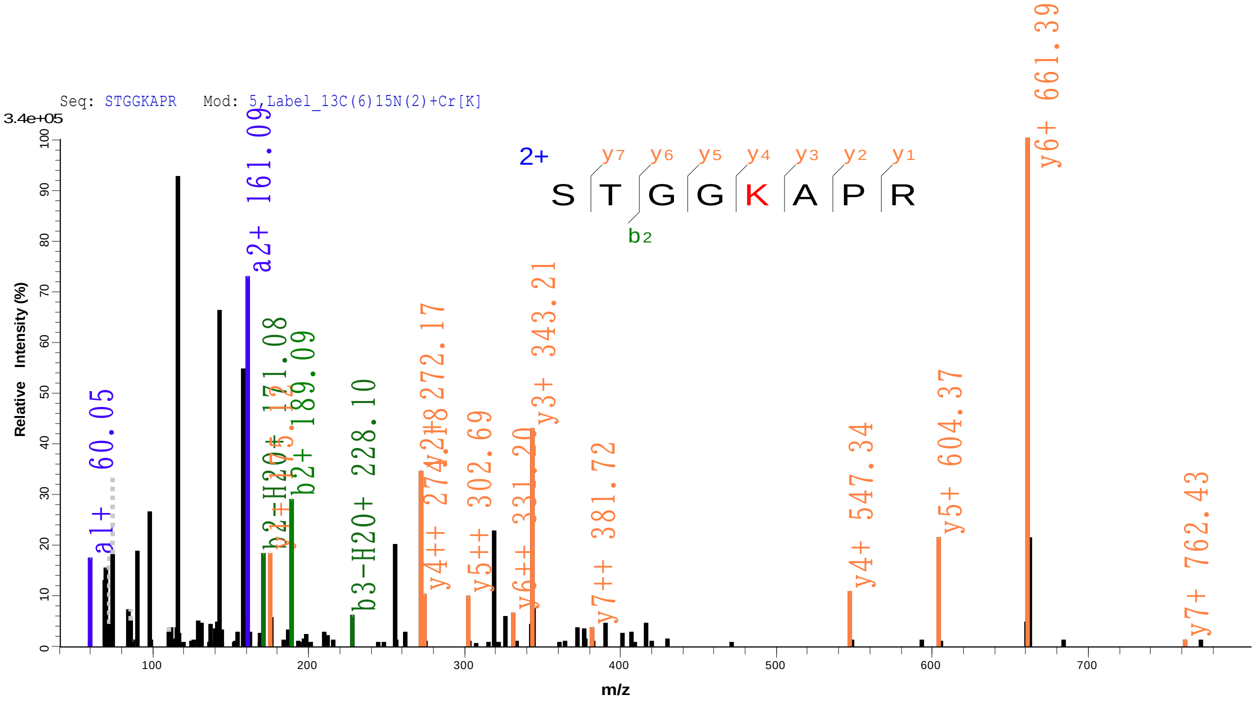
Seq: STGGKAPR Mod: 5,Label_13C(6)15N(2)+Cr[K]
3.4e+05
Relative Intensity (%)
2+
S T G G K A P R
y7 y6 y5 y4 y3 y2 y1
b2
a1+ 60.05
a2+ 161.09
b2+ 189.09
b3-H2O+ 228.10
y5++ 302.69
y3+ 343.21
y7++ 381.72
y4+ 547.34
y5+ 604.37
y6+ 661.39
y7+ 762.43
m/z

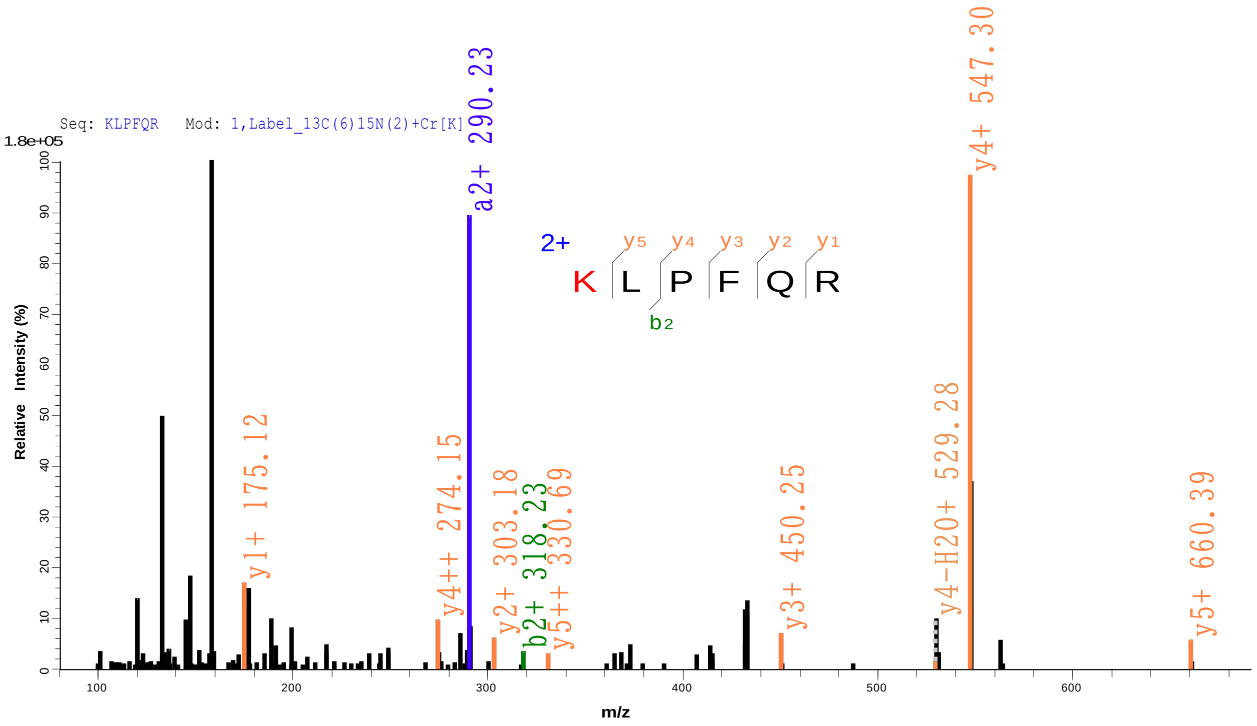
Seq: KLPFQR Mod: 1,Label_13C(6)15N(2)+Cr[K]
1.8e+05
Relative Intensity (%)
m/z
2+
K L P F Q R
y5 y4 y3 y2 y1
b2
a2+ 290.23
y4+ 547.30
y1+ 175.12
y4++ 274.15
y2+ 303.18
b2+ 318.23
y5++ 330.69
y3+ 450.25
y4-H2O+ 529.28
y5+ 660.39

H4

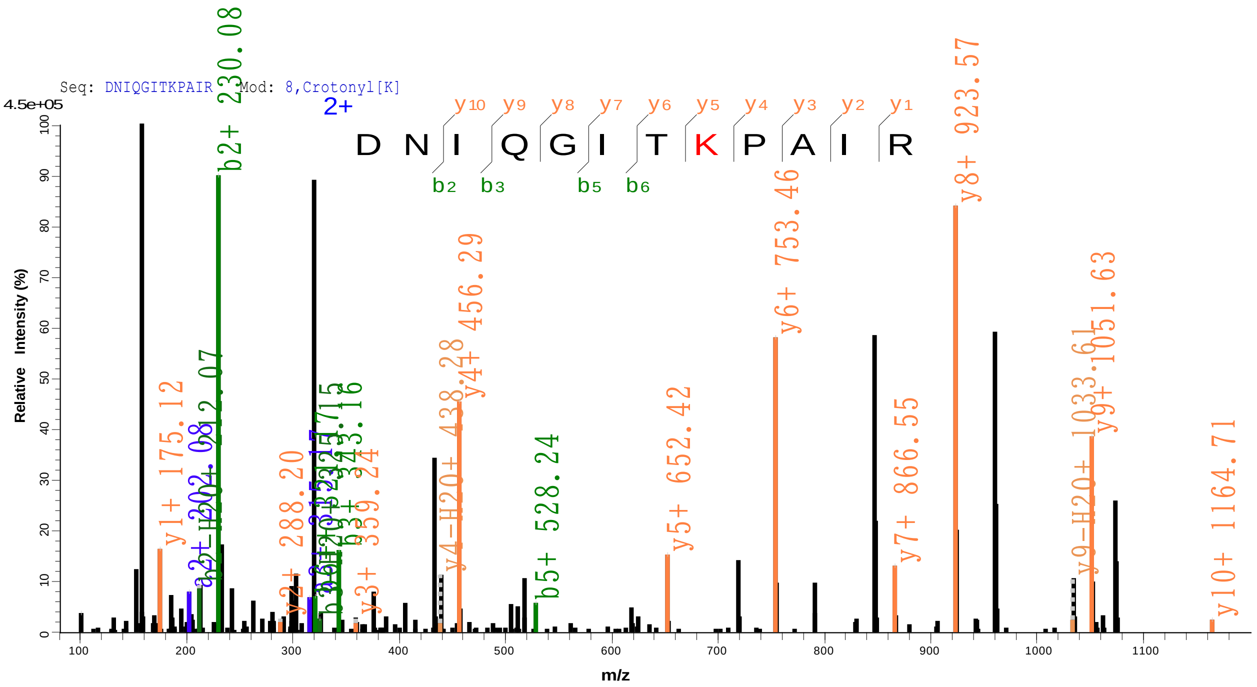
Seq: DNIQGITKPAIR Mod: 8,Crotonyl[K]
4.5e+05
Relative Intensity (%)
m/z
2+
D N I Q G I T K P A I R
y10 y9 y8 y7 y6 y5 y4 y3 y2 y1
b2 b3 b5 b6
b2+ 230.08
y8+ 923.57
y6+ 753.46
y4+ 456.29
y1+ 175.12
y2+ 288.20
y3+ 359.24
y4-H2O+ 438.28
b5+ 528.24
y5+ 652.42
y7+ 866.55
y9-H2O+ 1033.61
y9+ 1051.63
y10+ 1164.71

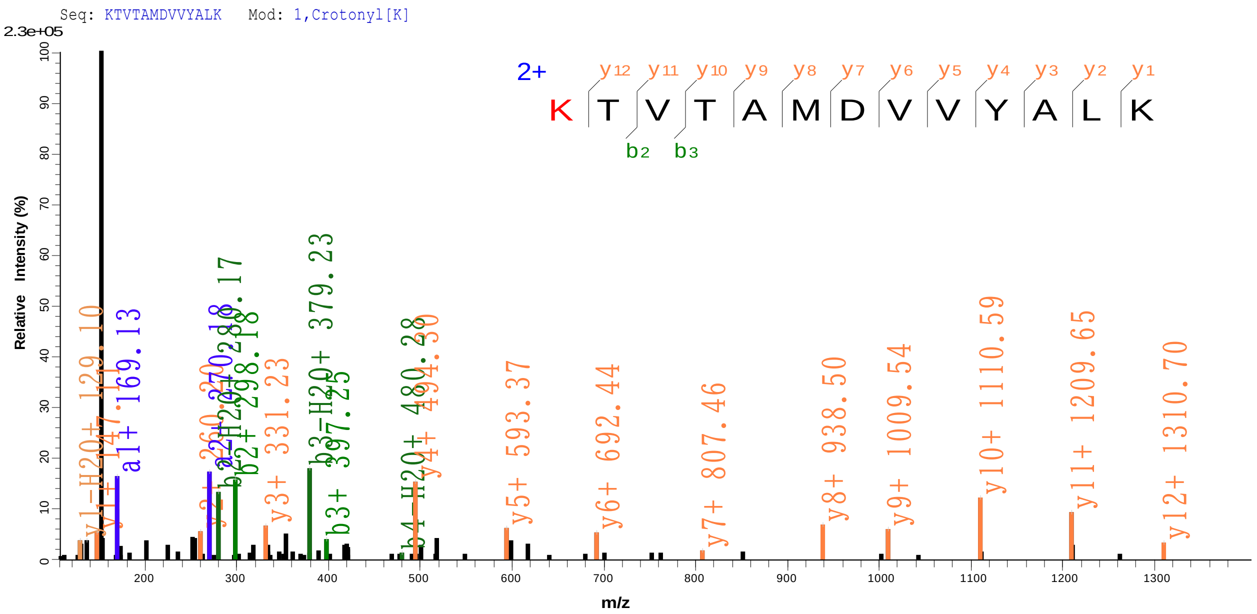
Seq: KTVTAMDVVYALK Mod: 1,Crotonyl[K]
2.3e+05
2+
K T V T A M D V V Y A L K
Relative Intensity (%)
m/z
y5+ 593.37
y6+ 692.44
y7+ 807.46
y8+ 938.50
y9+ 1009.54
y10+ 1110.59
y11+ 1209.65
y12+ 1310.70
b3-H2O+ 379.23
a1+ 169.13

C. Khib

H1

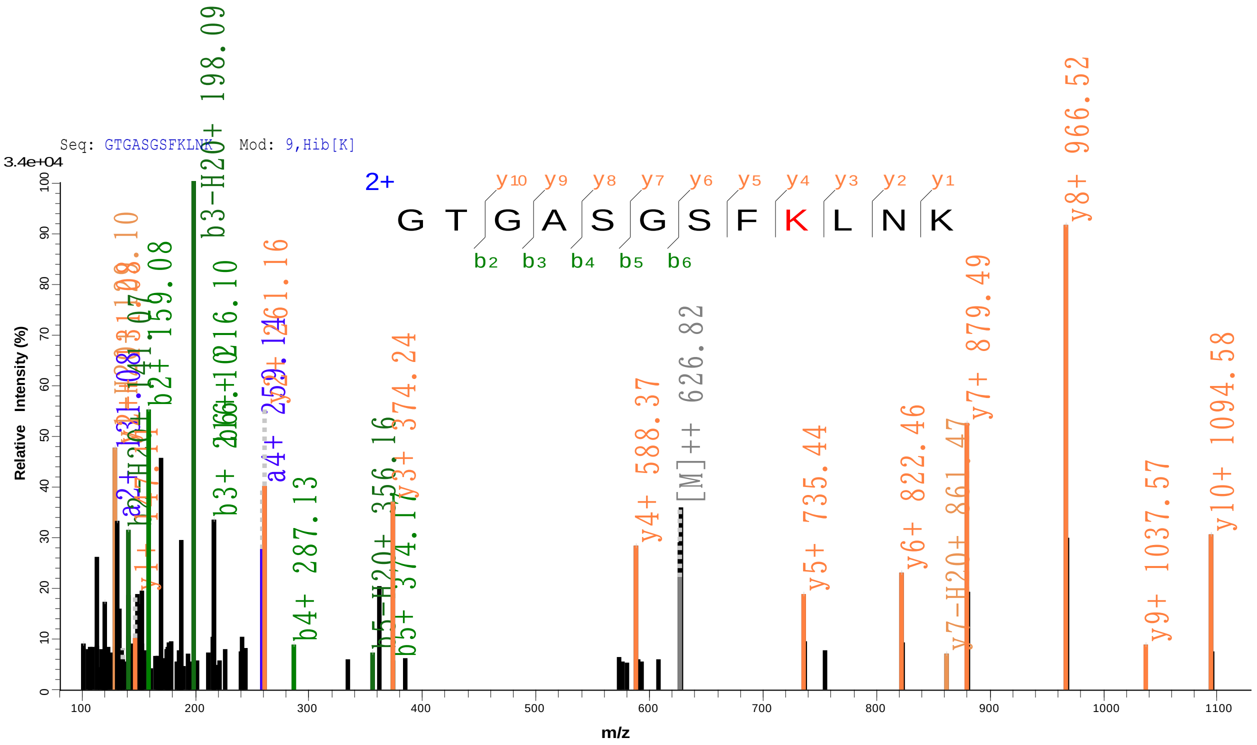
Seq: GTGASGSFKLNK Mod: 9,Hib[K]
3.4e+04
2+
G T G A S G S F K L N K
Relative Intensity (%)
m/z
b3-H2O+ 198.09
b2+ 159.08
b4+ 287.13
y4+ 588.37
[M]++ 626.82
y5+ 735.44
y6+ 822.46
y7+ 879.49
y8+ 966.52
y9+ 1037.57
y10+ 1094.58

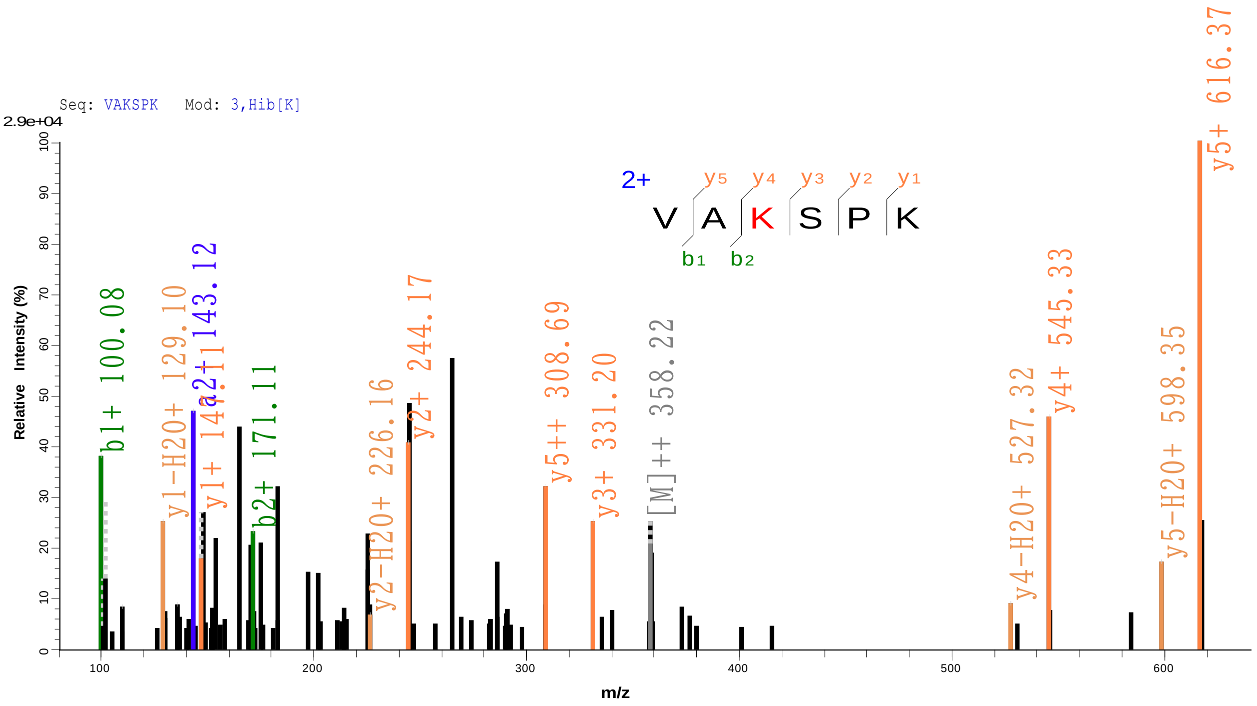

Seq: VAKSPK Mod: 3,Hib[K]
2.9e+04
Relative Intensity (%)
m/z
2+
V A K S P K
y5 y4 y3 y2 y1
b1 b2
b1+ 100.08
y1-H2O+ 129.10
a2+ 143.12
y1+ 147.11
b2+ 171.11
y2-H2O+ 226.16
y2+ 244.17
y5++ 308.69
y3+ 331.20
[M]++ 358.22
y4-H2O+ 527.32
y4+ 545.33
y5-H2O+ 598.35
y5+ 616.37

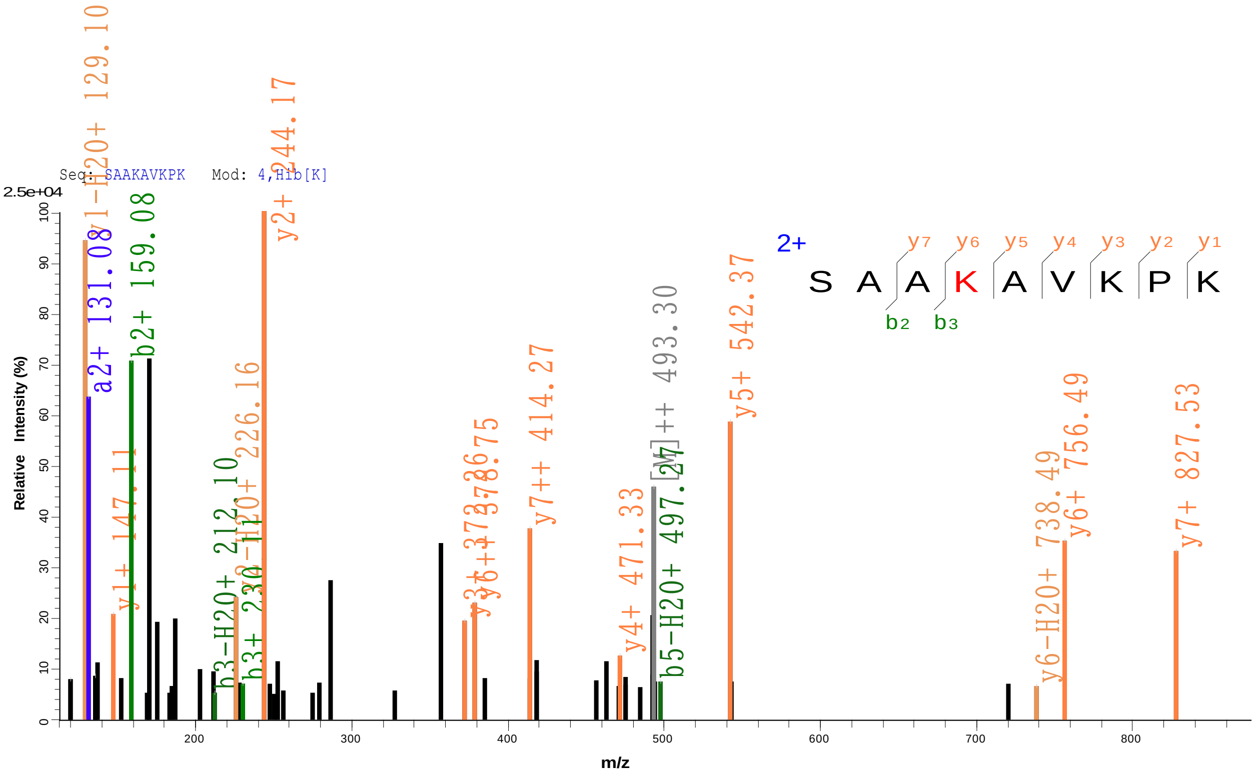

Seq: SAAKAVKPK Mod: 4,Hib[K]
2.5e+04
Relative Intensity (%)
m/z
2+
S A A K A V K P K
y7 y6 y5 y4 y3 y2 y1
b2 b3
y1-H2O+ 129.10
a2+ 131.08
y1+ 147.11
b2+ 159.08
b3-H2O+ 212.10
y2-H2O+ 226.16
b3+ 230.11
y2+ 244.17
y7++ 414.27
y4+ 471.33
[M]++ 493.30
b5-H2O+ 497.27
y5+ 542.37
y6-H2O+ 738.49
y6+ 756.49
y7+ 827.53

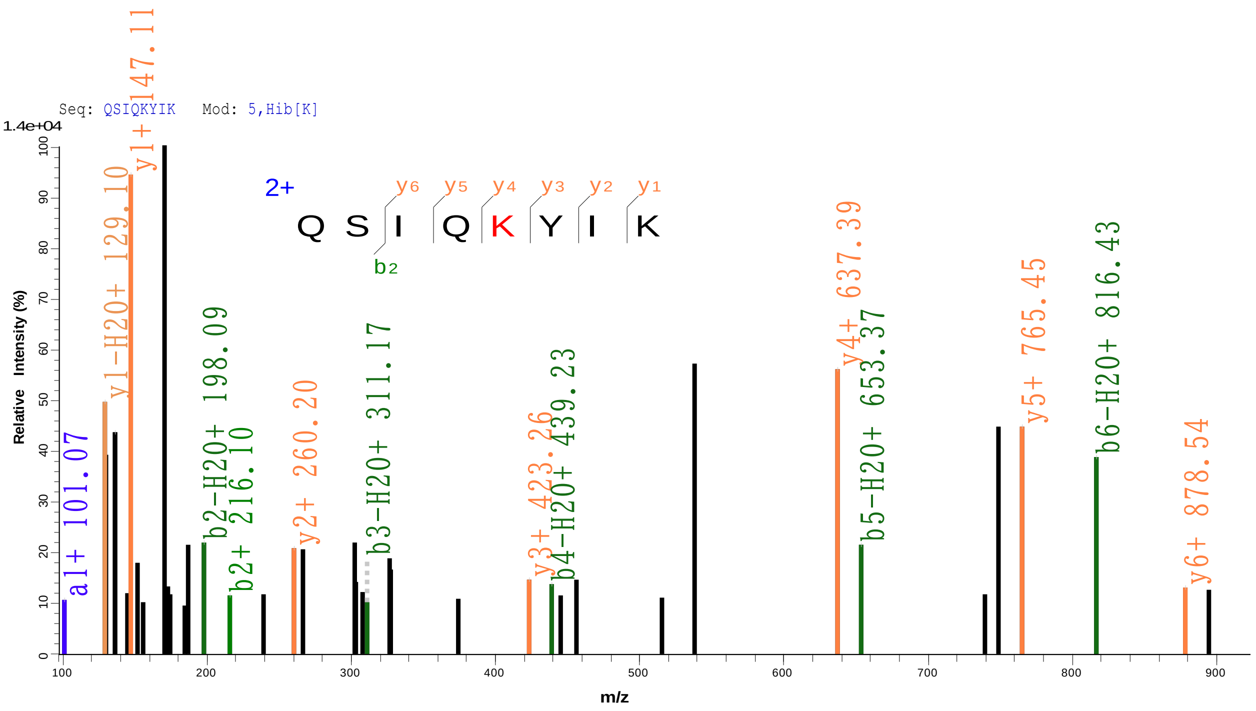
Seq: QSIQKYIK Mod: 5,Hib[K]
1.4e+04
Relative Intensity (%)
m/z
2+
Q S I Q K Y I K
y6 y5 y4 y3 y2 y1
b2
a1+ 101.07
y1-H2O+ 129.10
y1+ 147.11
b2-H2O+ 198.09
b2+ 216.10
y2+ 260.20
b3-H2O+ 311.17
y3+ 423.26
b4-H2O+ 439.23
y4+ 637.39
b5-H2O+ 653.37
y5+ 765.45
b6-H2O+ 816.43
y6+ 878.54

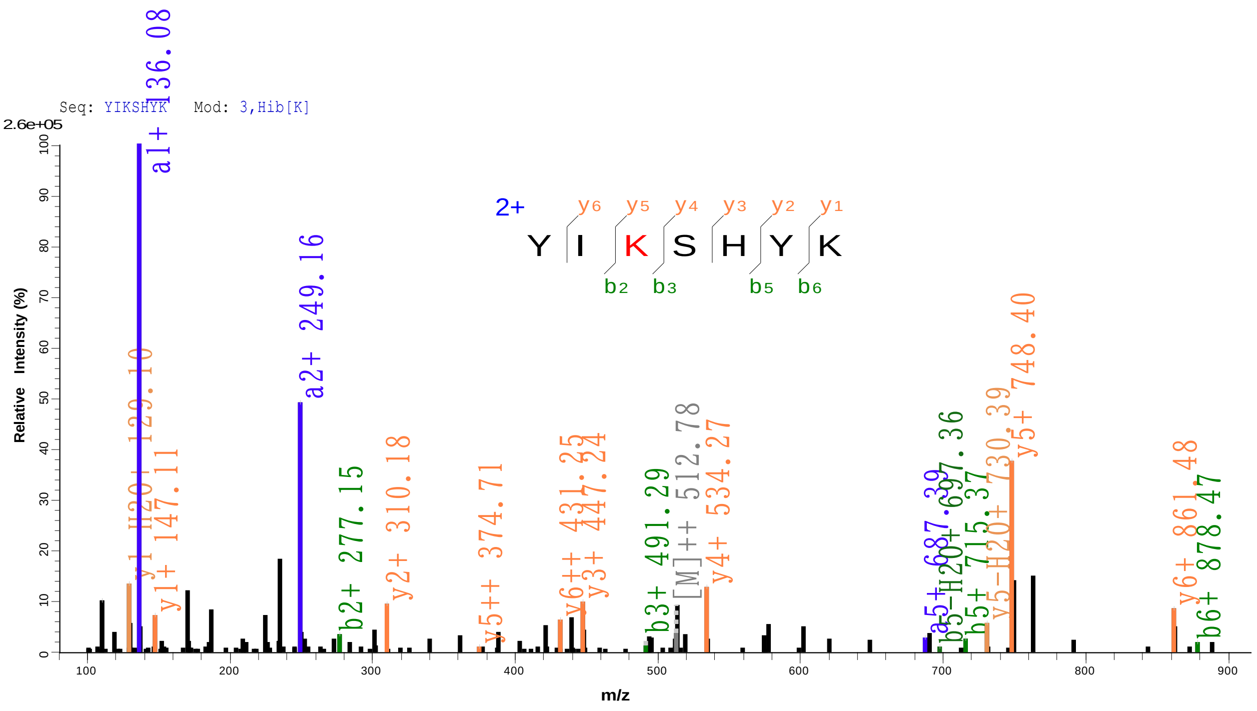
Seq: YIKSHYK Mod: 3,Hib[K]
2.6e+05
Relative Intensity (%)
m/z
2+
Y I K S H Y K
y6 y5 y4 y3 y2 y1
b2 b3 b5 b6
a1+ 136.08
y1-H2O+ 129.10
y1+ 147.11
a2+ 249.16
b2+ 277.15
y2+ 310.18
y5++ 374.71
y6++ 431.25
y3+ 447.24
b3+ 491.29
[M]++ 512.78
y4+ 534.27
a5+ 687.39
b5-H2O+ 697.36
b5+ 715.37
y5-H2O+ 730.39
y5+ 748.40
y6+ 861.48
b6+ 878.47

Seq: LVTTGVLKQTK   Mod: 8,Hib[K]

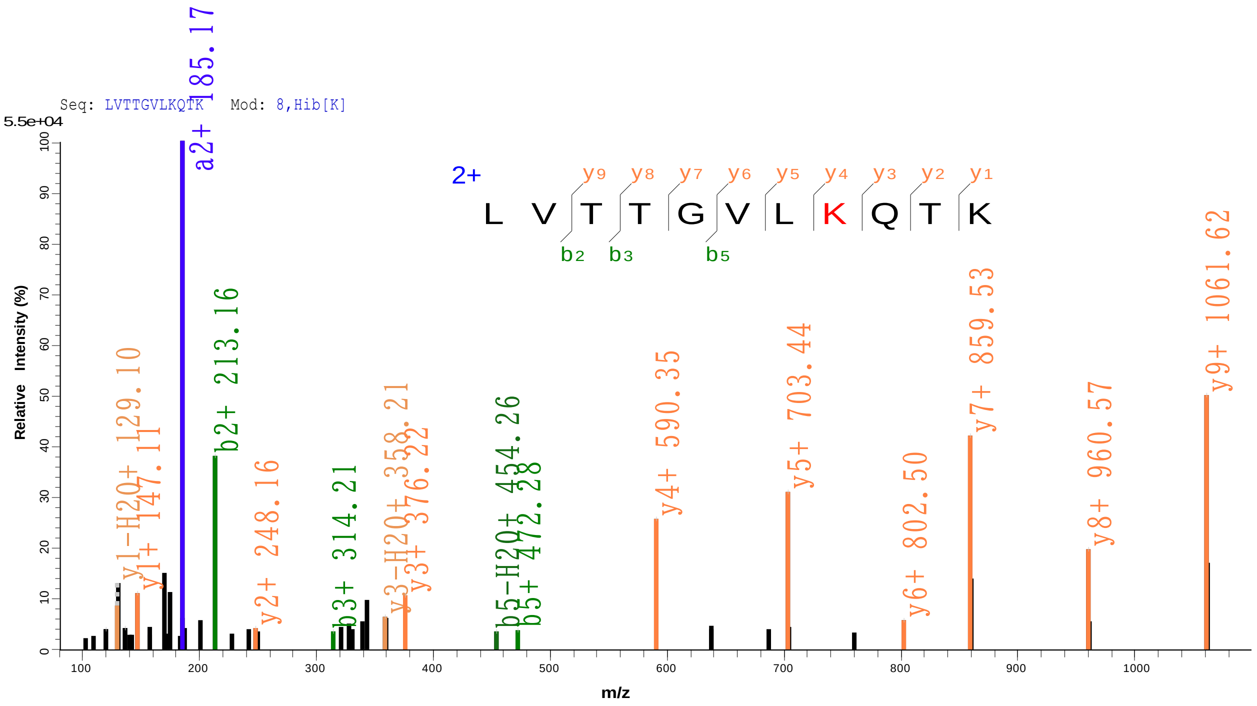

Seq: QTKGVGASGSFR   Mod: 3,Hib[K]

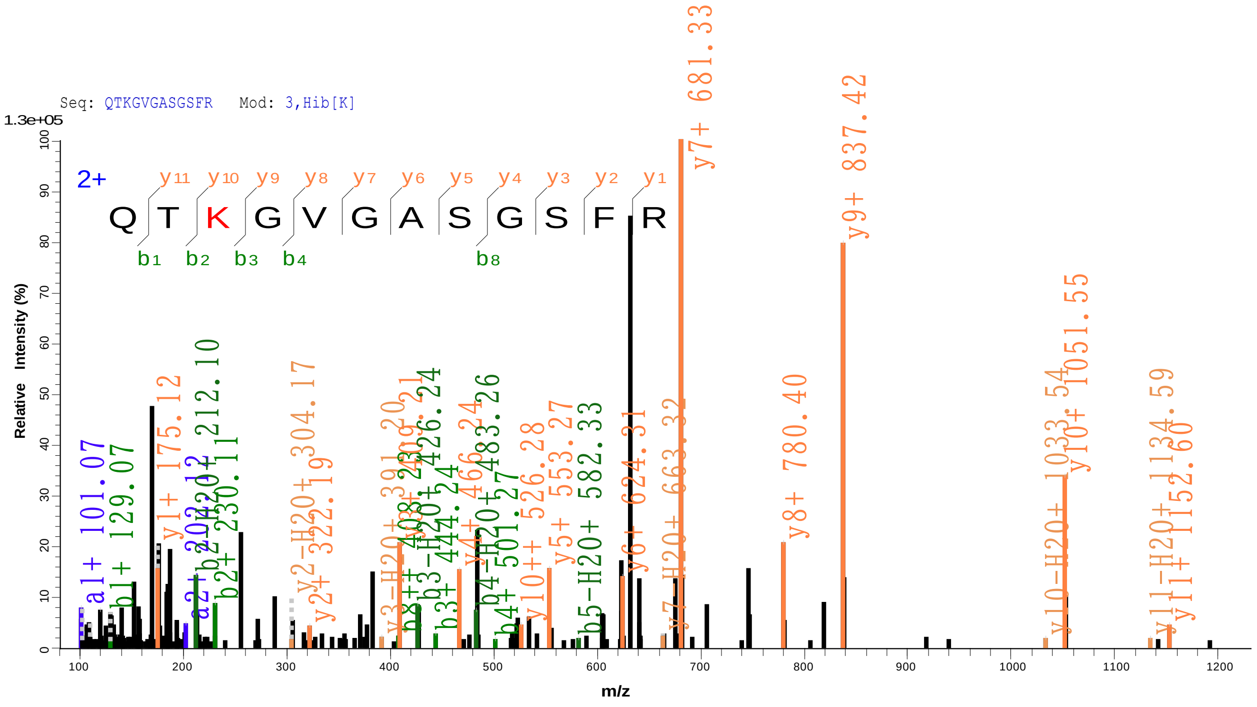

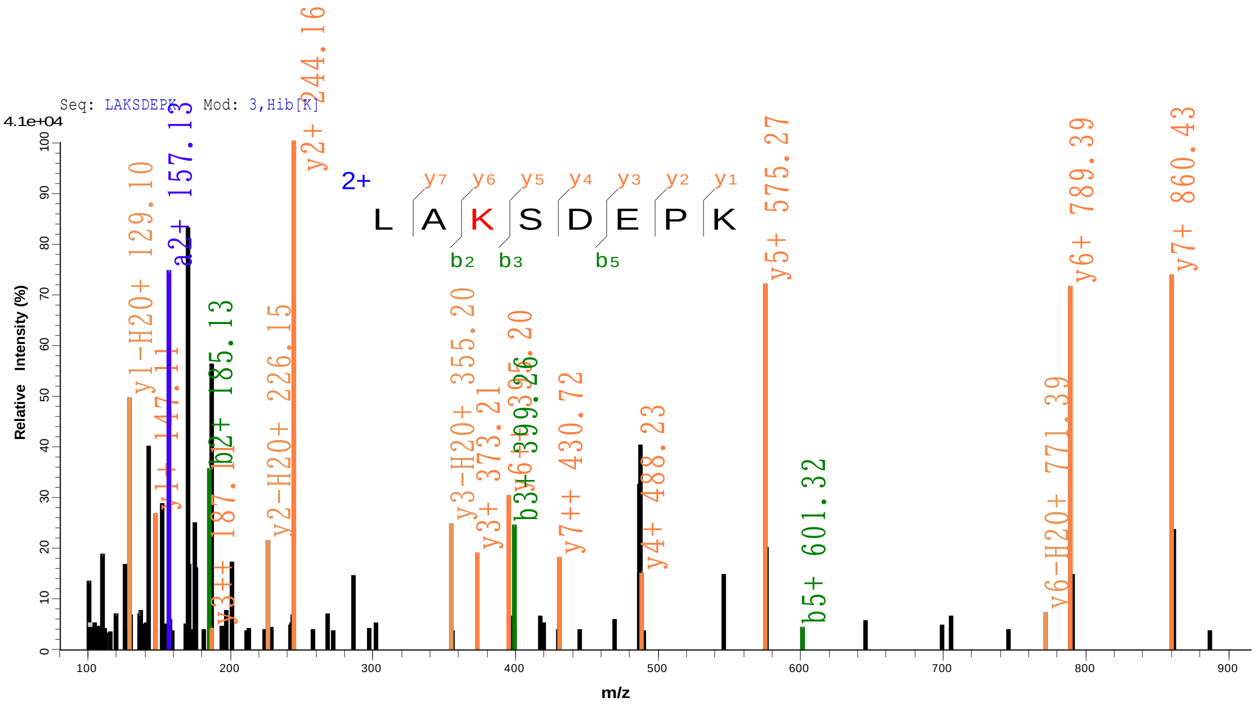
Seq: LAKSDEPK Mod: 3,Hib[K]
4.1e+04
Relative Intensity (%)
m/z
2+
L A K S D E P K
y7 y6 y5 y4 y3 y2 y1
b2 b3 b5
y2+ 244.16
a2+ 157.13
y1-H2O+ 129.10
y5+ 575.27
y6+ 789.39
y7+ 860.43
y2-H2O+ 226.15
y3-H2O+ 355.20
y7++ 430.72
y4+ 488.23
b5+ 601.32
y6-H2O+ 771.39

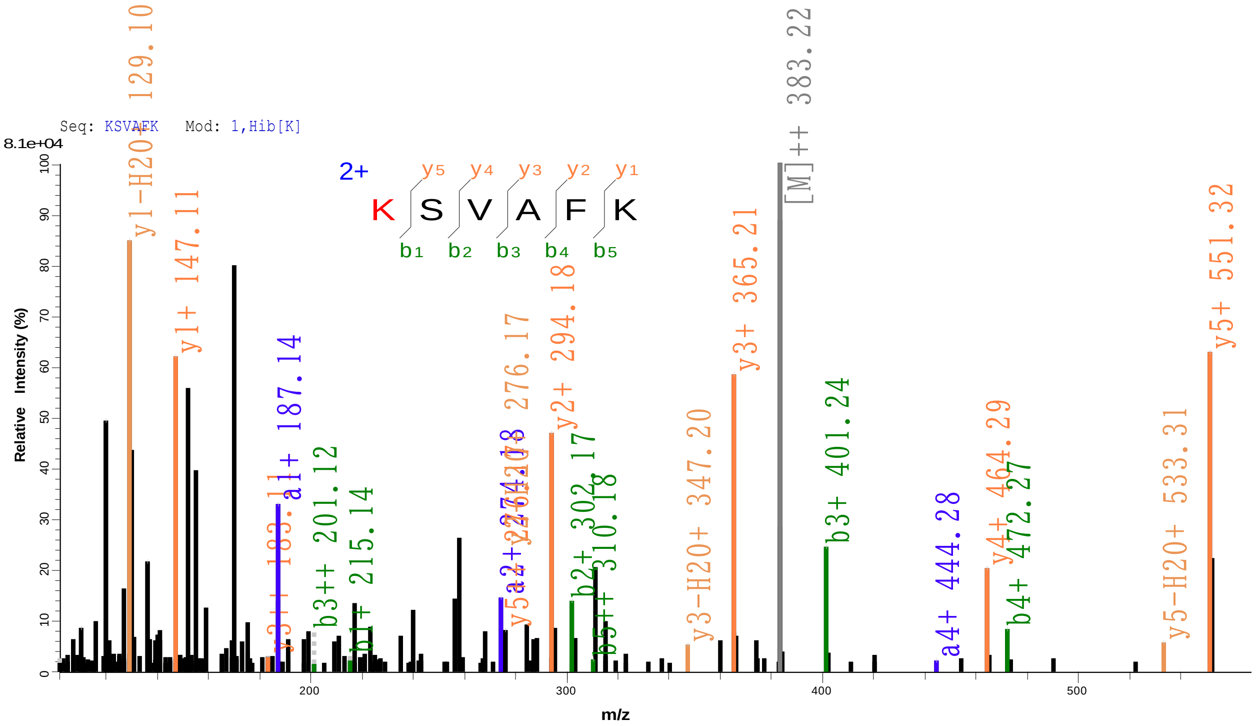
Seq: KSVAFK Mod: 1,Hib[K]
8.1e+04
Relative Intensity (%)
m/z
2+
K S V A F K
y5 y4 y3 y2 y1
b1 b2 b3 b4 b5
y1-H2O+ 129.10
y1+ 147.11
a1+ 187.14
b3++ 201.12
y3+ 365.21
[M]++ 383.22
y2+ 294.18
y3-H2O+ 347.20
b3+ 401.24
a4+ 444.28
y4+ 464.29
b4+ 472.27
y5-H2O+ 533.31
y5+ 551.32

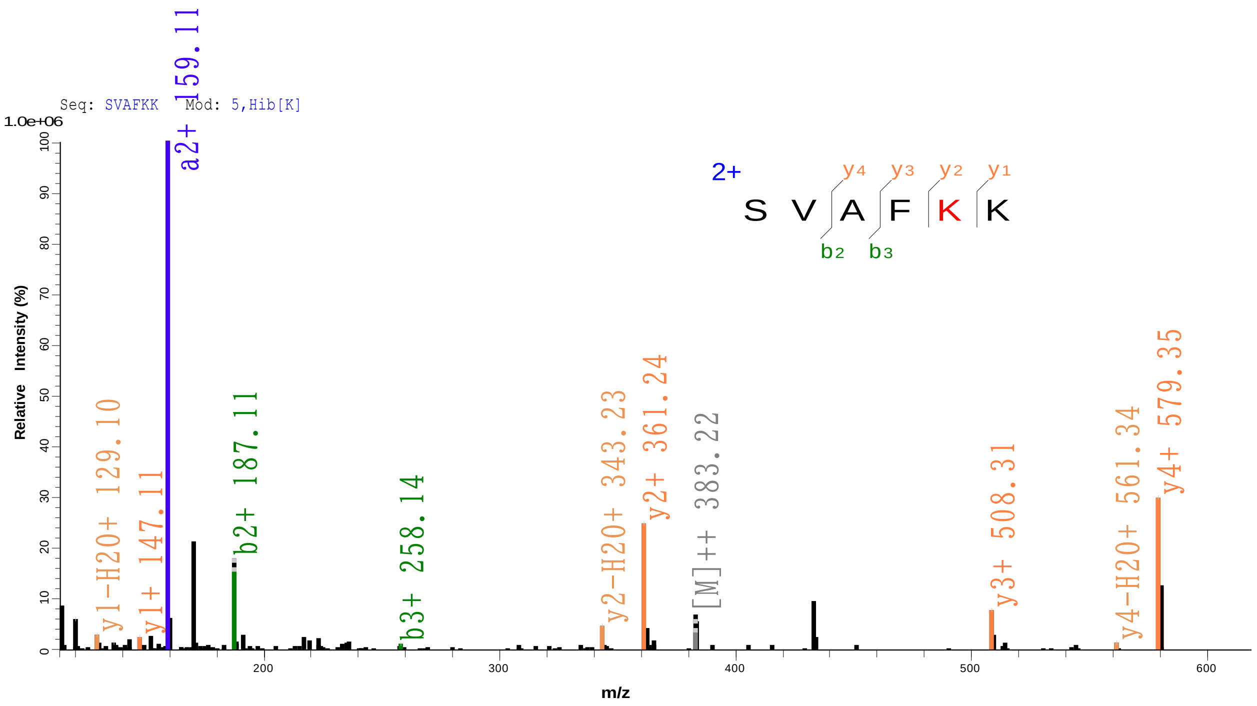
Seq: SVAFKK Mod: 5,Hib[K]
1.0e+06
Relative Intensity (%)
m/z
2+
S V A F K K
y4 y3 y2 y1
b2 b3
a2+ 159.11
y1-H2O+ 129.10
y1+ 147.11
b2+ 187.11
b3+ 258.14
y2-H2O+ 343.23
y2+ 361.24
[M]++ 383.22
y3+ 508.31
y4-H2O+ 561.34
y4+ 579.35

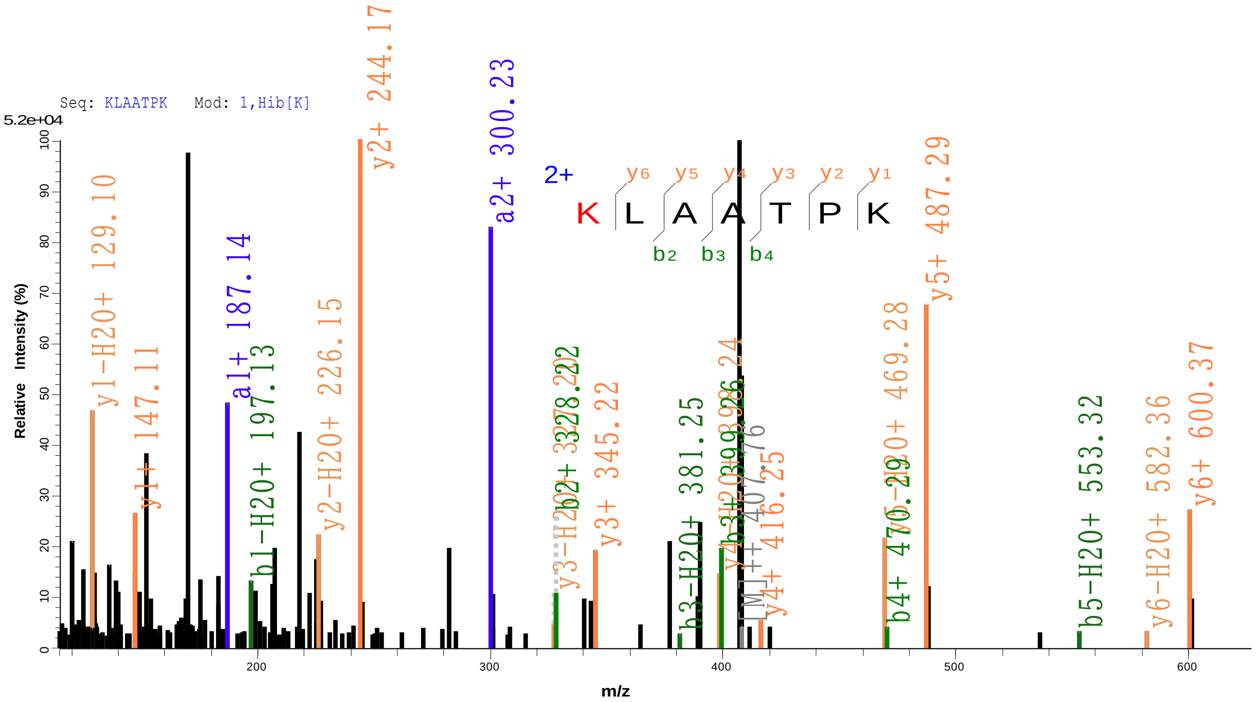
Seq: KLAATPK Mod: 1,Hib[K]
5.2e+04
Relative Intensity (%)
m/z
2+
K L A A T P K
y6 y5 y4 y3 y2 y1
b2 b3 b4
y2+ 244.17
a2+ 300.23
y1-H2O+ 129.10
y1+ 147.11
a1+ 187.14
b1-H2O+ 197.13
y2-H2O+ 226.15
y3+ 345.22
b3-H2O+ 381.25
y4+ 416.25
y5+ 487.29
b5-H2O+ 553.32
y6-H2O+ 582.36
y6+ 600.37

Seq: VTKAGGSAALSPSK   Mod: 3,Hib[K]

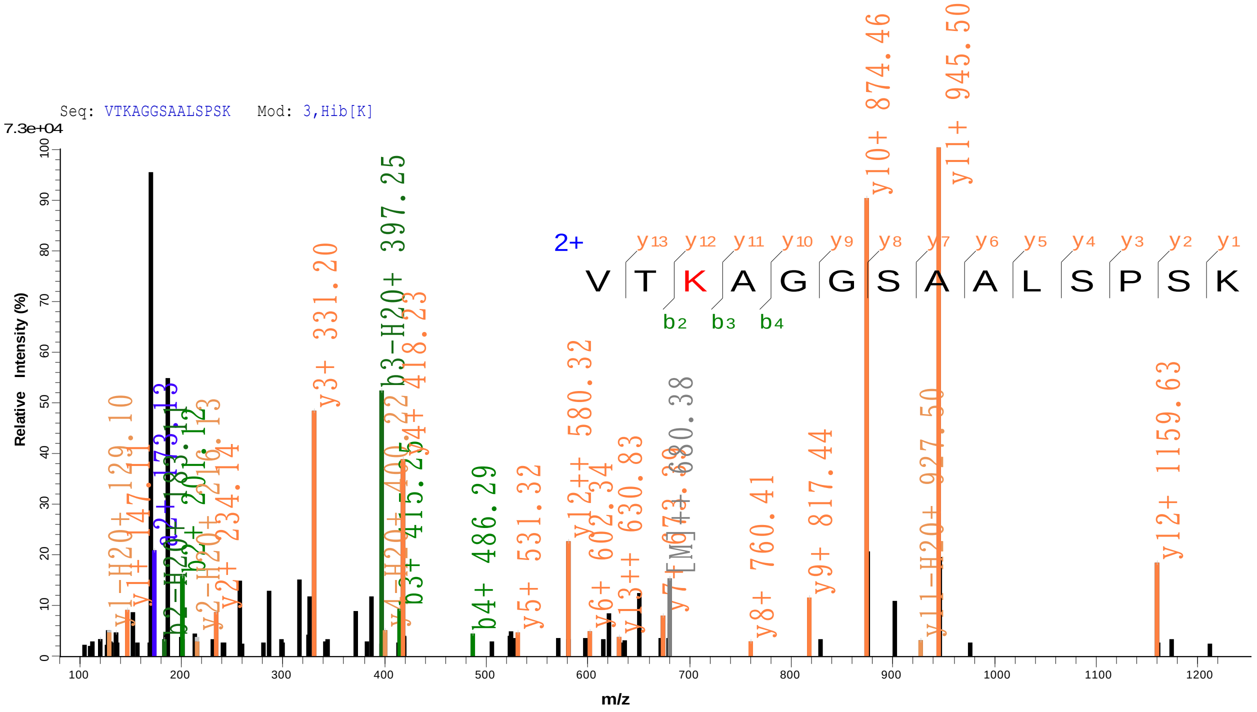

Seq: IYTEAKK   Mod: 6,Hib[K]

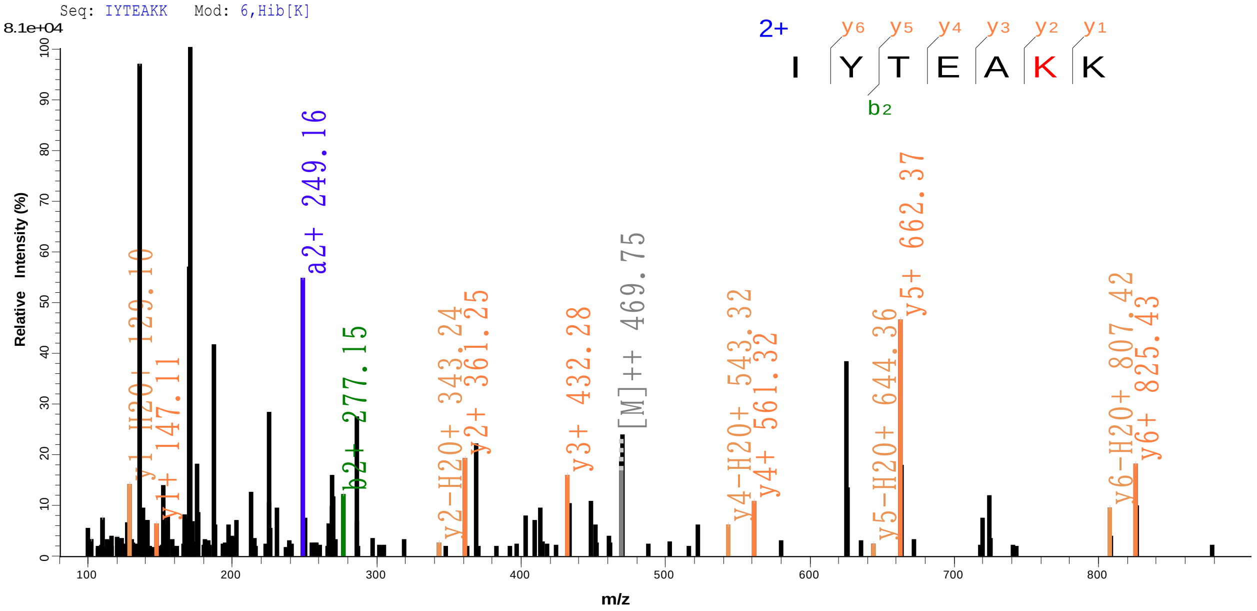

H2A

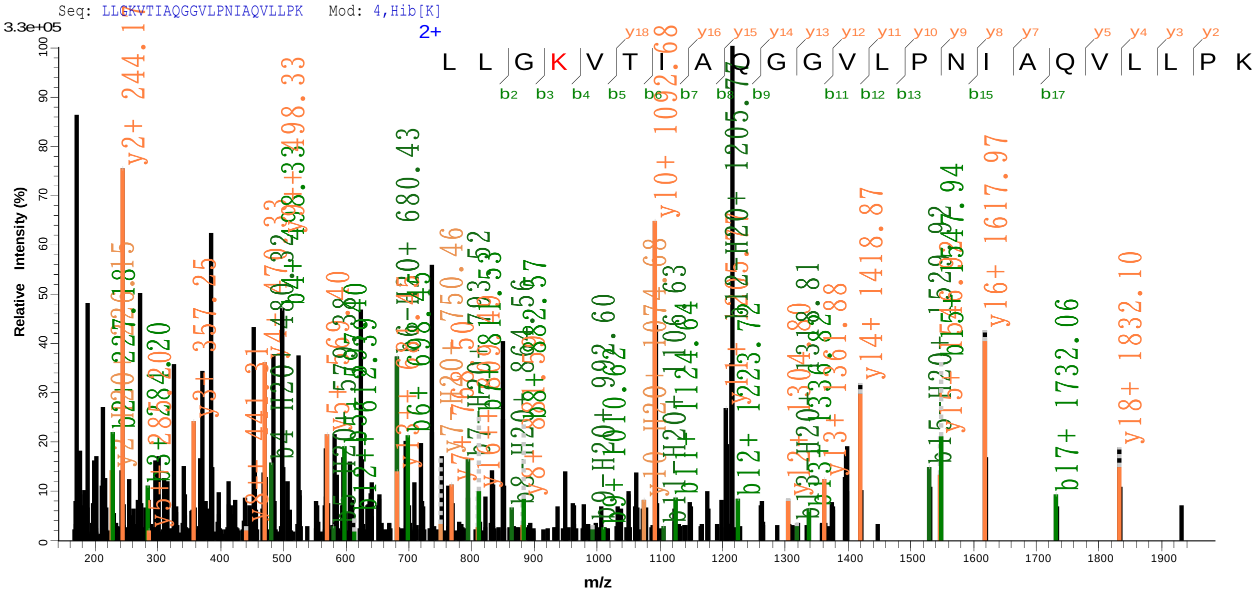
Seq: LLGKVTIAQGGVLPNIAQVLLPK Mod: 4,Hib[K]
3.3e+05
2+
L L G K V T I A Q G G V L P N I A Q V L L P K
Relative Intensity (%)
m/z
y2+ 244.17
y10+ 1092.68
y14+ 1418.87
y16+ 1617.97
b17+ 1732.06
y18+ 1832.10

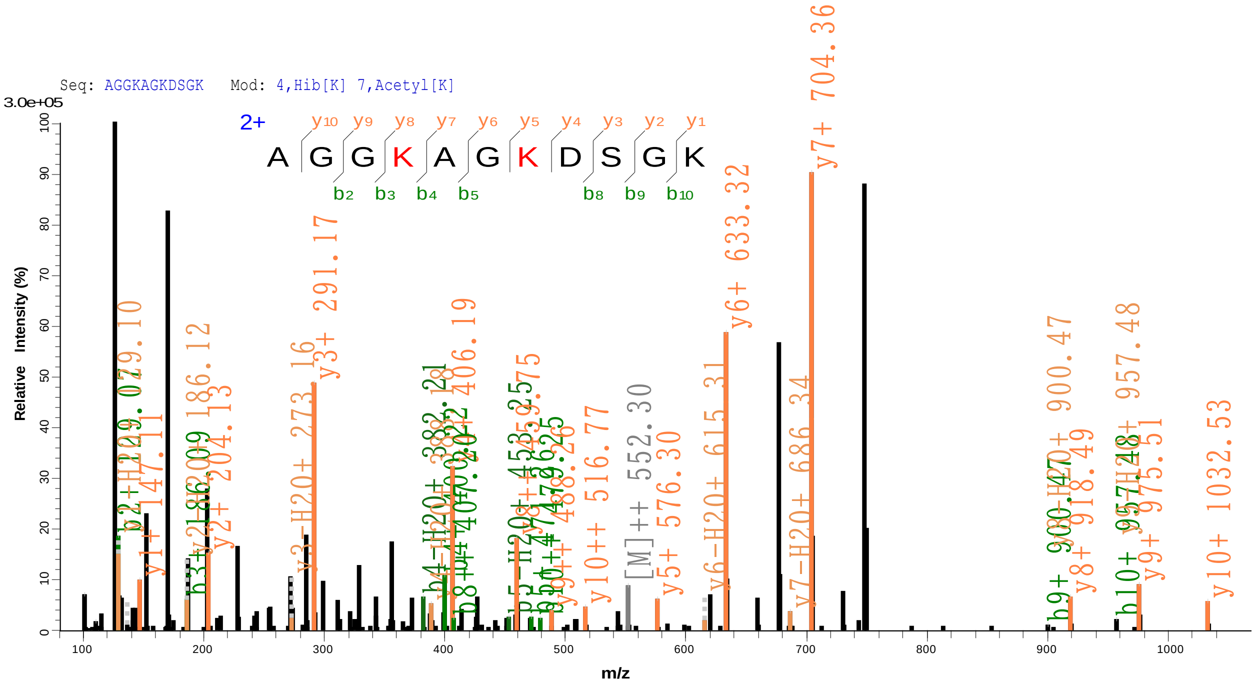
Seq: AGGKAGKDSGK Mod: 4,Hib[K] 7,Acetyl[K]
3.0e+05
2+
A G G K A G K D S G K
Relative Intensity (%)
m/z
y3+ 291.17
y6+ 633.32
y7+ 704.36
[M]++ 552.30
y10+ 1032.53

Seq: AGKDSGKAK Mod: 3,Acetyl[K] 7,Hib[K]

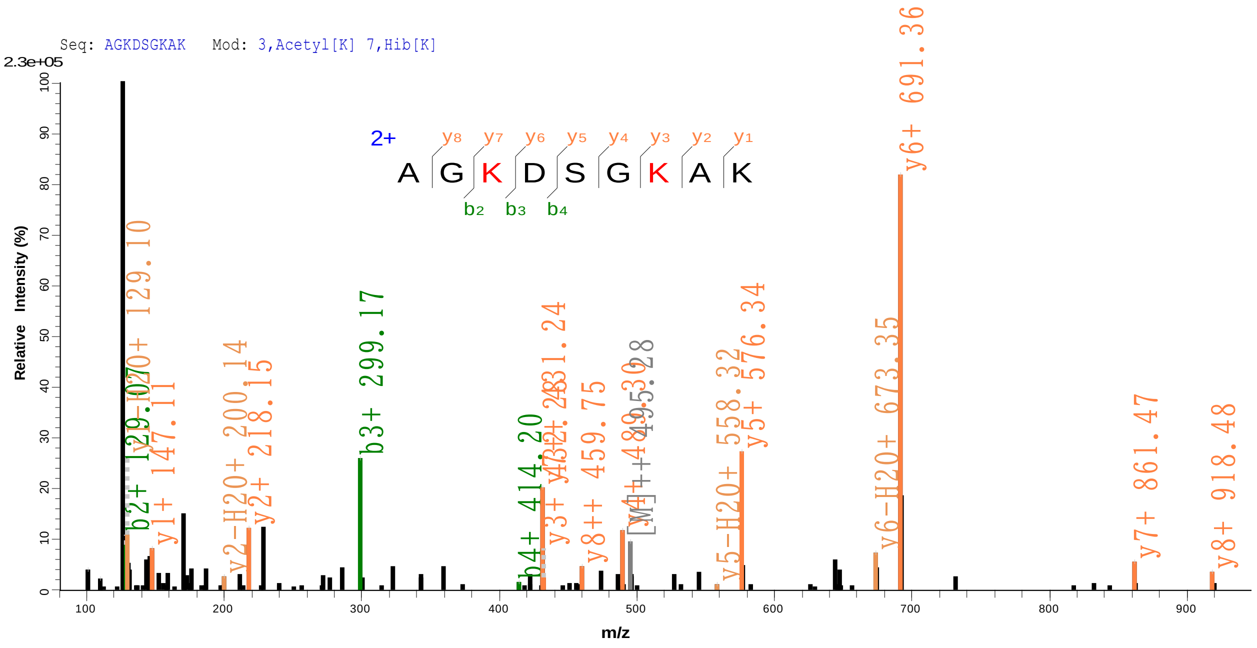

Seq: DSGKAKTK Mod: 4,Acetyl[K] 6,Hib[K]

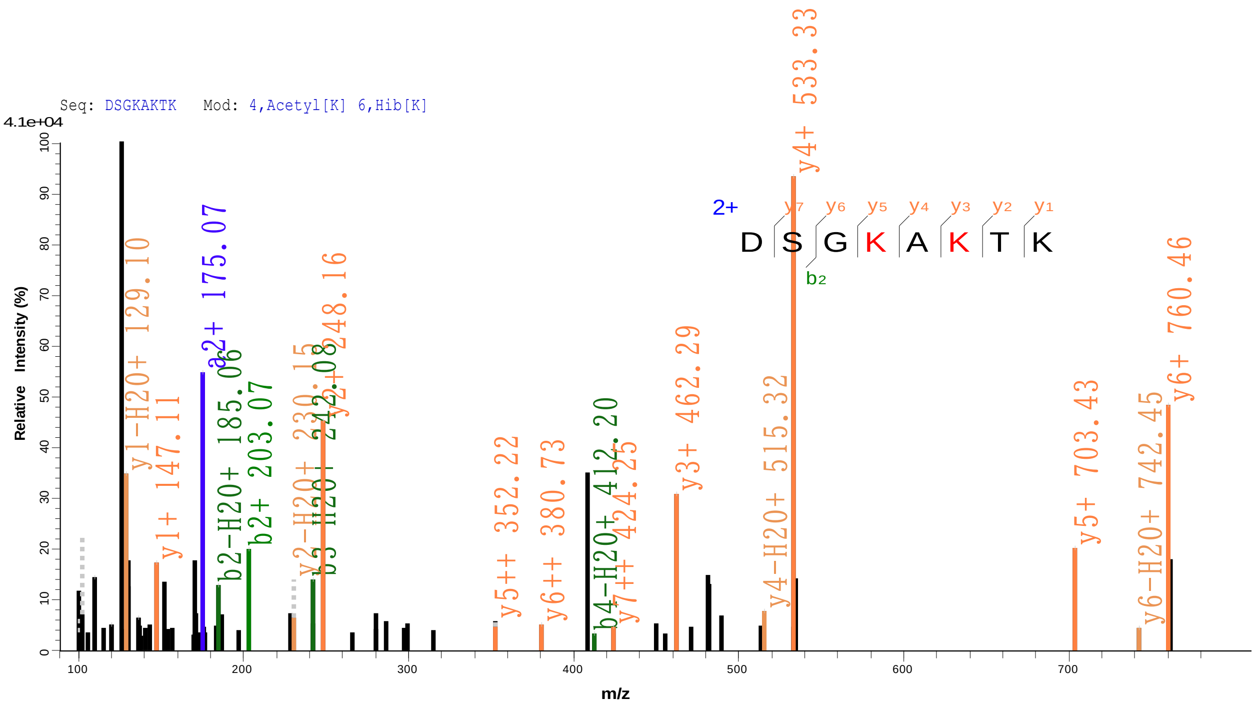

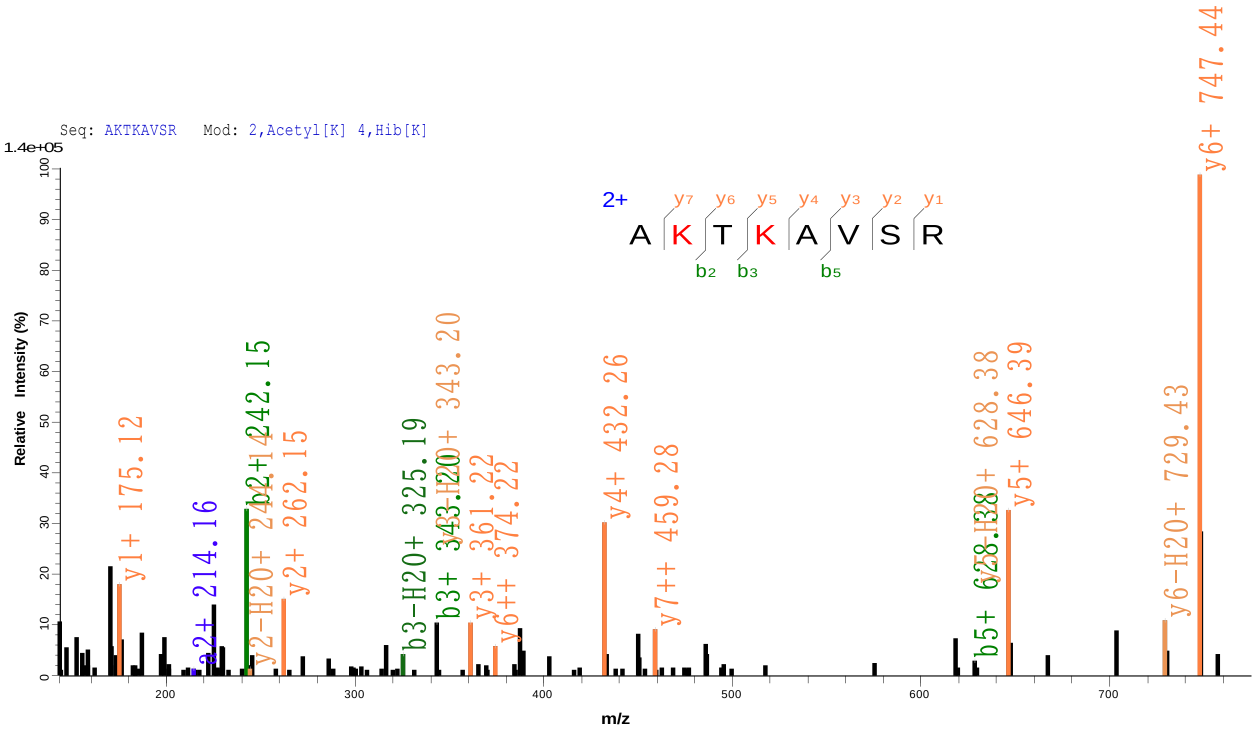
Seq: AKTKAVSR Mod: 2,Acetyl[K] 4,Hib[K]
1.4e+05
Relative Intensity (%)
m/z
2+
A K T K A V S R
y7 y6 y5 y4 y3 y2 y1
b2 b3 b5
y1+ 175.12
a2+ 214.16
b2+ 242.15
y2-H20+ 244.14
y2+ 262.15
b3-H20+ 325.19
b3+ 343.20
y3-H20+ 343.20
y3+ 361.22
y6++ 374.22
y4+ 432.26
y7++ 459.28
b5+ 628.38
y5-H20+ 628.38
y5+ 646.39
y6-H20+ 729.43
y6+ 747.44

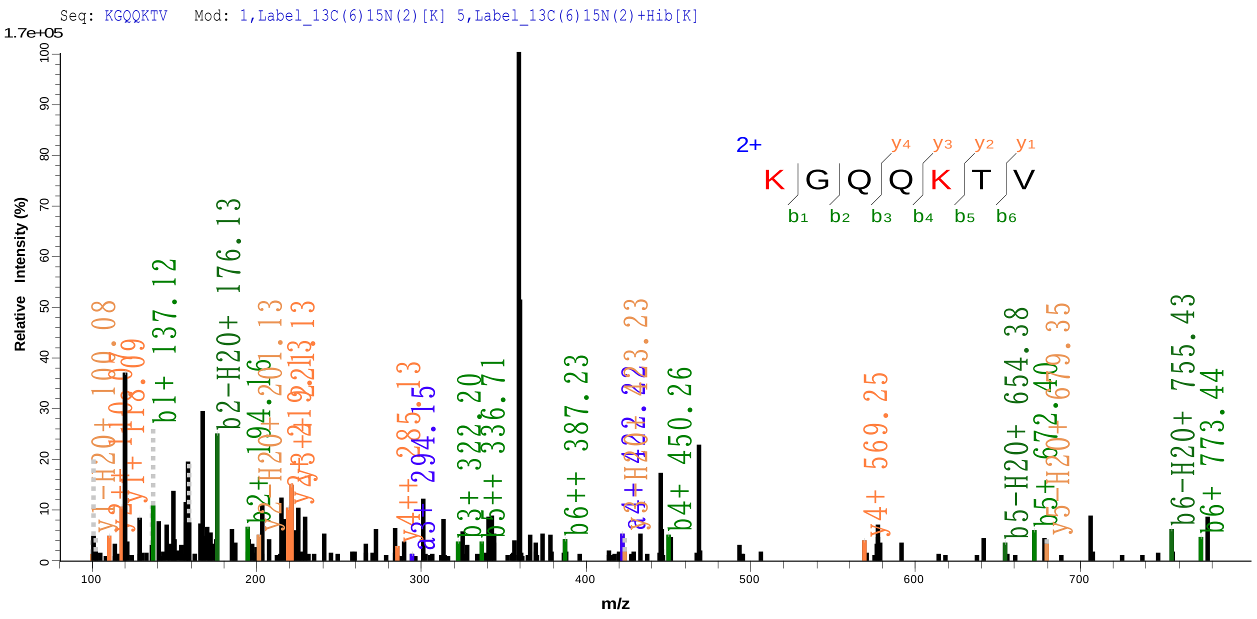
Seq: KGQQKTV Mod: 1,Label_13C(6)15N(2)[K] 5,Label_13C(6)15N(2)+Hib[K]
1.7e+05
Relative Intensity (%)
m/z
2+
K G Q Q K T V
y4 y3 y2 y1
b1 b2 b3 b4 b5 b6
y1-H20+ 100.08
y1+ 118.09
b1+ 137.12
b2-H20+ 176.13
b2+ 194.16
y2-H20+ 201.13
y2+ 219.13
y4++ 285.13
a3+ 294.15
b3+ 322.20
b5++ 336.71
b6++ 387.23
a4+ 422.23
b4+ 450.26
y4+ 569.25
b5-H20+ 654.38
b5+ 672.40
y5-H20+ 679.35
b6-H20+ 755.43
b6+ 773.44

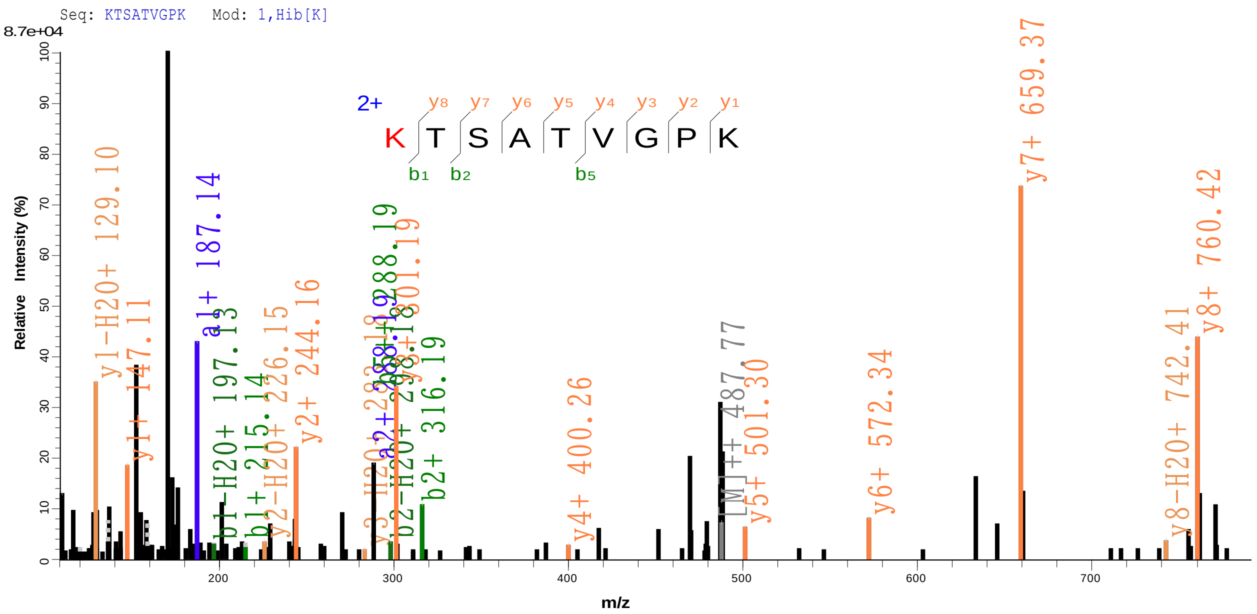
Seq: KTSATVGPK Mod: 1,Hib[K]
8.7e+04
Relative Intensity (%)
m/z
2+
K T S A T V G P K
y8 y7 y6 y5 y4 y3 y2 y1
b1 b2 b5
y1-H2O+ 129.10
y1+ 147.11
a1+ 187.14
b1-H2O+ 197.13
b1+ 215.14
y2-H2O+ 226.15
y2+ 244.16
b2+ 316.19
y4+ 400.26
[M]++ 487.77
y5+ 501.30
y6+ 572.34
y7+ 659.37
y8-H2O+ 742.41
y8+ 760.42

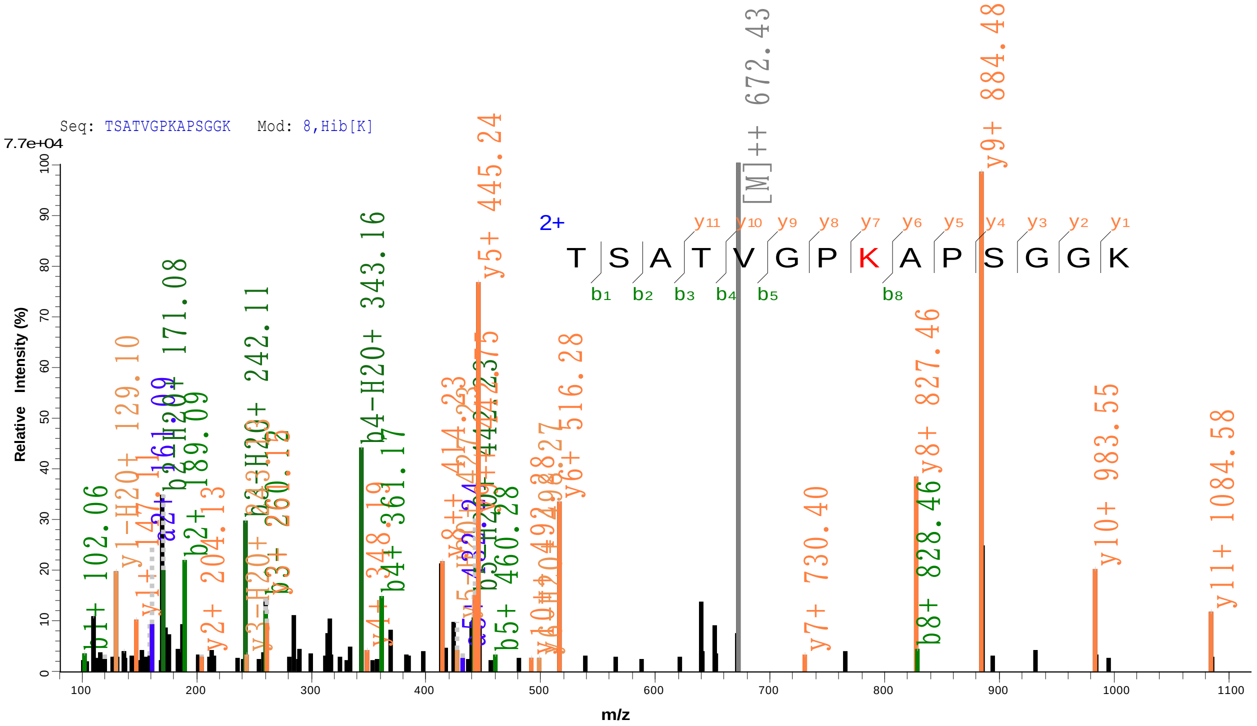
Seq: TSATVGPKAPSGGK Mod: 8,Hib[K]
7.7e+04
Relative Intensity (%)
m/z
2+
T S A T V G P K A P S G G K
y11 y10 y9 y8 y7 y6 y5 y4 y3 y2 y1
b1 b2 b3 b4 b5 b8
b1+ 102.06
y1-H2O+ 129.10
b2+ 171.08
b2+ 189.09
y2+ 204.13
b3-H2O+ 242.11
b3+ 260.12
b4-H2O+ 343.16
y4+ 348.19
b4+ 361.17
y5+ 445.24
b5+ 460.28
y6+ 516.28
[M]++ 672.43
y7+ 730.40
y8+ 827.46
b8+ 828.46
y9+ 884.48
y10+ 983.55
y11+ 1084.58

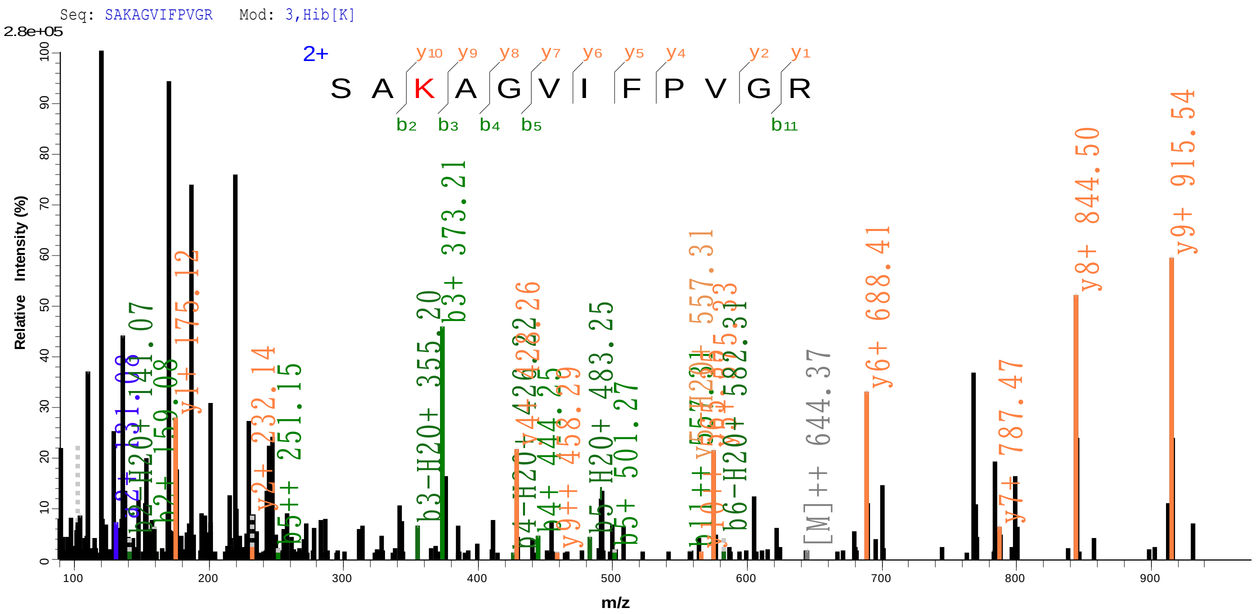
Seq: SAKAGVIFPVGR Mod: 3,Hib[K]
2.8e+05
2+
S A K A G V I F P V G R
y10 y9 y8 y7 y6 y5 y4 y2 y1
b2 b3 b4 b5 b11
b3+ 373.21
b3-H2O+ 355.20
y1+ 175.12
y2+ 232.14
b5++ 251.15
b4+ 444.25
y9++ 458.29
b5-H2O+ 483.25
b5+ 501.27
b6-H2O+ 582.31
[M]++ 644.37
y6+ 688.41
y7+ 787.47
y8+ 844.50
y9+ 915.54
Relative Intensity (%)
m/z

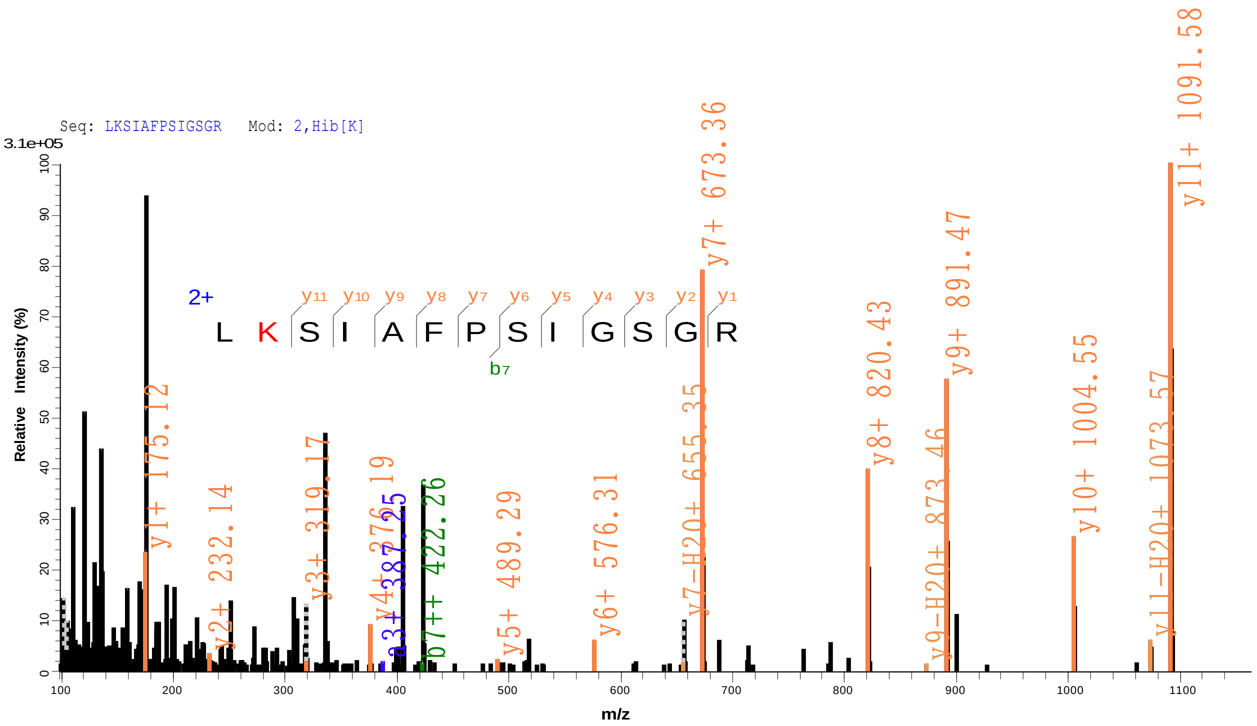
Seq: LKSIAFPSIGSGR Mod: 2,Hib[K]
3.1e+05
2+
L K S I A F P S I G S G R
y11 y10 y9 y8 y7 y6 y5 y4 y3 y2 y1
b7
y1+ 175.12
y2+ 232.14
y3+ 319.17
y4+ 376.19
a3+ 387.25
b7++ 422.26
y5+ 489.29
y6+ 576.31
y7-H2O+ 655.35
y7+ 673.36
y8+ 820.43
y9-H2O+ 873.46
y9+ 891.47
y10+ 1004.55
y11-H2O+ 1073.57
y11+ 1091.58
Relative Intensity (%)
m/z

H2B

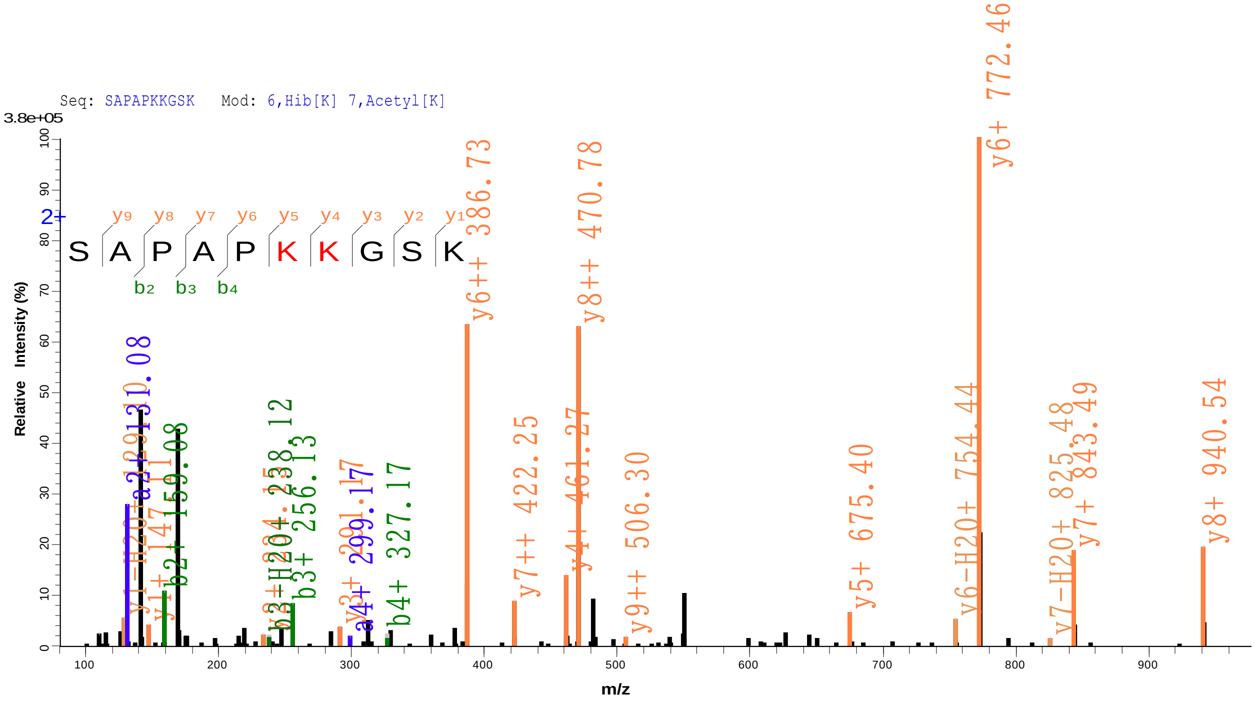

Seq: SAPAPKKGSK Mod: 6,Hib[K] 7,Acetyl[K]
3.8e+05
2+
S A P A P K K G S K
y9 y8 y7 y6 y5 y4 y3 y2 y1
b2 b3 b4
Relative Intensity (%)
m/z
y6+ 772.46
y6++ 386.73
y8++ 470.78
y4+ 461.27
y7++ 422.25
y9++ 506.30
y5+ 675.40
y6-H2O+ 754.44
y7-H2O+ 825.48
y7+ 843.49
y8+ 940.54
b2+ 159.08
b3+ 256.13
b4+ 327.17
a4+ 299.17

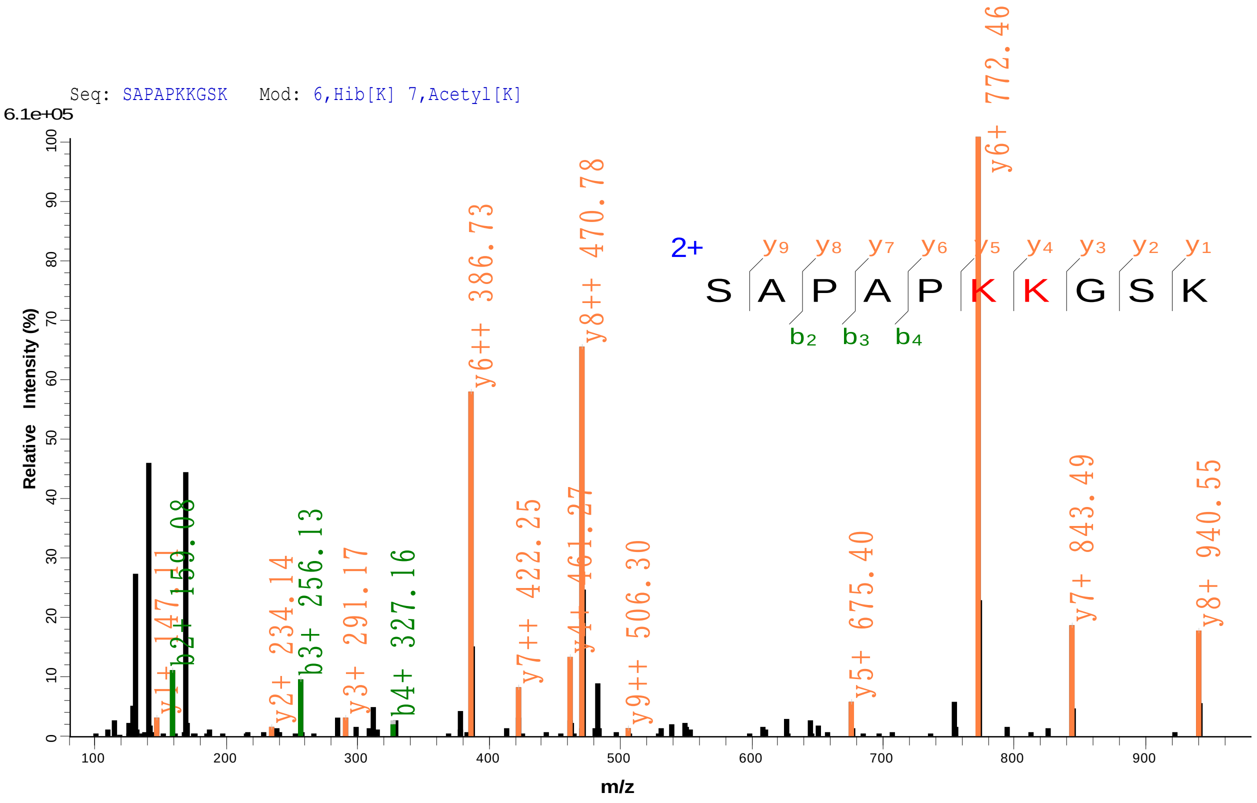

Seq: SAPAPKKGSK Mod: 6,Hib[K] 7,Acetyl[K]
6.1e+05
2+
S A P A P K K G S K
y9 y8 y7 y6 y5 y4 y3 y2 y1
b2 b3 b4
Relative Intensity (%)
m/z
y6+ 772.46
y6++ 386.73
y8++ 470.78
y4+ 461.27
y7++ 422.25
y9++ 506.30
y5+ 675.40
y7+ 843.49
y8+ 940.55
y1+ 147.11
b2+ 159.08
y2+ 234.14
b3+ 256.13
y3+ 291.17
b4+ 327.16

Seq: GSKKAVTK   Mod: 3,Acetyl[K] 4,Hib[K]

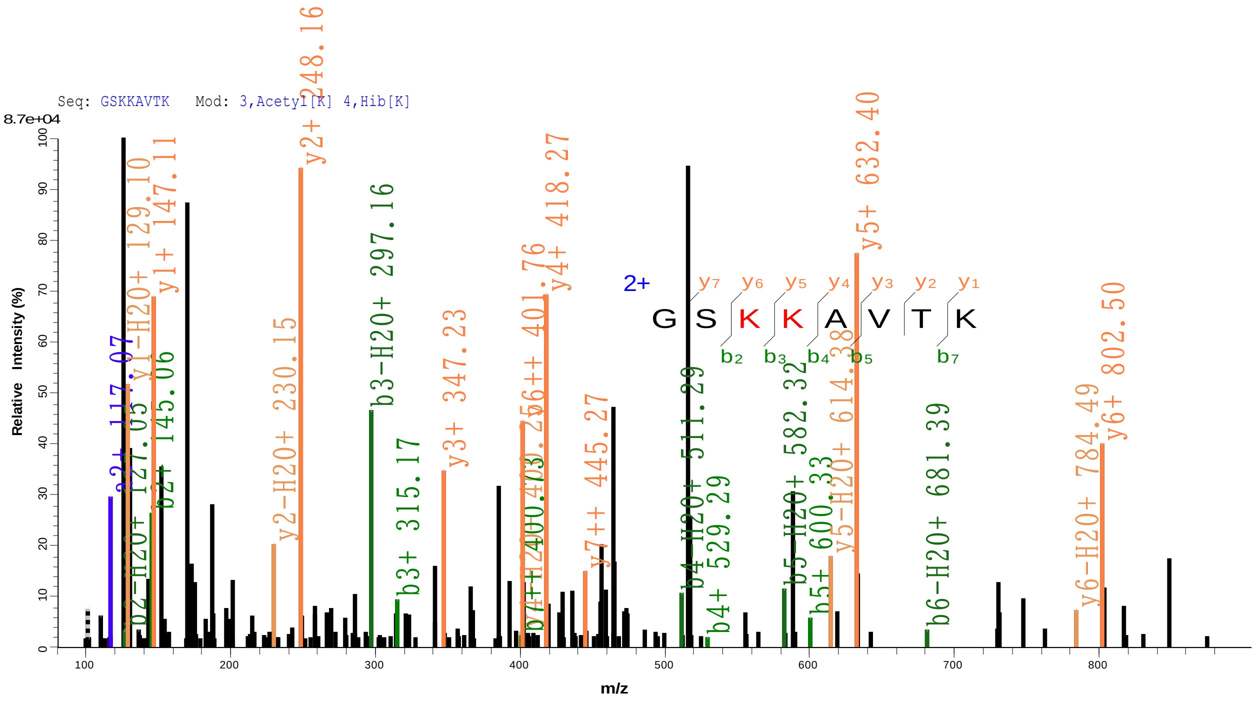

D. Kbz

H1

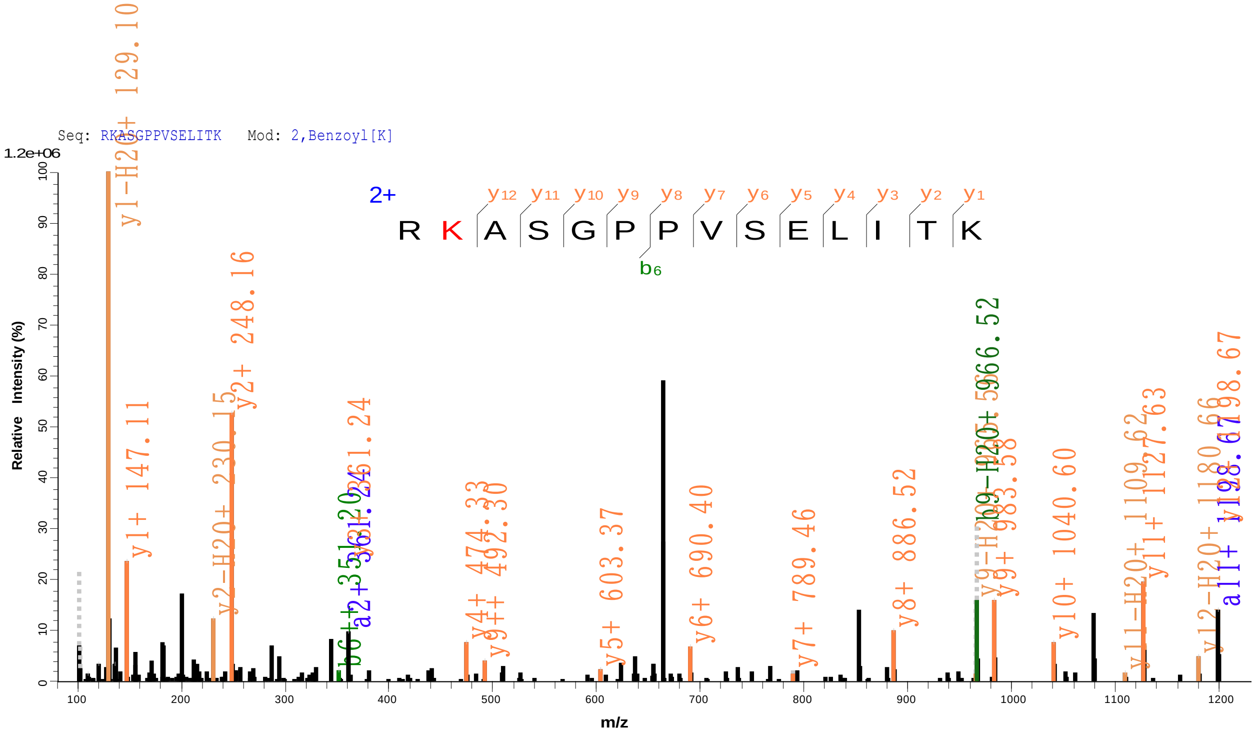

Seq: RKASGPPVSELITK Mod: 2,Benzoyl[K]
1.2e+06
Relative Intensity (%)
m/z
2+
R K A S G P P V S E L I T K
y12 y11 y10 y9 y8 y7 y6 y5 y4 y3 y2 y1
b6
y1-H2O+ 129.10
y1+ 147.11
y2+ 248.16
y5+ 603.37
y6+ 690.40
y7+ 789.46
y8+ 886.52
y10+ 1040.60

H2A

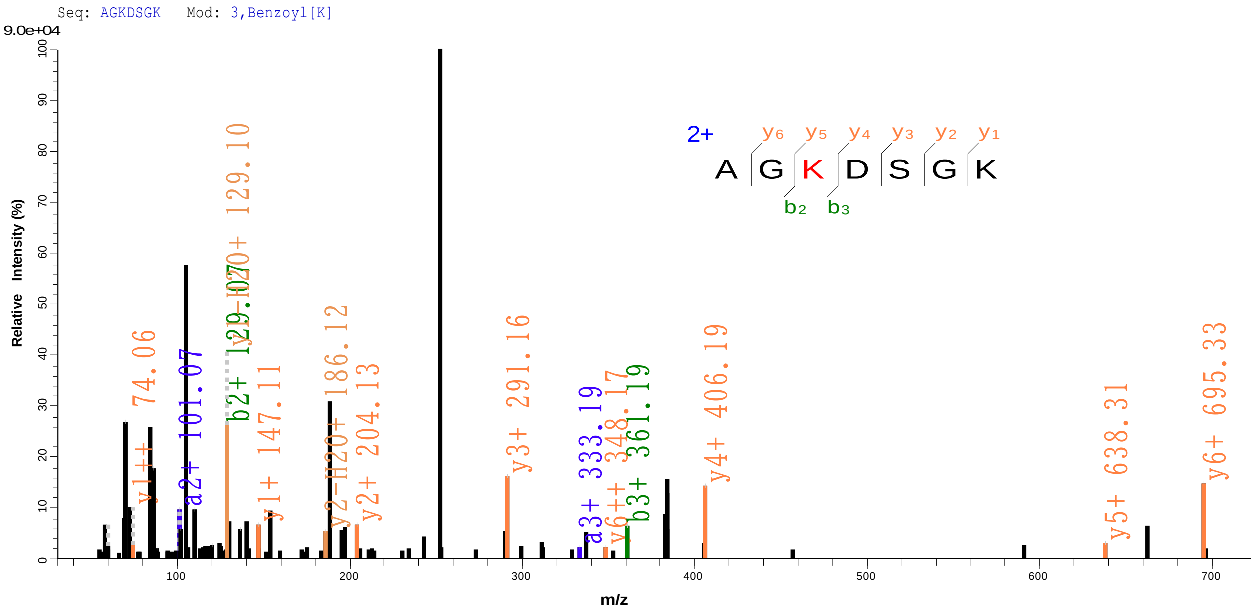

Seq: AGKDSGK Mod: 3,Benzoyl[K]
9.0e+04
Relative Intensity (%)
m/z
2+
A G K D S G K
y6 y5 y4 y3 y2 y1
b2 b3
a2+ 101.07
y1+ 147.11
y2+ 204.13
y3+ 291.16
a3+ 333.19
b3+ 361.19
y4+ 406.19
y5+ 638.31
y6+ 695.33

Seq: AGKDSGKAK Mod: 3,Acetyl[K] 7,Benzoyl[K]

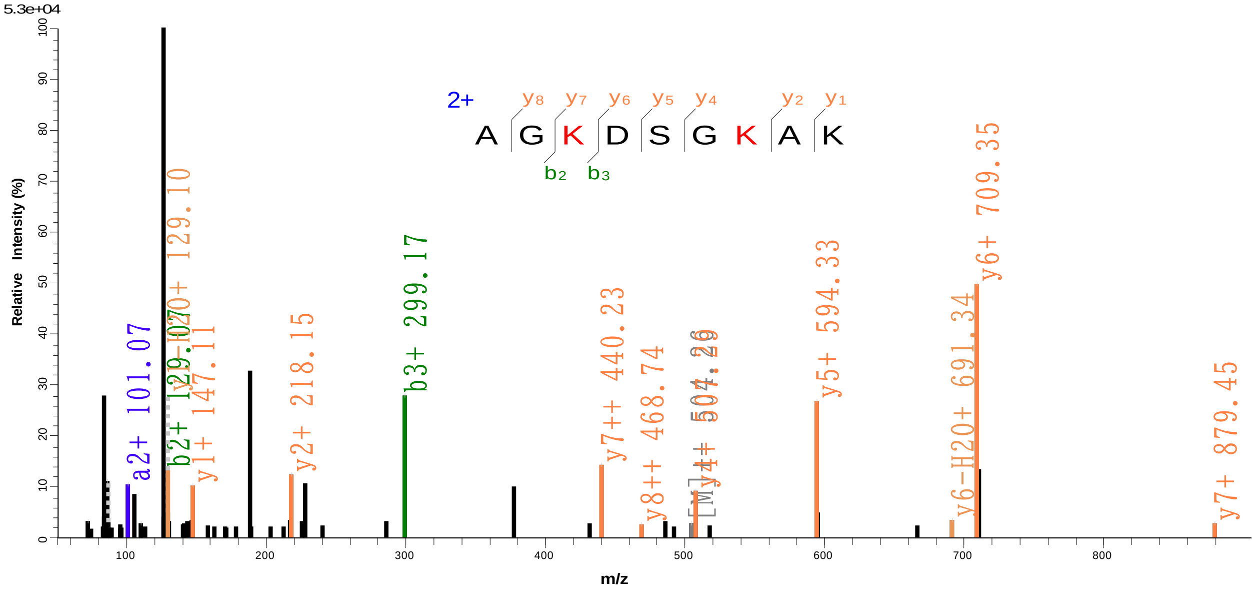

20160107_Human_Histone_BZ_IP.3853.3853.2 Seq: TKAVSR Mod: 2,Benzoyl[K]

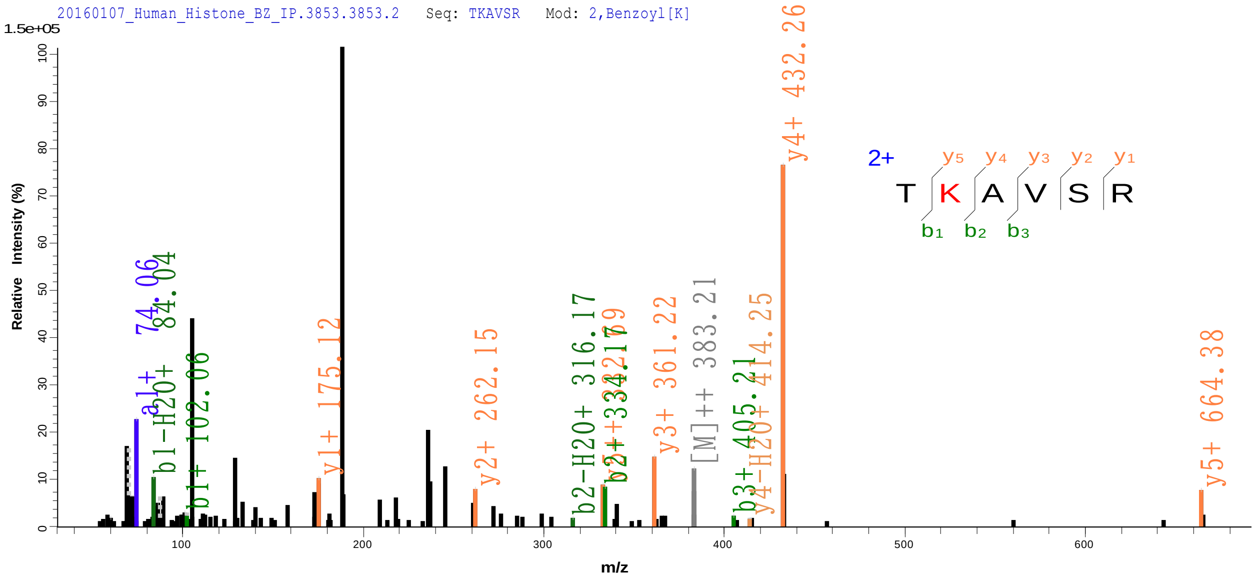

H2B

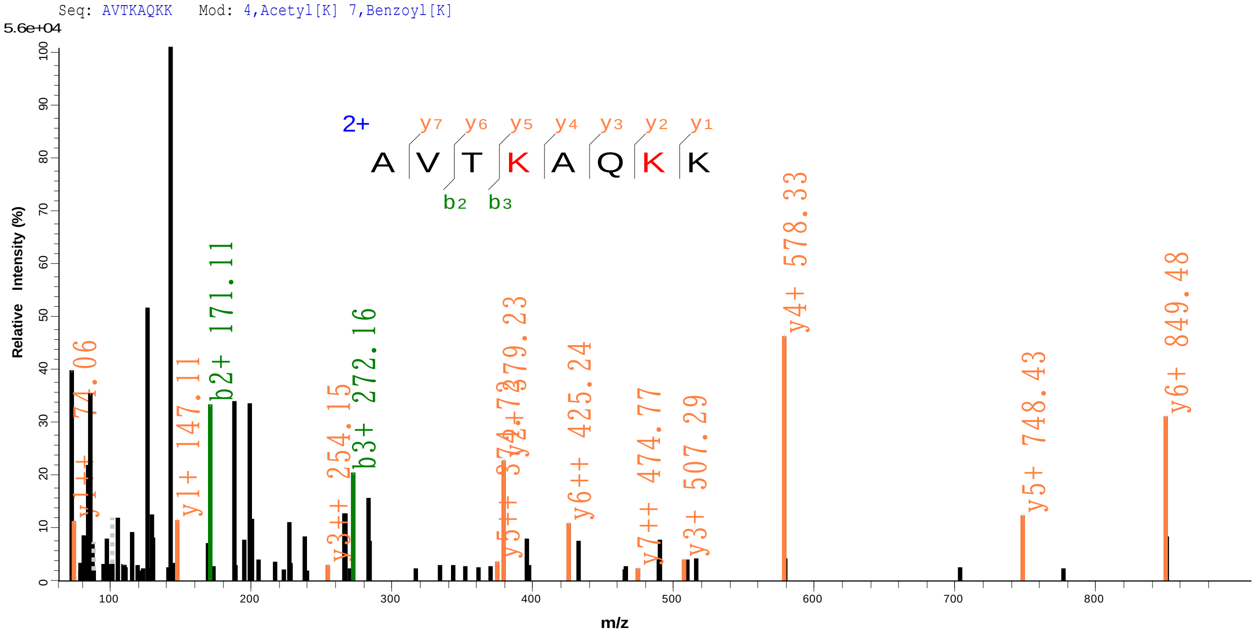

Seq: AVTKAQKK Mod: 4,Acetyl[K] 7,Benzoyl[K]
5.6e+04
Relative Intensity (%)
m/z
2+
A V T K A Q K K
y7 y6 y5 y4 y3 y2 y1
b2 b3
y1++ 74.06
y1+ 147.11
b2+ 171.11
y3++ 254.15
b3+ 272.16
y4+ 578.33
y5+ 748.43
y6+ 849.48
y6++ 425.24
y7++ 474.77
y3+ 507.29

H3

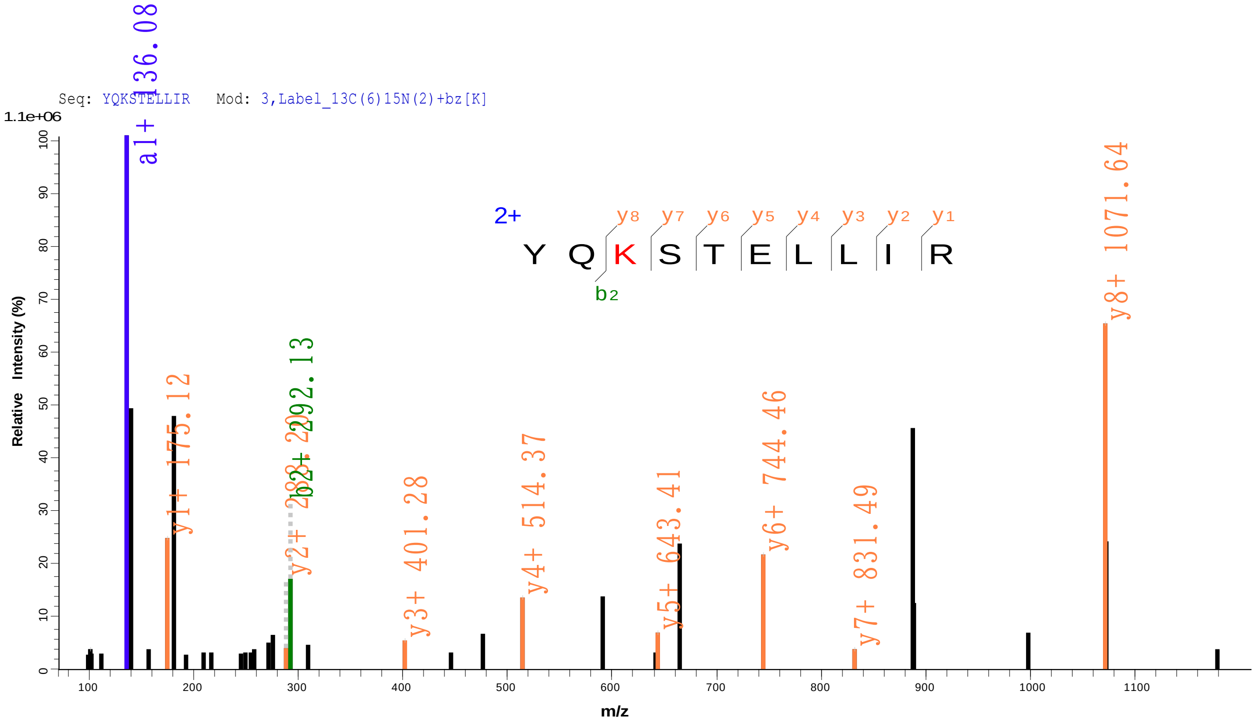

Seq: YQKSTELLIR Mod: 3,Label_13C(6)15N(2)+bz[K]
1.1e+06
Relative Intensity (%)
m/z
2+
Y Q K S T E L L I R
y8 y7 y6 y5 y4 y3 y2 y1
b2
a1+ 136.08
y1+ 175.12
y3+ 401.28
y4+ 514.37
y5+ 643.41
y6+ 744.46
y7+ 831.49
y8+ 1071.64

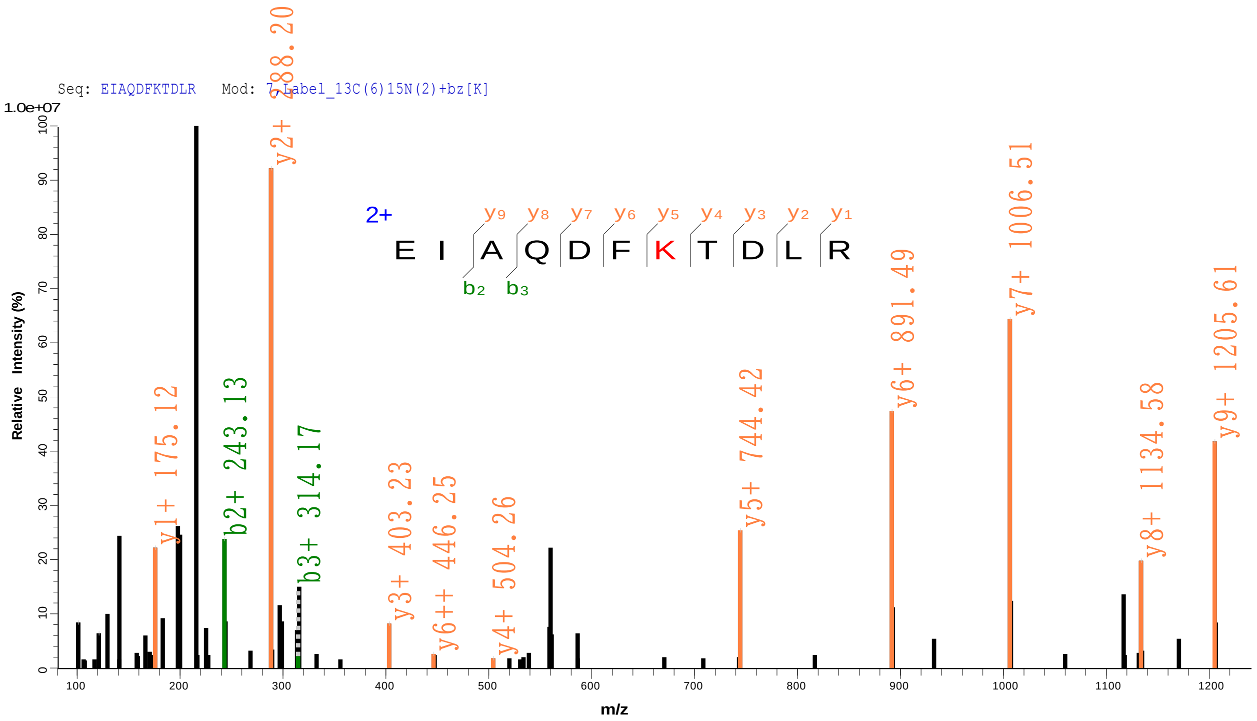

Seq: EIAQDFKTDLR Mod: 7,Label_13C(6)15N(2)+bz[K]
1.0e+07
Relative Intensity (%)
m/z
2+
E I A Q D F K T D L R
y9 y8 y7 y6 y5 y4 y3 y2 y1
b2 b3
y1+ 175.12
b2+ 243.13
y2+ 288.20
b3+ 314.17
y3+ 403.23
y6++ 446.25
y4+ 504.26
y5+ 744.42
y6+ 891.49
y7+ 1006.51
y8+ 1134.58
y9+ 1205.61

H4

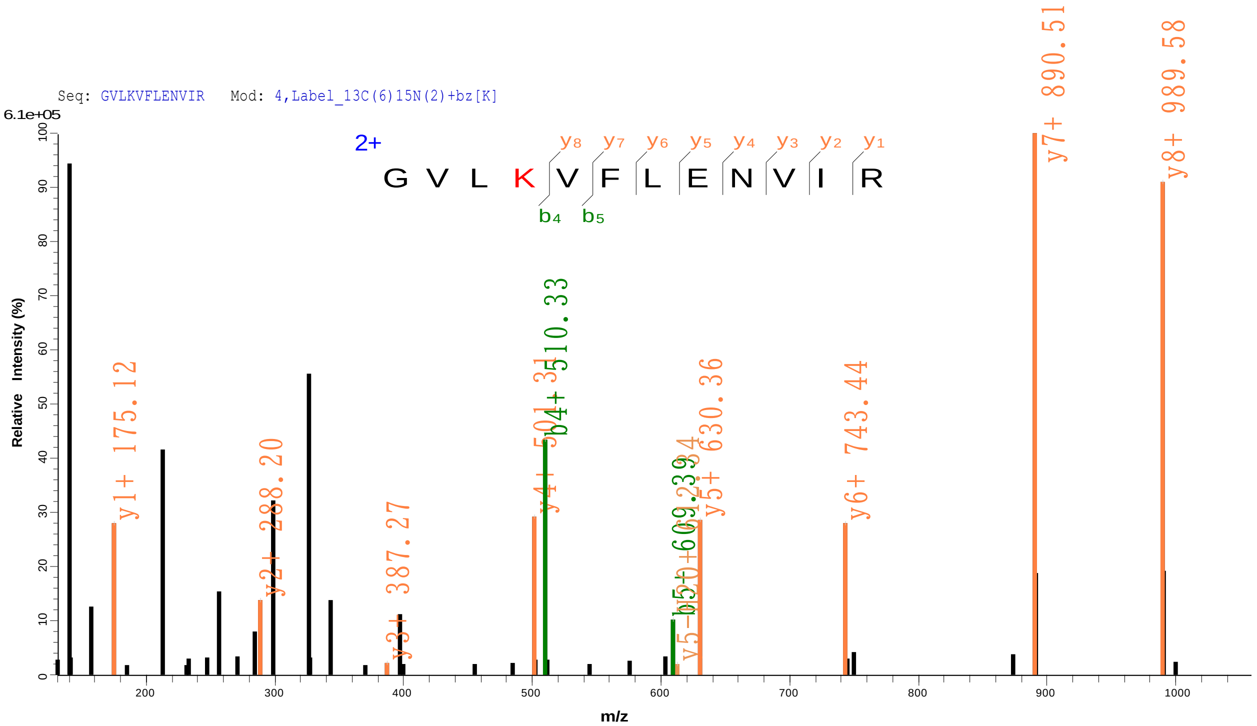

Seq: GVLKVFLENVIR Mod: 4,Label_13C(6)15N(2)+bz[K]
6.1e+05
Relative Intensity (%)
m/z
2+
G V L K V F L E N V I R
y8 y7 y6 y5 y4 y3 y2 y1
b4 b5
y1+ 175.12
y2+ 288.20
y3+ 387.27
b4+ 510.33
y5+ 630.36
y6+ 743.44
y7+ 890.51
y8+ 989.58
